# Supplementary material for: Factors associated with disease severity and mortality among patients with COVID-19: A systematic review and meta-analysis
Source: PLoS One. 2020 Nov 18;15(11):e0241541. doi: 10.1371/journal.pone.0241541 (PMC7673562; doi:10.1371/journal.pone.0241541)
Supplement: S1 Appendix — (PDF) [file pone.0241541.s001.pdf]

SUPPLEMENTARY APPENDIX

Section	Content	Page
**I**	Search Strategy	3
**II**	A) Study Characteristics that compared patients who died with those who survived (Supplementary table S1)	4
	B) Study Characteristics that compared patients with severe disease with those with non-severe disease (Supplementary table S2)	7
**III**	Quality assessment (Supplementary tables 3 and 4)	12
**IV**	Subgroup analysis based on the inclusion of only critically ill or all COVID-19 patients as study participants	17
**V**	Supplementary figures (Forest and funnel plots 1-121)	18
**VI**	Supplementary figures (Bubble plot 122-165)	147
**VII**	Definitions	192
**VIII**	References	193

Section I
Search Strategy

PubMed Search Strategy

"COVID-19" [Supplementary Concept] OR "severe acute respiratory syndrome coronavirus 2" [Supplementary Concept] OR 2019 novel coronavirus [tw] OR SARS2 [tw] OR coronavirus disease-19 OR COVID-19 [tw] OR "COVID 19"[tw] OR "COVID19"[tw] OR "COVID2019"[tw] OR "COVID 2019"[tw] OR "COVID-2019"[tw] OR "novel coronavirus"[tw] OR "new coronavirus"[tw] OR "novel corona virus"[tw] OR "new corona virus"[tw] OR "SARS-CoV-2"[tw] OR "SARSCoV2"[tw] OR "SARS-CoV2"[tw] OR "2019nCoV"[tw] OR "2019-nCoV"[tw] OR "2019 coronavirus"[tw] OR "2019 corona virus"[tw] OR "coronavirus disease 2019"[tw] OR "severe acute respiratory syndrome coronavirus 2"[nm] OR "severe acute respiratory syndrome coronavirus 2"[tw] OR "sars-coronavirus-2"[tw] OR "coronavirus disease 2019"[tw] OR "corona virus disease 2019"[tw] OR (wuhan[tw] AND (“2019/01/01”[PDAT]: “3000/12/31”[PDAT]))

Embase Search Strategy

'2019 novel coronavirus'/exp OR SARS2:ab,ti,kw OR ‘Wuhan coronavirus’:ab,ti,kw OR ‘Wuhan seafood market pneumonia virus’:ab,ti,kw OR coronavirus disease-19:ab,ti,kw OR COVID-19:ab,ti,kw OR ‘COVID 19’:ab,ti,kw OR ‘COVID19’:ab,ti,kw OR ‘COVID2019’:ab,ti,kw OR ‘COVID 2019’:ab,ti,kw OR ‘COVID-2019’:ab,ti,kw OR ‘novel coronavirus’:ab,ti,kw OR ‘new coronavirus’:ab,ti,kw OR ‘novel corona virus’:ab,ti,kw OR ‘new corona virus’:ab,ti,kw OR ‘SARS-CoV-2 ’:ab,ti,kw OR ‘SARSCoV2’:ab,ti,kw OR ‘SARS-CoV2:ab,ti,kw’ OR ‘2019nCo:ab,ti,kw V’:ab,ti,kw OR ‘2019-nCoV’:ab,ti,kw OR ‘2019 coronavirus’:ab,ti,kw OR ‘2019 corona virus’:ab,ti,kw OR ‘coronavirus disease 2019’:ab,ti,kw OR ‘severe acute respiratory syndrome coronavirus 2’:ab,ti,kw OR ‘severe acute respiratory syndrome coronavirus 2’:ab,ti,kw OR ‘sars-coronavirus-2’:ab,ti,kw OR ‘coronavirus disease 2019’:ab,ti,kw OR ‘corona virus disease 2019’:ab,ti,kw OR (wuhan:ti,ab,kw AND [2019-2020]/py)

Section II

A) Supplementary Table S1: Study characteristics for the studies that compared patients who died with those who survived.

Name of the Author	Study duration	Month and year of publication	Study design	Country of study	Study setting, City/Province	Patient characteristics	Number of participants	Age Mean (SD) / Mean*[IQR]	Male: Female	Time of laboratory assessment	Time of Radiological assessment
Xiaobo Yang(1)	24 Dec 2019 – 9 Feb 2020	Feb 2020	Retrospective Cohort	China	Jin Yin-tan hospital, Wuhan	Critically ill patients	52	59.7 (13.3)	35:17	Admission	-
Tao Chen(2)	13 Jan 2020 – 28 Feb 2020	Mar 2020	Retrospective Cohort	China	Tongji Hospital, Wuhan	Moderate to critically ill	274	62* [44 -70]	171:103	Admission	Admission
Ning Tang(3)	1 Jan 2020 – 13 Feb 2020	Feb 2020	Retrospective Cohort	China	Tongji Hospital, Wuhan	All confirmed case of COVID-19	183	54.1 (16.2)	98:85	Admission	-
Qiurong Ruan(4)	NA	Mar 2020	Retrospective Cohort	China	Jin Yin-tan hospital and Tongji hospital, Wuhan	All confirmed case of COVID-19	150	NA	NA	Timeline NA	-
Chaomin Wu(5)	25 Dec 2019 – 26 Jan 2020	Mar 2020	Retrospective Cohort	China	Jin Yin-tan hospital, Wuhan	All confirmed case of COVID-19	84	51* [43-60]	60:24	Admission	Admission
Yang-kai Li(6)	1 Jan 2020 – 3 Mar 2020	Mar 2020	Retrospective Cohort	China	Tongji Hospital, Wuhan	From Thoracic surgery department	25	48.4 (14.4)	12:13	Timeline NA	-
Yan Deng(7)	1 Jan 2020 – 21 Feb 2020	Mar 2020	Retrospective Cohort	China	Tongji Hospital, Wuhan	All confirmed case of COVID-19	225	NA	124:104	Admission	-
Mingli Yuan(8)	1 Jan 2020 – 25 Jan 2020	Mar 2020	Retrospective Cohort	China	Hubei Public Health Clinical Center, Hubei Province	All confirmed case of COVID-19	27	60* [47 - 69]	12:15	-	Admission
Fei Zhou(9)	29 Dec 2019 – 31 Jan 2020	Mar 2020	Retrospective Cohort	China	Jin Yin-tan hospital and Wuhan Pulmonary Hospital, Wuhan	All confirmed case of COVID-19	191	56* [46 - 67]	119:72	Admission	Admission
Jianlei Cao(10)	3 Jan 2020 – 1 Feb 2020	Mar 2020	Retrospective Cohort	China	Wuhan University Zhongnan Hospital, Wuhan	All confirmed case of COVID-19	102	54* [37-67]	53:49	Admission	Admission
Rong-Hui Du(11)	25 Dec 2019 – 7 Feb 2020	Mar 2020	Prospective Cohort	China	Wuhan Pulmonary Hospital, Wuhan	All confirmed case of COVID-19	179	57.6 (13.7)	97:82	Timeline NA	-
Wen-Jun Tu(12)	3 Jan 2019 – 24 Feb 2020	Apr 2020	Retrospective Cohort	China	Wuhan University Zhongnan Hospital, Wuhan	All confirmed case of COVID-19	174	70* [64-80]	79:95	Admission	-
Lang Wang(13)	1 Jan 2020 – 5 Mar 2020	Apr 2020	Retrospective Cohort	China	Renmin Hospital of Wuhan University, Wuhan	Elderly patients	339	69* [65, 76]	173:166	Timeline NA	-

Yang Wang(14)	25 Jan 2020 – 25 Feb 2020	Apr 2020	Retrospective Cohort	China	Tongji Hospital, Wuhan	Critically ill patients	344	64* [52-72]	179:165	Admission	Admission
Mengyuan Dai(15)	1 Jan 2020 - 24 Feb 2020	Apr 2020	Retrospective Cohort	China	Multicenter study, Wuhan	All confirmed case of COVID-19	641	64* [57-71]	57:48	-	-
Ming Ding(16)	1 Jan 2020 – 20 Mar 2020	Apr 2020	Retrospective Cohort	China	Huang-shi Traditional Chinese Medicine Hospital, Hubei Province	All confirmed case of COVID-19	32	63 (16)	13:19	Admission	-
Helena Barrasaa(17)	4 Mar 2020 - 31 Mar 2020	Apr 2020	Prospective Cohort	Spain	University Hospital Araba, Vitoria	ICU patients	48	63 (12)	27:21	Admission	-
TieLong Chen(18)	1 Jan 2020 - 10 Feb 2020.	Apr 2020	Retrospective Cohort	China	Zhongnan Hospital, Wuhan	All confirmed case of COVID-19	203	54* [41–68]	108:95	Admission	Admission
Hernando Trujillo(19)	NA	May 2020	Retrospective Cohort	Spain		Patients with end- stage renal disease on dialysis (n=25) and renal transplantation (n=26) who developed COVID-19	51	64(15)	29:22	Admission	Admission
Gwilym Webb (20)	Till 22 Apr 2020	Apr 2020	Retrospective Cohort	Americas, China, Japan, South Korea	Multi-center, from 21 countries	All confirmed case of COVID-19	39	58* [50-64]	NA	Admission	-
Qingchun Yao(21)	30 Jan 2020 to 11 Feb 2020	Apr 2020	Retrospective Cohort	China	Dabieshan Medical Center, Huanggang, Hubei province	All confirmed case of COVID-19	108	52 * [37-58]	43:65	Admission	Admission
Jia-Fu Wei(22)	Jan 2020 - 10 Mar 2020	Apr 2020	Prospective Cohort	China	Two designated covid-19 treatment centers in Sichuan	All confirmed COVID-19 All confirmed case of COVID-19	101	49 [34 - 62]	NA	Admission	Admission
Jennifer Tomlins(23)	10 Mar 2020 - 30 Mar 2020	Apr 2020	Retrospective cohort	United Kingdom	North Bristol NHS Trust, a large, regional teaching hospital in the UK.	All confirmed case of COVID-19	95	75* [59-82]	60:35	Timeline NA	Timeline NA
Xiaobo Yang(24)	Till 25 Feb 2020	Apr 2020	Retrospective cohort	China	Jin Yin-tan Hospital, Wuhan	All confirmed case of COVID-19	1476	57 [47-67]	776:700	Timeline NA	-
Rongrong Yang (25)	11 Jan 2020 - 16 Mar 2020	Apr 2020	Retrospective Cohort	China	Zhongnan Hospital of Wuhan University, Wuhan	All confirmed case of COVID-19	212	55.6* [40–67]	107:105	-	-
Safiya Richardson(26)	1 Mar 2020 – 4 Apr 2020	Apr 2020	Prospective Cohort	United States	Multi-center, 12 hospitals in New York City, Long Island, and Westchester County, New York, within the Northwell Health system	All confirmed case of COVID-19	5700	63* [52-75]	3437:2263	Admission	-
Jeremy Gold(27)	March 2020	Apr 2020	Prospective Cohort	United States	8 Georgia hospitals (7 in metropolitan Atlanta and 1 in Southern Georgia)	All confirmed case of COVID-19	305	60* [46–69]	151:154	-	-
Lei Gao(28)	NA	Apr 2020	Retrospective Cohort	China	Hubei general Hospital, Hubei Province	All confirmed case of COVID-19	54	60.4 (16.1)	NA	Admission	-

Shailendra Singh(29)	Till 12 Apr 2020	Apr 2020	Retrospective Cohort	United States	Multi-center, 34 health care centers across the US	All confirmed case of COVID-19	2780	52 (18)	1070:1710	-	-
Alberto Zangrillo(30)	20 Feb 2020 - 2 April 2020.	Apr 2020	Retrospective Cohort	Italy	Large tertiary hospital in Milan, Italy	Critically ill patients	73	61* [54–69]	61:12	Admission	Admission
Yongli Yan(31)	10 Jan 2020 - 24 Feb 2020	Apr 2020	Retrospective cohort	China	Tongji Hospital, Wuhan	All patients had “severe” COVID-19	193	64* [49–73]	114:79	-	-
Kun Wang (32)	7 Jan 2020 - 11 Feb 2020	May 2020	Retrospective Cohort	China	First People’s Hospital of Jiangxia district in Wuhan	All confirmed case of COVID-19	296	47 (15)	140:156	Admission	-
Ke Wang(33)	NA	Apr 2020	Ambispective Cohort	China	Unspecified hospital in Wuhan	All confirmed case of COVID-19	548		NA	Admission	-
Litao Zhang(34)	12 Jan 2020 - 15 Mar 2020	Apr 2020	Retrospective Cohort	China	Wuhan Asia General Hospital, Wuhan	All confirmed case of COVID-19	343	62* [48-69]	NA	Admission	-
Dawei Wang(35)	Till 10 Feb 2020	Apr 2020	Retrospective Cohort	China	Zhongnan Hospital of Wuhan University in Wuhan and Xishui Hospital, Hubei Province	All COVID-19 positive patients that were discharged from the two hospitals	107	51* [36–65]	57:50	Admission	-
Yanli Liu(36)	Till 1 Mar 2020	Apr 2020	Retrospective Cohort	China	Central Hospital of Wuhan, Wuhan	All confirmed case of COVID-19	383	46* [34-61]	162:221	Admission	-
Yuwei Liu(37)	1 Jan 2020 – 29 Feb 2020	Apr 2020	Retrospective Cohort	China	the Zhongnan Hospital of Wuhan University, Wuhan	All confirmed case of COVID-19	245	54 (17)	114:131	Admission	-
Juyi Li(38)	15 Jan 2020 -25 Mar 2020	Apr 2020	Retrospective Cohort	China	Central hospital of Wuhan, Hubei Province	All confirmed case of COVID-19	1178	55.5* [38-67]	545:633	Timeline NA	-
Mohamad Nikpouraghdam(39)	19 Feb 2020 - 15 Apr 2020	Apr 2020	Retrospective Cohort	Iran	Baqiyatallah Hospital in Tehran	All confirmed case of COVID-19	2964	56 (15)	1.93:1	-	-
J. Zhang(40)	11 Jan 2020 – 6 Feb 2020	Apr 2020	Retrospective Cohort	China	Renmin Hospital of Wuhan University, Wuhan	All confirmed case of COVID-19	663	56* [44-69]	321:342	Admission	Admission
Vikas Mehta(41)	18 Mar 2020 - 8 Apr 2020	May 2020	Retrospective Cohort	United States	Montefiore Health system, New York	All COVID-19 positive patients, and cancer patients (75% of patients had solid tumors and 25% with hematologic malignancies)	218	69* [Range 10-92]	127:91	Timeline NA	-
Xiaojing Zou(42)	10 Jan 2020 - 10 Feb 2020	May 2020	Retrospective Cohort	China	Tongji Hospital in Wuhan, China	All confirmed case of COVID-19	154	61 (13)	67:87	Admission	Admission

* = Mean. [..] = IQR. (..) = SD. Timeline NA= Timeline Not available.

B) Supplementary Table S2: Study characteristics for the studies that compared patients with severe disease with those with non-severe disease

Name of the Author	Study duration	Month and year of publication	Study design	Country of study	Study setting, City/Province	Number of participants	Age Mean (SD) / Mean*[IQR]	Male: Female	Time of laboratory assessment	Time of Radiological assessment	Time of Disease severity assessment	Criteria for severe disease
Wei-jie Guan(43)	11 Dec 2019 - 29 Jan 2020	Feb 2020	Retrospective cohort	China	552 hospitals in 30 provinces	1099	54.1 (16)	637:459	Admission	Admission	Admission	ATS
Yu-Huan Xu (44)	Jan 2020 - Feb 2020	Feb 2020	Retrospective cohort	China	The Fifth Medical Center of Chinese PLA General Hospital, Beijing	50	43.9 (16.8)	29:21	-	Admission	Admission	CNHC
Sijia Tian (45)	20 Jan 2020 - 10 Feb 2020	Feb 2020	Retrospective cohort	China	Beijing Emergency Medical Center, Beijing	262	47.5* [Range 1-97]	127:135	-	-	Admission	CNHC
Kunhua Li(46)	Jan 2020 - Feb 2020	Feb 2020	Retrospective cohort	China	The second affiliated Hospital of Chongqing Medical university, Chongqing	83	45.5(12.3)	44:39	Timeline NA	Timeline NA	Admission	CNHC
Wei Zhao(47)	NA	Feb 2020	Retrospective cohort	China	four institutions in Hunan province	101	44.4 (12.3)	56:45	Timeline NA	Admission	Admission	CNHC
Huan Han(48)	31 Jan 2020 - 10 Feb 2020	Feb 2020	Prospective cohort	China	Renmin Hospital of Wuhan University, Wuhan	94	NA	48:46	Timeline NA	-	Timeline NA	CNHC
Guo-Qing Qian(49)	20 Jan 2020 - 11 Feb 2020	Mar 2020	Retrospective cohort	China	Ningbo City First Hospital & many, Zhejiang	91	50* [36.5-57]	37:54	Admission	Admission	Timeline NA	CNHC
Rong Qu(50)	Jan 2020 - Feb 2020	Mar 2020	Retrospective cohort	China	Huizhou municipal central hospital, Huizhou	30	50.5* [36-65]	16:14	Admission	-	Timeline NA	CNHC
Yong Gao (51)	23 Jan 2020 - 2 Feb 2020	Feb 2020	Retrospective cohort	China	Fuyang Second People's Hospital, Fuyang	43	45.2(7.68)	9:6	Timeline NA	-	Timeline NA	CNHC
Kai-Cai Liu (52)	21 Jan 2020 - 3 Feb 2020	Feb 2020	Retrospective cohort	China	Hefei Second People's Hospital, Hufei	73	41.6 (14.5)	41:32	Timeline NA	Timeline NA	Timeline NA	CNHC
Suxin Wan (53)	23 Jan 2020 - 8 Feb 2020	Mar 2020	Retrospective cohort	China	Chongqing University Three Gorges Hospital, Chongqing	135	47* [36-55]	72:63	Admission	-	Timeline NA	CNHC
Yu Shi(54)	Up to 17 Feb 2020	Mar 2020	Retrospective cohort	China	The First Affiliated Hospital, Wenzhou	487	46(19)	259:228	Admission	Admission	Admission	CNHC

Kunwei Li (55)	18 Jan 2020 - 7 Feb 2020	Mar 2020	Retrospective cohort	China	The Fifth Affiliated Hospital of Sun Yat-sen University, Guangzhou	78	44.6(17.9)	38:40	Admission	Admission	Admission	CNHC
Chuan Qin (56)	10 Jan 2020 - 12 Feb 2020	Feb 2020	Retrospective cohort	China	Tongji Hospital, Wuhan	452	58* [47-67]	235:217	Admission	-	Admission	CNHC
Lu Huang (57)	1 Jan 2020 - 3 Feb 2020	N/A	Retrospective cohort	China	Tongji Hospital, Wuhan	126	52(15)	67:59	-	Admission	Admission	CNHC
Jin-Jin Zhang(58)	16 Jan 2020 - Feb 2020	Feb 2020	Retrospective cohort	China	No. 7 Hospital of Wuhan, Wuhan	140	57* [Range 25-87]	71:69	Admission	Admission	Admission	CNHC
Gemin Zhang (59)	16 Jan 2020 - 25 Feb 2020	Mar 2020	Retrospective cohort	China	Wuhan Xinzhou District People’s Hospital, Wuhan	95	49* [Range 39-58]	53:42	Admission	Admission	Admission	CNHC
Guang Chen(60)	Dec 2019- 27 Jan 2020	Mar 2020	Retrospective cohort	China	Tongji hospital, Wuhan	21	56* [50-65]	17:4	Admission	Admission	Admission	CNHC
Jiaojiao Chu(61)	7 Jan 2020 - 11 Feb 2020	Mar 2020	Retrospective cohort	China	Tongji hospital, Wuhan	54	39* [Range 26-73]	36:18	Admission	Admission	Admission	CNHC
Yang-kai Li(6)	1 Jan 2020 - 3 Mar 2020	Mar 2020	Retrospective cohort	China	Tongji Hospital affiliated to Tongji Medical College of Huazhong University of Science and Technology, Wuhan	25	61* [51-59]	12:13	Timeline NA	Timeline NA	Timeline NA	CNHC
Jing Yuan(62)	11 Jan 2020 - 4 Feb 2020	Mar 2020	Retrospective cohort	China	Shenzhen Third People’s Hospital, Shenzen	94	40* [Range 1-78]	42:52	Admission	-	Timeline NA	CNHC
Luwen Wang(63)	14 Jan 2020 - 13 Feb 2020	Mar 2020	Prospective cohort	China	Renmin Hospital of Wuhan University, Wuhan	116	54* [38-69]	67:49	Timeline NA	-	Timeline NA	CNHC
Huan Han(64)	1 Jan 2020 – 18 Feb 2020	Mar 2020	Retrospective cohort	China	Renmin Hospital of Wuhan University, Wuhan	273	58.8(12.1)	97:176	Admission	-	Admission	CNHC
Hangsheng Xie(65)	2 Feb 2020 - 23 Feb 2020	Mar 2020	Retrospective Cohort	China	Jinyintan Hospital, Wuhan	79	60* [Range 27-87]	44:35	Admission	Admission	Admission	CNHC
Qingzian Cai(66)	11 Jan 2020 – 6 March 2020	Mar 2020	Retrospective Cohort	China	Third People's Hospital of Shenzhen, Shenzen	298	47.5* [33-61]	145:153	Admission	Admission	Admission	CNHC
Yafei Zhang(67)	18 Jan 2020 – 22 Feb 2020	Apr 2020	Retrospective Cohort	China	Zhongnan Hospital of Wuhan University, Wuhan	115	49.52 (17.06)	49:66	Admission	-	Admission	CNHC
Wang Ling(68)	23 Jan 2020 – 29 Feb 2020	Mar 2020	Retrospective Cohort	China	Qiandongnan People’s Hospital, Kaili City, Qiandongna Miao and Dong Autonomous Prefecture, Guizhou	27	33 (13)	13:14	Admission	Admission	Admission	CNHC
Xinyi Chen(69)	26 Jan 2020 – 31 Jan 2020	Apr 2020	Retrospective Cohort	China	Chongqing Three Gorges Central Hospital, Chongqing	78	45* [Range 15-79]	39:39	Timeline NA	Timeline NA	Admission	CNHC
Fang Zheng(70)	17 Jan 2020 – 7 Feb 2020	Mar 2020	Retrospective Cohort	China	Changsha First Hospital, Changsha	161	45* [33.5-57]	80:81	Admission	Admission	Admission	CNHC
Qing Deng(71)	6 Jan 2020 – 20 Feb 2020	Mar 2020	Retrospective Cohort	China	Renmin Hospital of Wuhan University, Wuhan	112	65* [49-70.7]	57:55	Admission	-	Admission	CNHC

Huan Li (72)	18 Jan 2020 - 26 Feb 2020	Apr 2020	Retrospective Cohort	China	Tianyou hospital, Wuhan	132	64 (13)	75:57	Admission	Admission	Admission	CNHC
Ying Zou(73)	20 Jan 2020 - 24 Feb 2020	Apr 2020	Retrospective Cohort	China	Shanghai public health clinical center, Shanghai	303	51* [Range 16-88]	158:145	Admission	-	Admission	CNHC
Juyi Li(38)	15 Jan 2020 - 15 Mar 2020	Apr 2020	Retrospective cohort	China	Central Hospital of Wuhan, Wuhan	362	66* [59-73]	189:173	Admission	-	Timeline NA	CNHC
Fengjuan Shi(74)	Up to 4 Feb 2020	Apr 2020	Retrospective cohort	China	Multicenter,Jiangsu Province of China	114	43.5* [Range 6-79]	59:55	Admission	-	Admission	CNHC
Guyi Wang(75)	17 Jan 2020 – 20 Feb 2020	N/A	Retrospective cohort	China	The Public Health Treatment Center of Changsha, Changsha	209	54* [Range 35-68]	105:104	Admission	Admission	Admission	CNHC
Ji Mengyao (76)	2 Jan 2020 – 28 Jan 2020	May 2020	Retrospective cohort	China	Wuhan University Renmin Hospital, Wuhan	101	51* [37-61]	48:53	Admission	Admission	Admission	CNHC
Liang Shen(77)	Jan 2020 - Feb 2020	May 2020	Retrospective cohort	China	Xiangyang Central Hospital, Xiangyang City, Hubei Province	119	49* [38-61]	56:63	Admission	-	Timeline NA	CNHC
Fan Yang(78)	1 Jan 2020 - 15 Apr 2020	May 2020	Retrospective cohort	China	Renmin Hospital of Wuhan University, Wuhan	52	63* [Range 34-98]	28:24	Admission	Admission	Admission	CNHC
Jia Ma(79)	1 Jan 2020 – 30 Mar 2020	Apr 2020	Retrospective cohort	China	Hubei Cancer Hospital, Tongji Medical College, Wuhan	34	62* [59-70]	20:17	Admission	-	Timeline NA	CNHC
Qingqing Chen(80)	1 Jan 2020 – 11 Mar 2020	Apr 2020	Retrospective cohort	China	Taizhou Public Health Medical Center, Zhejiang	145	47.5 (14.6)	79:66	Admission	Admission	Admission	CNHC
Marta Colaneri (81)	21 Feb 2020 - 28 Feb 2020	Apr 2020	Retrospective cohort	Italy	Pavia	44	47.5* [Range 10-94]	28:16	Admission	Admission	Admission	ATS
Xin-Ying Zhao(82)	16 Jan 2020 - 10 Feb 2020	Apr 2020	Retrospective cohort	China	Jingzhou central hospital, Jingzhou	90	46*	49:42	Admission	-	Timeline NA	CNHC
Lian Chen(83)	8 Dec 2019 - 20 Mar 2020	Apr 2020	Retrospective cohort	China	National Health Commission of China	118	31* [28-34]	0:118	Admission	Admission	Timeline NA	CNHC
Lijun Sun(84)	20 Jan 2020 - 15 Feb 2020	May 2020	Retrospective cohort	China	Beijing 302 hospital, Beijing	55	44* [34-56]	31:24	Admission	Admission	Admission	CNHC
Zhe Zhu(85)	23 Jan 2020 - 20 Feb 2020	Apr 2020	Retrospective cohort	China	Hwa Mei Hospital, University of Chinese Academy of Sciences, Ningbo	127	50.90 (15.26)	45:82	Admission	-	Timeline NA	CNHC
Ming Ding(16)	1 Jan 2020 – 20 Mar 2020	Apr 2020	Retrospective cohort	China	Huangshi Traditional Chinese Medicine Hospital, Huangshi City, Hubei Province	32	NA	NA	Admission	-	Admission	CNHC
Yang Liu(86)	Jan 2020 - Feb 2020	Apr 2020	Retrospective cohort	China	First Affiliated Hospital of Nanchang University, Nanchang	76	45* [Range 18-78]	49:27	Admission	-	Admission	CNHC
Yun Feng(87)	1 Jan 2020 – 15 Feb 2020	Apr 2020	Retrospective cohort	China	Three Hospitals from Wuhan, Shanghai, Anhui province	476	53* [40-64]	271:205	Admission	Admission	Timeline NA	CNHC
Rui Zhang(88)	10 January 2020 - 10 Feb 2020	Apr 2020	Retrospective cohort	China	Renmin Hospital of Wuhan University, Wuhan	120	45.4 (15.6)	43:77	Admission	Admission	Admission	CNHC
Xiaohua Chen(89)	1 Feb 2020 – 19 Feb 2020	Apr 2020	Retrospective cohort	China	General Hospital of Central Theater Command, PLA, Beijing	48	64.6 (18.1)	37:11	Admission	-	Timeline NA	CNHC

Xiuqi Wei(90)	13 Feb 2020 – 3 Mar 2020	Apr 2020	Retrospective cohort	China	Union Hospital of Tongji Medical College, Wuhan, Hubei Province	252	64.8 (13.3)	130 :122	Timeline NA	-	Timeline NA	ATS
Zheng Yi(91)	16 Jan 2020 – 4 Feb 2020	Apr 2020	Retrospective cohort	China	Taihe Hospital, Shiyan	73	43 [Range 21-76]	40: 33	Admission	-	Timeline NA	CNHC
Ruirui Wang(92)	20 Jan 2020 - 9 Feb 2020	Apr 2020	Retrospective cohort	China	NO.2 People's Hospital of Fuyang City, Fuyang city	125	38.8 (13.8)	71: 54	Admission	Admission	Timeline NA	CNHC
Zhixian Yao(93)	1 Jan 2020 – 6 Feb 2020	May 2020	Retrospective cohort	China	Jinyintan Hospital, Wuhan	463	51* [43-60]	244: 219	-	-	Timeline NA	CNHC
Fang Lei(94)	20 Dec 2019 - 8 Mar 2020	May 2020	Retrospective cohort	China	Multcenter, Wuhan	5771	56* [43-65]	2724: 3047	Admission	Admission	Admission	CNHC
Lu Lu(95)	18 Jan 2020 – 18 Feb 2020	Apr 2020	Retrospective cohort	China	Multicenter study - 42 officially designed hospitals in Hubei province	304	44* [33,59.25]	182:122	Timeline NA	-	Timeline NA	CNHC
Yang Yang(96)	NA	Apr 2020	Retrospective cohort	China	Shenzhen Third People's Hospital, Shenzen	50	62*[Range 22-78]	29:21	Admission	-	Timeline NA	CNHC
Xiaojie Bi(97)	23 Jan 2020 – 4 Feb 2020	Apr 2020	Retrospective cohort	China	Taizhou Hospital, Zhejiang Province	91	44* (36–54)	51:40	Admission	Admission	Timeline NA	CNHC
Fang Liu(98)	18 Jan 2020 – 12 Mar 2020	Apr 2020	Retrospective cohort	China	General Hospital of Central Theater Command of People's Liberation Army, Wuhan	140	65.5* (54.3-73)	49:91	Admission	Admission	Timeline NA	CNHC
Guangchang Pei (99)	Up to 9 Feb 2020	Apr 2020	Retrospective cohort	China	Tongji Medical College, Huazhong University of Science and Technology, Wuhan	333	56.3 (13.4)	182:151	Admission	Timeline NA	Admission	CNHC
Qingchun Yao(21)	30 Jan 2020 – 11 Feb 2020	Apr 2020	Retrospective cohort	China	Dabieshan Medical Center, Huanggang city, Hubei Province	108	52 (37 – 58)	43:65	Admission	Admission	Timeline NA	ATS
Ling Hu(100)	8 Jan 2020 – 20 Feb 2020	May 2020	Retrospective cohort	China	Tianyou Hospital, an affiliate of the Wuhan University of Science and Technology, Wuhan	323	61* [Range 29-91]	166:157	Admission	Admission	Admission	CNHC
Xiaofan Liu(101)	5 Feb 2020 - 14 Mar 2020	Apr 2020	Retrospective cohort	China	Renmin Hospital of Wuhan University, Wuhan	99	NA	NA	Timeline NA	-	Timeline NA	CNHC
Zhang J (40)	11 Jan 2020 – 6 Feb 2020	Apr 2020	Retrospective cohort	China	Renmin Hospital of Wuhan University, Wuhan	663	55.6* [44-69]	321:342	Admission	Admission	Admission	CNHC
Yuan-Yuan Wei(102)	N/A	Apr 2020	Retrospective cohort	China	Multicenter, Anhui Province	167	42.31 (15.29)	95 :72	Timeline NA	-	Timeline NA	CNHC
Xiaochen Li(103)	26 Jan 2020 - 5 Feb 2020	Apr 2020	Retrospective cohort	China	Sino-French New City Branch of Tongji Hospital, Huazhong University of Science and Technology, Wuhan	548	60* [48-69]	279:269	Admission	Admission	Admission	ATS/CNHC
Yufen Zheng(104)	17 Jan 2020 - 26 Feb 2020	Apr 2020	Retrospective cohort	China	Taizhou Public Health Medical Center, Taizhou Hospital, Taizhou, Zhejiang Province	141	47* [38-56]	74:67	Admission	-	Admission	CNHC
Feng Wang (105)	Up to Jan 2020	May 2020	Retrospective cohort	China	Tongji Hospital, Wuhan	65	57.1 (13.01)	37:28	Timeline NA	-	Timeline NA	CNHC

Peijie Lyu (106)	15 Jan 2019 - 24 Feb 2020	Apr 2020	Retrospective cohort	China	The First Affiliated Hospital of Zhengzhou University, Zhengzhou, Henan Province	51	54 (17)	29:22	-	Timeline NA	Timeline NA	CNHC
Rui Liu(107)	31 Jan 2020 – 26 Feb 2020	Mar 2020	Retrospective cohort	China	Renmin Hospital, Wuhan	119	63.3 (11.7)	62:57	Admission	-	Admission	CNHC
H Hou(108)	24 Jan 2020 – 15 Feb 2020	Apr 2020	Retrospective cohort	China	Tongji Hospital, Wuhan.	389	61·3 (13·8)	200:189	Admission	-	Admission	CNHC
Shufa Zheng(109)	19 Jan 2020 - 20 Mar 2020	Apr 2020	Retrospective cohort	China	First Affiliated Hospital, College of Medicine, Zhejiang University, Hangzhou	96	55* [44.3 – 64.8]	58:38	-	Admission	Admission	CNHC

ATS= American thoracic society guidelines(110). CNHC= Chinese National Health Commission guidelines(111).
PLA=People's Liberation Army. Timeline NA= Timeline Not available.
* = Mean. [ ] = IQR. ( ) = SD.

Section III

Quality assessment

Supplementary Table S3: Quality assessment for studies that compared patients who died with those who survived.

Name of the Author	New Castle-Ottawa Quality assessment Scale (Total score=9)							
	Representativeness of exposed	Selection of the non-exposed	Ascertainment of exposure	Outcome of interest was not present at the start	Comparability	Assessment of outcome	Long enough follow up	Adequacy of follow up
Xiaobo Yang(1)	*	*	*	*	*	*	*	*
Tao Chen(2)	*	*	*	*	*	*	*	*
Ning Tang(3)	*	*	*	*	*	*	*	*
Qiurong Ruan(4)	*	*	*	*	*	*	*	*
Chaomin Wu(5)	*	*	*	*	*	*	*	*
Yang-kai Li(6)	*			*		*	*	*
Yan Deng(7)	*	*	*	*	*	*	*	*
Mingli Yuan(8)	*	*	*	*	*	*	*	
Fei Zhou(9)	*	*	*	*	*	*	*	
Jianlei Cao(10)	*	*	*	*	*	*	*	*
Rong-Hui Du(11)	*	*	*	*	*	*	*	
Wen-Jun Tu(12)	*	*	*	*	*	*	*	*
Lang Wang(13)	*	*	*	*	*	*	*	*
Yang Wang(14)	*	*	*	*	*	*	*	*
Mengyuan Dai(15)	*	*	*	*	**	*	*	*
Ming Ding(16)			*	*	*	*	*	*
Helena Barrasaa(17)	*		*	*	*	*	*	*

TieLong Chen(18)	*	*	*	*	*	*	*	
Hernando Trujillo(19)	*		*	*	*	*	*	
Gwilym Webb (20)	*	*	*	*	*	*	*	*
Qingchun Yao(21)	*			*	*	*		
Jia-Fu Wei(22)	*	*	*	*	*	*	*	*
Jennifer Tomlins(23)	*	*	*	*	*	*	*	*
Xiaobo Yang(24)	*	*	*	*	*	*	*	*
Rongrong Yang (25)	*	*	*	*	*	*	*	*
Safiya Richardson(26)	*		*	*	*	*	*	*
Jeremy Gold(27)	*	*	*	*	*	*	*	*
Lei Gao(28)	*	*	*	*	*	*	*	*
Shailendra Singh(29)	*	*	*	*	*			
Alberto Zangrillo(30)	*	*	*	*	*	*	*	*
Yongli Yan(31)	*	*	*	*	*	*	*	*
Kun Wang (32)	*		*	*	*	*	*	*
Ke Wang(33)	*		*	*	*	*	*	*
Litao Zhang(34)	*		*	*	*	*	*	*
Dawei Wang(35)	*	*	*	*	*	*	*	*
Yanli Liu(36)	*	*	*	*	*	*	*	*
Yuwei Liu(37)	*		*	*	*	*	*	*
Juyi Li(38)	*	*	*	*	*	*	*	*
Mohamad Nikpouraghdam(39)	*		*	*	*	*	*	*
J. Zhang(40)	*		*		*			*
Vikas Mehta(41)	*	*	*	*	*	*	*	*
Xiaojing Zou(42)	*	*	*	*	*	*	*	*

Supplementary Table S4: Quality assessment of studies that compared patients with severe disease with those with non-severe disease

Name of the Author	New Castle-Ottawa - Quality assessment Scale (Total score=9)							
	Representativeness of exposed	Selection of the non-exposed	Ascertainment of exposure	Outcome of interest was not present at the start	Comparability	Assessment of outcome	Long enough follow up	Adequacy of follow up
Wei-jie Guan(43)	*	*	*	*	*	*	*	*
Yu-Huan Xu (44)	*	*	*	*	*	*	*	*
Sijia Tian (45)	*	*	*	*	*	*	*	*
Kunhua Li(46)	*		*	*	*	*	*	*
Wei Zhao(47)	*	*	*	*	*	*	*	*
Huan Han(48)			*	*	*	*	*	*
Guo-Qing Qian(49)	*	*	*	*	*	*	*	
Rong Qu(50)	*	*	*	*	*	*	*	
Yong Gao (51)	*	*	*	*	*	*	*	*
Kai-Cai Liu (52)	*	*	*	*	*	*	*	
Suxin Wan (53)	*	*	*	*	*	*	*	
Yu Shi(54)	*	*	*	*	*	*	*	*
Kunwei Li (55)	*	*	*	*	*	*	*	*
Chuan Qin (56)	*	*	*	*	*	*	*	*
Lu Huang (57)	*	*	*	*	*	*	*	
Jin-Jin Zhang(58)	*	*	*	*	*	*	*	*
Gemin Zhang (59)	*	*		*	*	*		
Guang Chen(60)	*	*	*	*	*	*	*	*

Jiaojiao Chu(61)	*	*	*	*	*	*	*	
Yang-kai Li(6)	*			*		*	*	*
Jing Yuan(62)	*	*	*	*	*	*	*	
Luwen Wang(63)	*	*	*	*	*	*	*	
Huan Han(64)	*	*	*	*	*	*	*	*
Hangsheng Xie(65)	*	*	*		*	*	*	*
Qingzian Cai(66)	*	*		*	*	*		
Yafei Zhang(67)	*	*	*		*		*	*
Wang Ling(68)	*	*	*		*		*	*
Xinyi Chen(69)	*	*	*	*	*	*	*	*
Fang Zheng(70)	*	*	*		*	*	*	*
Qing Deng(71)	*	*	*		*	*	*	*
Huan Li (72)	*	*	*		*	*	*	*
Ying Zou(73)	*	*	*	*	*	*	*	*
Juyi Li(38)	*	*	*	*	*	*	*	*
Fengjuan Shi(74)	*	*	*	*	*	*	*	
Guyi Wang(75)	*	*	*	*	*	*	*	*
Ji Mengyao (76)	*	*	*	*	*	*	*	
Liang Shen(77)	*	*	*	*	*	*	*	*
Fan Yang(78)	*	*	*	*	*	*	*	*
Jia Ma(79)	*	*	*	*	*	*	*	*
Qingqing Chen(80)	*	*	*	*	*	*	*	*
Marta Colaneri (81)	*	*	*	*	*	*	*	*
Xin-Ying Zhao(82)	*	*	*	*	*	*	*	
Lian Chen(83)	*	*	*	*	*	*	*	*
Lijun Sun(84)	*	*	*	*	*	*	*	
Zhe Zhu(85)	*	*	*	*	*	*	*	
Ming Ding(16)	*	*	*	*	*	*	*	
Yang Liu(86)	*	*	*	*	*	*	*	*
Yun Feng(87)	*	*	*	*	*	*	*	*
Rui Zhang(88)	*			*	*			*

Xiaohua Chen(89)	*	*	*	*	*	*	*	*
Xiuqi Wei(90)	*	*	*	*	*	*	*	
Zheng Yi(91)	*	*	*	*	*	*	*	*
Ruirui Wang(92)	*	*	*	*	*	*	*	
Zhixian Yao(93)	*	*	*	*	*	*	*	*
Fang Lei(94)	*	*	*	*		*	*	*
Lu Lu(95)	*	*	*	*	*	*	*	*
Yang Yang(96)	*	*		*	*	*		
Xiaojie Bi(97)	*	*	*	*	*	*	*	*
Fang Liu(98)	*	*	*	*	*	*	*	*
Guangchang Pei (99)	*	*	*	*	*	*	*	
Qingchun Yao(21)	*			*	*	*		
Ling Hu(100)	*	*	*	*	*	*	*	
Xiaofan Liu(101)	*	*	*	*	*	*	*	
Zhang J (40)	*	*	*	*	*	*	*	
Yuan-Yuan Wei(102)	*	*	*	*	*	*	*	*
Xiaochen Li(103)	*	*	*	*		*	*	
Yufen Zheng(104)	*	*	*	*	*	*	*	*
Feng Wang (105)	*	*	*	*	*	*	*	
Peijie Lyu (106)	*	*	*	*	*	*	*	
Rui Liu(107)	*	*	*	*	*	*	*	
H Hou(108)	*	*	*	*	*	*	*	*
Shufa Zheng(109)	*	*	*	*	*	*	*	

Section IV

Supplementary Table S5: Subgroup analysis based on whether critically ill patients were included in the study

Clinical characteristics	All studies			Studies with all COVID-19 patients			Studies with only critically ill COVID-19 patients		
	No. of Studies	Pooled RR [95% CI]	I^2	No. of Studies	Pooled RR [95% CI]	I^2	No. of Studies	Pooled RR [95% CI]	I^2
Cardiovascular diseases	25	2.27 [1.88–2.79]	70.78%	22	2.47[2.06–2.95]	63.76%	3	1.27 [0.90–1.80]	20.87%
Cerebrovascular Disease	15	2.63 [1.97–3.51]	75.22%	14	2.76[2.02–3.75]	73.28%	1	1.69[1.23–2.32]	··
Diarrhea	14	1.15 [0.85–1.57]	64.65%	13	1.23[0.8–1.70]	60.55%	1	0.76[0.55–1.07]	··
Dyspnea	20	2.55 [1.88–3.46]	77.13%	16	3.17[2.32 –4.32]	63.89%	4	1.38 [0.98–1.94]	51.88%
Shock	9	6.12 [3.59–10.45]	93.35%	8	5.66 [3.15–10.15]	93.57%	1	10.76 [7.15–16.19]	··
Bacteremia	6	5.07 [2.02–12.67]	94.28%	5	6.55 [2.60–16.54]	93.92%	1	1.24 [0.54–2.84]	··
Acute cardiac injury	14	5.42 [3.79–7.77]	86.24 %	14	5.82 [4.40–7.71]	69.79%	2	3.37 [0.53–21.47]	97.74%
Acute Respiratory Distress Syndrome	14	20.19 [10.87–37.52]	79.05%	12	22.76 [13.07–39.66]	63.32%	2	8.48 [0.54–133.79]	96.04%
Acute Kidney Injury	15	4.65 [3.25–6.65]	94.89%	13	5.07 [3.49–7.37]	94.38%	2	2.70 [0.85–8.59]	96.06%
Acute liver injury	10	2.54 [1.77–3.66]	93.14%	8	2.84 [1.94–4.17]	91.17%	2	1.67 [0.60–4.64]	93.37%
Disseminated intravascular coagulation	4	3.41 [2.00–5.81]	94.86%	3	3.17 [1.53–6.57]	95.98%	1	4.27 [3.43–5.31]	··
Gastrointestinal bleeding	4	2.53 [1.42–4.49]	76.21%	3	3.13 [1.79–5.46]	64.09%	1	1.39 [0.80–2.42]	··
TLC > 10 x 109/L	5	4.72 [2.69–8.30]	86.80%	5	4.72 [2.69–8.30]	86.80%	··	··	··
Na+ > 145 mmol/L	2	2.70 [2–3.64]	63.06%	2	2.70 [2–3.64]	63.06%	··	··	··
Procalcitonin >0.5 ng/mL	6	2.44 [1.40–4.26]	92.33%	5	3.39 [2.62–4.39]	43.43%	1	0.88 [0.69–1.12]	··
BNP > 100 ng/L	2	14.65 [0.49–440.36]	90.98%	2	14.65 [0.49–440.36]	90.98%	··	··	··

Section V

Supplementary figures

The following symbols in the forest plots denote the primary hospital where the study was conducted

! Shenzhen Third People's Hospital, Shenzhen, China

Tongji Hospital, Wuhan, China

$ Wuhan Jinyintan Hospital, Wuhan, China

* Zhongnan Hospital of Wuhan University, Wuhan, China

% Tianyou Hospital, Wuhan, China

& Taizhou Public Health Medical Center, Zhejiang, China; Central hospital of Wuhan, Wuhan

+ Chongqing Three Gorges Central Hospital, Chongqing, China

@ Renmin Hospital of Wuhan University, Wuhan, China

^ General Hospital of Central Theater Command of People's Liberation Army, Wuhan, China

eFigure 1. Pooled Risk ratio of death in male patients compared to female patients. a) Forest plot b) Funnel Plot

1.a

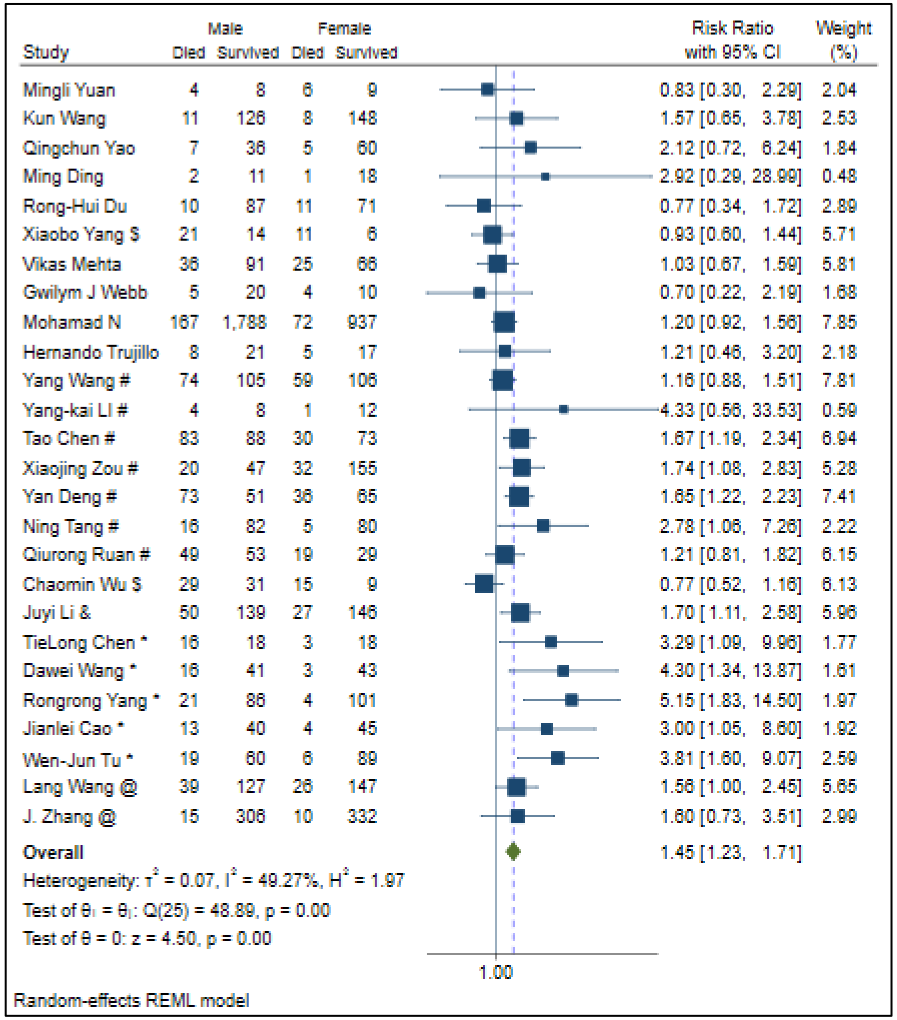

1.b

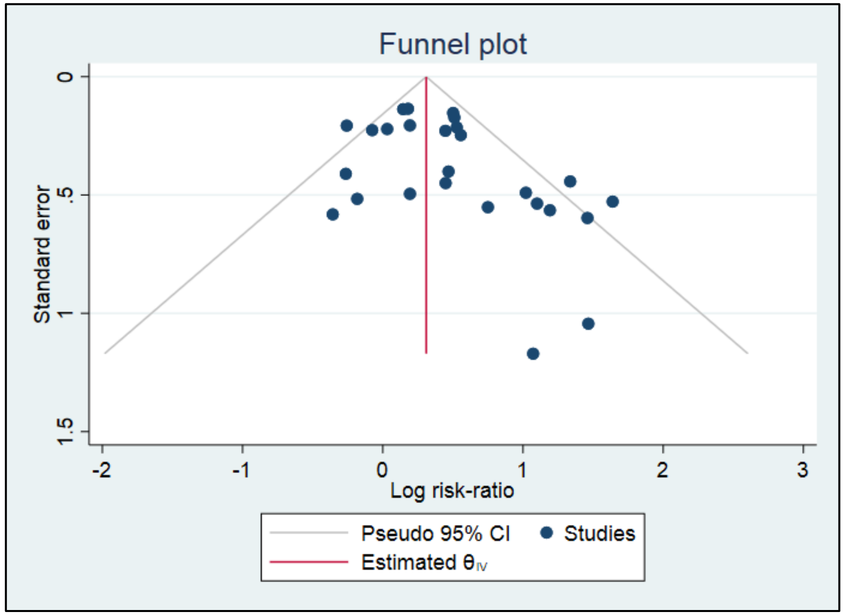

eFigure 2. Pooled Risk ratio of death in ever smoker patients. a) Forest plot

2a

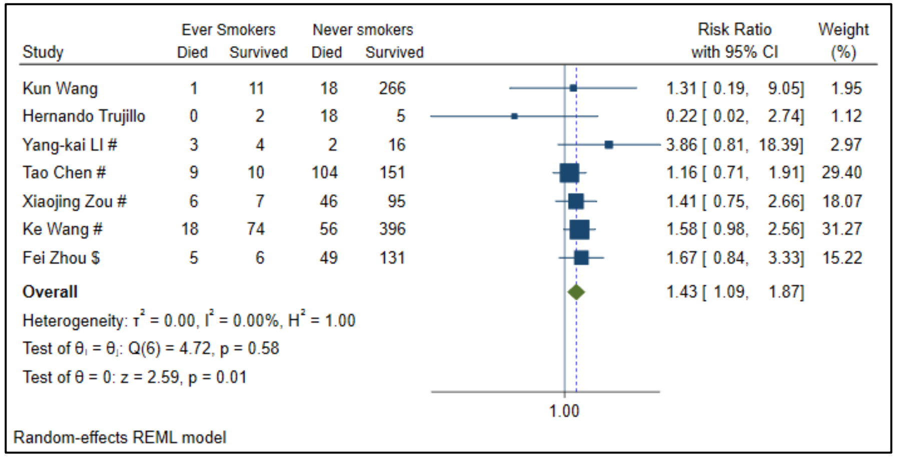

eFigure 3. Pooled Risk ratio of death in patients with Diabetes Mellitus (DM). a) Forest plot b) Funnel Plot

3a

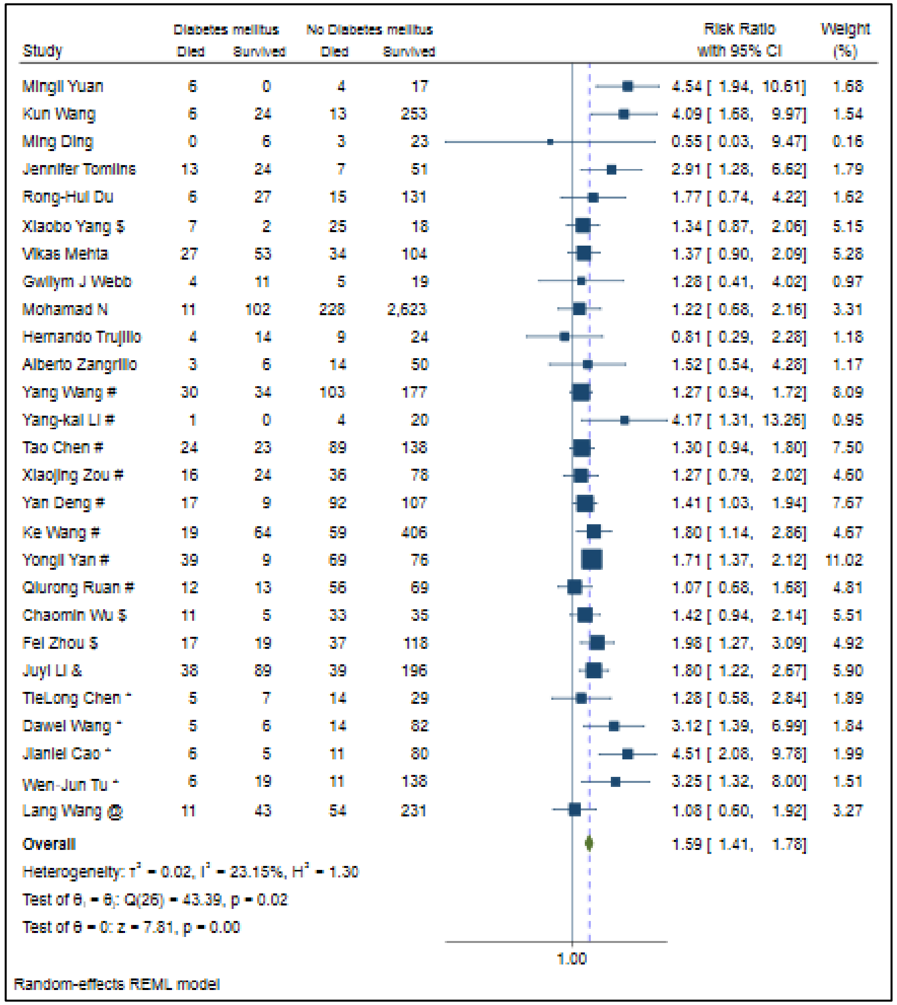

3b

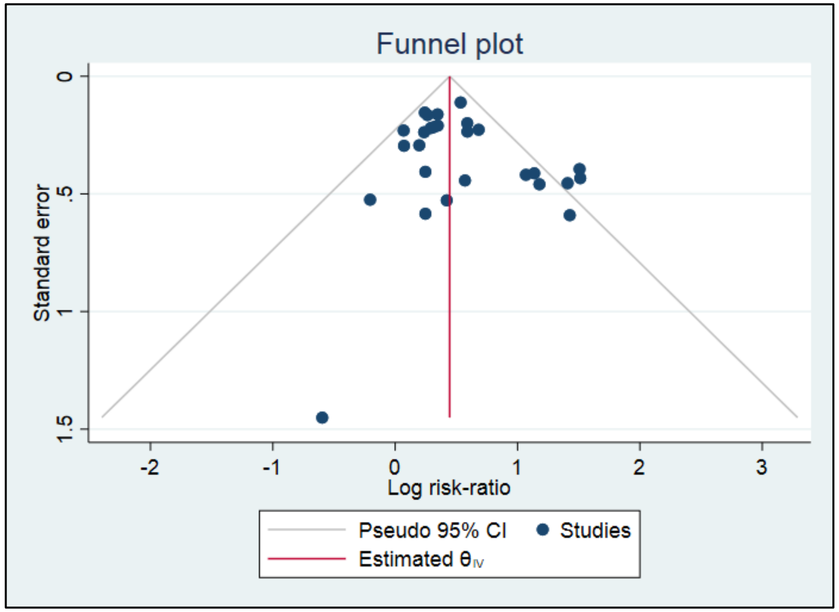

eFigure 4. Pooled Risk ratio of death in patients with Hypertension (HTN). a) Forest plot b) Funnel Plot

4a

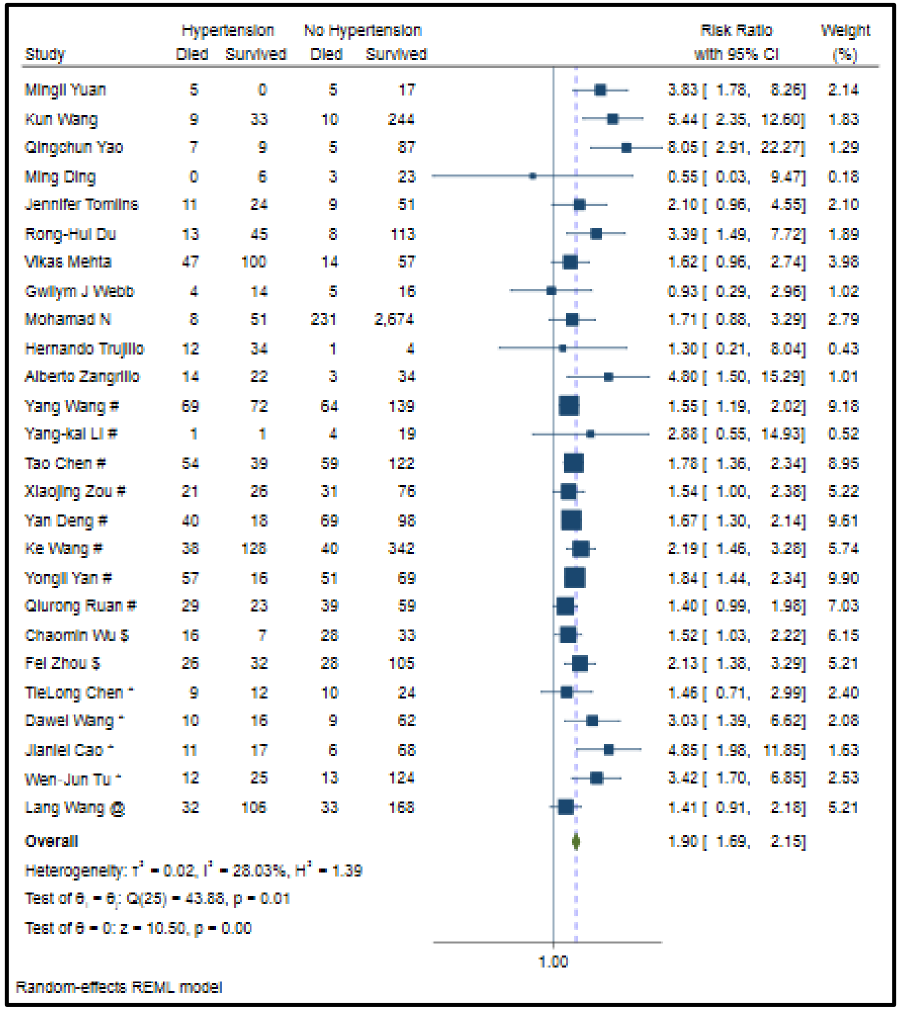

4b

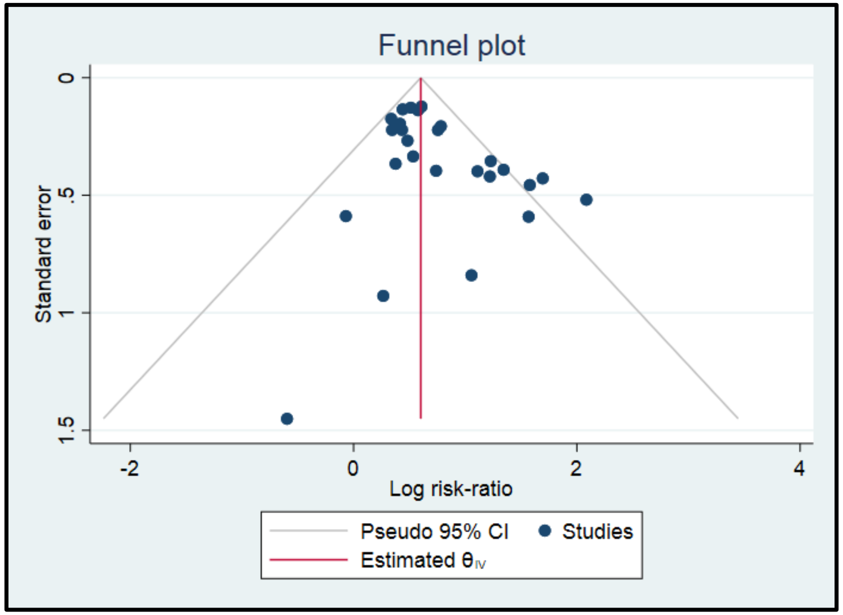

eFigure 5. Pooled Risk ratio of death in patients with Cardiovascular disease a) Forest plot b) Funnel Plot c) Subgroup analysis

5a

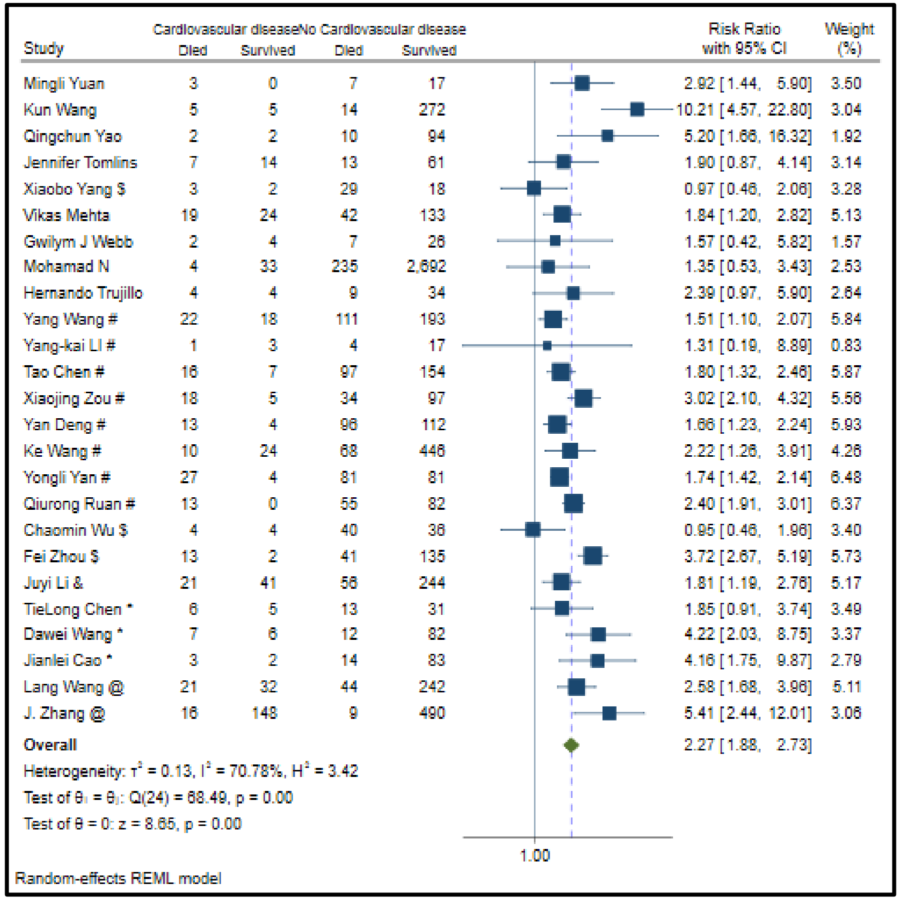

5b

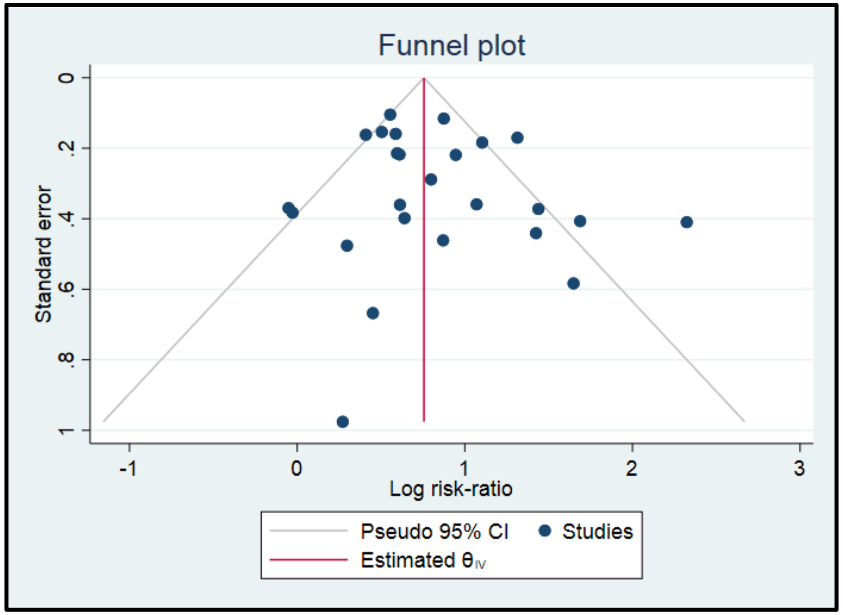

5c

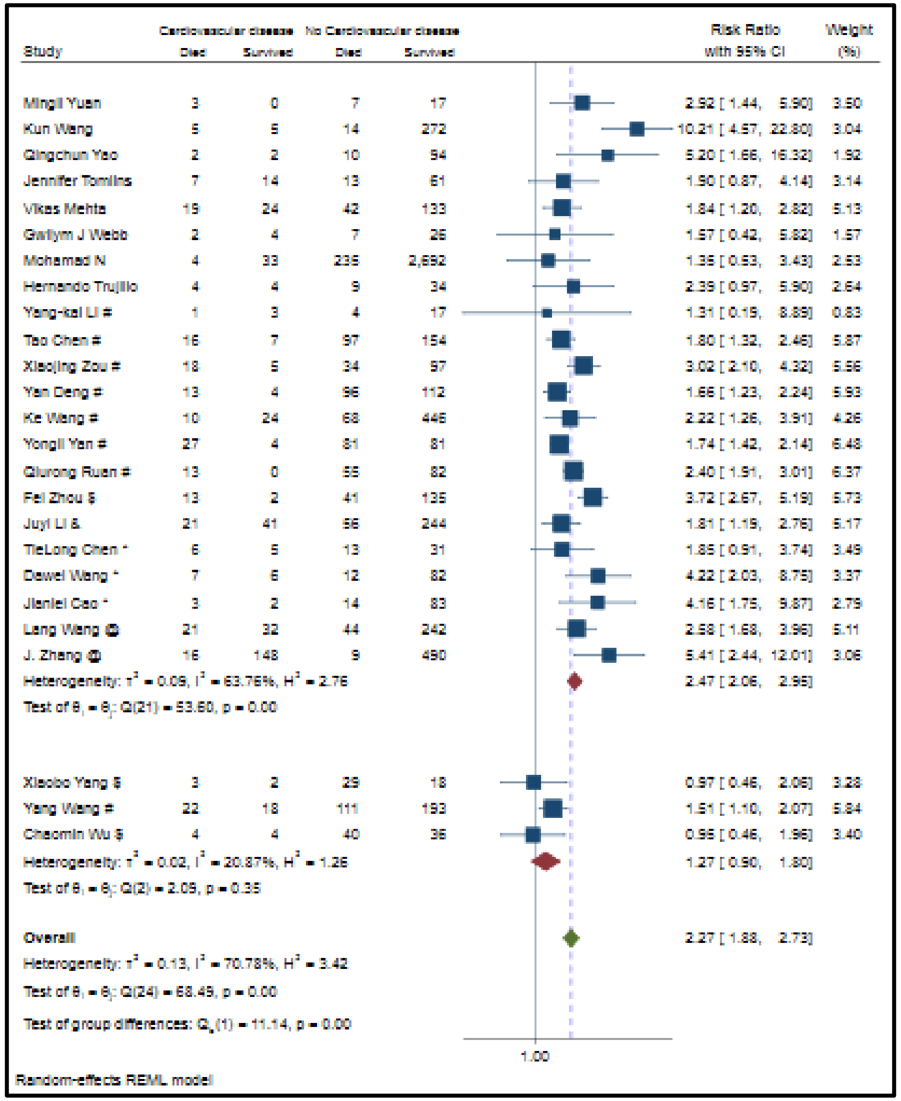

Cardiovascular disease
No Cardiovascular disease
Study
Died
Survived
Died
Survived
Risk Ratio with 95% CI
Weight (%)
Mingli Yuan 3 0 7 17 2.92 [1.44, 5.90] 3.50
Kun Wang 5 5 14 272 10.21 [4.57, 22.80] 3.04
Qingchun Yao 2 2 10 94 5.20 [1.66, 16.32] 1.92
Jennifer Tomlins 7 14 13 61 1.90 [0.87, 4.14] 3.14
Vikas Mehta 15 24 42 133 1.84 [1.20, 2.82] 5.13
Gwilym J Webb 2 4 7 26 1.57 [0.42, 5.82] 1.57
Mohamad N 4 33 235 2,692 1.35 [0.53, 3.43] 2.53
Hernando Trujillo 4 4 9 34 2.39 [0.97, 5.90] 2.64
Yang-kai Li # 1 3 4 17 1.31 [0.19, 8.85] 0.83
Tao Chen # 16 7 97 154 1.80 [1.32, 2.46] 5.87
Xiaojing Zou # 18 5 34 97 3.02 [2.10, 4.32] 5.56
Yan Deng # 13 4 96 112 1.66 [1.23, 2.24] 5.93
Ke Wang # 10 24 68 446 2.22 [1.26, 3.91] 4.26
Yongli Yan # 27 4 81 81 1.74 [1.42, 2.14] 6.48
Qiurong Ruan # 13 0 55 82 2.40 [1.91, 3.01] 6.37
Fei Zhou $ 13 2 41 135 3.72 [2.67, 5.19] 5.73
Juyi Li & 21 41 56 244 1.81 [1.19, 2.76] 5.17
TieLong Chen * 6 5 13 31 1.85 [0.91, 3.74] 3.49
Dawei Wang * 7 6 12 82 4.22 [2.03, 8.75] 3.37
Jianlei Cao * 3 2 14 83 4.16 [1.75, 9.87] 2.79
Lang Wang @ 21 32 44 242 2.58 [1.68, 3.96] 5.11
J. Zhang @ 16 148 9 490 5.41 [2.44, 12.01] 3.06
Heterogeneity: τ² = 0.09, I² = 63.76%, H² = 2.76 2.47 [2.06, 2.95]
Test of θi = θj: Q(21) = 53.60, p = 0.00
Xiaobo Yang $ 3 2 29 18 0.97 [0.46, 2.06] 3.28
Yang Wang # 22 18 111 193 1.51 [1.10, 2.07] 5.84
Chaomin Wu $ 4 4 40 36 0.95 [0.46, 1.96] 3.40
Heterogeneity: τ² = 0.02, I² = 20.87%, H² = 1.26 1.27 [0.90, 1.80]
Test of θi = θj: Q(2) = 2.05, p = 0.35
Overall 2.27 [1.88, 2.73]
Heterogeneity: τ² = 0.13, I² = 70.78%, H² = 3.42
Test of θi = θj: Q(24) = 68.49, p = 0.00
Test of group differences: Qb(1) = 11.14, p = 0.00
1.00
Random-effects REML model

eFigure 6. Pooled Risk ratio of death in patients with Congestive Heart Failure a) Forest plot

6a

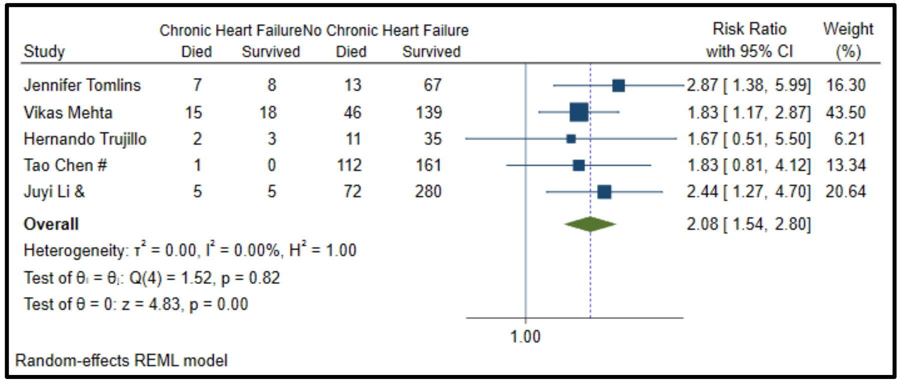

eFigure 7. Pooled Risk ratio of death in patients with Cerebrovascular diseases a) Forest plot b) Funnel Plot c) Subgroup analysis

7a

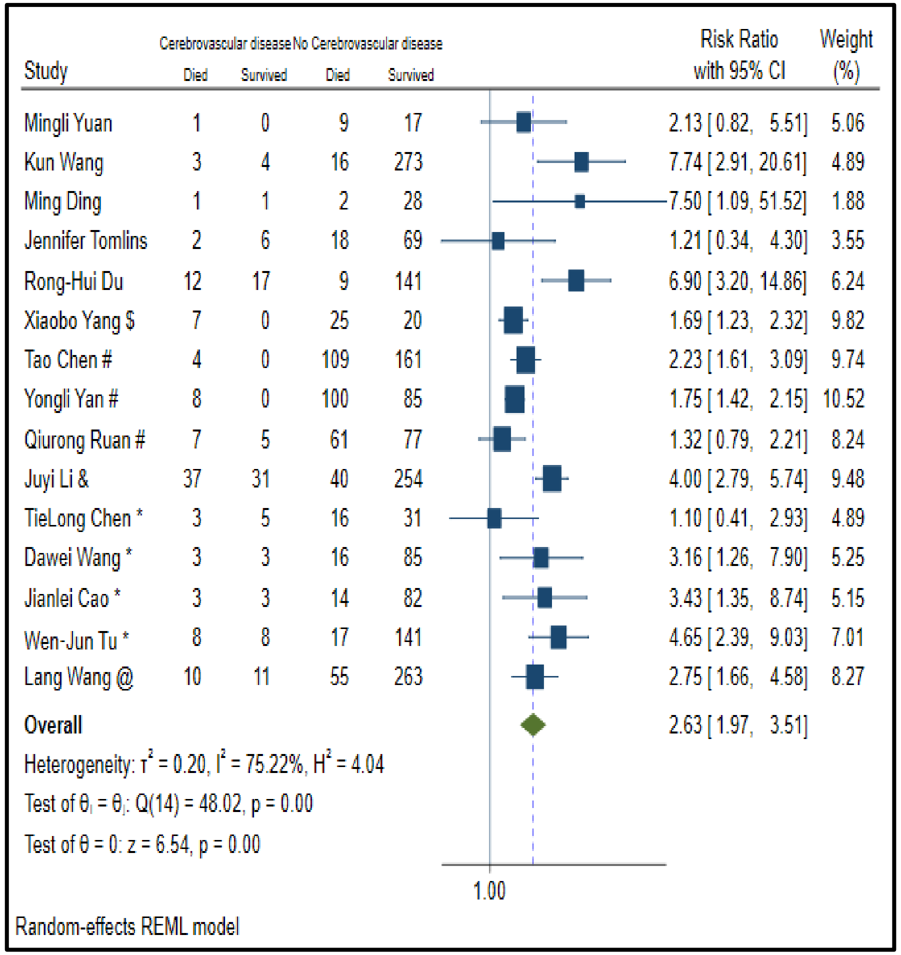

7b

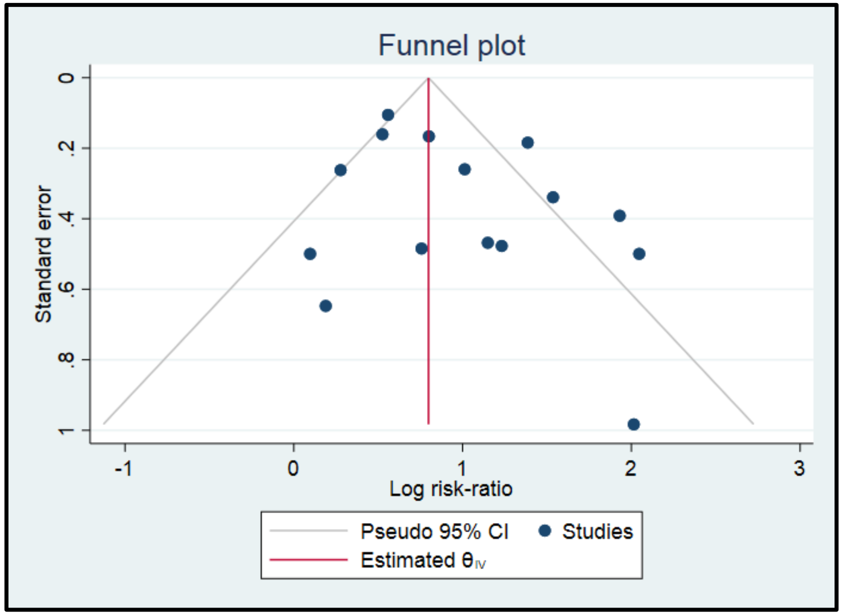

7c

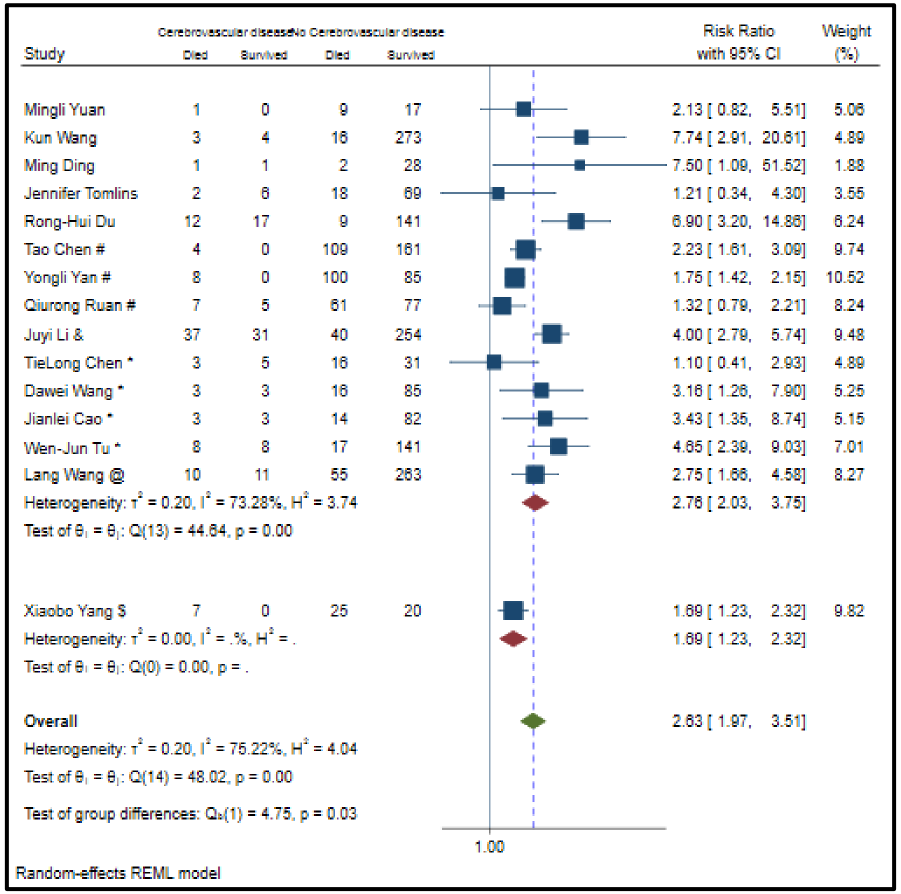
Study
Cerebrovascular disease
No Cerebrovascular disease
Died
Survived
Died
Survived
Risk Ratio with 95% CI
Weight (%)
Mingli Yuan 1 0 9 17 2.13 [0.82, 5.51] 5.06
Kun Wang 3 4 16 273 7.74 [2.91, 20.61] 4.89
Ming Ding 1 1 2 28 7.50 [1.09, 51.52] 1.88
Jennifer Tomlins 2 6 18 69 1.21 [0.34, 4.30] 3.55
Rong-Hui Du 12 17 9 141 6.90 [3.20, 14.86] 6.24
Tao Chen # 4 0 109 161 2.23 [1.61, 3.09] 9.74
Yongli Yan # 8 0 100 85 1.75 [1.42, 2.15] 10.52
Qiurong Ruan # 7 5 61 77 1.32 [0.79, 2.21] 8.24
Juyi Li & 37 31 40 254 4.00 [2.79, 5.74] 9.48
TieLong Chen * 3 5 16 31 1.10 [0.41, 2.93] 4.89
Dawei Wang * 3 3 16 85 3.16 [1.26, 7.90] 5.25
Jianlei Cao * 3 3 14 82 3.43 [1.35, 8.74] 5.15
Wen-Jun Tu * 8 8 17 141 4.65 [2.39, 9.03] 7.01
Lang Wang @ 10 11 55 263 2.75 [1.66, 4.58] 8.27
Heterogeneity: τ² = 0.20, I² = 73.28%, H² = 3.74 2.76 [2.03, 3.75]
Test of θi = θj: Q(13) = 44.64, p = 0.00
Xiaobo Yang $ 7 0 25 20 1.69 [1.23, 2.32] 9.82
Heterogeneity: τ² = 0.00, I² = .%, H² = . 1.69 [1.23, 2.32]
Test of θi = θj: Q(0) = 0.00, p = .
Overall 2.63 [1.97, 3.51]
Heterogeneity: τ² = 0.20, I² = 75.22%, H² = 4.04
Test of θi = θj: Q(14) = 48.02, p = 0.00
Test of group differences: Qb(1) = 4.75, p = 0.03
1.00
Random-effects REML model

eFigure 8. Pooled Risk ratio of death in patients with COPD a) Forest plot b) Funnel Plot

8a

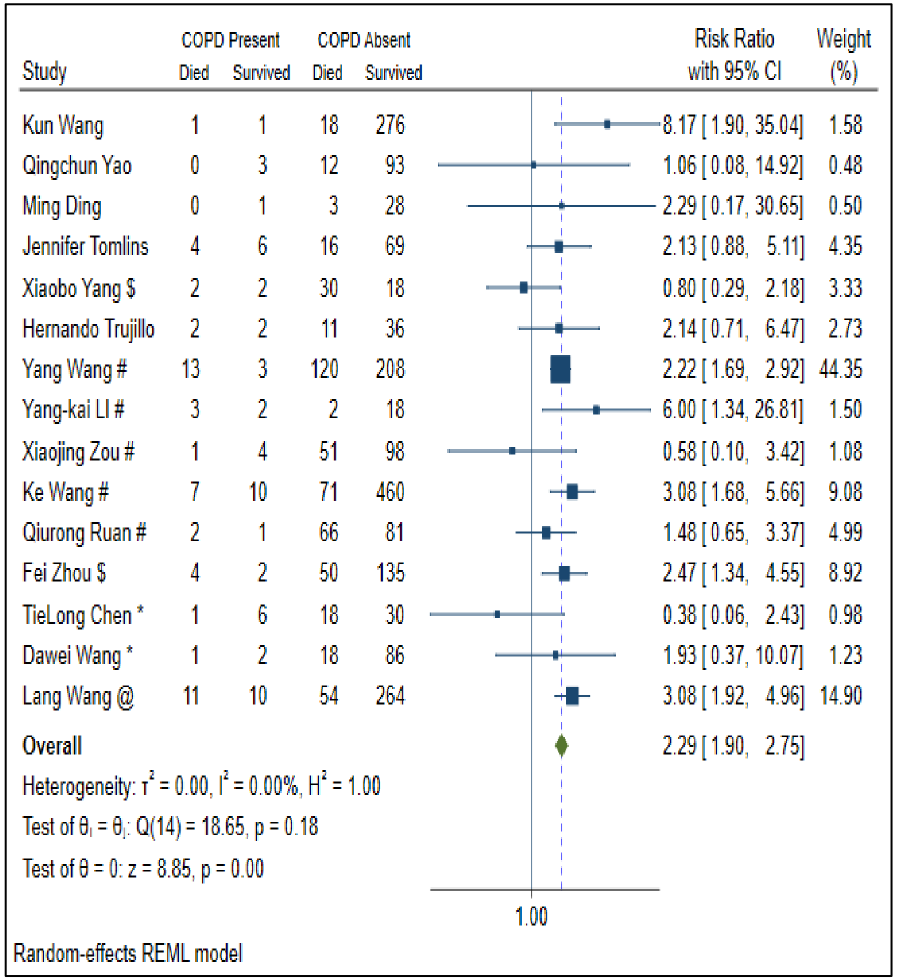

8b

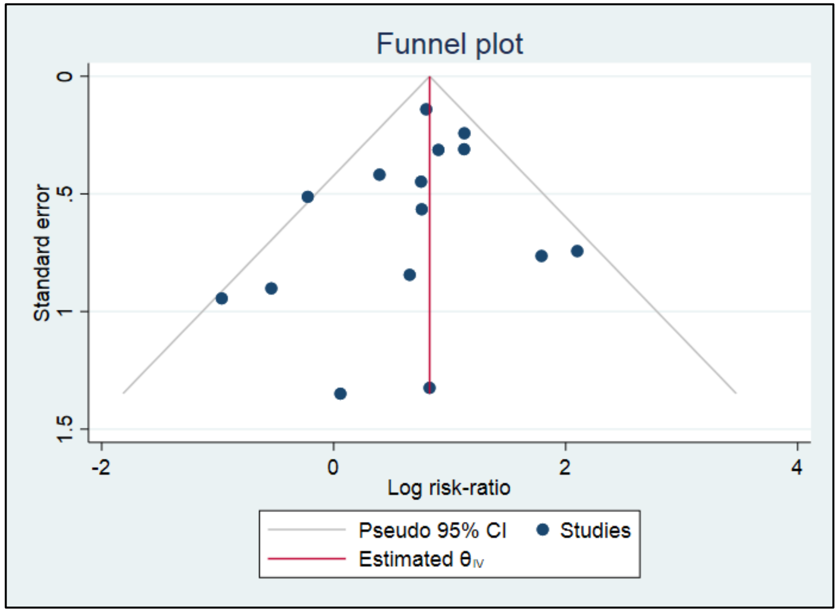

eFigure 9. Pooled Risk ratio of death in patients with Asthma a) Forest plot

9a

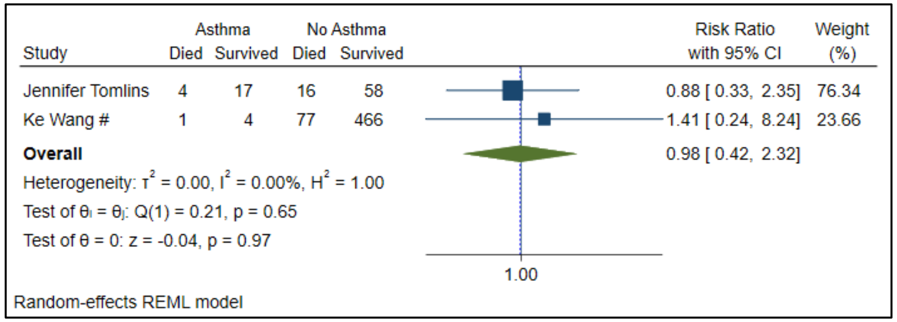

eFigure 10. Pooled Risk ratio of death in patients with Chronic Kidney Disease a) Forest plot b) Funnel Plot

10a

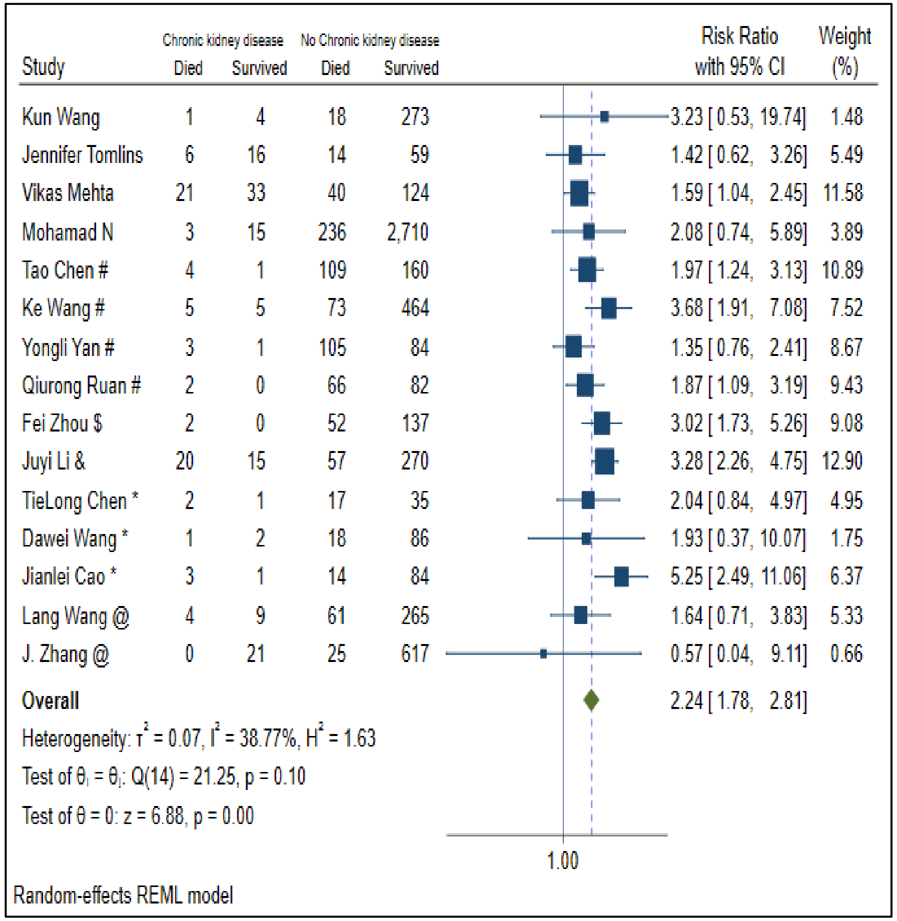

10b

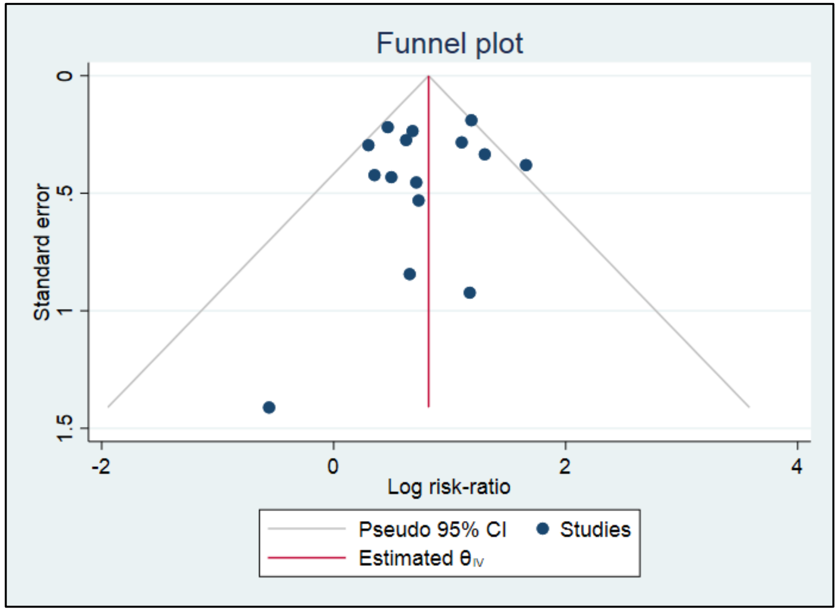

eFigure 11. Pooled Risk ratio of death in patients with Hepatitis B infection a) Forest plot

11a

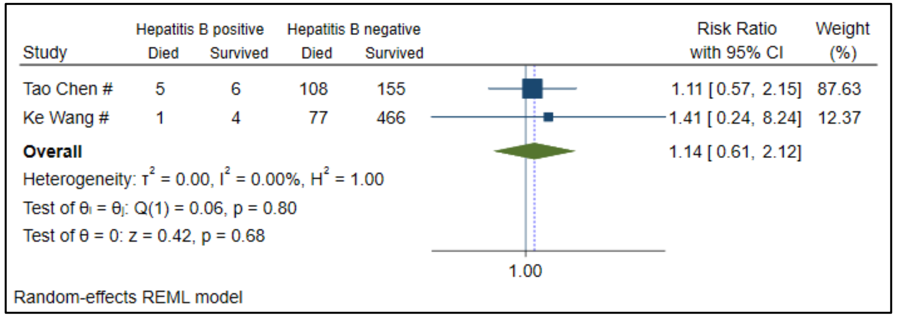

eFigure 12. Pooled Risk ratio of death in patients with HIV infection a) Forest plot

12a

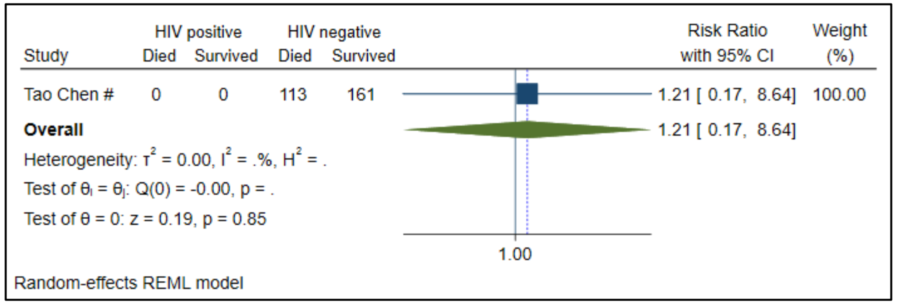

eFigure 13. Pooled Risk ratio of death in patients with Malignancy a) Forest plot b) Funnel Plot

13a

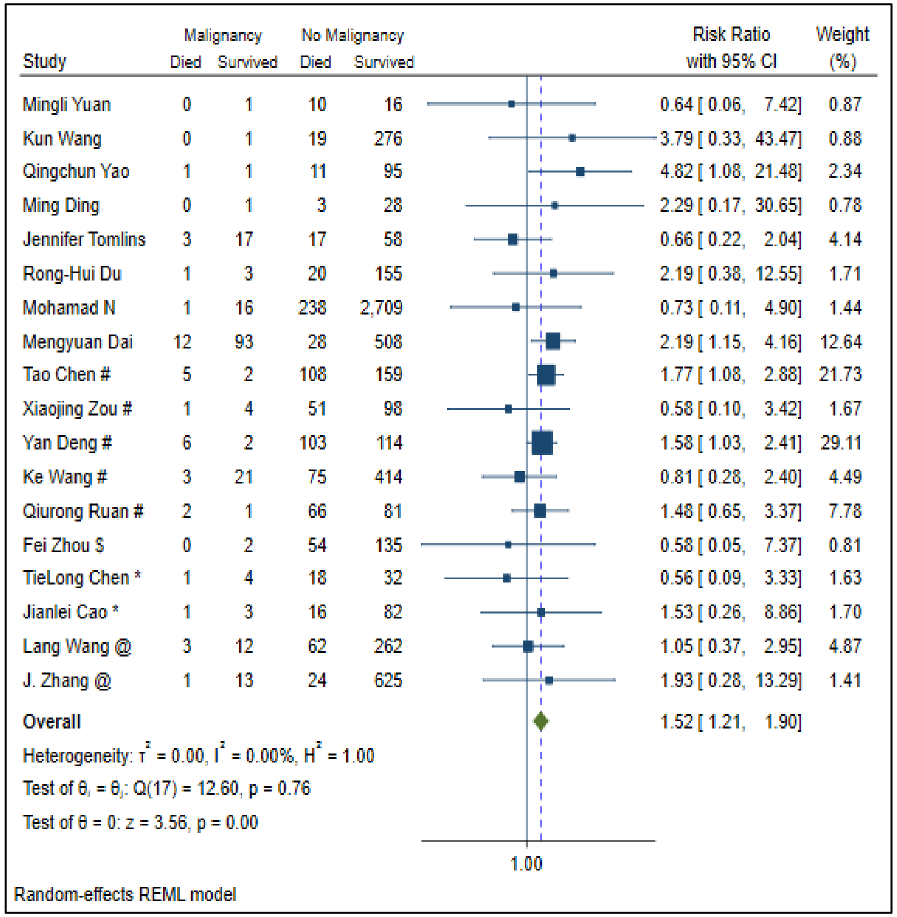

13b

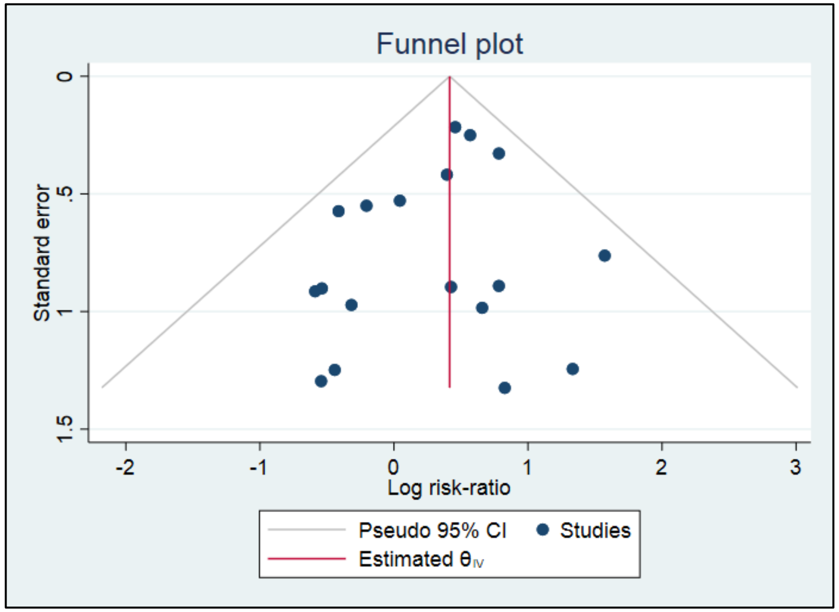

eFigure 14. Pooled Risk ratio of death in patients with fever a) Forest plot b) Funnel Plot

14a

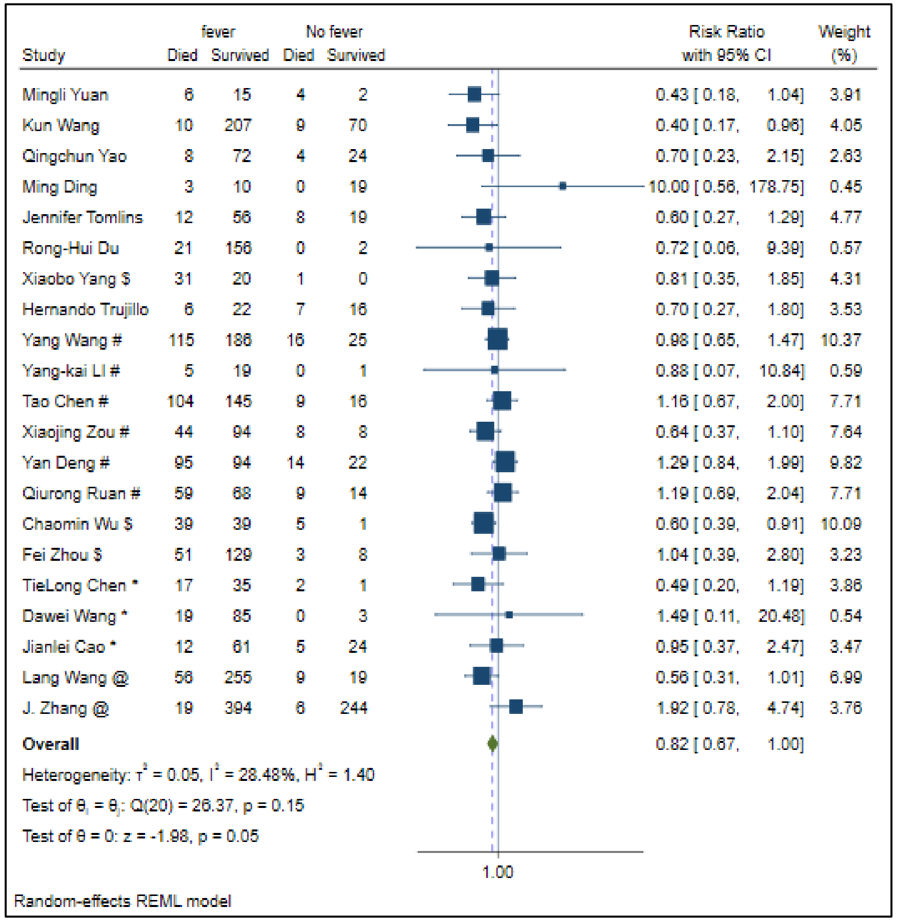

14b

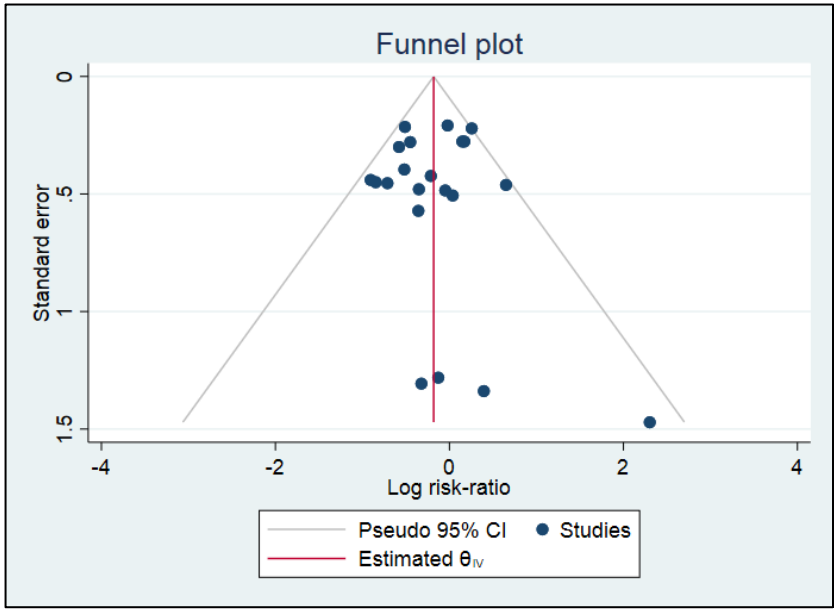

eFigure 15. Pooled Risk ratio of death in patients with sore throat a) Forest plot

15a

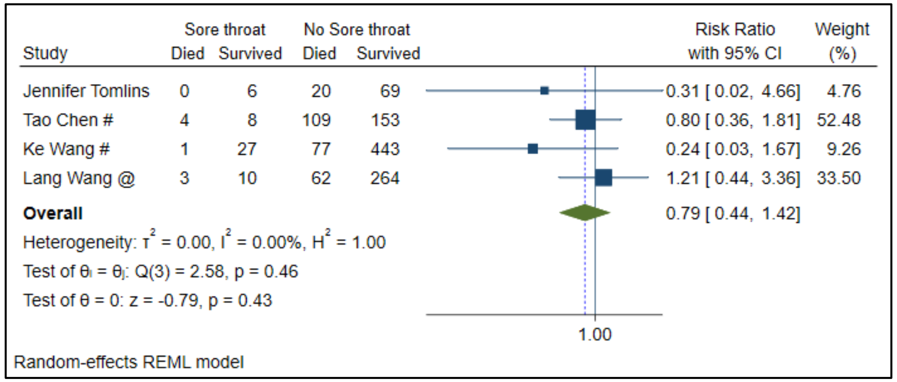

eFigure 16. Pooled Risk ratio of death in patients with cough a) Forest plot b) Funnel Plot

16a

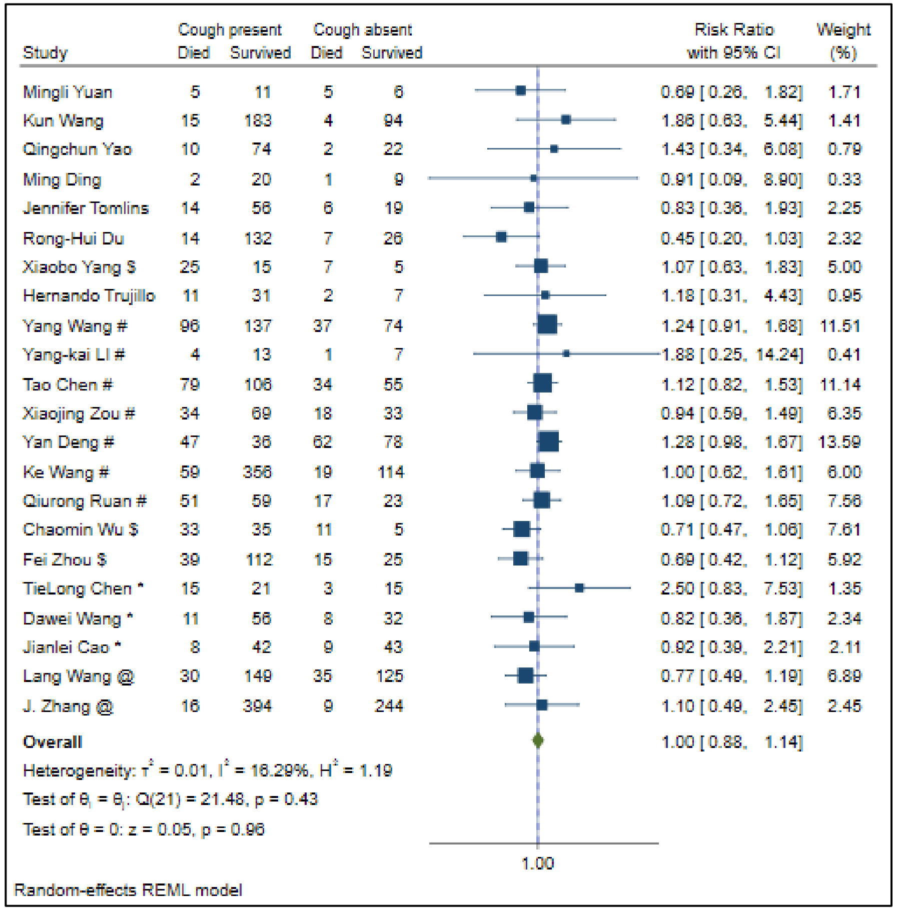

16b

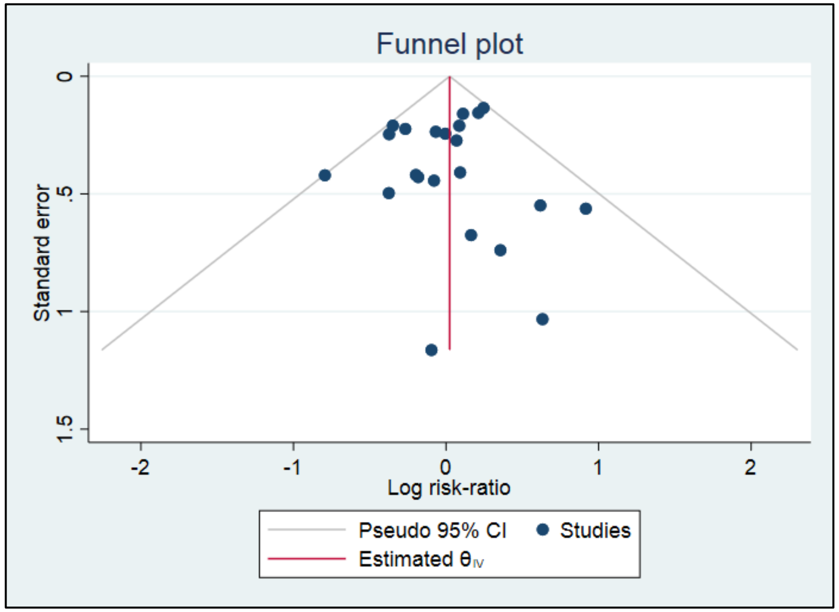

eFigure 17. Pooled Risk ratio of death in patients with expectoration a) Forest plot

17a

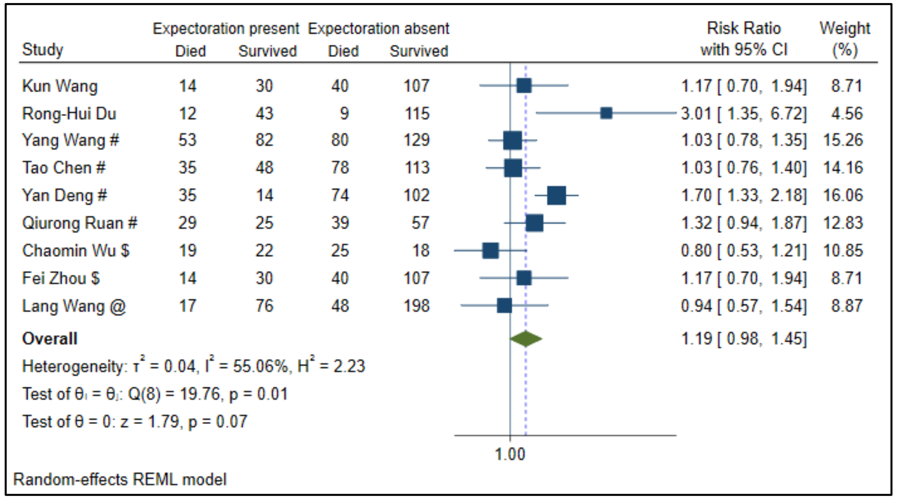

eFigure 18. Pooled Risk ratio of death in patients with vomiting a) Forest plot

18a

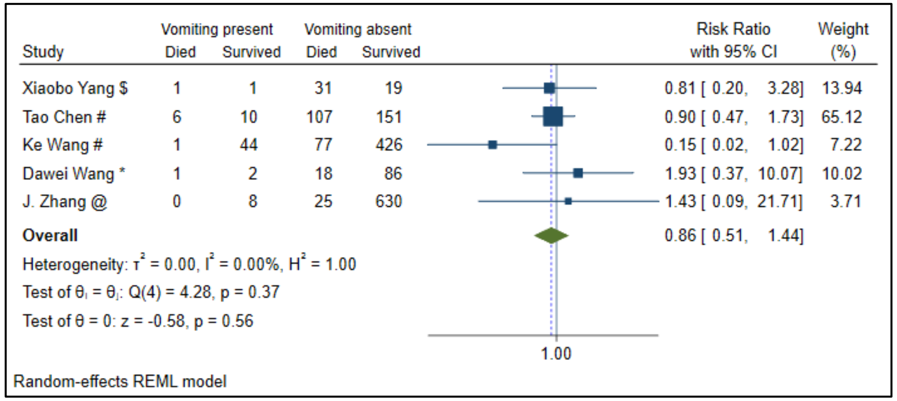

eFigure 19. Pooled Risk ratio of death in patients with Diarrhea a) Forest plot b) Funnel Plot c) Subgroup analysis

19a

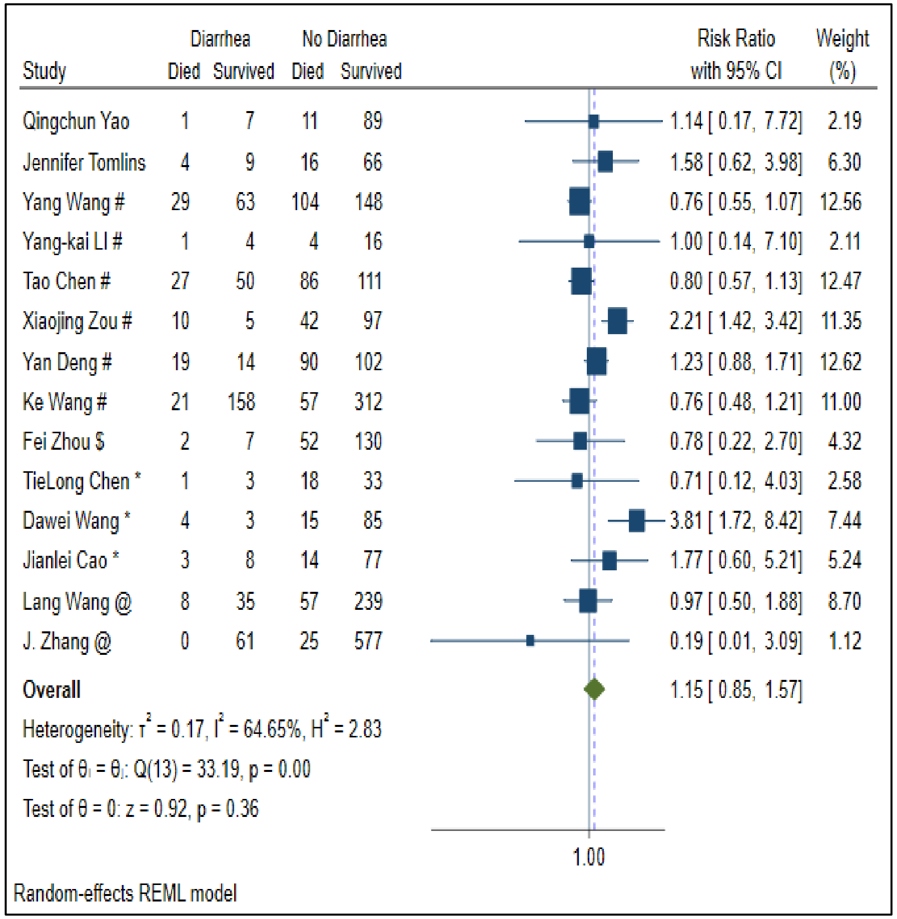

19b

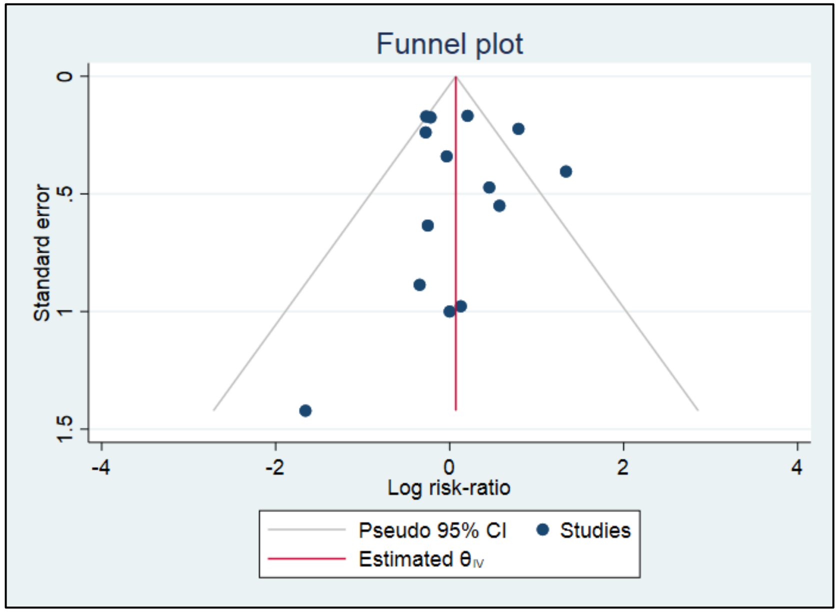

19c

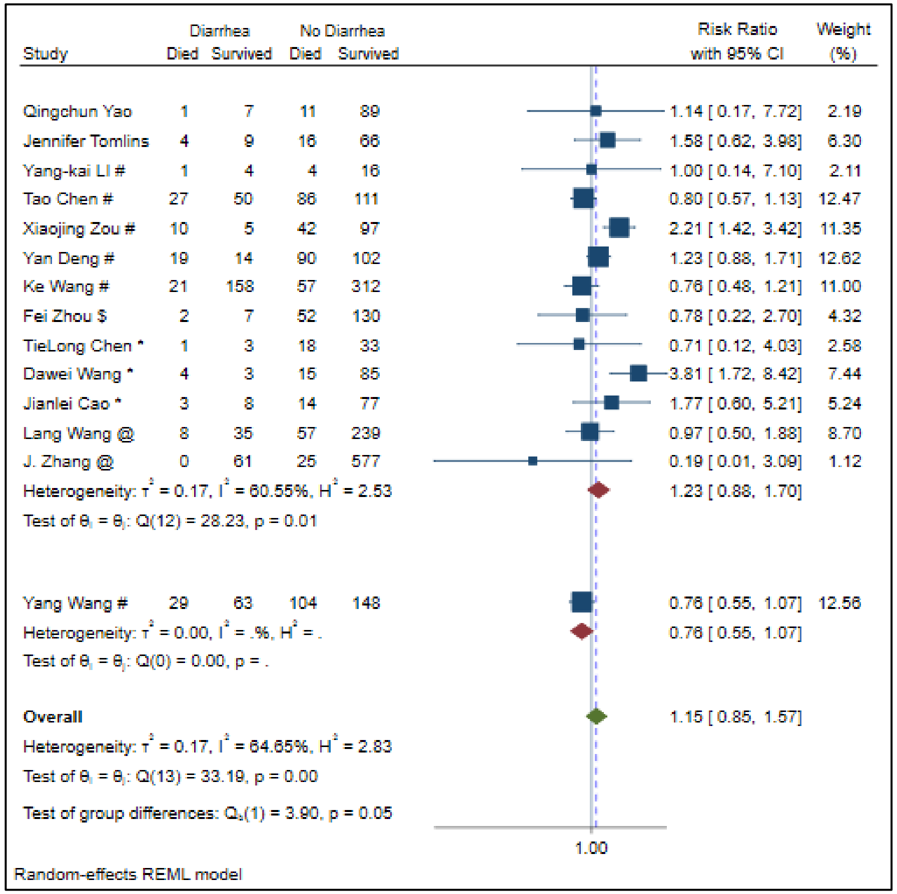
Study
Diarrhea
Died Survived
No Diarrhea
Died Survived
Risk Ratio with 95% CI
Weight (%)
Qingchun Yao 1 7 11 89 1.14 [0.17, 7.72] 2.19
Jennifer Tomlins 4 9 16 66 1.58 [0.62, 3.98] 6.30
Yang-kai LI # 1 4 4 16 1.00 [0.14, 7.10] 2.11
Tao Chen # 27 50 86 111 0.80 [0.57, 1.13] 12.47
Xiaojing Zou # 10 5 42 97 2.21 [1.42, 3.42] 11.35
Yan Deng # 19 14 90 102 1.23 [0.88, 1.71] 12.62
Ke Wang # 21 158 57 312 0.76 [0.48, 1.21] 11.00
Fei Zhou $ 2 7 52 130 0.78 [0.22, 2.70] 4.32
TieLong Chen * 1 3 18 33 0.71 [0.12, 4.03] 2.58
Dawei Wang * 4 3 15 85 3.81 [1.72, 8.42] 7.44
Jianlei Cao * 3 8 14 77 1.77 [0.60, 5.21] 5.24
Lang Wang @ 8 35 57 239 0.97 [0.50, 1.88] 8.70
J. Zhang @ 0 61 25 577 0.19 [0.01, 3.09] 1.12
Heterogeneity: τ² = 0.17, I² = 60.55%, H² = 2.53 1.23 [0.88, 1.70]
Test of θi = θj: Q(12) = 28.23, p = 0.01
Yang Wang # 29 63 104 148 0.76 [0.55, 1.07] 12.56
Heterogeneity: τ² = 0.00, I² = .%, H² = . 0.76 [0.55, 1.07]
Test of θi = θj: Q(0) = 0.00, p = .
Overall 1.15 [0.85, 1.57]
Heterogeneity: τ² = 0.17, I² = 64.65%, H² = 2.83
Test of θi = θj: Q(13) = 33.19, p = 0.00
Test of group differences: Qb(1) = 3.90, p = 0.05
1.00
Random-effects REML model

eFigure 20. Pooled Risk ratio of death in patients with nausea a) Forest plot

20a

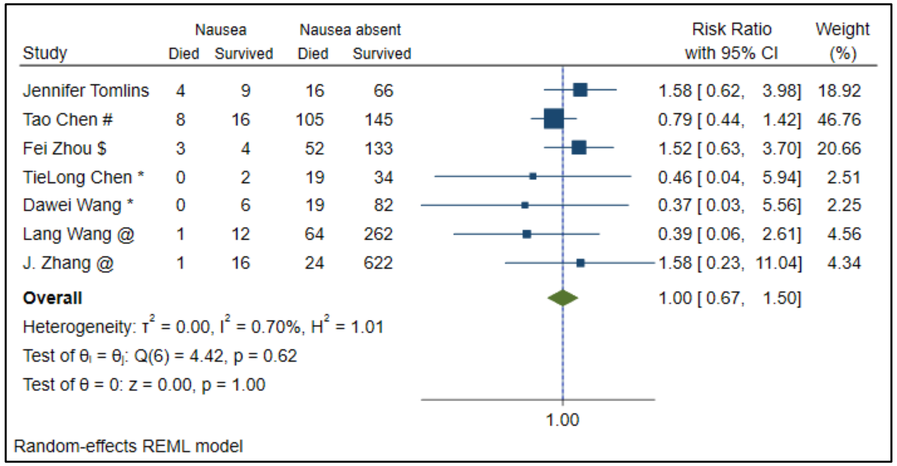

eFigure 21. Pooled Risk ratio of death in patients with Myalgia a) Forest plot b) Funnel Plot

21a

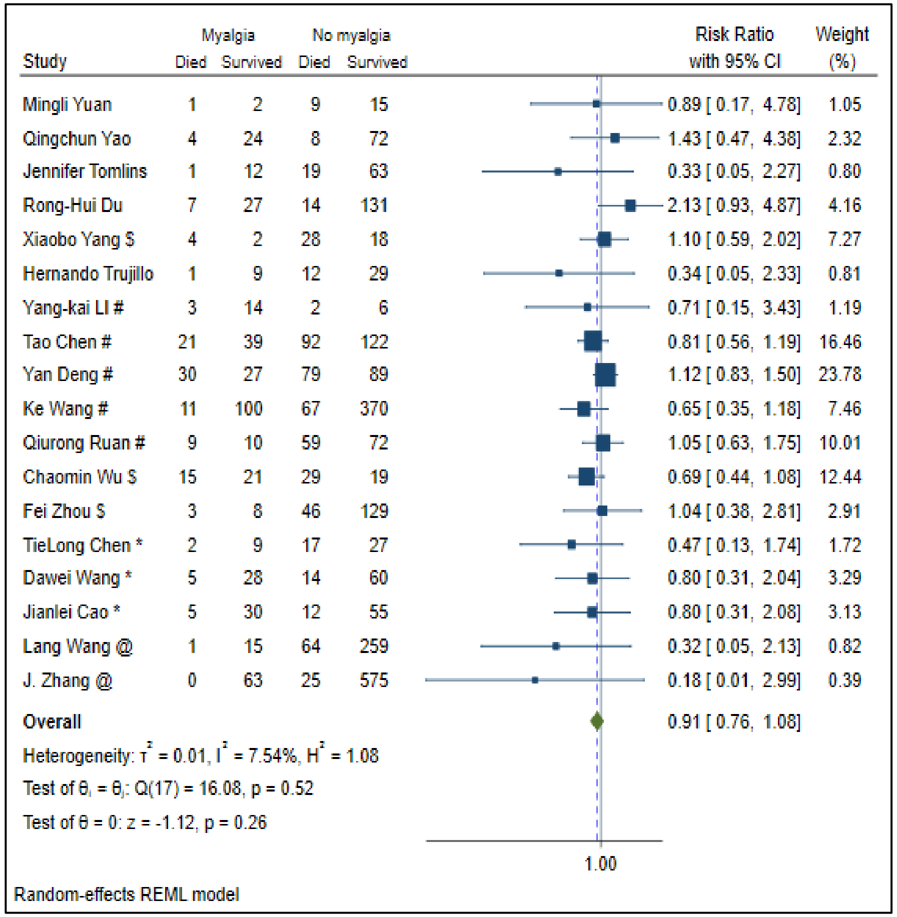

21b

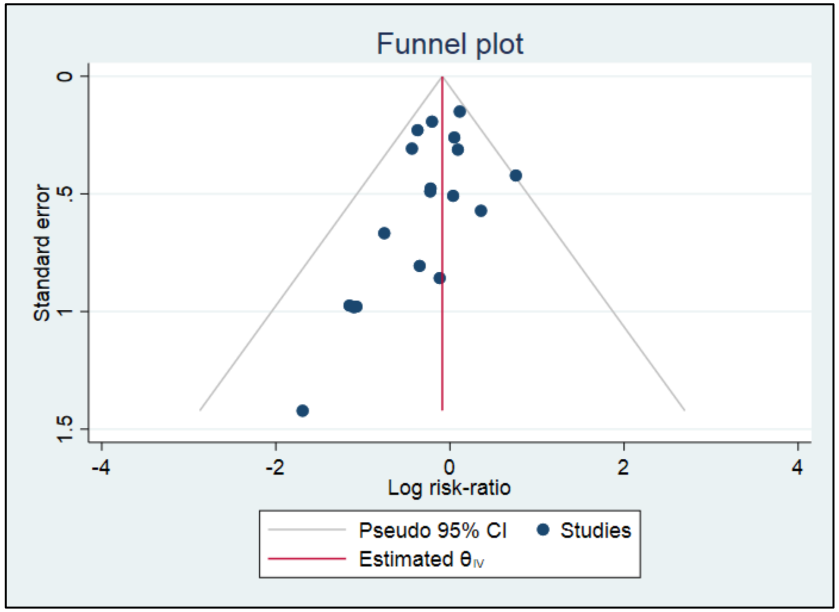

eFigure 22. Pooled Risk ratio of death in patients with headache a) Forest plot b) Funnel Plot

22a

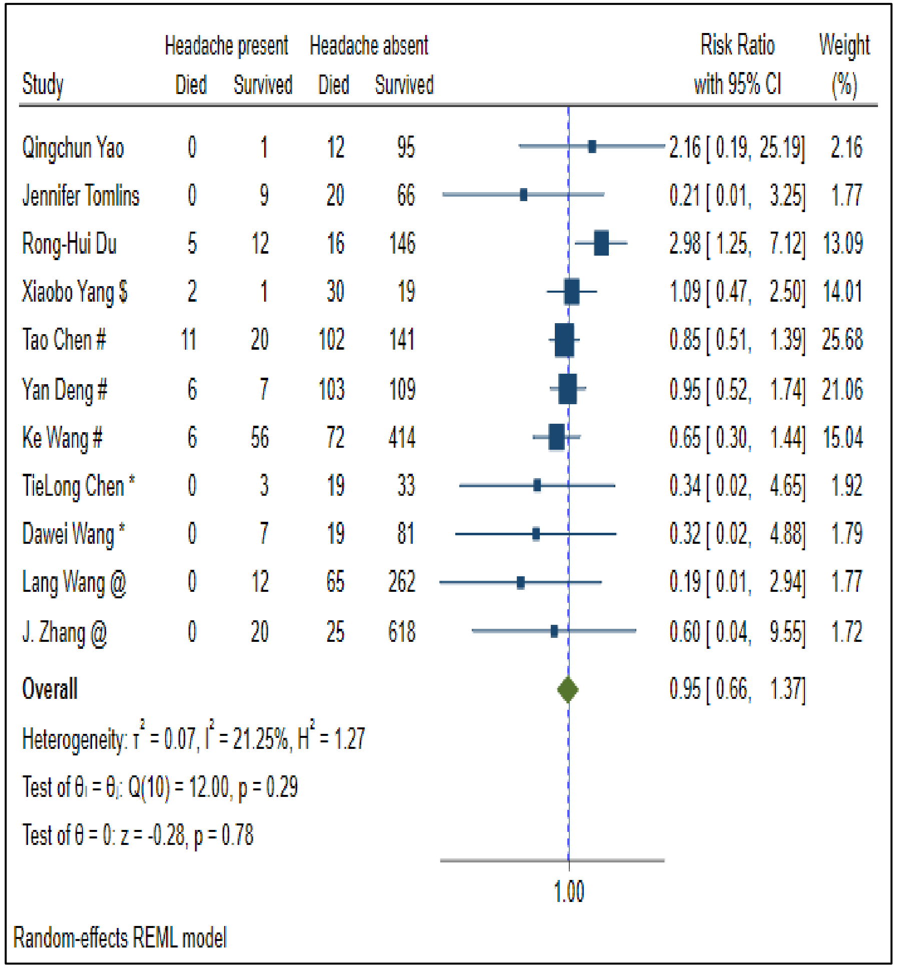

22b

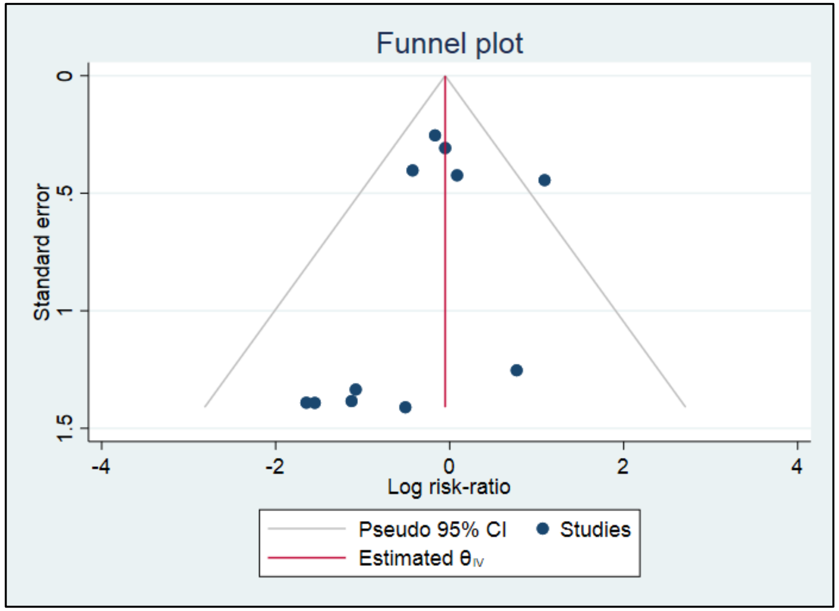

eFigure 23. Pooled Risk ratio of death in patients with anorexia a) Forest plot

23a

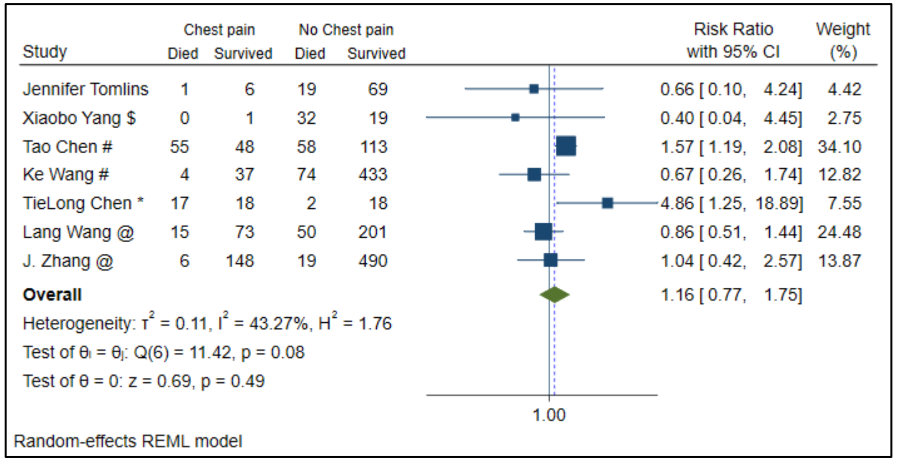

eFigure 24. Pooled Risk ratio of death in patients with chest pain a) Forest plot

24a

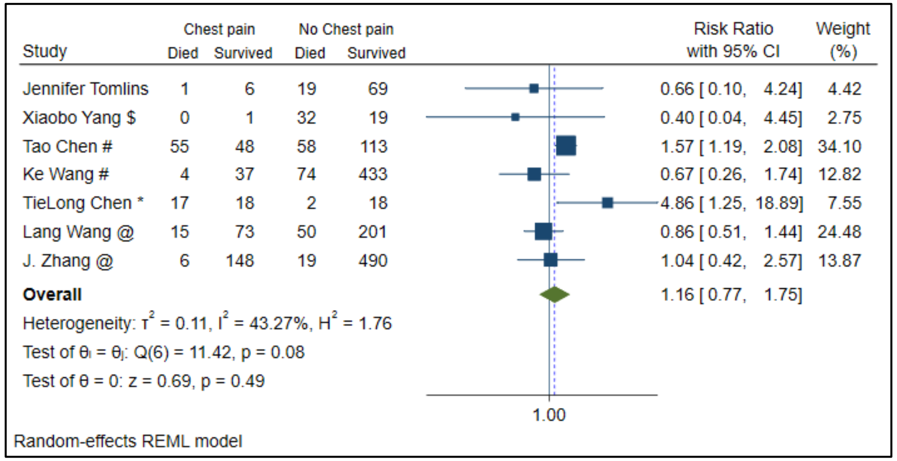

eFigure 25. Pooled Risk ratio of death in patients with dyspnea a) Forest plot b) Funnel Plot c) Subgroup analysis

25a

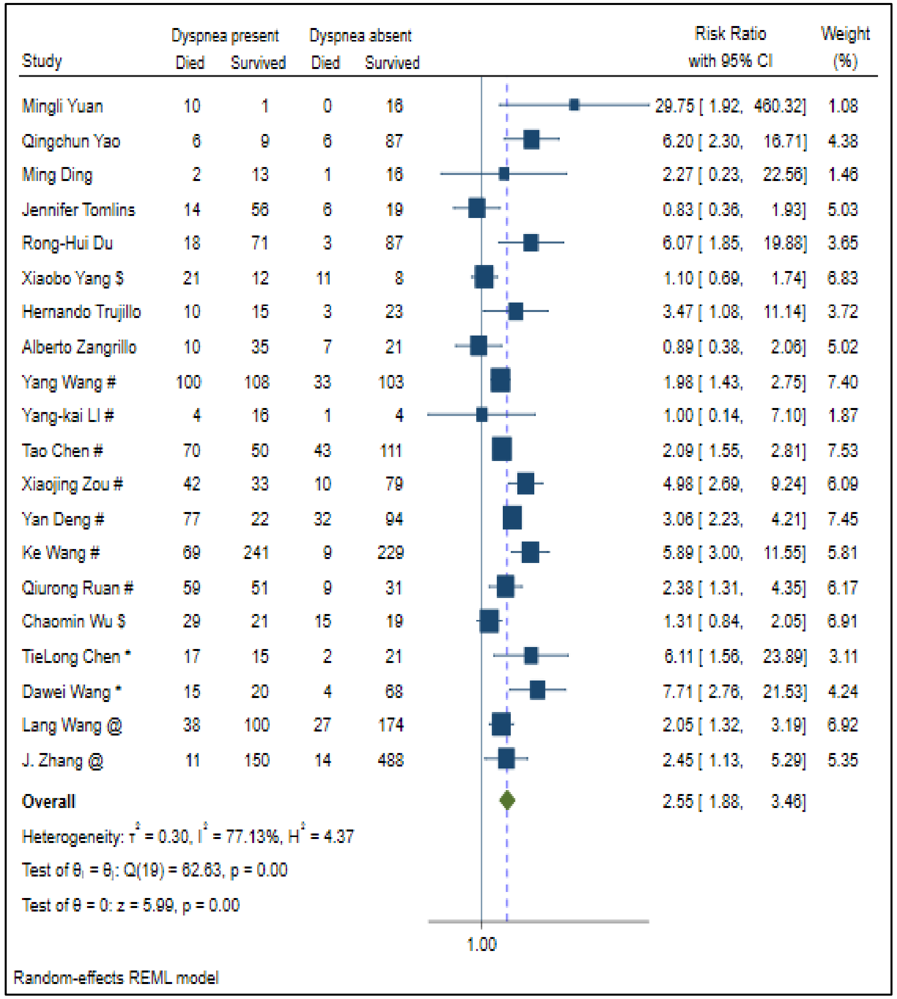

25b

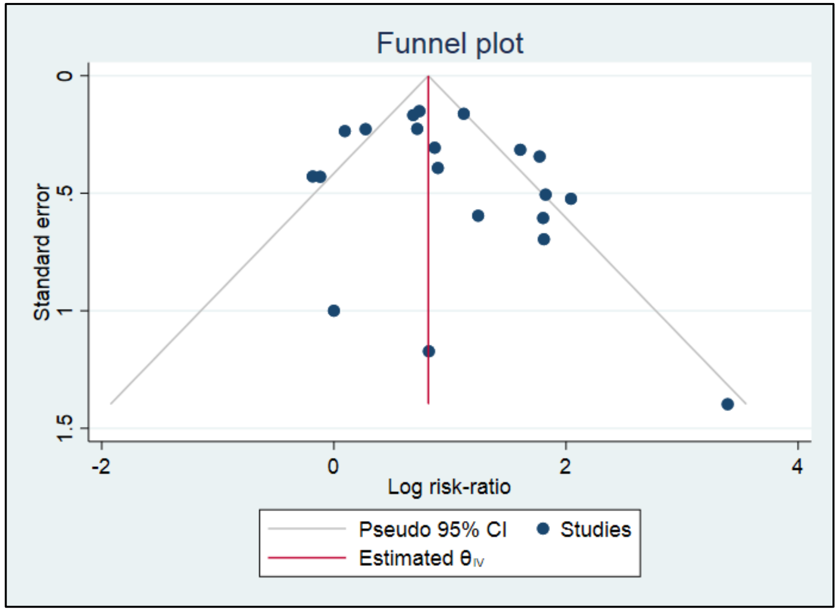

25c

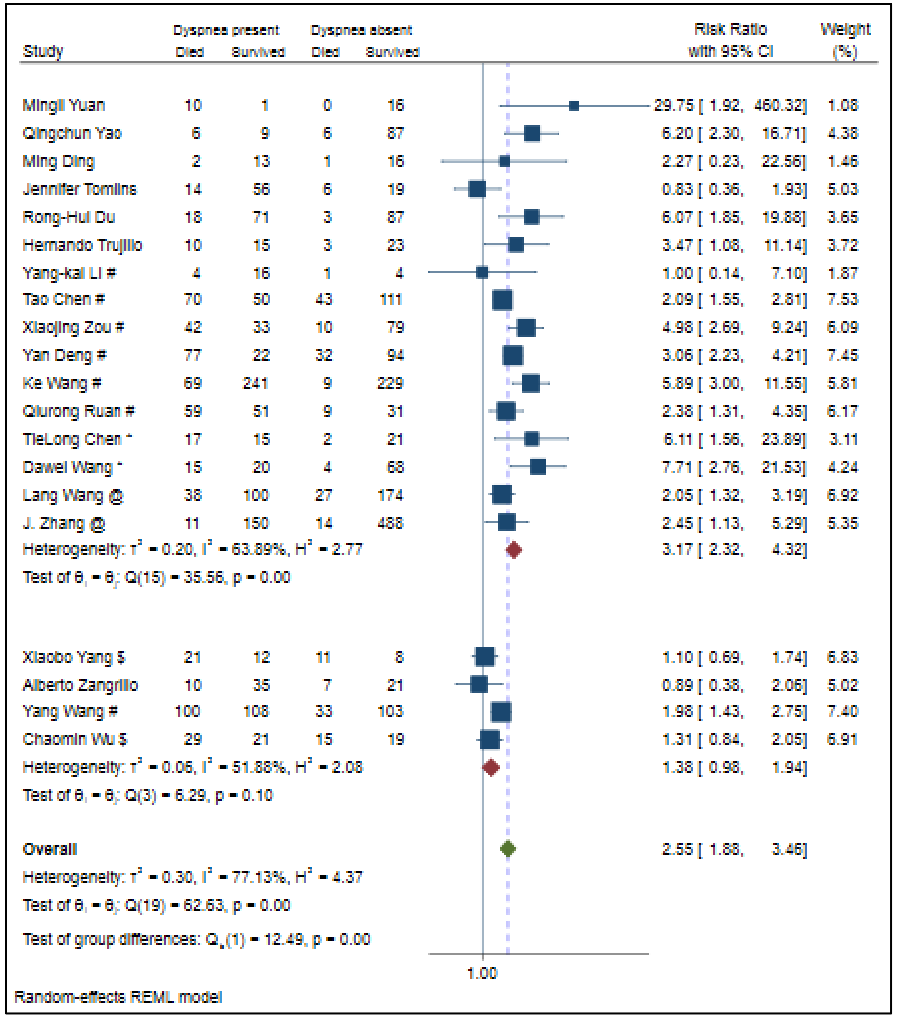

Dyspnea present
Dyspnea absent
Study Died Survived Died Survived
Risk Ratio with 95% CI
Weight (%)
Mingli Yuan 10 1 0 16 29.75 [1.92, 460.32] 1.08
Qingchun Yao 6 9 6 87 6.20 [2.30, 16.71] 4.38
Ming Ding 2 13 1 16 2.27 [0.23, 22.56] 1.46
Jennifer Tomlins 14 56 6 19 0.83 [0.36, 1.93] 5.03
Rong-Hui Du 18 71 3 87 6.07 [1.85, 19.88] 3.65
Hernando Trujillo 10 15 3 23 3.47 [1.08, 11.14] 3.72
Yang-kai Li # 4 16 1 4 1.00 [0.14, 7.10] 1.87
Tao Chen # 70 50 43 111 2.09 [1.55, 2.81] 7.53
Xiaojing Zou # 42 33 10 79 4.98 [2.69, 9.24] 6.09
Yan Deng # 77 22 32 94 3.06 [2.23, 4.21] 7.45
Ke Wang # 69 241 9 229 5.89 [3.00, 11.55] 5.81
Qiurong Ruan # 59 51 9 31 2.38 [1.31, 4.35] 6.17
TieLong Chen ^ 17 15 2 21 6.11 [1.56, 23.89] 3.11
Dawei Wang ^ 15 20 4 68 7.71 [2.76, 21.53] 4.24
Lang Wang @ 38 100 27 174 2.05 [1.32, 3.19] 6.92
J. Zhang @ 11 150 14 488 2.45 [1.13, 5.29] 5.35
Heterogeneity: τ² = 0.20, I² = 63.89%, H² = 2.77 3.17 [2.32, 4.32]
Test of θᵢ = θⱼ: Q(15) = 35.56, p = 0.00
Xiaobo Yang $ 21 12 11 8 1.10 [0.69, 1.74] 6.83
Alberto Zangrillo 10 35 7 21 0.89 [0.38, 2.06] 5.02
Yang Wang # 100 108 33 103 1.98 [1.43, 2.75] 7.40
Chaomin Wu $ 29 21 15 19 1.31 [0.84, 2.05] 6.91
Heterogeneity: τ² = 0.06, I² = 51.88%, H² = 2.08 1.38 [0.98, 1.94]
Test of θᵢ = θⱼ: Q(3) = 6.29, p = 0.10
Overall 2.55 [1.88, 3.46]
Heterogeneity: τ² = 0.30, I² = 77.13%, H² = 4.37
Test of θᵢ = θⱼ: Q(19) = 62.63, p = 0.00
Test of group differences: Qb(1) = 12.49, p = 0.00
1.00
Random-effects REML model

eFigure 26. Pooled Risk ratio of death in patients with hemoptysis a) Forest plot

26a

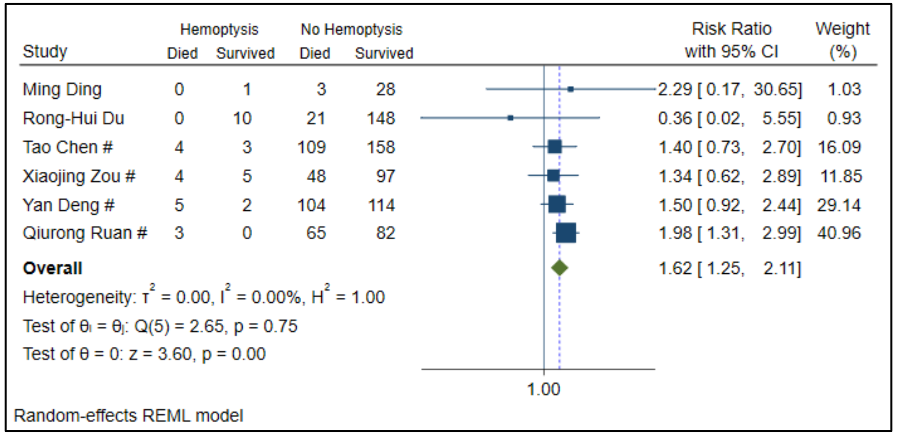

eFigure 27. Pooled Risk ratio of death in patients with Abdomen pain a) Forest plot

27a

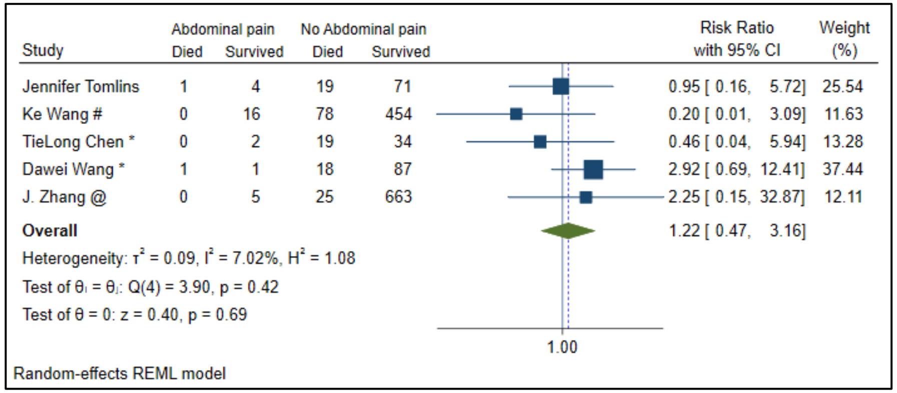

eFigure 28. Pooled Risk ratio of death in patients with Acute respiratory syndrome a) Forest plot b) Funnel Plot c) Subgroup analysis

28a

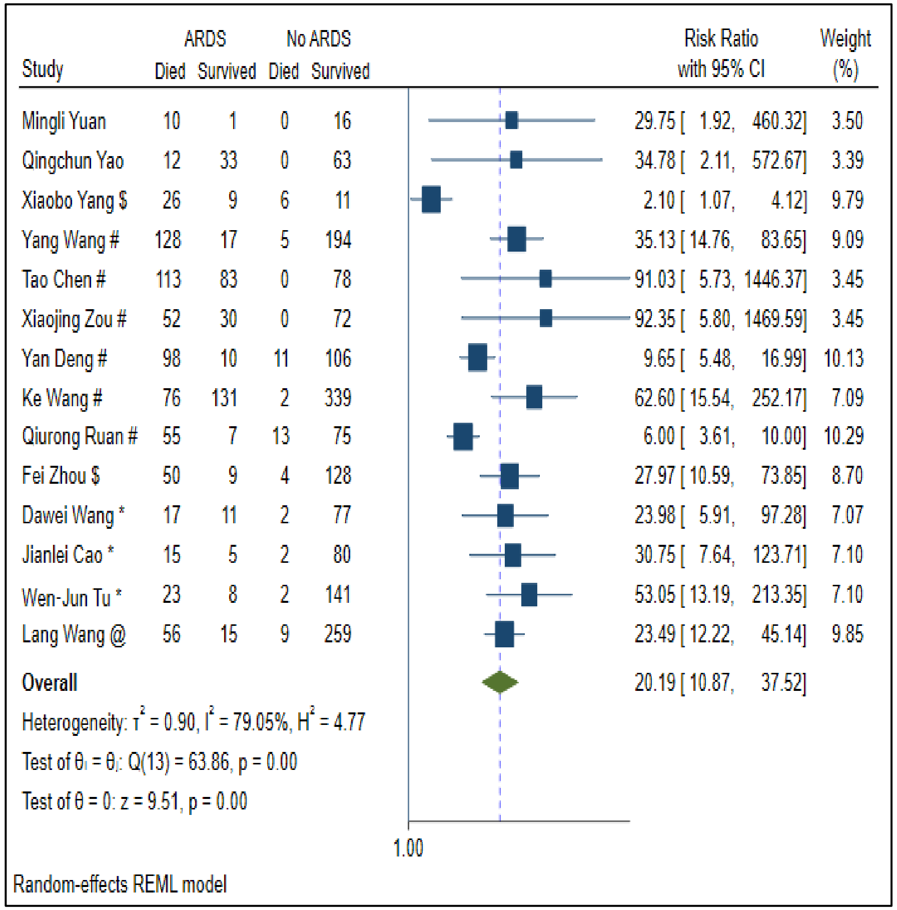

28b

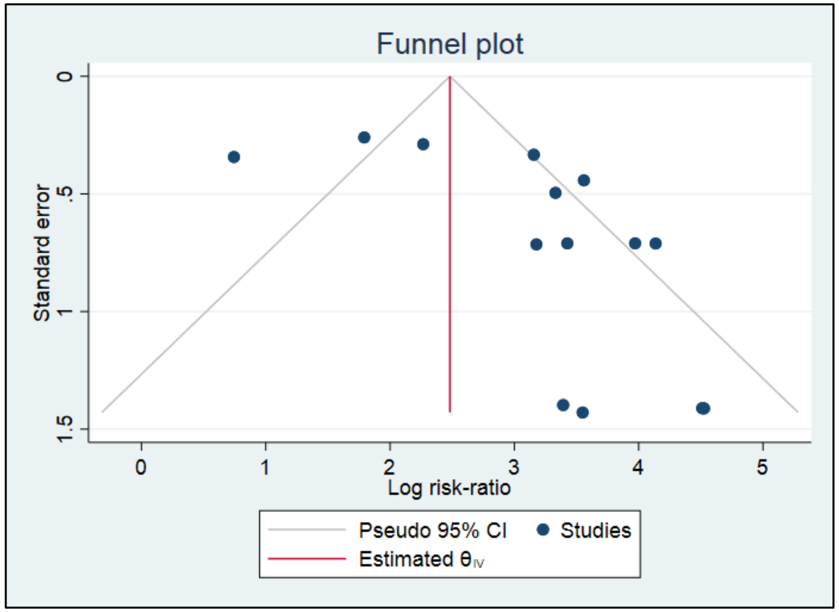

28c

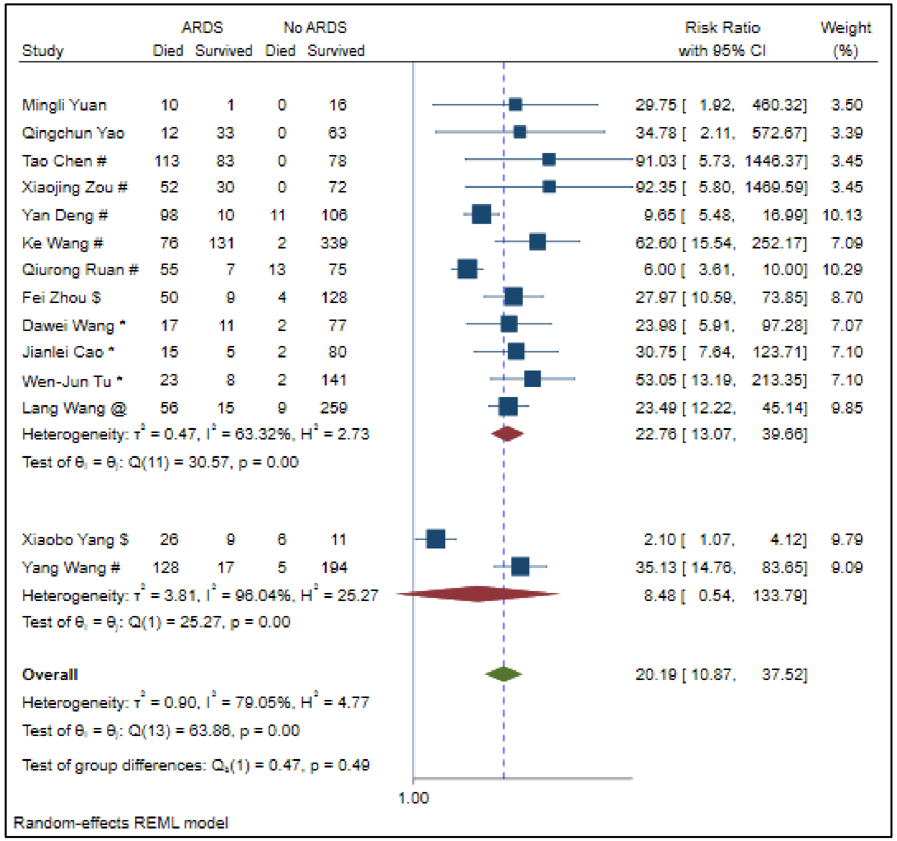
Study
ARDS
Died Survived
No ARDS
Died Survived
Risk Ratio
with 95% CI
Weight
(%)
Mingli Yuan 10 1 0 16 29.75 [1.92, 460.32] 3.50
Qingchun Yao 12 33 0 63 34.78 [2.11, 572.67] 3.39
Tao Chen # 113 83 0 78 91.03 [5.73, 1446.37] 3.45
Xiaojing Zou # 52 30 0 72 92.35 [5.80, 1469.59] 3.45
Yan Deng # 98 10 11 106 9.65 [5.48, 16.99] 10.13
Ke Wang # 76 131 2 339 62.60 [15.54, 252.17] 7.09
Qiurong Ruan # 55 7 13 75 6.00 [3.61, 10.00] 10.29
Fei Zhou $ 50 9 4 128 27.97 [10.59, 73.85] 8.70
Dawei Wang * 17 11 2 77 23.98 [5.91, 97.28] 7.07
Jianlei Cao * 15 5 2 80 30.75 [7.64, 123.71] 7.10
Wen-Jun Tu * 23 8 2 141 53.05 [13.19, 213.35] 7.10
Lang Wang @ 56 15 9 259 23.49 [12.22, 45.14] 9.85
Heterogeneity: τ^2 = 0.47, I^2 = 63.32%, H^2 = 2.73 22.76 [13.07, 39.66]
Test of $\theta_i = \theta_j$: Q(11) = 30.57, p = 0.00
Xiaobo Yang $ 26 9 6 11 2.10 [1.07, 4.12] 9.79
Yang Wang # 128 17 5 194 35.13 [14.76, 83.65] 9.09
Heterogeneity: τ^2 = 3.81, I^2 = 96.04%, H^2 = 25.27 8.48 [0.54, 133.79]
Test of $\theta_i = \theta_j$: Q(1) = 25.27, p = 0.00
Overall 20.19 [10.87, 37.52]
Heterogeneity: τ^2 = 0.90, I^2 = 79.05%, H^2 = 4.77
Test of $\theta_i = \theta_j$: Q(13) = 63.86, p = 0.00
Test of group differences: $Q_b(1)$ = 0.47, p = 0.49
1.00
Random-effects REML model

eFigure 29. Pooled Risk ratio of death in patients with shock a) Forest plot b) Funnel Plot c) Subgroup analysis.

29a

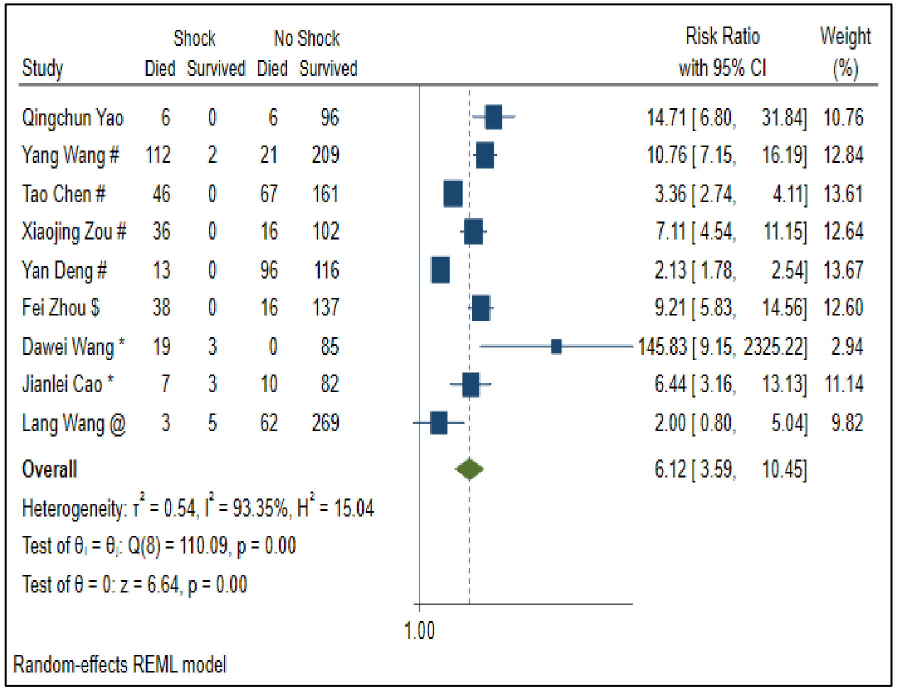

29b

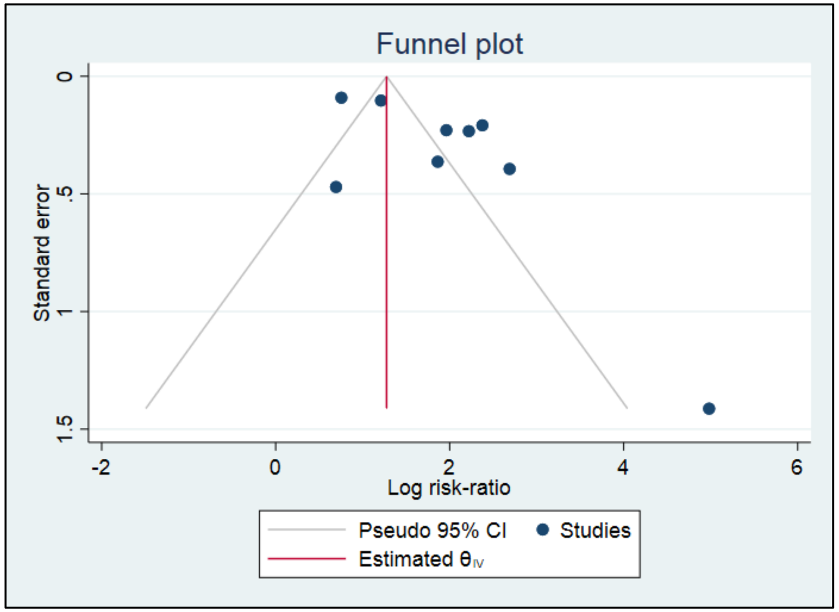

29c

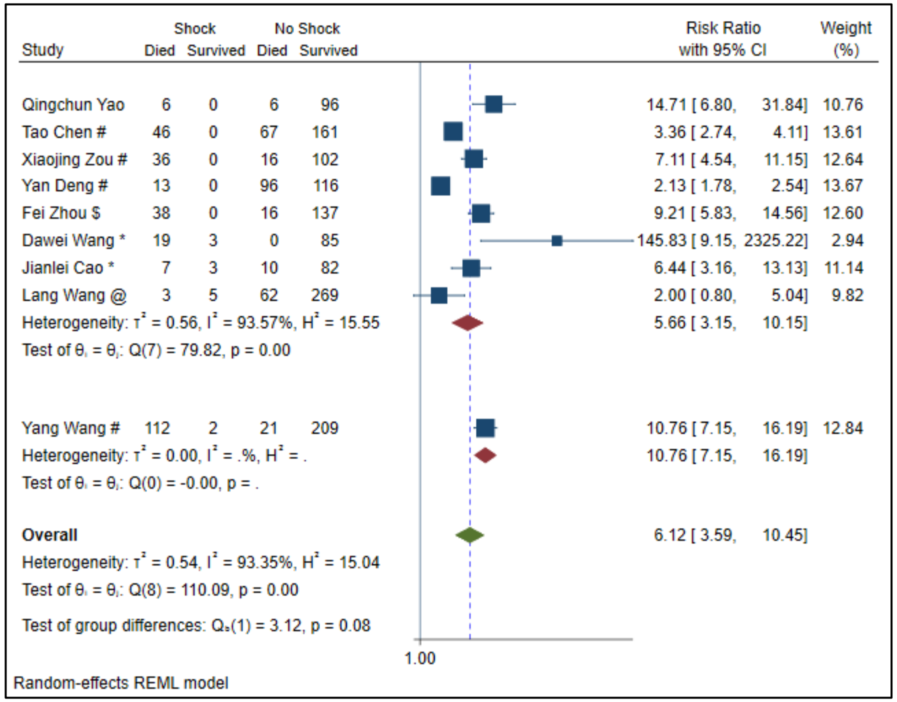
Study
Shock
No Shock
Died Survived Died Survived
Risk Ratio with 95% CI
Weight (%)
Qingchun Yao 6 0 6 96 14.71 [6.80, 31.84] 10.76
Tao Chen # 46 0 67 161 3.36 [2.74, 4.11] 13.61
Xiaojing Zou # 36 0 16 102 7.11 [4.54, 11.15] 12.64
Yan Deng # 13 0 96 116 2.13 [1.78, 2.54] 13.67
Fei Zhou $ 38 0 16 137 9.21 [5.83, 14.56] 12.60
Dawei Wang * 19 3 0 85 145.83 [9.15, 2325.22] 2.94
Jianlei Cao * 7 3 10 82 6.44 [3.16, 13.13] 11.14
Lang Wang @ 3 5 62 269 2.00 [0.80, 5.04] 9.82
Heterogeneity: τ^2 = 0.56, I^2 = 93.57%, H^2 = 15.55 5.66 [3.15, 10.15]
Test of $\theta_i = \theta_j$: Q(7) = 79.82, p = 0.00
Yang Wang # 112 2 21 209 10.76 [7.15, 16.19] 12.84
Heterogeneity: τ^2 = 0.00, I^2 = .%, H^2 = . 10.76 [7.15, 16.19]
Test of $\theta_i = \theta_j$: Q(0) = -0.00, p = .
Overall 6.12 [3.59, 10.45]
Heterogeneity: τ^2 = 0.54, I^2 = 93.35%, H^2 = 15.04
Test of $\theta_i = \theta_j$: Q(8) = 110.09, p = 0.00
Test of group differences: $Q_b(1)$ = 3.12, p = 0.08
1.00
Random-effects REML model

eFigure 30. Pooled Risk ratio of death in patients with sepsis a) Forest plot b) Funnel Plot

30a

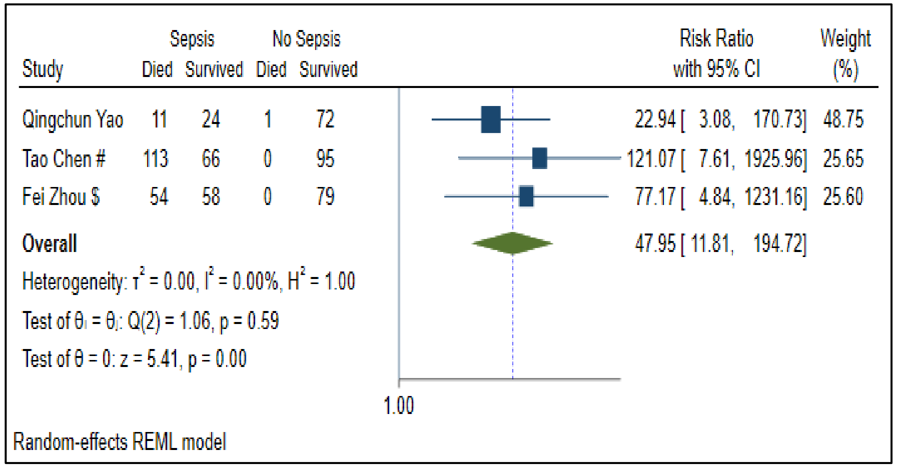

30b

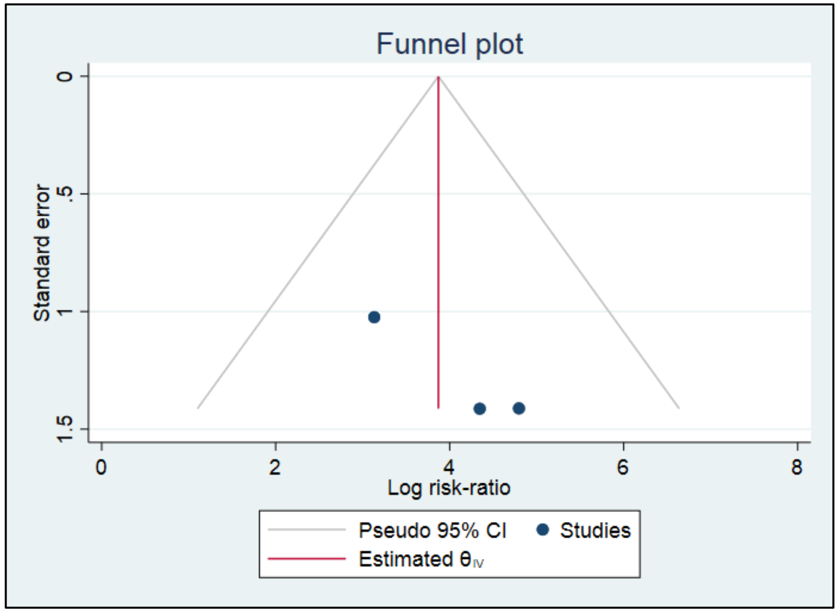

eFigure 31. Pooled Risk ratio of death in patients with bacteremia a) Forest plot b) Funnel Plot c) Subgroup analysis

31a

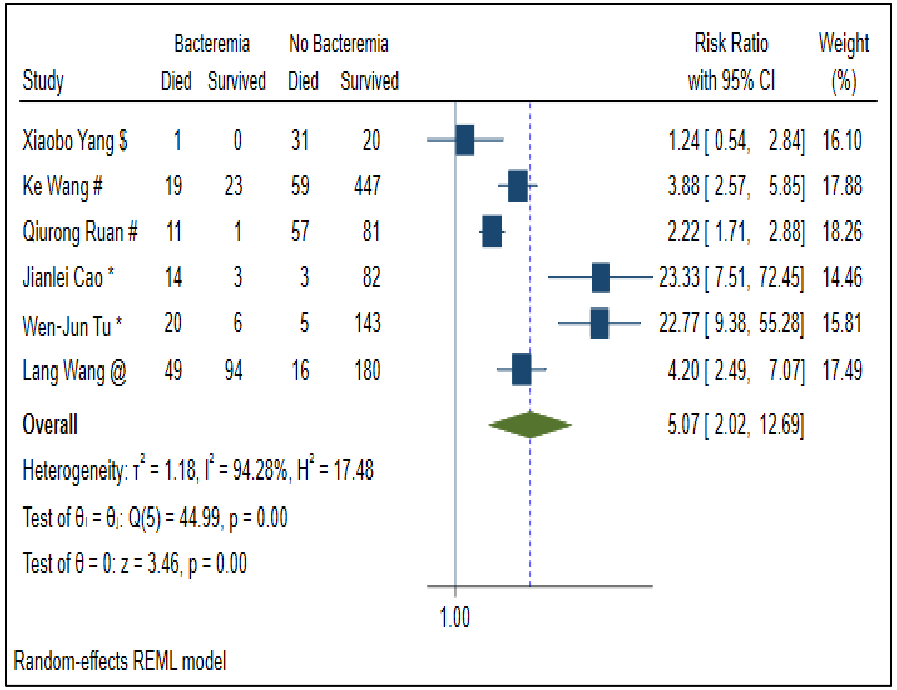

31b

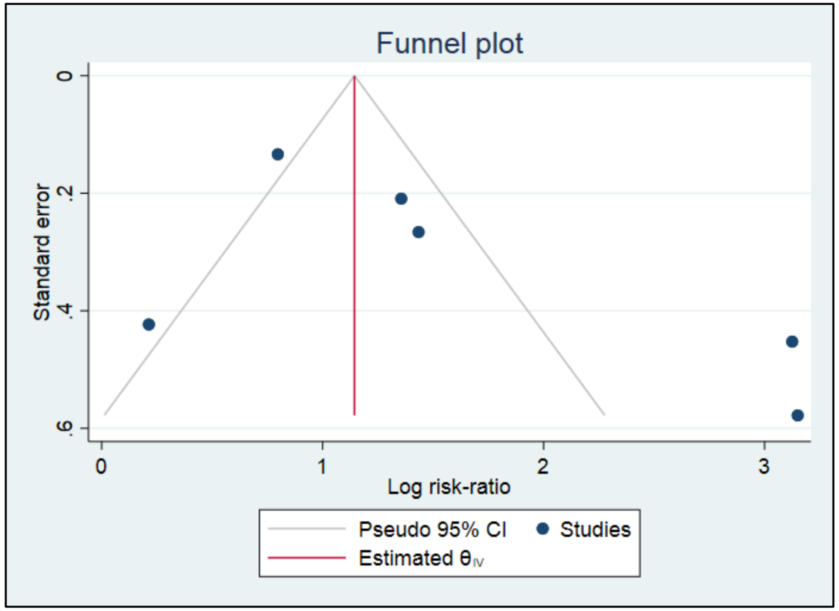

31c

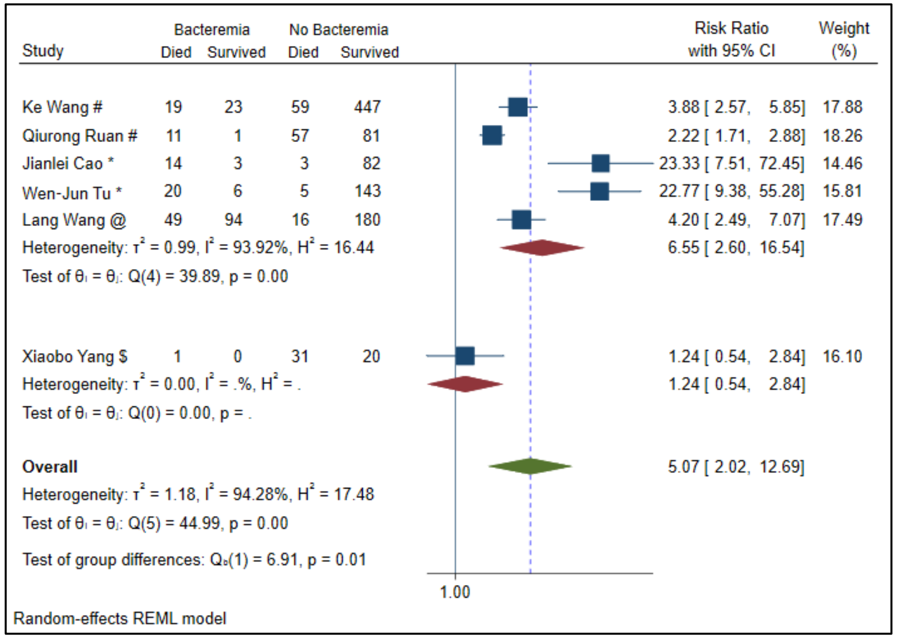

Study
Bacteremia
Died Survived
No Bacteremia
Died Survived
Risk Ratio with 95% CI
Weight (%)
Ke Wang # 19 23 59 447 3.88 [2.57, 5.85] 17.88
Qiurong Ruan # 11 1 57 81 2.22 [1.71, 2.88] 18.26
Jianlei Cao * 14 3 3 82 23.33 [7.51, 72.45] 14.46
Wen-Jun Tu * 20 6 5 143 22.77 [9.38, 55.28] 15.81
Lang Wang @ 49 94 16 180 4.20 [2.49, 7.07] 17.49
Heterogeneity: τ² = 0.99, I² = 93.92%, H² = 16.44 6.55 [2.60, 16.54]
Test of θi = θj: Q(4) = 39.89, p = 0.00
Xiaobo Yang $ 1 0 31 20 1.24 [0.54, 2.84] 16.10
Heterogeneity: τ² = 0.00, I² = .%, H² = . 1.24 [0.54, 2.84]
Test of θi = θj: Q(0) = 0.00, p = .
Overall 5.07 [2.02, 12.69]
Heterogeneity: τ² = 1.18, I² = 94.28%, H² = 17.48
Test of θi = θj: Q(5) = 44.99, p = 0.00
Test of group differences: Qb(1) = 6.91, p = 0.01
1.00
Random-effects REML model

eFigure 32. Pooled Risk ratio of death in patients with Acute cardiac injury a) Forest plot b) Funnel Plot c) Subgroup analysis

32a

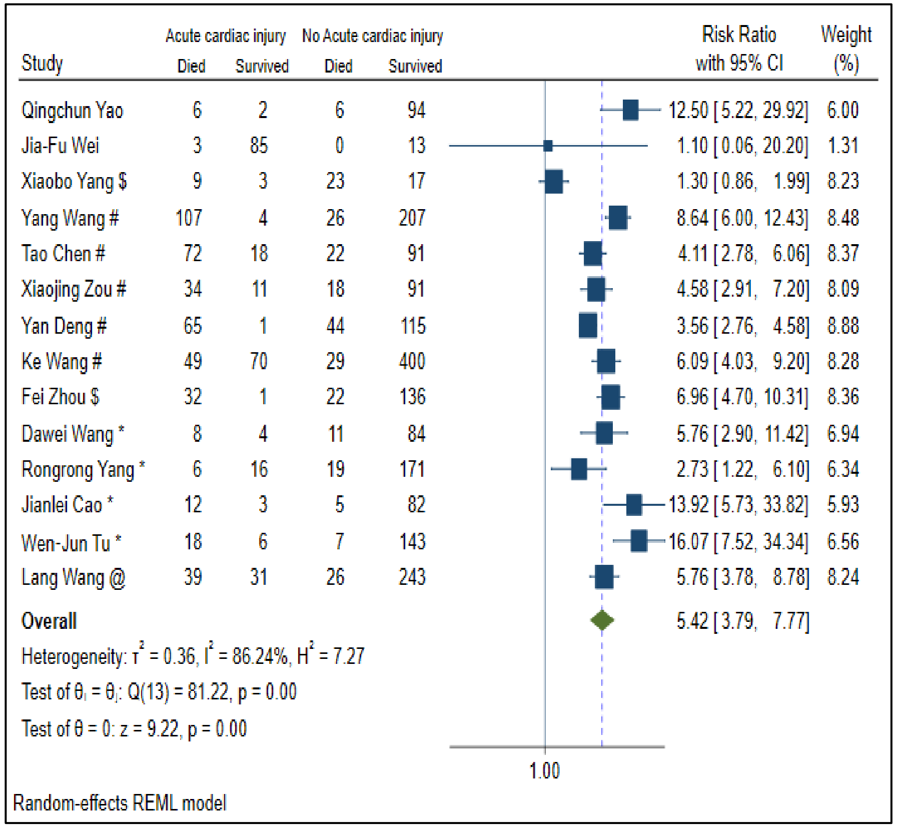

32b

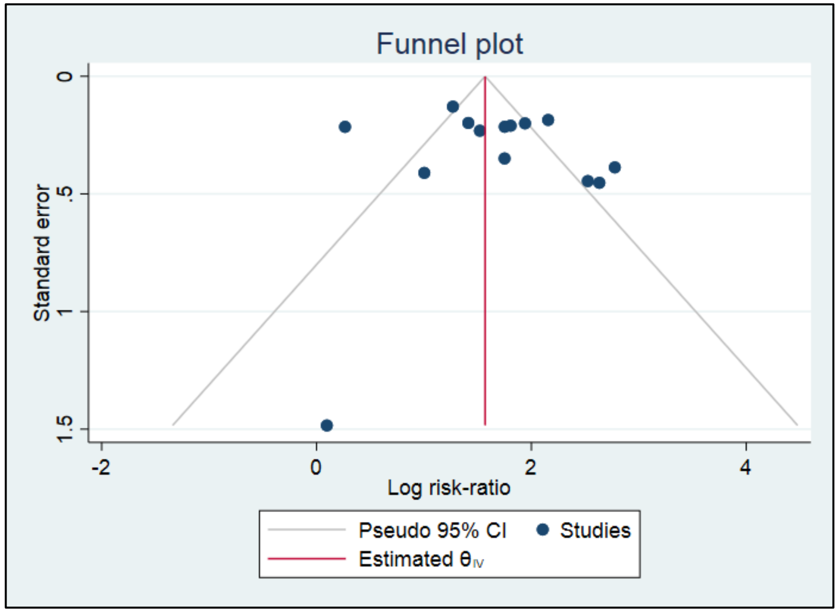

32c

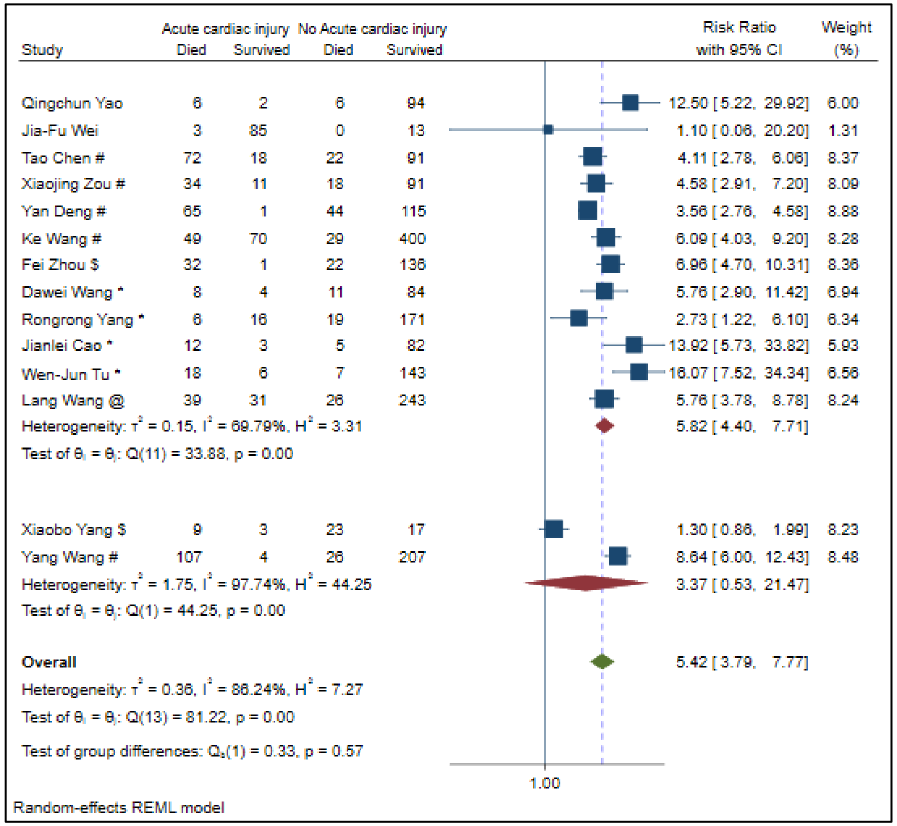
Study
Acute cardiac injury
No Acute cardiac injury
Died
Survived
Died
Survived
Risk Ratio with 95% CI
Weight (%)
Qingchun Yao 6 2 6 94 12.50 [5.22, 29.92] 6.00
Jia-Fu Wei 3 85 0 13 1.10 [0.06, 20.20] 1.31
Tao Chen # 72 18 22 91 4.11 [2.78, 6.06] 8.37
Xiaojing Zou # 34 11 18 91 4.58 [2.91, 7.20] 8.09
Yan Deng # 65 1 44 115 3.56 [2.76, 4.58] 8.88
Ke Wang # 49 70 29 400 6.09 [4.03, 9.20] 8.28
Fei Zhou $ 32 1 22 136 6.96 [4.70, 10.31] 8.36
Dawei Wang * 8 4 11 84 5.76 [2.90, 11.42] 6.94
Rongrong Yang * 6 16 19 171 2.73 [1.22, 6.10] 6.34
Jianlei Cao * 12 3 5 82 13.92 [5.73, 33.82] 5.93
Wen-Jun Tu * 18 6 7 143 16.07 [7.52, 34.34] 6.56
Lang Wang @ 39 31 26 243 5.76 [3.78, 8.78] 8.24
Heterogeneity: τ² = 0.15, I² = 69.79%, H² = 3.31 5.82 [4.40, 7.71]
Test of θi = θj: Q(11) = 33.88, p = 0.00
Xiaobo Yang $ 9 3 23 17 1.30 [0.86, 1.99] 8.23
Yang Wang # 107 4 26 207 8.64 [6.00, 12.43] 8.48
Heterogeneity: τ² = 1.75, I² = 97.74%, H² = 44.25 3.37 [0.53, 21.47]
Test of θi = θj: Q(1) = 44.25, p = 0.00
Overall 5.42 [3.79, 7.77]
Heterogeneity: τ² = 0.36, I² = 86.24%, H² = 7.27
Test of θi = θj: Q(13) = 81.22, p = 0.00
Test of group differences: Qb(1) = 0.33, p = 0.57
1.00
Random-effects REML model

eFigure 33. Pooled Risk ratio of death in patients with Acute heart failure a) Forest plot

33a

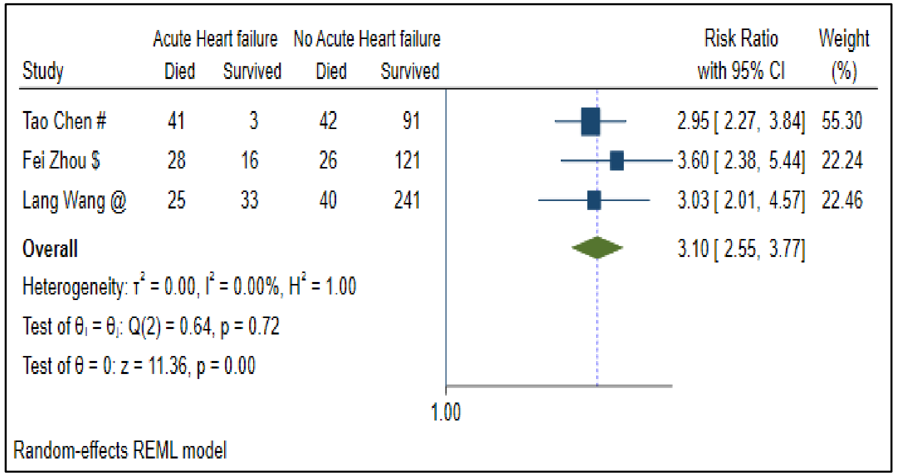

eFigure 34. Pooled Risk ratio of death in patients with DIC a) Forest plot b) Subgroup analysis

34a

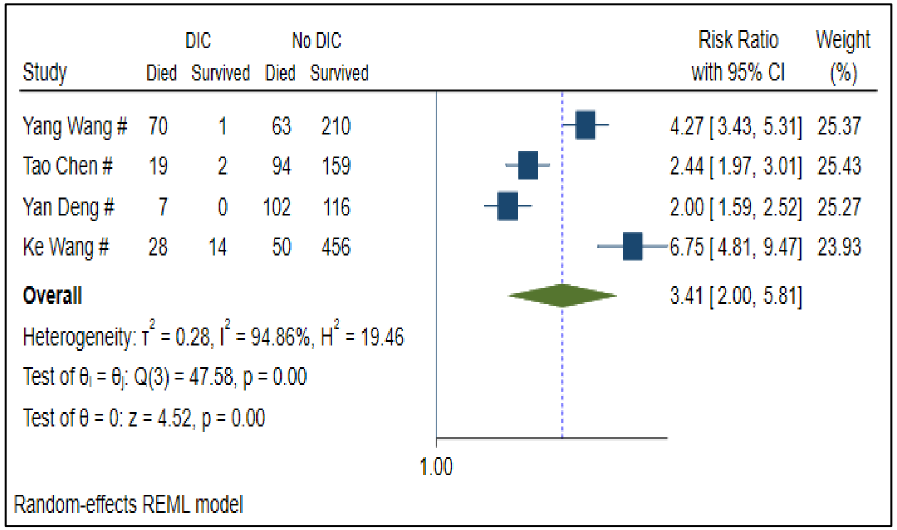

34b

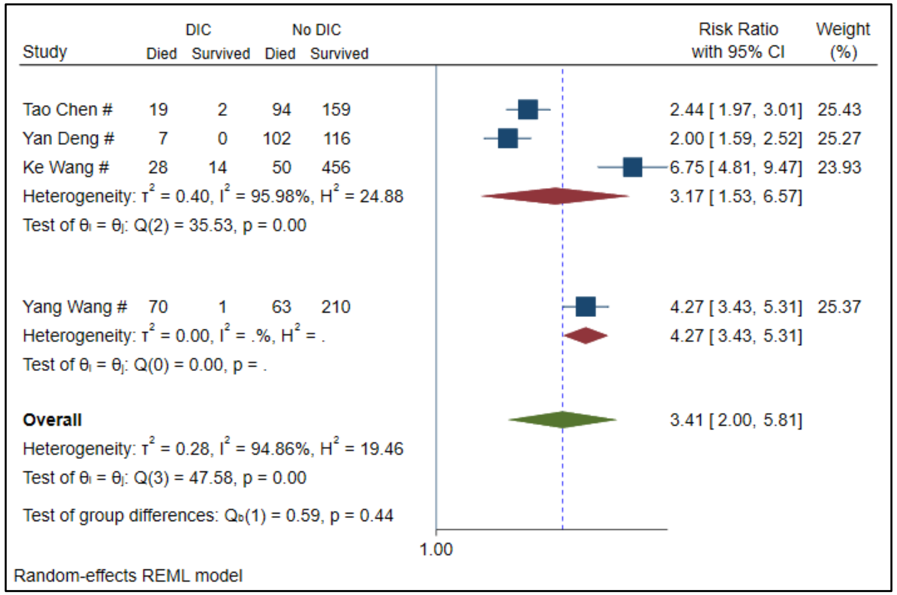
Study
DIC
Died Survived
No DIC
Died Survived
Risk Ratio with 95% CI
Weight (%)
Tao Chen # 19 2 94 159 2.44 [1.97, 3.01] 25.43
Yan Deng # 7 0 102 116 2.00 [1.59, 2.52] 25.27
Ke Wang # 28 14 50 456 6.75 [4.81, 9.47] 23.93
Heterogeneity: τ² = 0.40, I² = 95.98%, H² = 24.88 3.17 [1.53, 6.57]
Test of θi = θj: Q(2) = 35.53, p = 0.00
Yang Wang # 70 1 63 210 4.27 [3.43, 5.31] 25.37
Heterogeneity: τ² = 0.00, I² = .%, H² = . 4.27 [3.43, 5.31]
Test of θi = θj: Q(0) = 0.00, p = .
Overall 3.41 [2.00, 5.81]
Heterogeneity: τ² = 0.28, I² = 94.86%, H² = 19.46
Test of θi = θj: Q(3) = 47.58, p = 0.00
Test of group differences: Qb(1) = 0.59, p = 0.44
1.00
Random-effects REML model

eFigure 35. Pooled Risk ratio of death in patients with GI bleeding a) Forest plot b) Subgroup analysis

35a

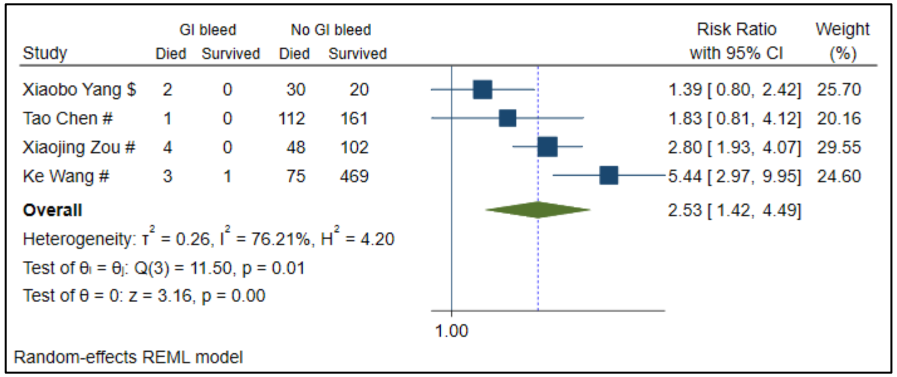

35b

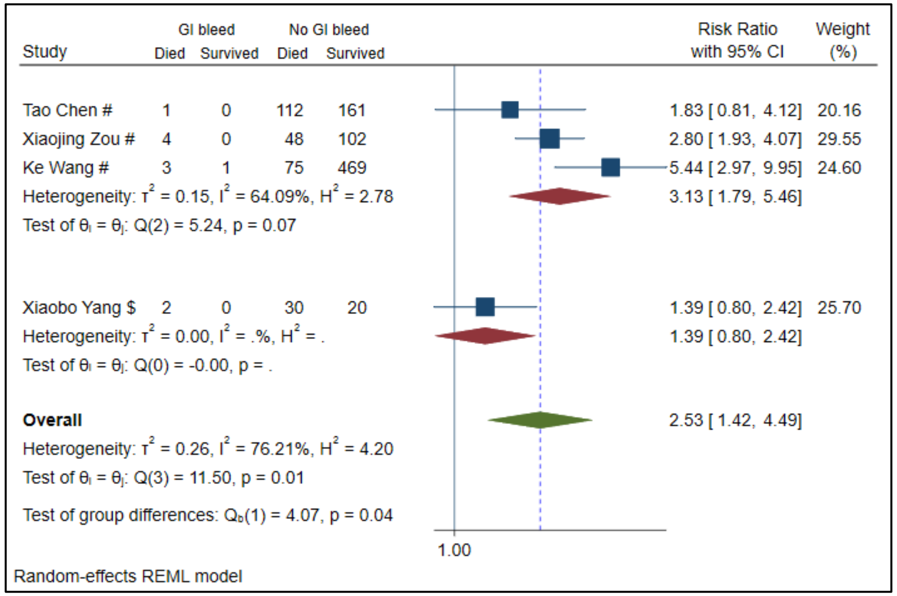

eFigure 36. Pooled Risk ratio of death in patients with Acute Kidney Injury a) Forest plot b) Funnel Plot c) Subgroup analysis.

36a

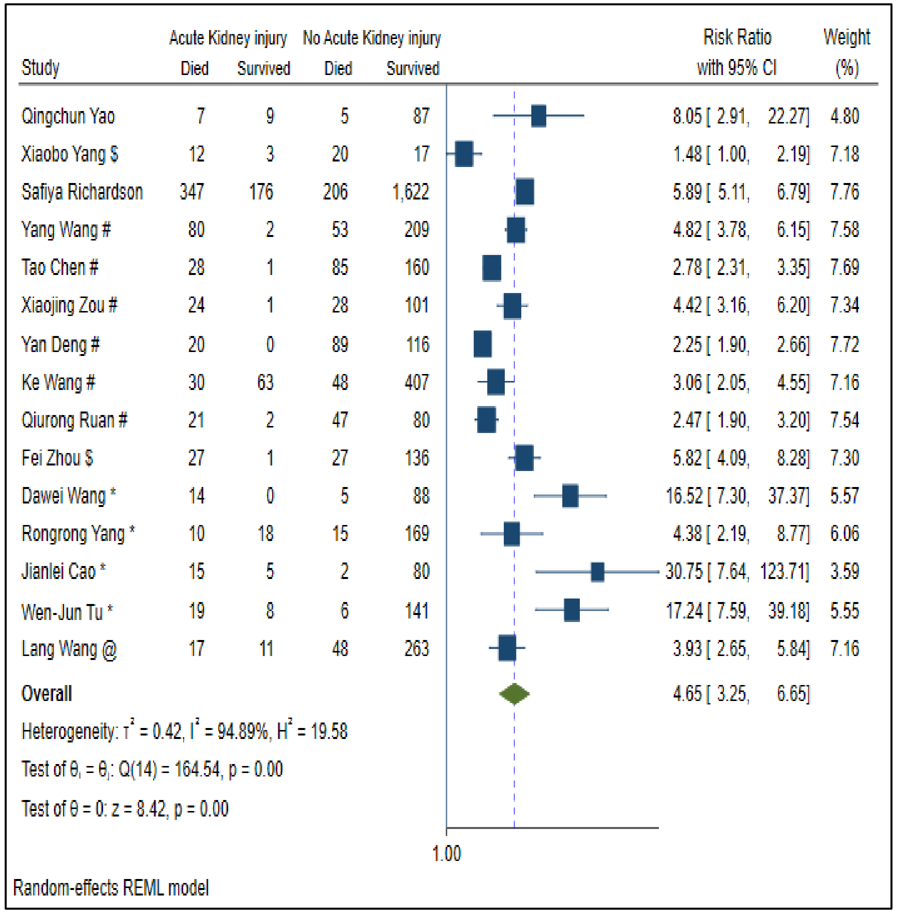

36b

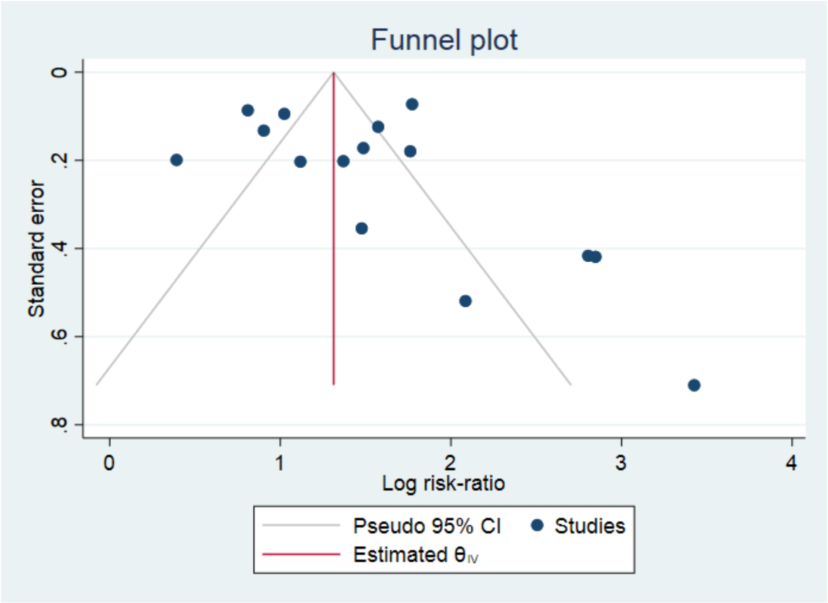

36c

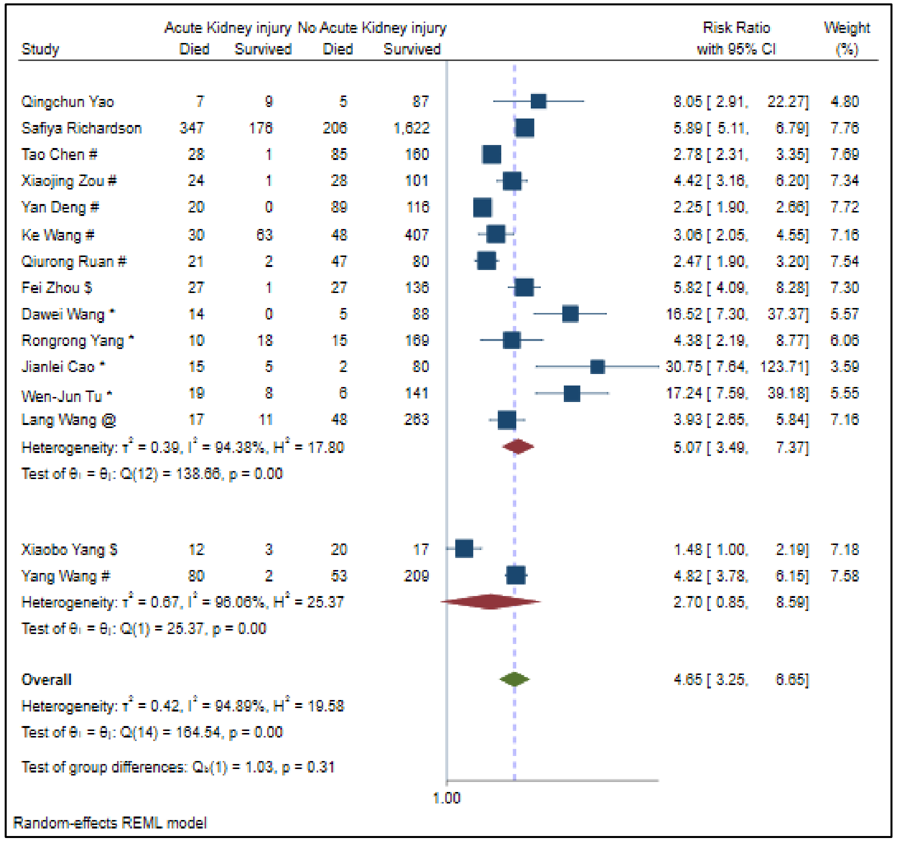
Study
Acute Kidney injury
Died
Survived
No Acute Kidney injury
Died
Survived
Risk Ratio with 95% CI
Weight (%)
Qingchun Yao 7 9 5 87 8.05 [2.91, 22.27] 4.80
Safiya Richardson 347 176 206 1,622 5.89 [5.11, 6.79] 7.76
Tao Chen # 28 1 85 160 2.78 [2.31, 3.35] 7.69
Xiaojing Zou # 24 1 28 101 4.42 [3.16, 6.20] 7.34
Yan Deng # 20 0 89 116 2.25 [1.90, 2.66] 7.72
Ke Wang # 30 63 48 407 3.06 [2.05, 4.55] 7.16
Qiurong Ruan # 21 2 47 80 2.47 [1.90, 3.20] 7.54
Fei Zhou $ 27 1 27 136 5.82 [4.09, 8.28] 7.30
Dawei Wang * 14 0 5 88 16.52 [7.30, 37.37] 5.57
Rongrong Yang * 10 18 15 169 4.38 [2.19, 8.77] 6.06
Jianlei Cao * 15 5 2 80 30.75 [7.64, 123.71] 3.59
Wen-Jun Tu * 19 8 6 141 17.24 [7.59, 39.18] 5.55
Lang Wang @ 17 11 48 263 3.93 [2.65, 5.84] 7.16
Heterogeneity: τ² = 0.39, I² = 94.38%, H² = 17.80 5.07 [3.49, 7.37]
Test of θᵢ = θⱼ: Q(12) = 138.66, p = 0.00
Xiaobo Yang $ 12 3 20 17 1.48 [1.00, 2.19] 7.18
Yang Wang # 80 2 53 209 4.82 [3.78, 6.15] 7.58
Heterogeneity: τ² = 0.67, I² = 96.06%, H² = 25.37 2.70 [0.85, 8.59]
Test of θᵢ = θⱼ: Q(1) = 25.37, p = 0.00
Overall 4.65 [3.25, 6.65]
Heterogeneity: τ² = 0.42, I² = 94.89%, H² = 19.58
Test of θᵢ = θⱼ: Q(14) = 164.54, p = 0.00
Test of group differences: Qb(1) = 1.03, p = 0.31
1.00
Random-effects REML model

eFigure 37. Pooled Risk ratio of death in patients with liver dysfunction a) Forest plot b) Funnel Plot c) Subgroup analysis

37a

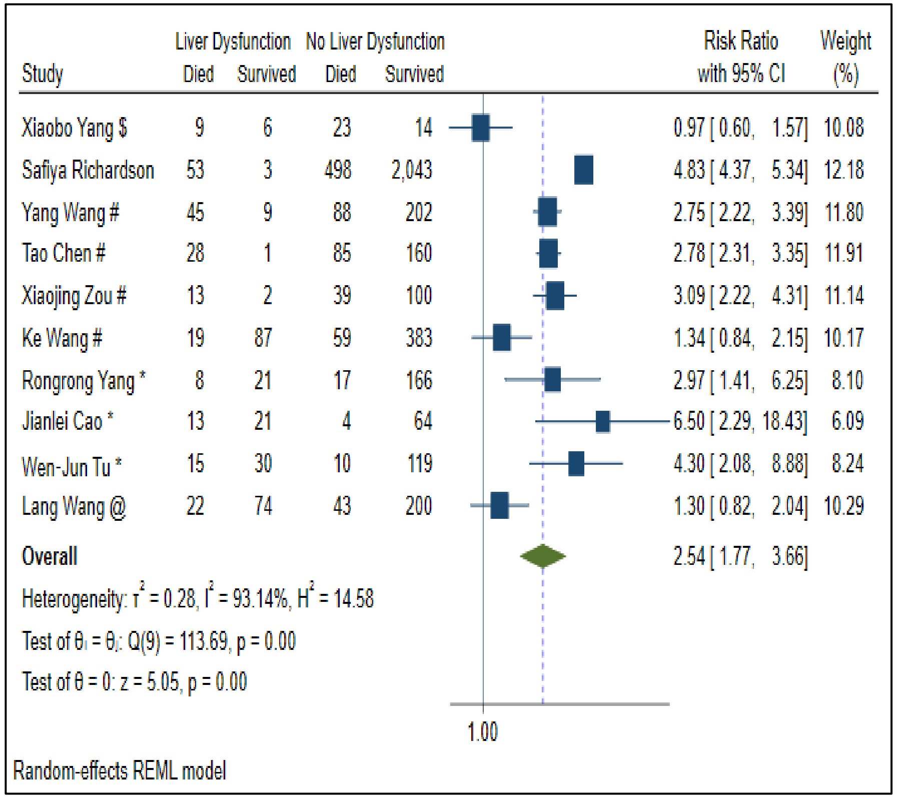

37b

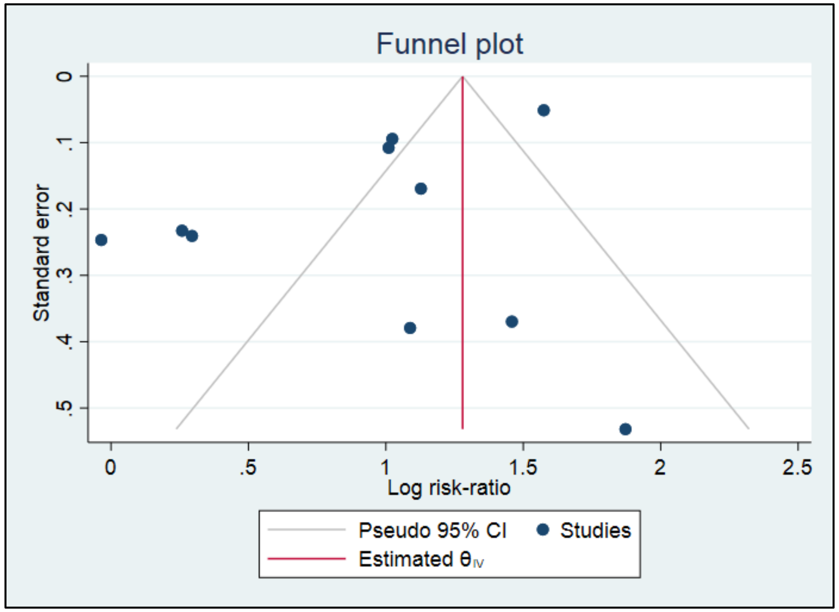

37c

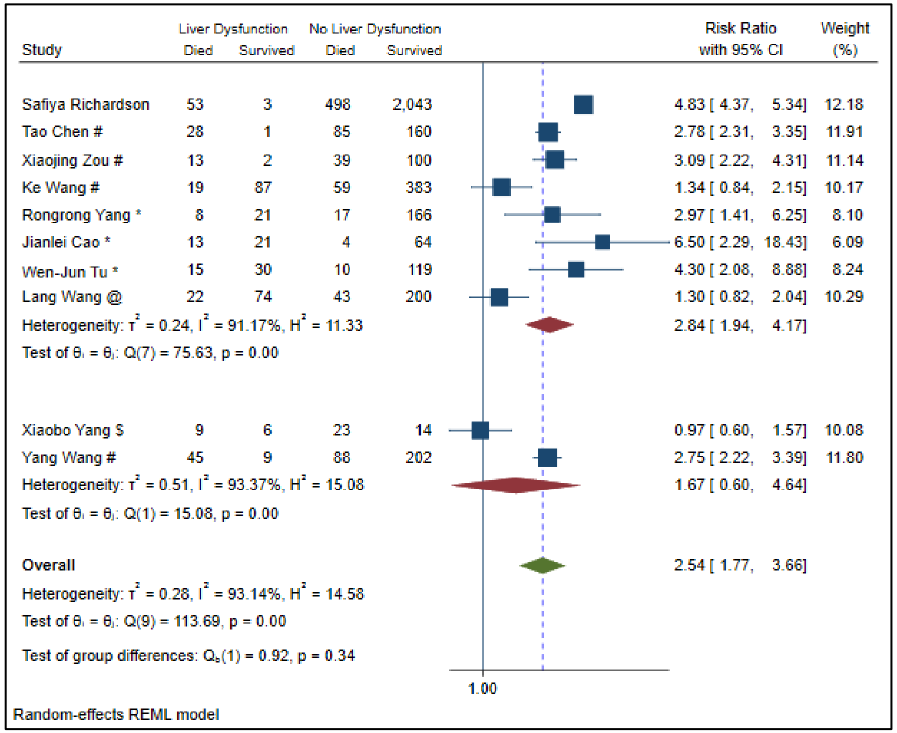
Study
Liver Dysfunction
Died
Survived
No Liver Dysfunction
Died
Survived
Risk Ratio with 95% CI
Weight (%)
Safiya Richardson 53 3 498 2,043 4.83 [4.37, 5.34] 12.18
Tao Chen # 28 1 85 160 2.78 [2.31, 3.35] 11.91
Xiaojing Zou # 13 2 39 100 3.09 [2.22, 4.31] 11.14
Ke Wang # 19 87 59 383 1.34 [0.84, 2.15] 10.17
Rongrong Yang * 8 21 17 166 2.97 [1.41, 6.25] 8.10
Jianlei Cao * 13 21 4 64 6.50 [2.29, 18.43] 6.09
Wen-Jun Tu * 15 30 10 119 4.30 [2.08, 8.88] 8.24
Lang Wang @ 22 74 43 200 1.30 [0.82, 2.04] 10.29
Heterogeneity: τ^2 = 0.24, I^2 = 91.17%, H^2 = 11.33 2.84 [1.94, 4.17]
Test of $\theta_i = \theta_j$: Q(7) = 75.63, p = 0.00
Xiaobo Yang $ 9 6 23 14 0.97 [0.60, 1.57] 10.08
Yang Wang # 45 9 88 202 2.75 [2.22, 3.39] 11.80
Heterogeneity: τ^2 = 0.51, I^2 = 93.37%, H^2 = 15.08 1.67 [0.60, 4.64]
Test of $\theta_i = \theta_j$: Q(1) = 15.08, p = 0.00
Overall 2.54 [1.77, 3.66]
Heterogeneity: τ^2 = 0.28, I^2 = 93.14%, H^2 = 14.58
Test of $\theta_i = \theta_j$: Q(9) = 113.69, p = 0.00
Test of group differences: $Q_b(1)$ = 0.92, p = 0.34
1.00
Random-effects REML model

eFigure 38. Pooled Risk ratio of death in patients with Decreased TLC - Forest plot

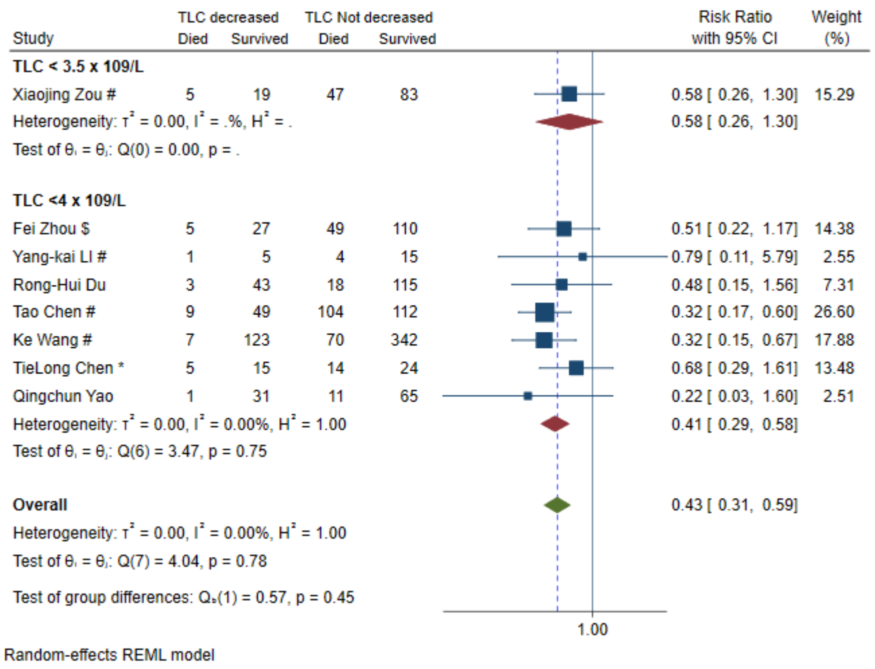

eFigure 39. Pooled Risk ratio of death in patients with Increased TLC – Forest plot

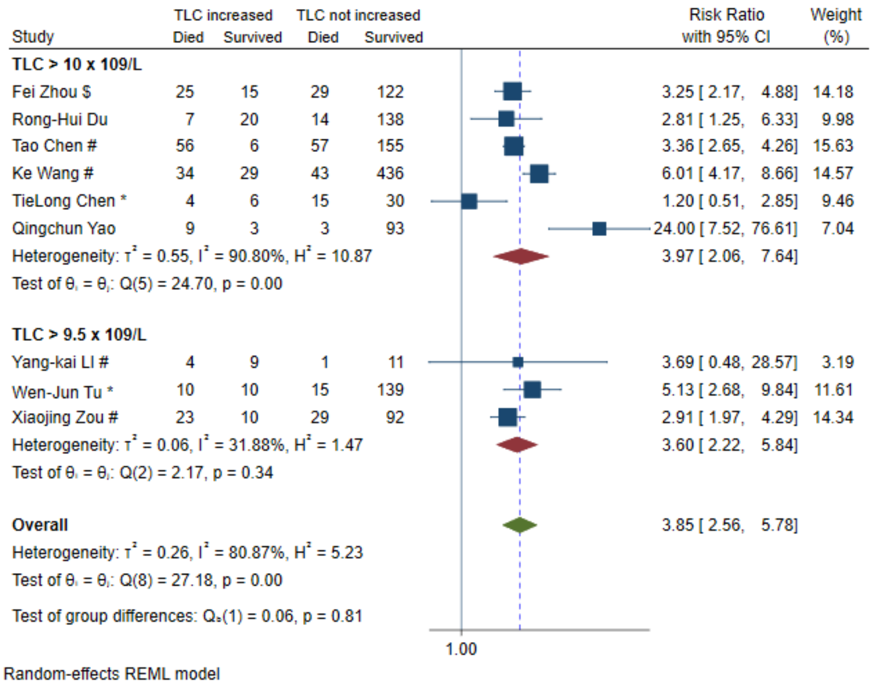

eFigure 40. Pooled Risk ratio of death in patients with Decreased Neutrophil - Forest plot

Study	Neutrophil count decreased: Died	Neutrophil count decreased: Survived	Died	Survived	Risk Ratio with 95% CI	Weight (%)
Rong-Hui Du	2	16	19	142	0.94 [0.24, 3.72]	54.73
Ke Wang #	1	66	76	399	0.09 [0.01, 0.66]	45.27
**Overall**					0.33 [0.03, 3.15]	

Heterogeneity: $\tau^2 = 1.93$, $I^2 = 72.18\%$, $H^2 = 3.59$

Test of $\theta_i = \theta_j$: Q(1) = 3.59, p = 0.06

Test of $\theta = 0$: z = -0.96, p = 0.34

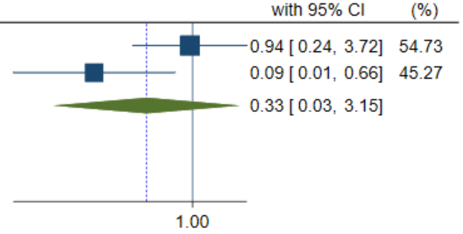

Random-effects DerSimonian-Laird model

eFigure 41. Pooled Risk ratio of death in patients with Increased Neutrophil - Forest plot

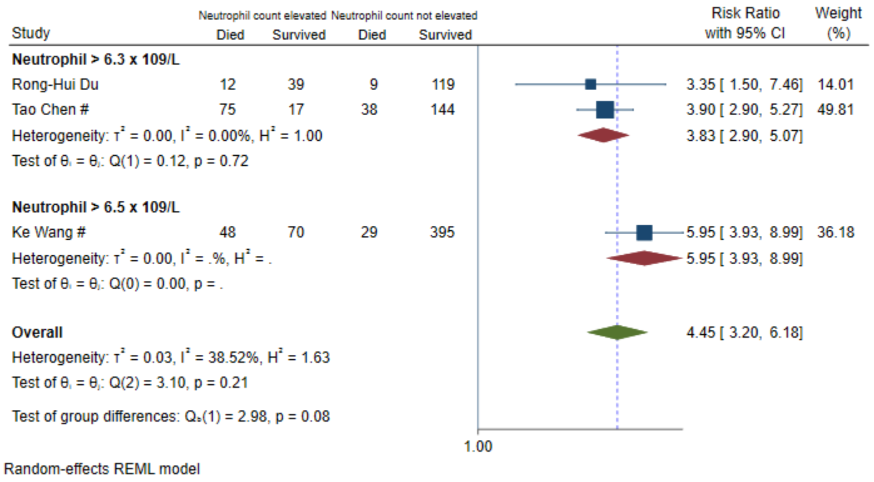

eFigure 42. Pooled Risk ratio of death in patients with Decreased lymphocyte - Forest plot.

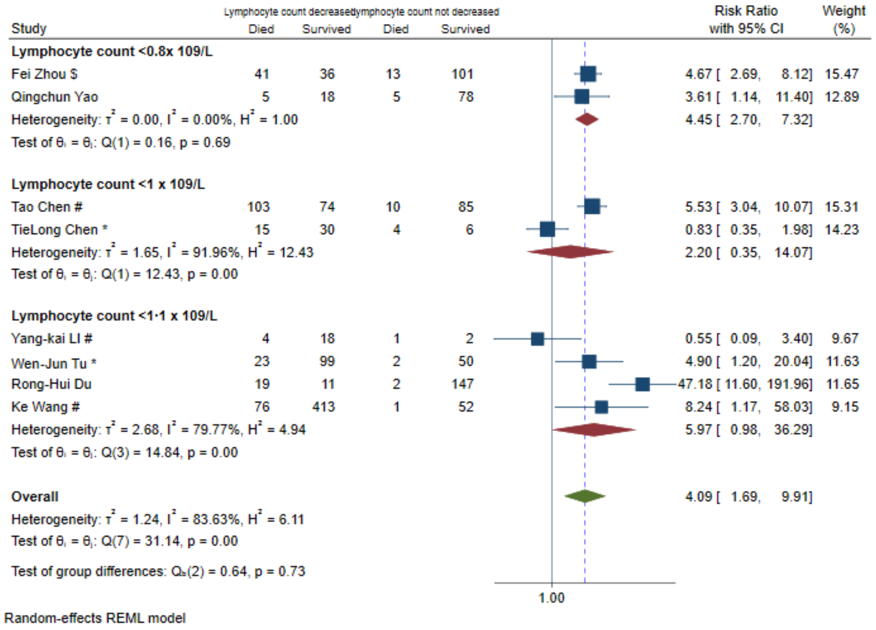

eFigure 43. Pooled Risk ratio of death in patients with Decreased Platelet - Forest plot

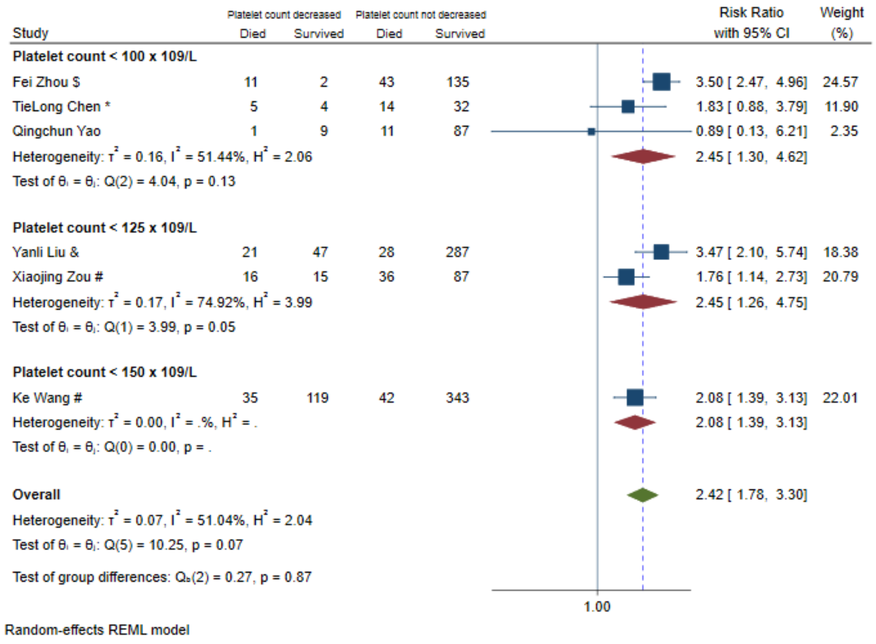

eFigure 44. Pooled Risk ratio of death in patients with Decreased Albumin - Forest plot

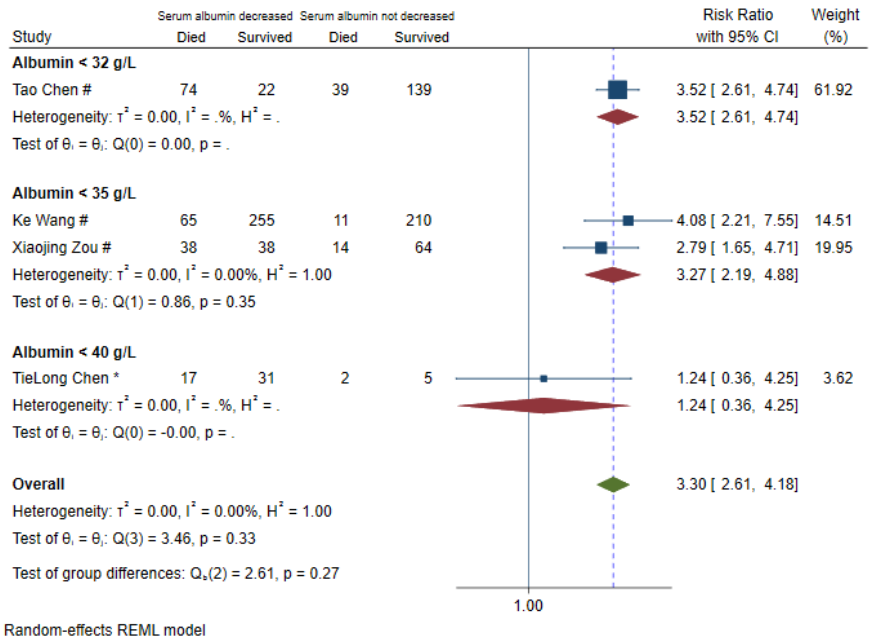

eFigure 45. Pooled Risk ratio of death in patients with Increased Globulin - Forest plot

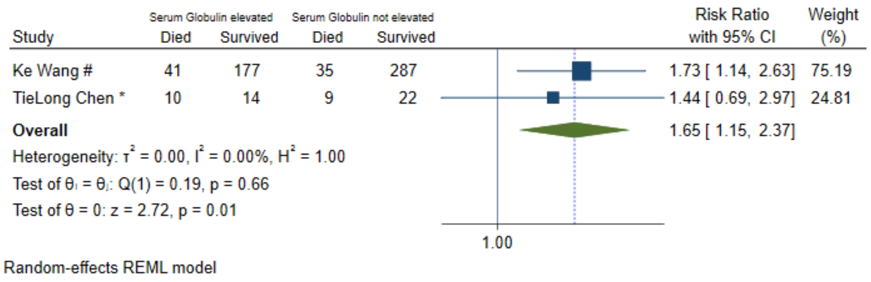

eFigure 46. Pooled Risk ratio of death in patients with Increased Globulin - Forest plot

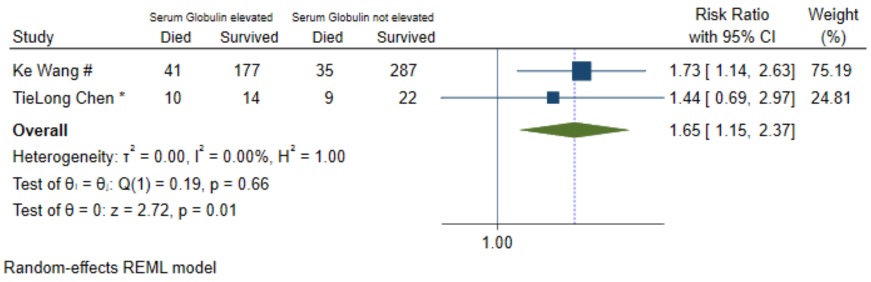

eFigure 47. Pooled Risk ratio of death in patients with Increased T Bilirubin - Forest plot

Study	Serum Bilirubin elevated Died	Serum Bilirubin elevated Survived	Serum Bilirubin not elevated Died	Serum Bilirubin not elevated Survived	Risk Ratio with 95% CI	Weight (%)
Ke Wang #	10	14	66	451	3.26 [1.93, 5.51]	40.33
Qingchun Yao	4	9	8	87	3.65 [1.28, 10.45]	10.04
Xiaojing Zou #	7	3	45	99	2.24 [1.40, 3.59]	49.64
**Overall**					2.74 [1.96, 3.82]	

Heterogeneity: $\tau^2 = 0.00$, $I^2 = 0.00\%$, $H^2 = 1.00$

Test of $\theta_i = \theta_j$: $Q(2) = 1.41$, $p = 0.49$

Test of $\theta = 0$: $z = 5.93$, $p = 0.00$

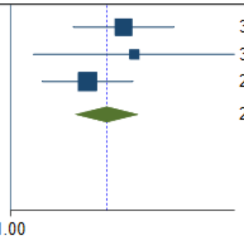

Random-effects REML model

eFigure 48. Pooled Risk ratio of death in patients with Increased AST- Forest plot.

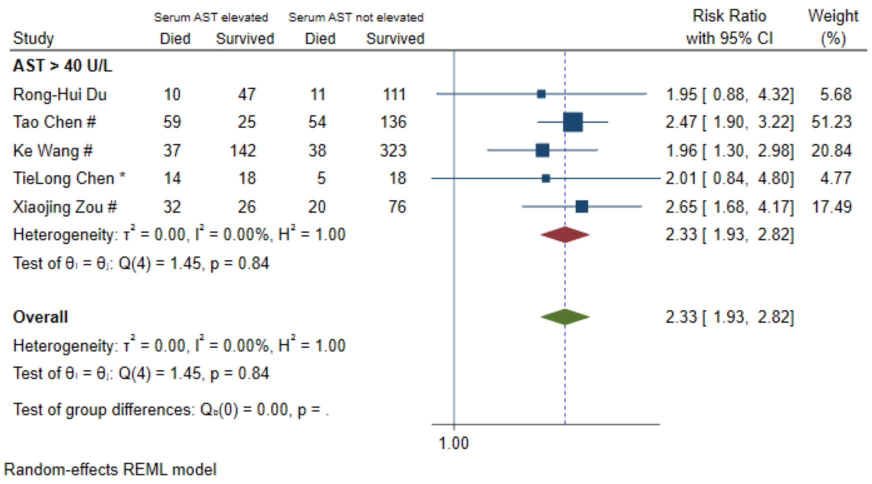

eFigure 49. Pooled Risk ratio of death in patients with Increased ALT- Forest plot.

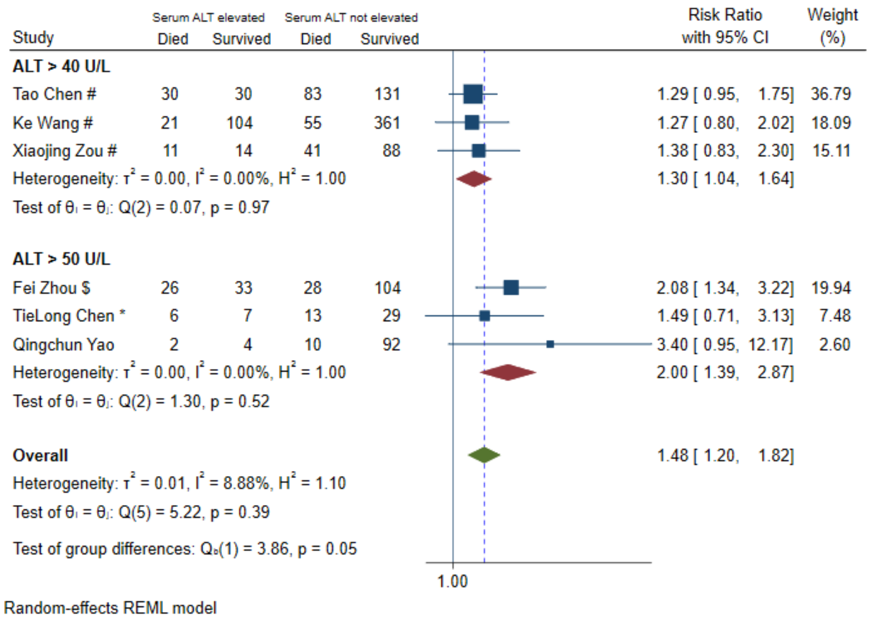

eFigure 50. Pooled Risk ratio of death in patients with Increased PT- Forest plot.

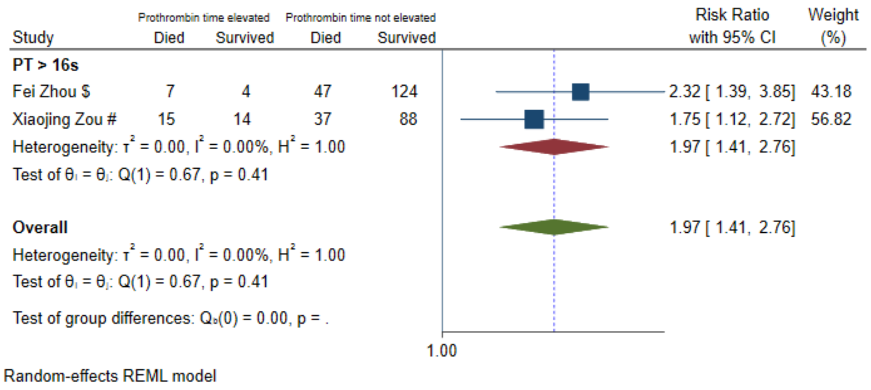

eFigure 51. Pooled Risk ratio of death in patients with Increased CK Total- Forest plot

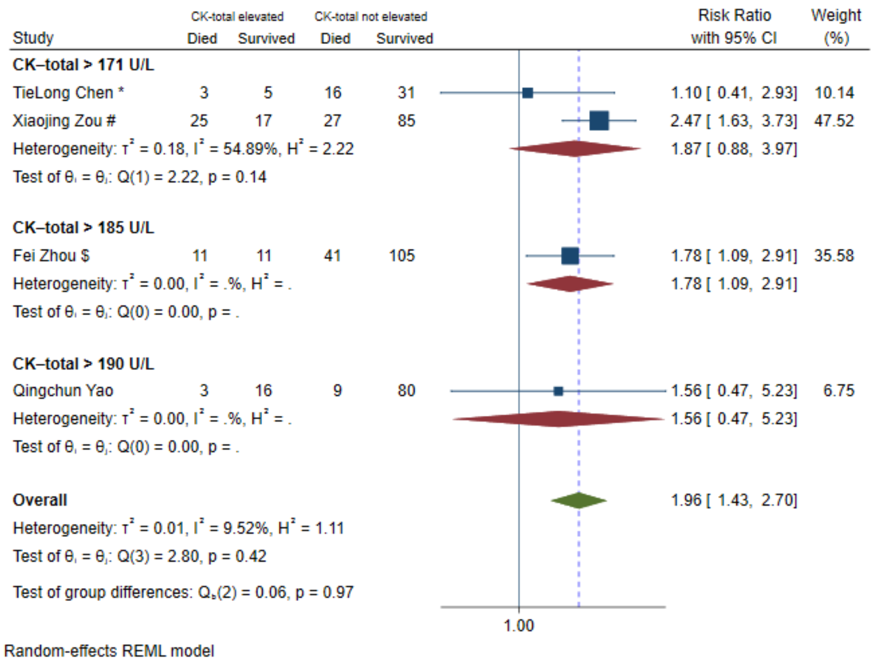

eFigure 52. Pooled Risk ratio of death in patients with Increased CK MB- Forest plot

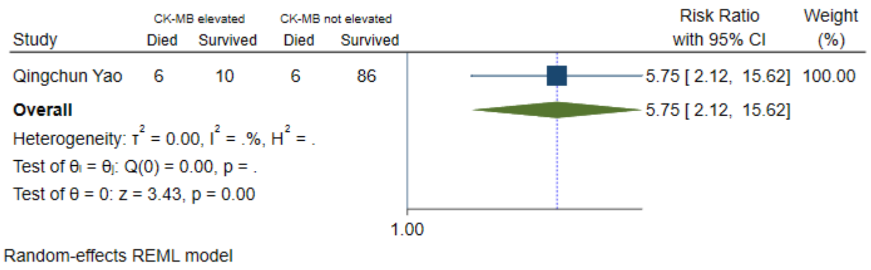

eFigure 53. Pooled Risk ratio of death in patients with Increased BUN- Forest plot

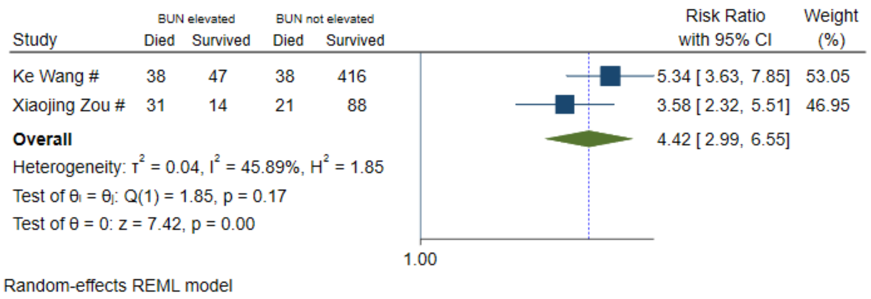

eFigure 54. Pooled Risk ratio of death in patients with Increased Creatinine- Forest plot

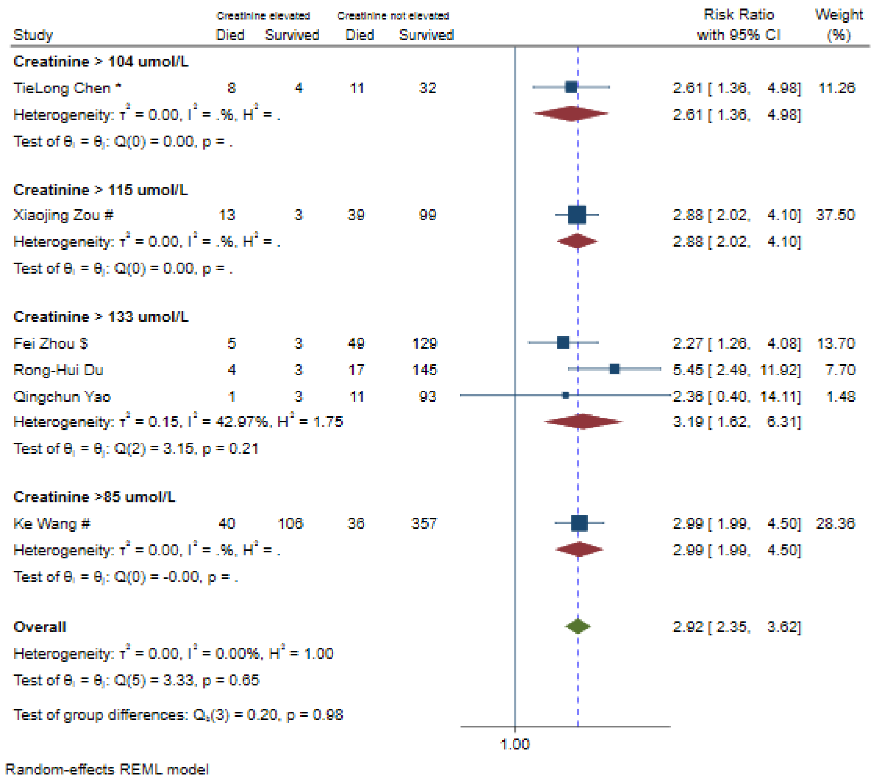

eFigure 55. Pooled Risk ratio of death in patients with Increased Na^+ - Forest plot

Study	Serum Sodium elevated Died	Serum Sodium elevated Survived	Serum Sodium not elevated Died	Serum Sodium not elevated Survived	Risk Ratio with 95% CI	Weight (%)
**Na+ > 145 mmol/L**						
Tao Chen #	20	3	93	158	2.35 [1.87, 2.94]	54.32
Xiaojing Zou #	9	0	43	102	3.19 [2.39, 4.25]	45.68
Heterogeneity: $\tau^2 = 0.03$, $I^2 = 63.06\%$, $H^2 = 2.71$					2.70 [2.00, 3.64]	
Test of $\theta_i = \theta_j$: Q(1) = 2.71, p = 0.10						
**Overall**					2.70 [2.00, 3.64]	
Heterogeneity: $\tau^2 = 0.03$, $I^2 = 63.06\%$, $H^2 = 2.71$						
Test of $\theta_i = \theta_j$: Q(1) = 2.71, p = 0.10						
Test of group differences: $Q_b(0) = 0.00$, p = .						

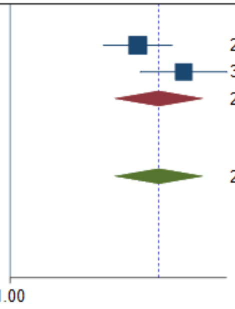

Random-effects REML model

eFigure 56. Pooled Risk ratio of death in patients with Increased K^+ - Forest plot

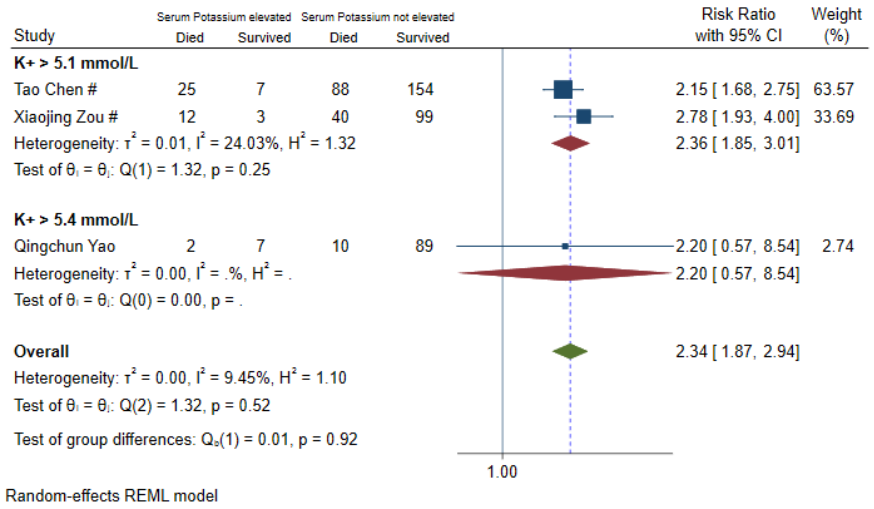

eFigure 57. Pooled Risk ratio of death in patients with Elevated LDH - Forest plot

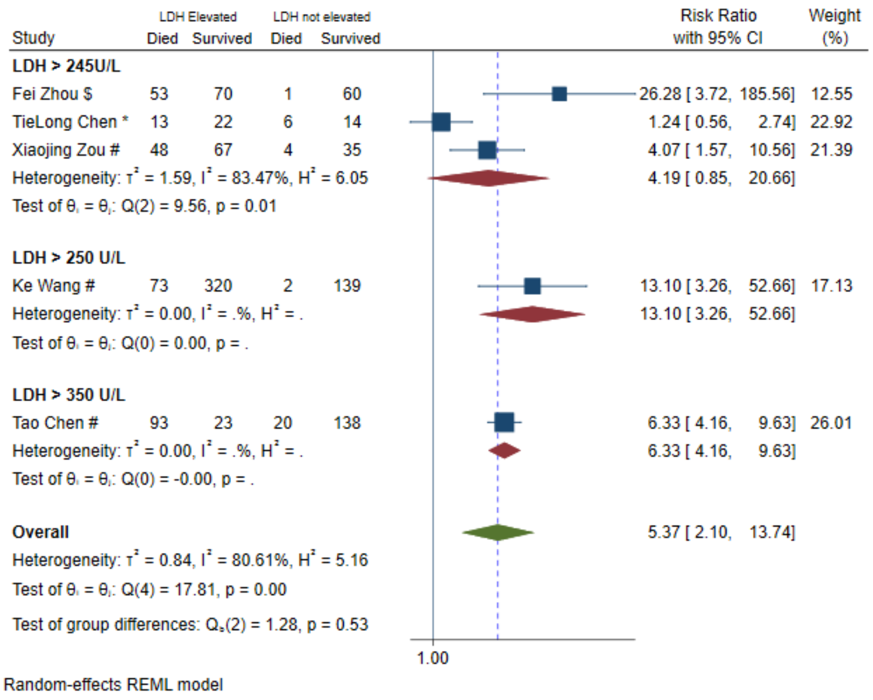

eFigure 58. Pooled Risk ratio of death in patients with Elevated D-Dimer - Forest plot

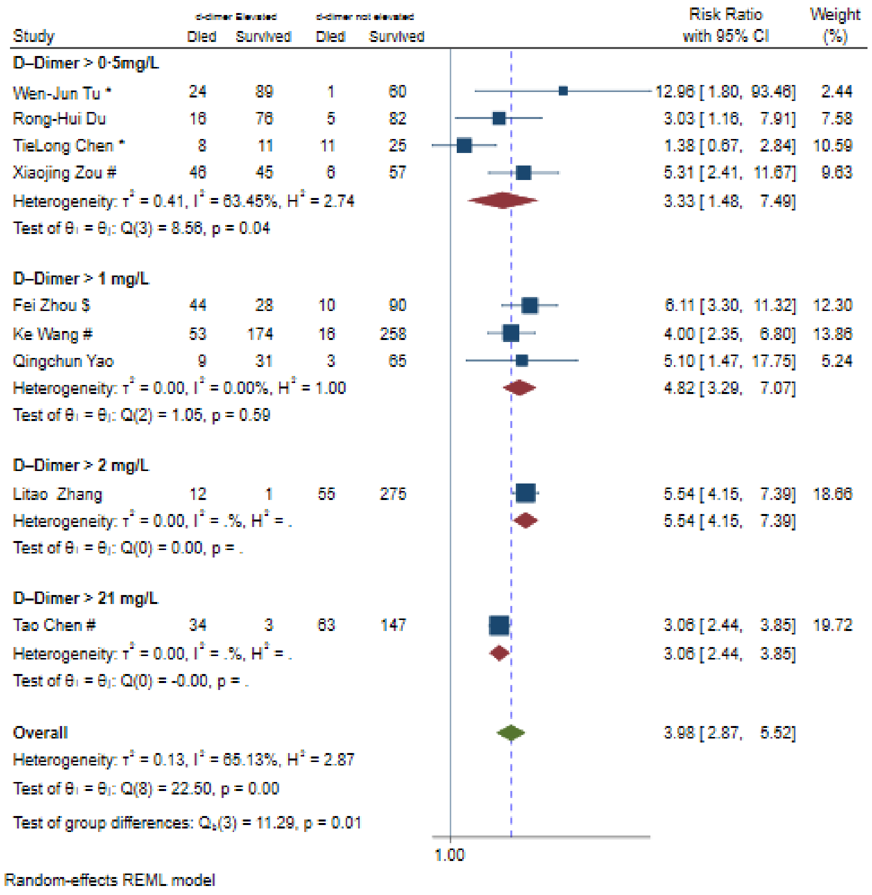

eFigure 59. Pooled Risk ratio of death in patients with Increased CRP - Forest plot

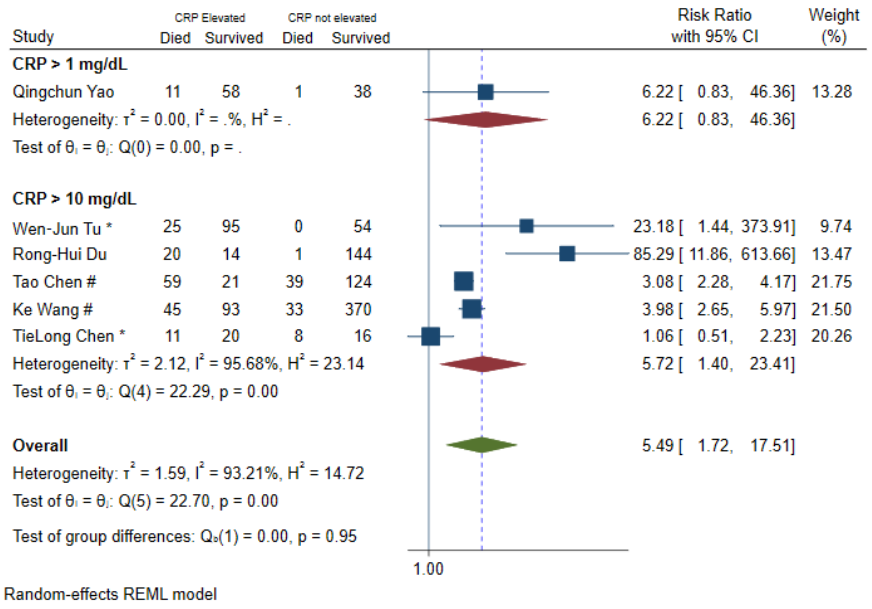

eFigure 60. Pooled Risk ratio of death in patients with Elevated ESR - Forest plot

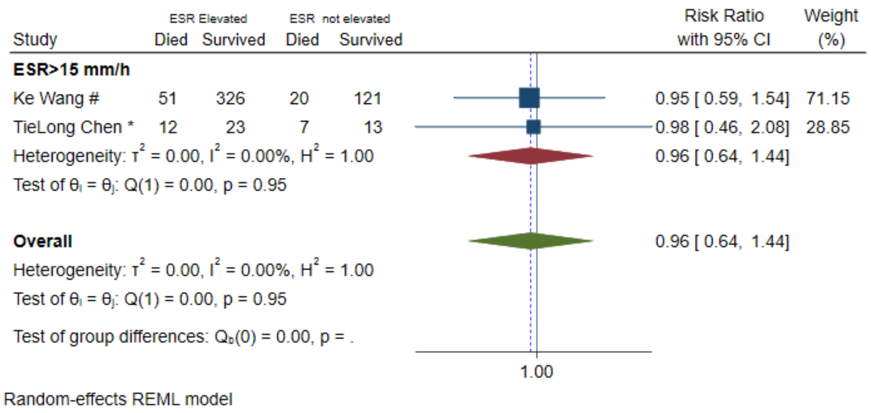

eFigure 61. Pooled Risk ratio of death in patients with Elevated Procalcitonin - Forest plot

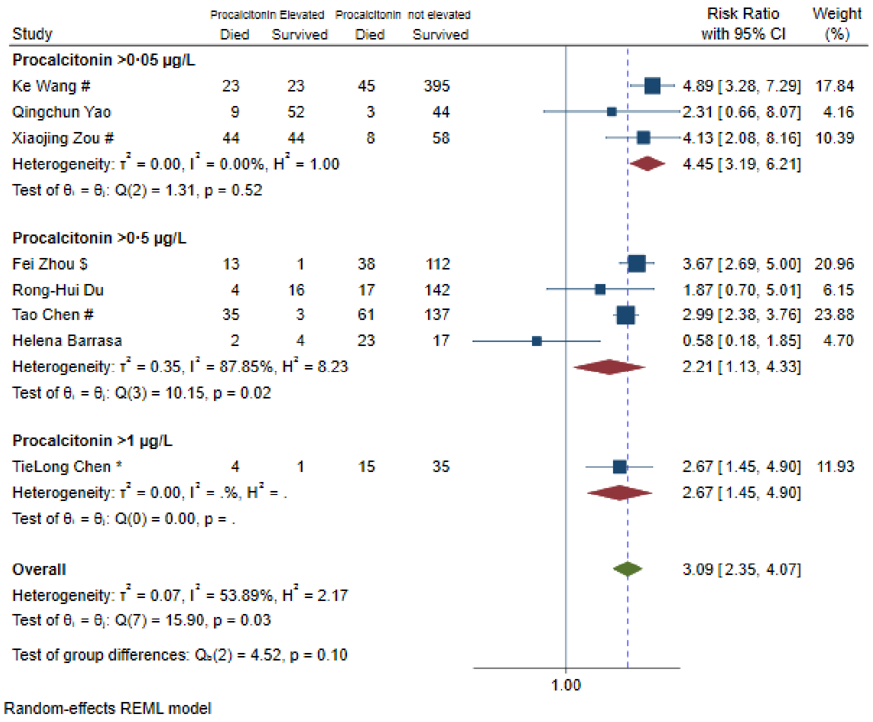

eFigure 62. Pooled Risk ratio of death in patients with Increased IL-6- Forest plot

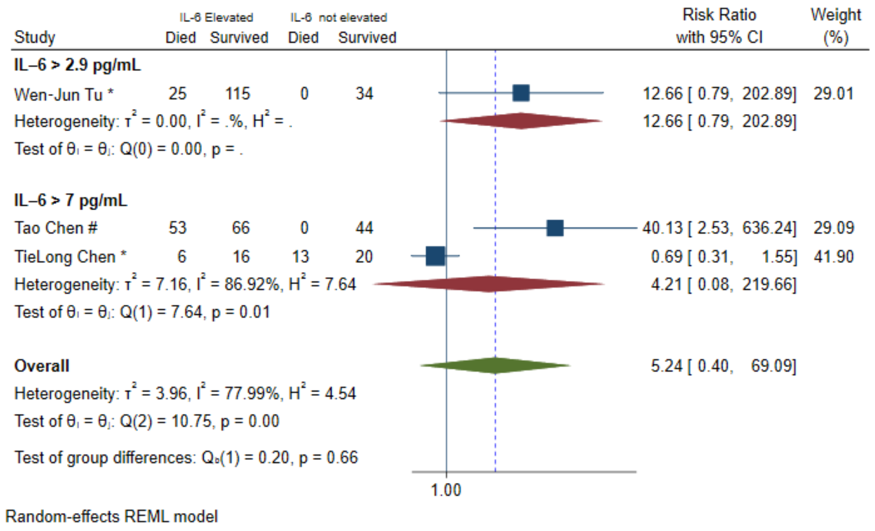

eFigure 63. Pooled Risk ratio of death in patients with Increased NT ProBNP- Forest plot

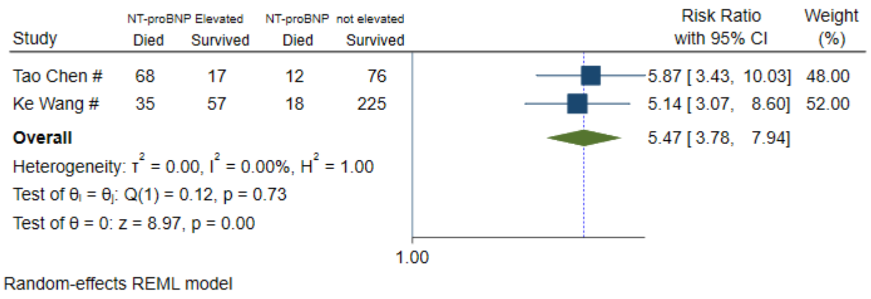

eFigure 64. Pooled Risk ratio of death in patients with Elevated Troponin- Forest plot

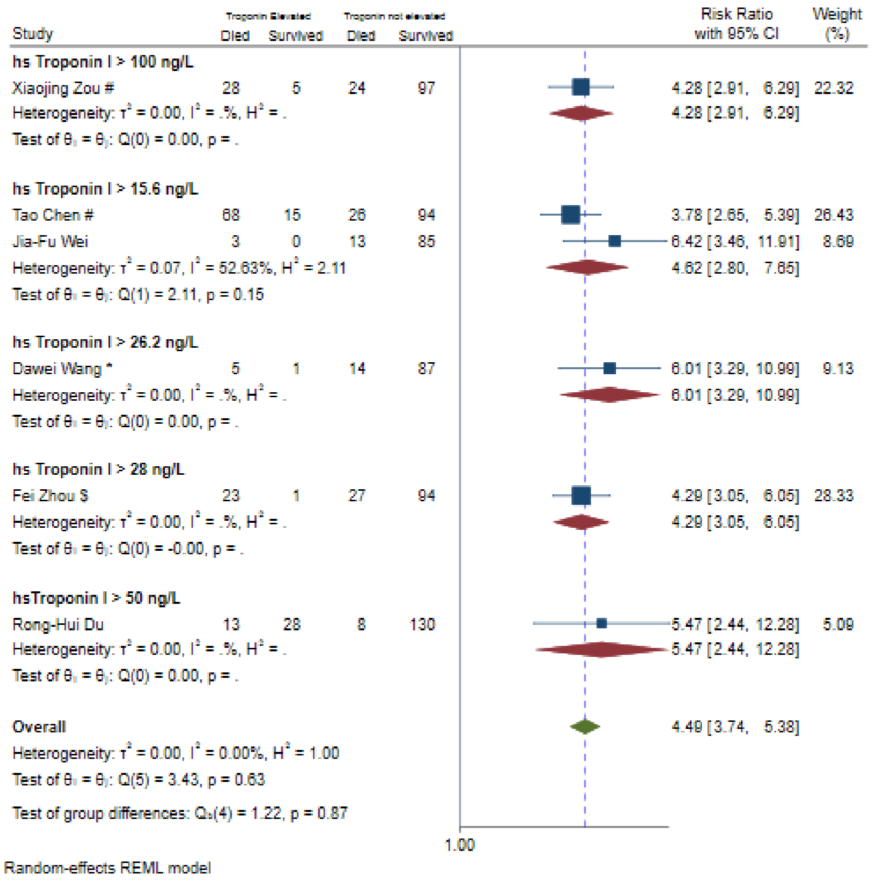

Figure 65. Pooled Risk ratio of death in patients with bilateral lung involvement a) Forest plot b) Funnel Plot.

65a

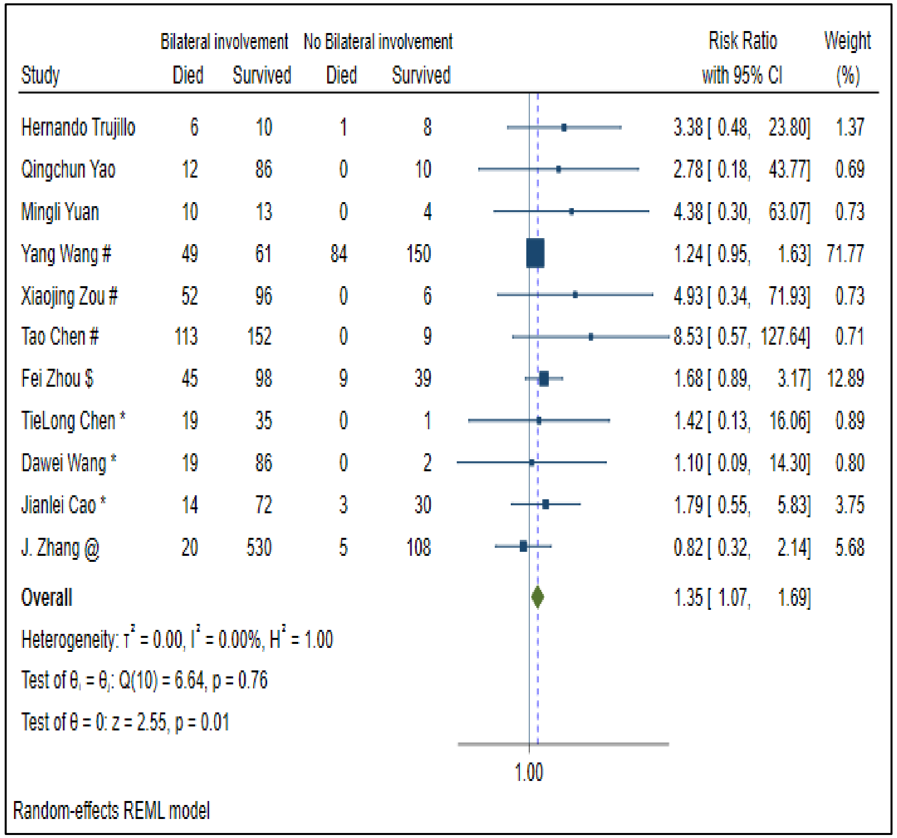

65b

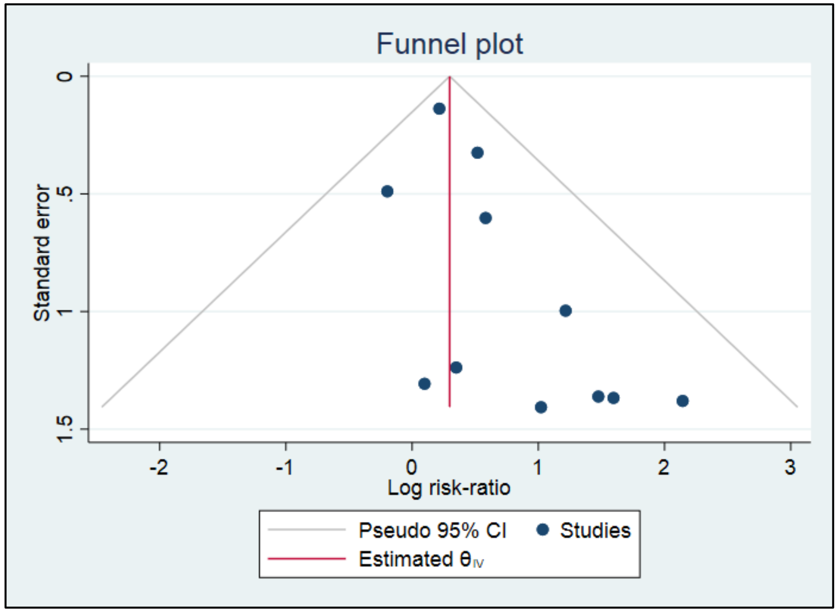

eFigure 66. Pooled Risk ratio of death in patients with consolidation -Forest plot

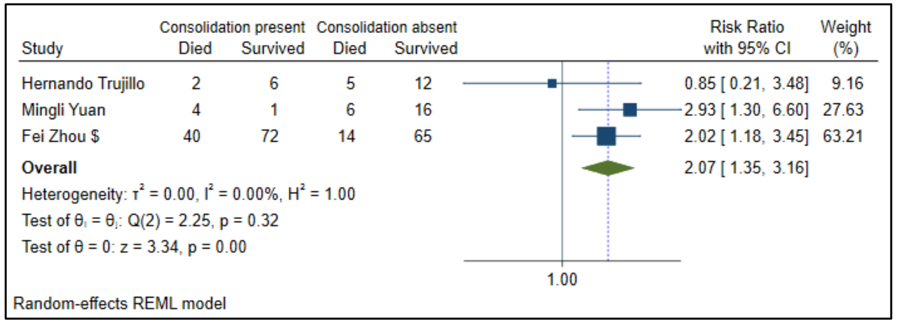

eFigure 67. Pooled Risk ratio of death in patients with ground glass opacity- Forest plot

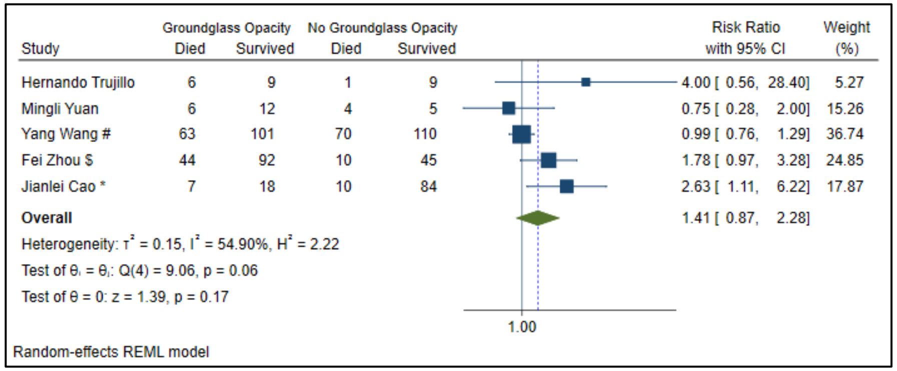

eFigure 68. Pooled Risk ratio of death in patients with unifocal involvement -Forest plot.

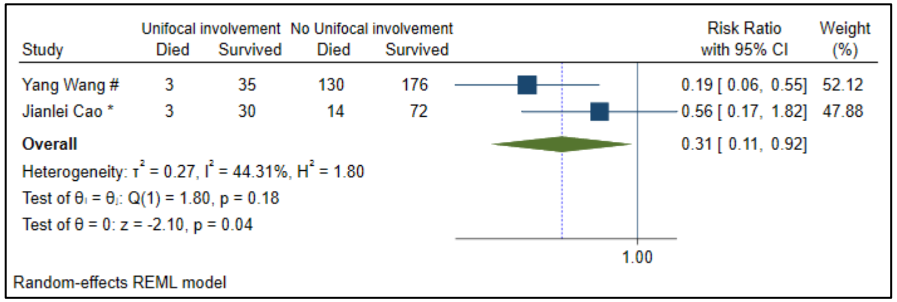

eFigure 69. Pooled Risk ratio of death in patients with pleural effusion- Forest plot

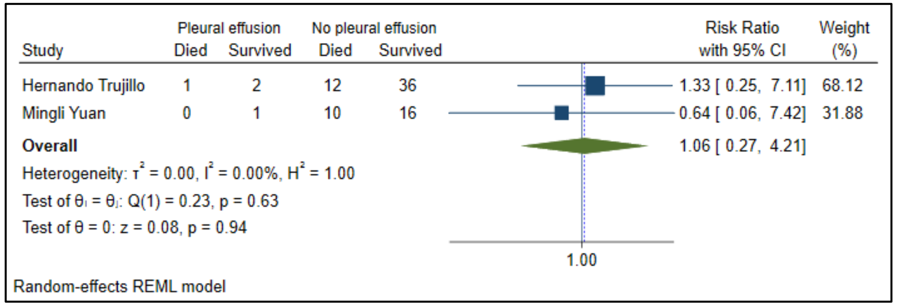

Association
with Severe disease

eFigure 70. Pooled Odds ratio of severe disease in patients with male gender. a) Forest plot b) Funnel Plot

70a

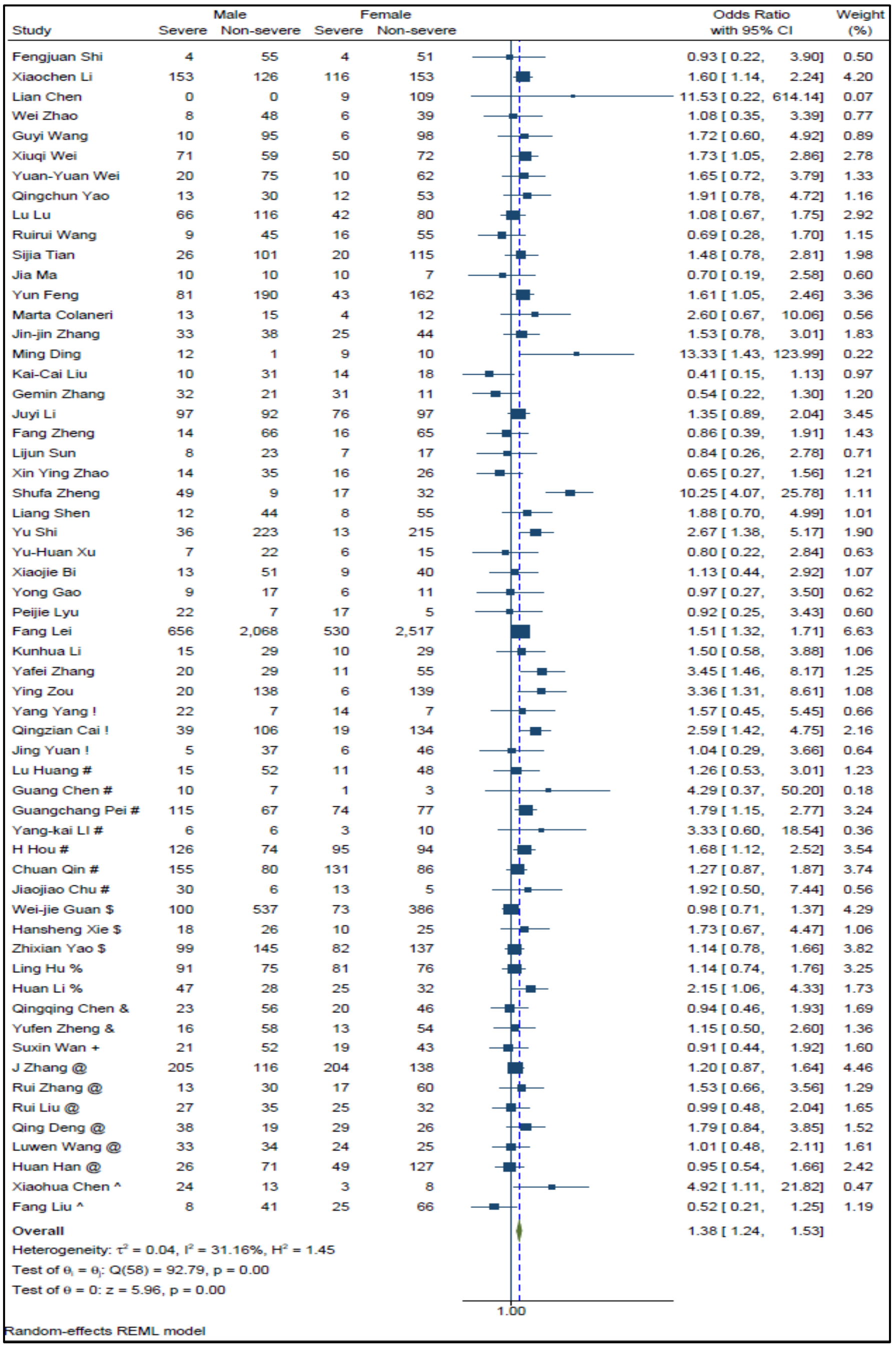

70b

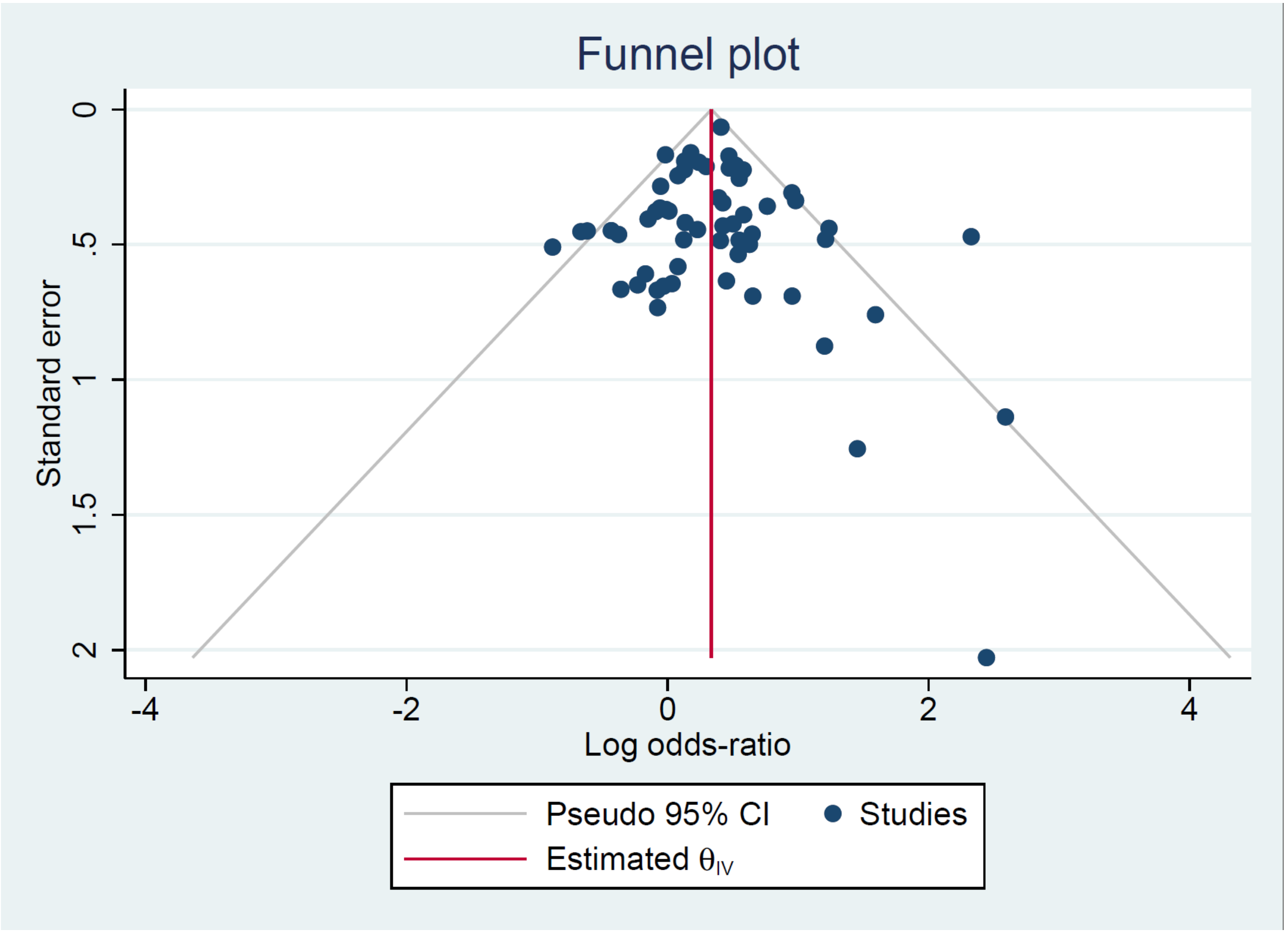

Funnel plot
Standard error
0
.5
1
1.5
2
-4
-2
0
2
4
Log odds-ratio
Pseudo 95% CI
Studies
Estimated θIV

eFigure 71. Pooled Odds ratio of severe disease in patients who was ever smoker. a) Forest plot b) Funnel Plot

71a

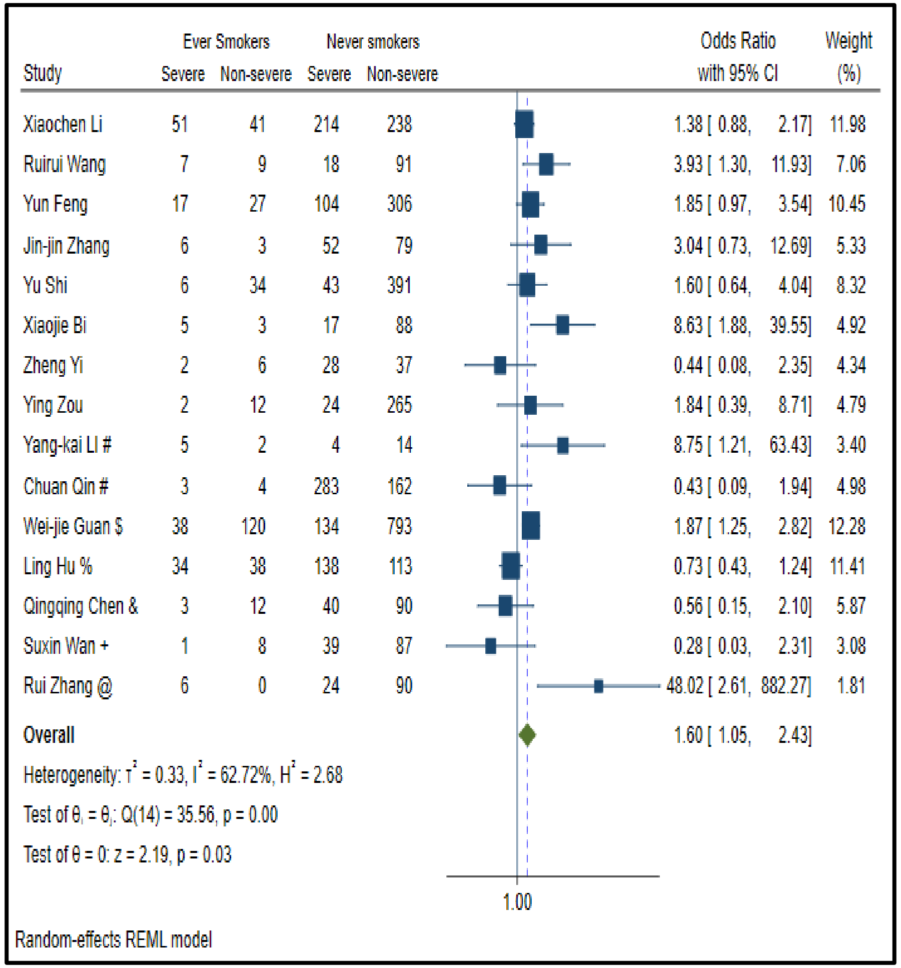

71b

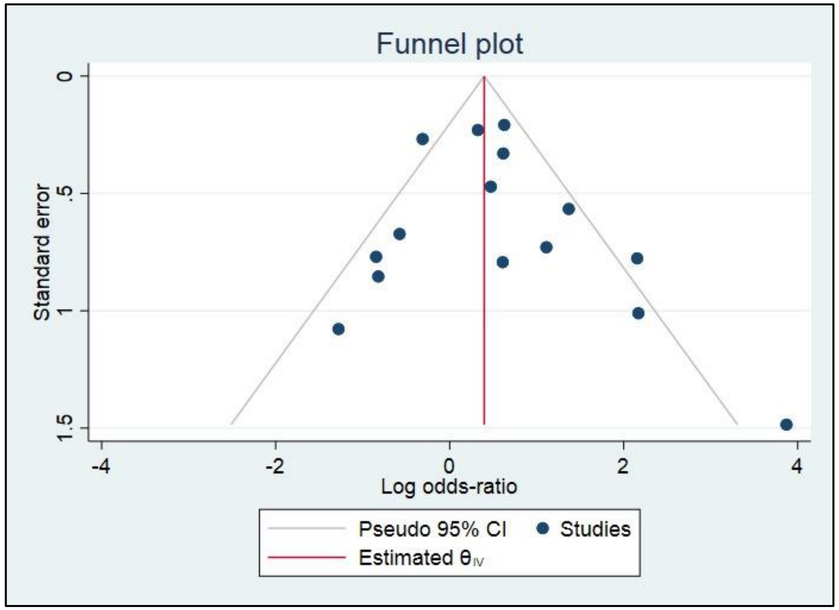

eFigure 72 Pooled Odds ratio of severe disease in patients with diabetes mellitus. a) Forest plot b) Funnel Plot

72a

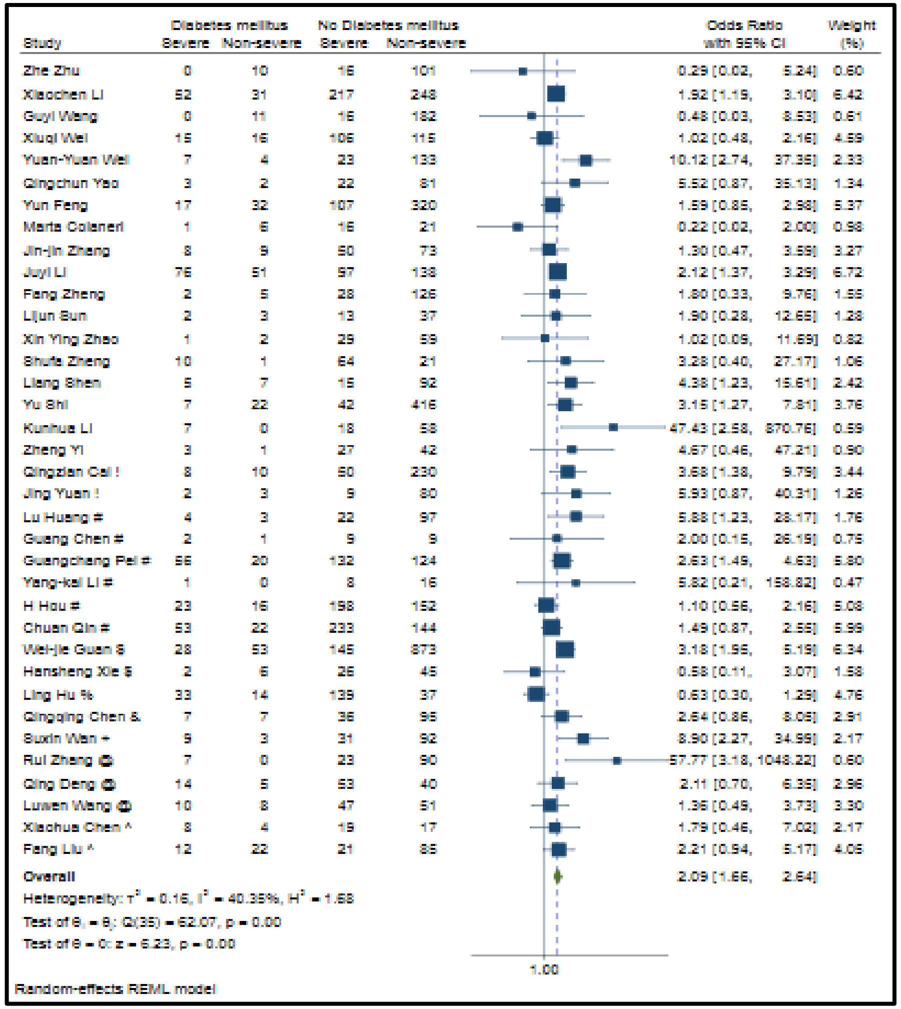

72b

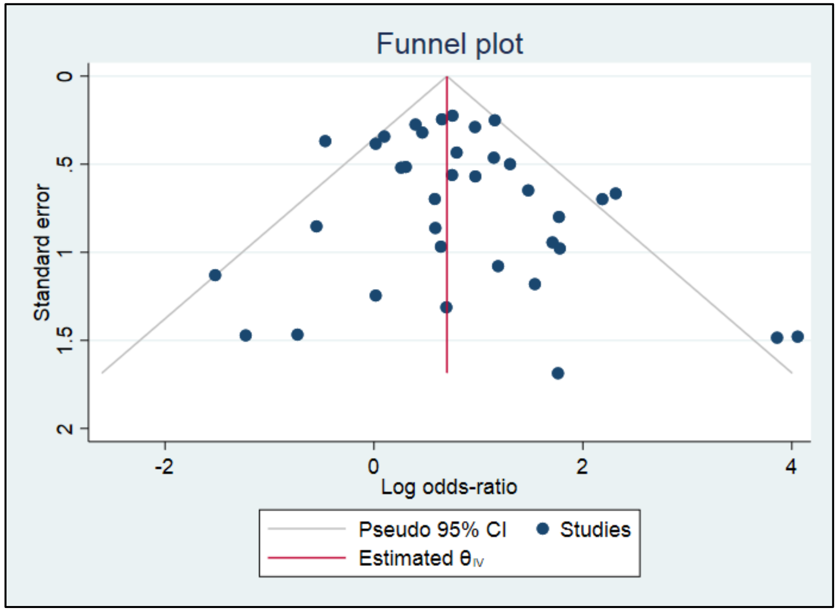

eFigure 73 Pooled Odds ratio of severe disease in patients with hypertension. a) Forest plot b) Funnel Plot

73a

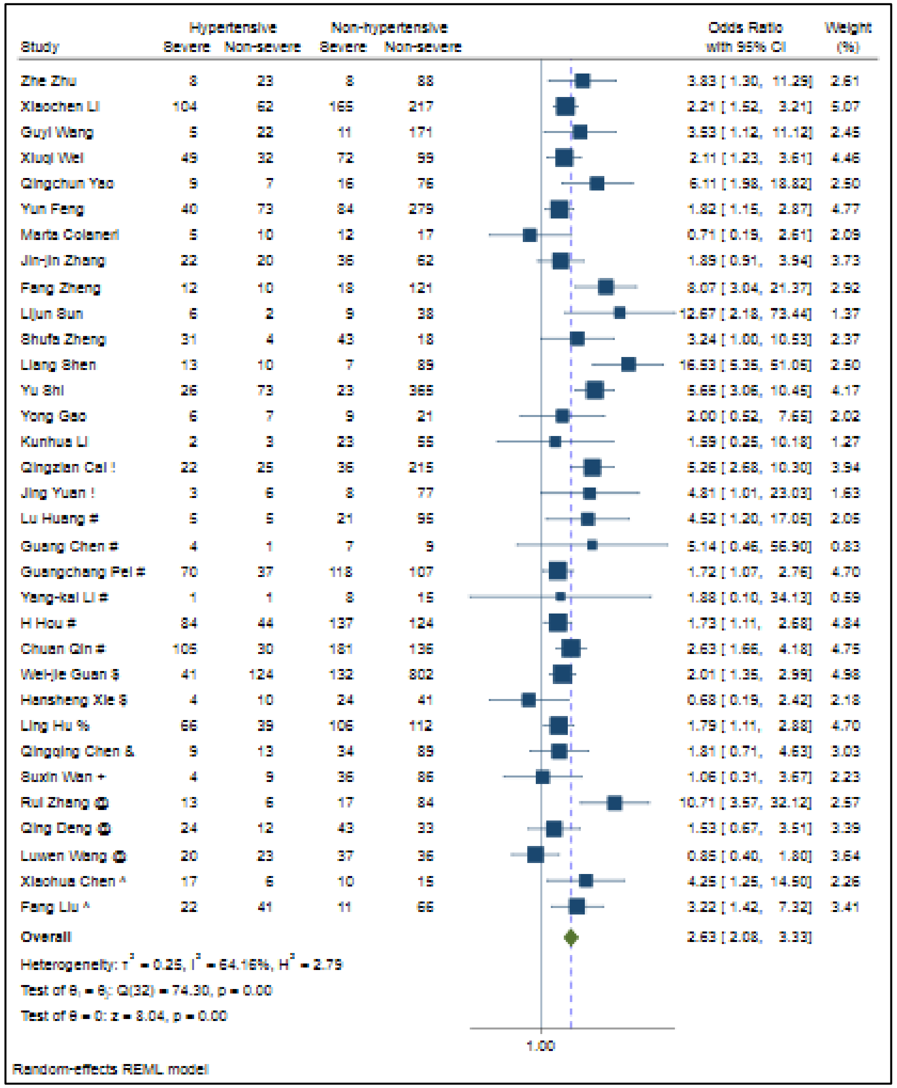

73b

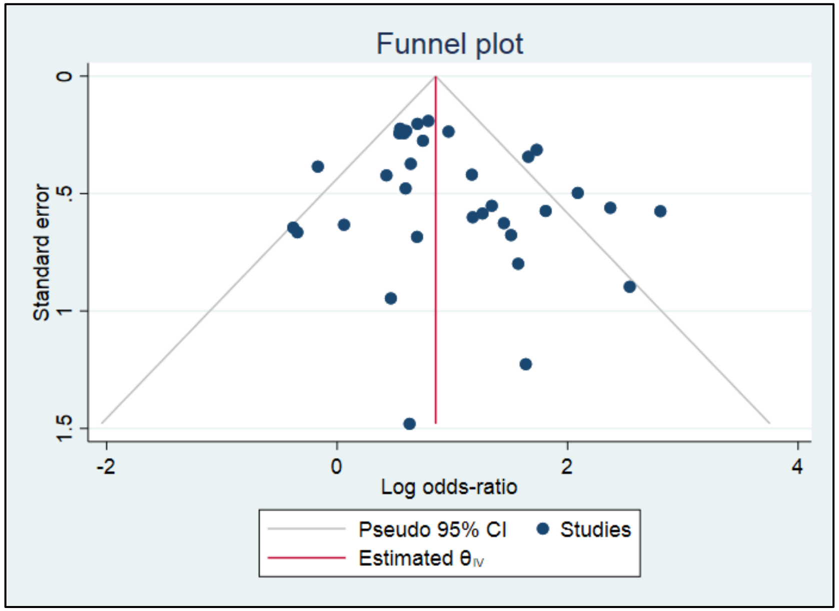

eFigure 74 Pooled Odds ratio of severe disease in patients with Cardiovascular diseases a) Forest plot b) Funnel Plot

74a

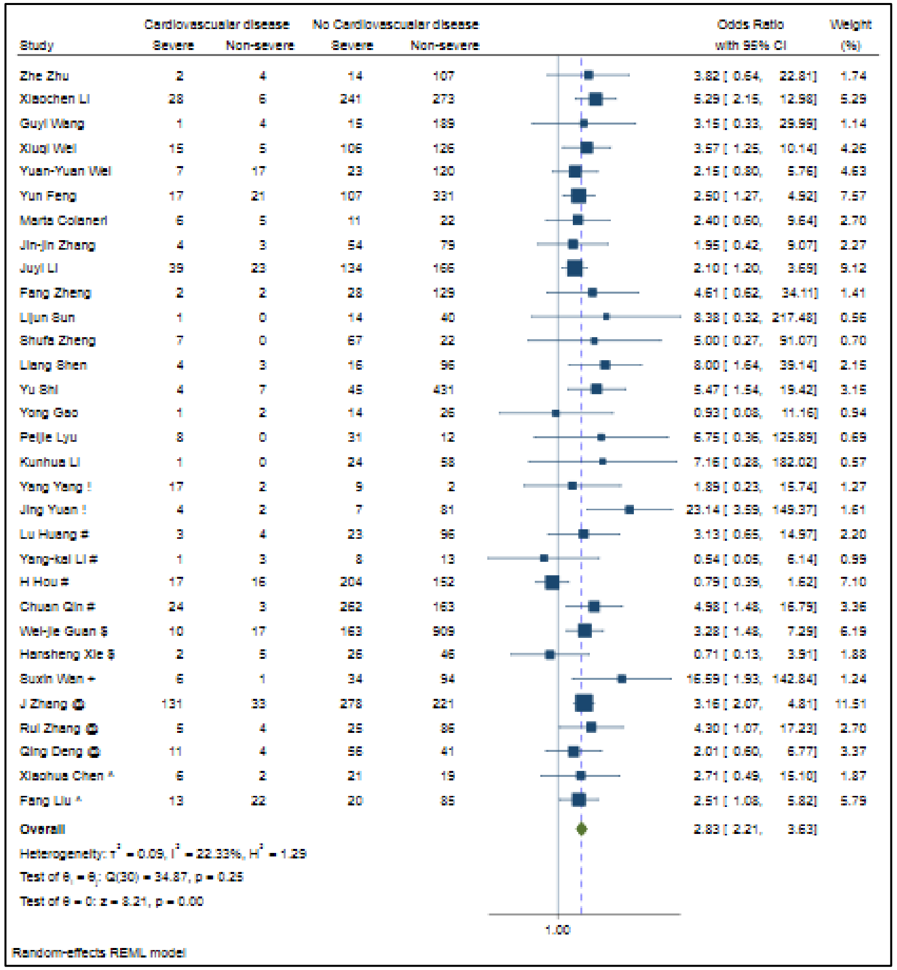

74b

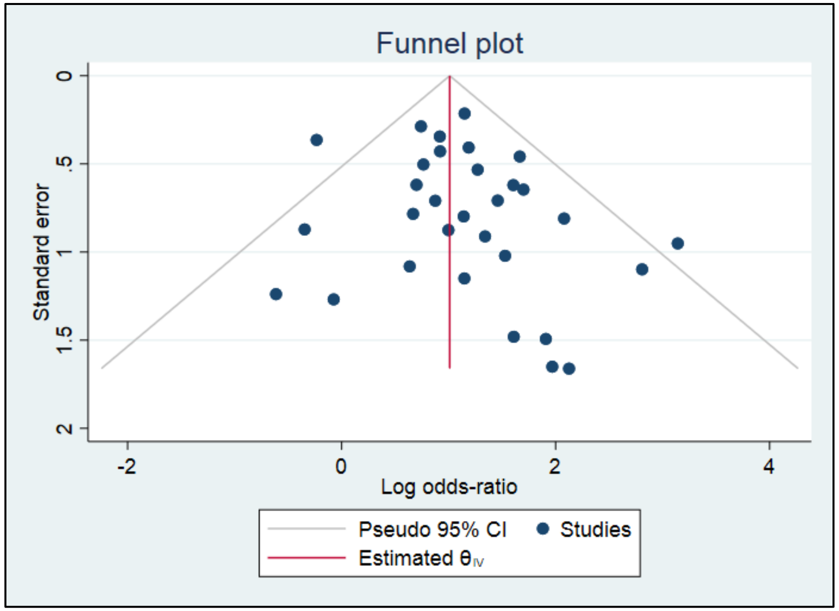

eFigure 75 Pooled Odds ratio of severe disease in patients with Congestive Heart Failure- Forest plot

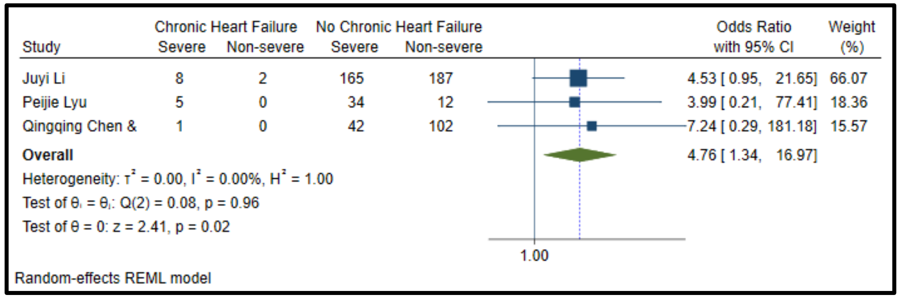

eFigure 76 Pooled Odds ratio of severe disease in patients with Cerebrovascular Disease a) Forest plot b) Funnel Plot

76a

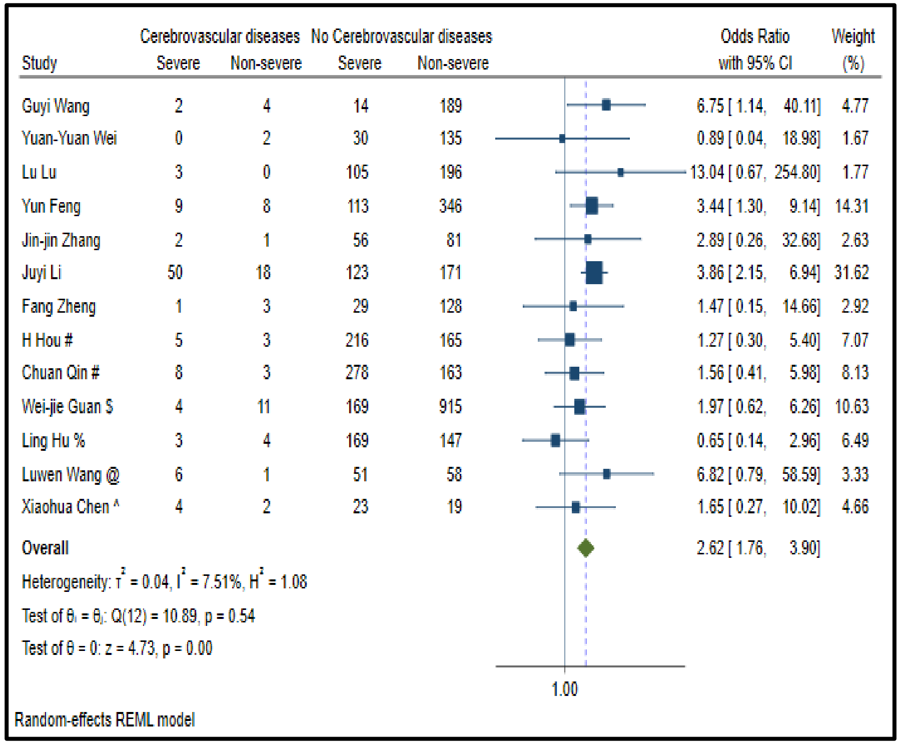

76b

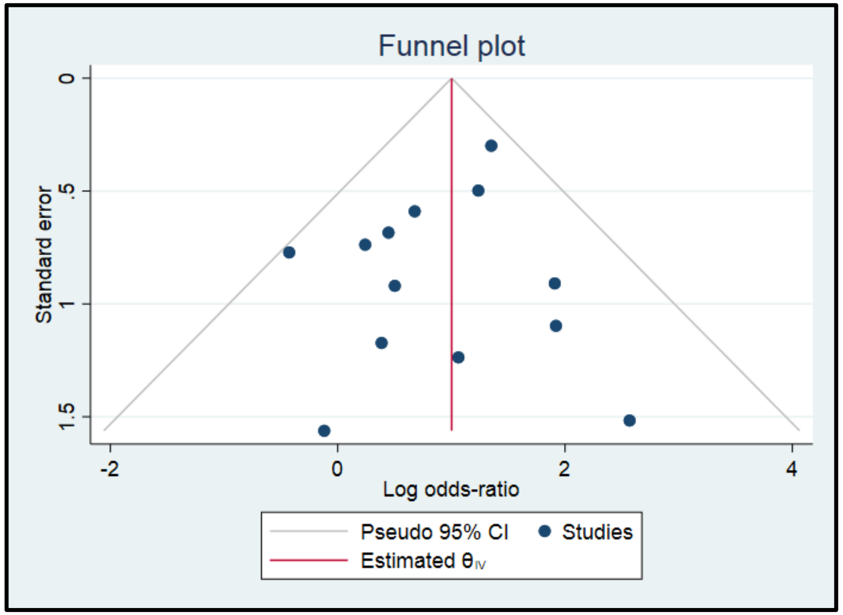

eFigure 77 Pooled Odds ratio of severe disease in patients with Chronic Obstructive pulmonary disease a) Forest plot b) Funnel Plot

77a

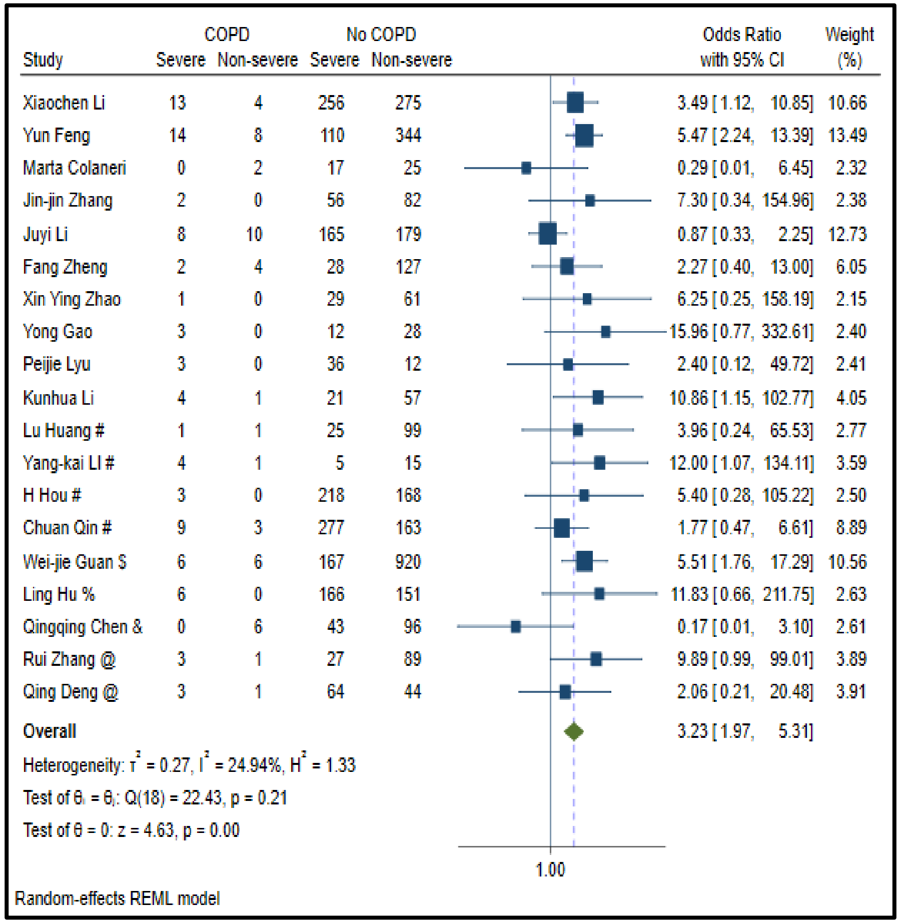

77b

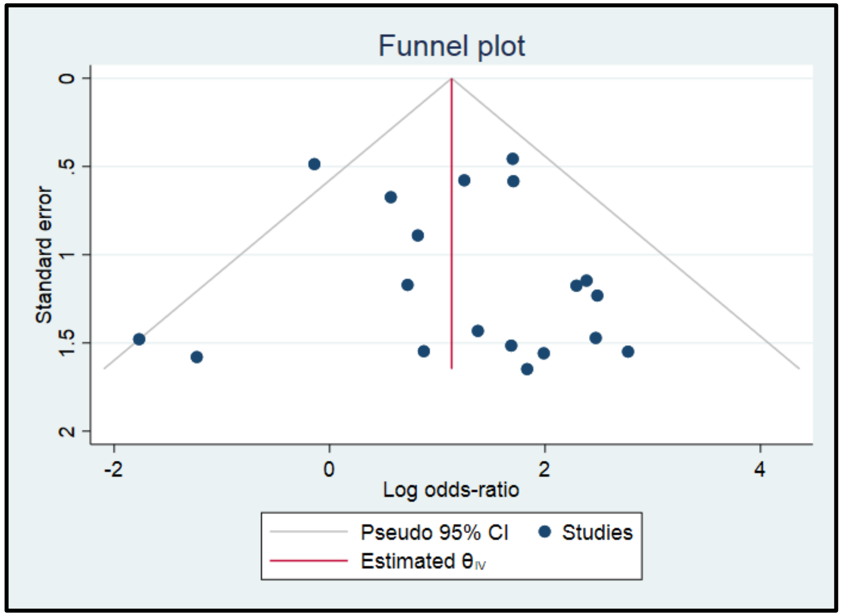

eFigure 78 Pooled Odds ratio of severe disease in patients with chronic kidney disease a) Forest plot b) Funnel Plot

78a

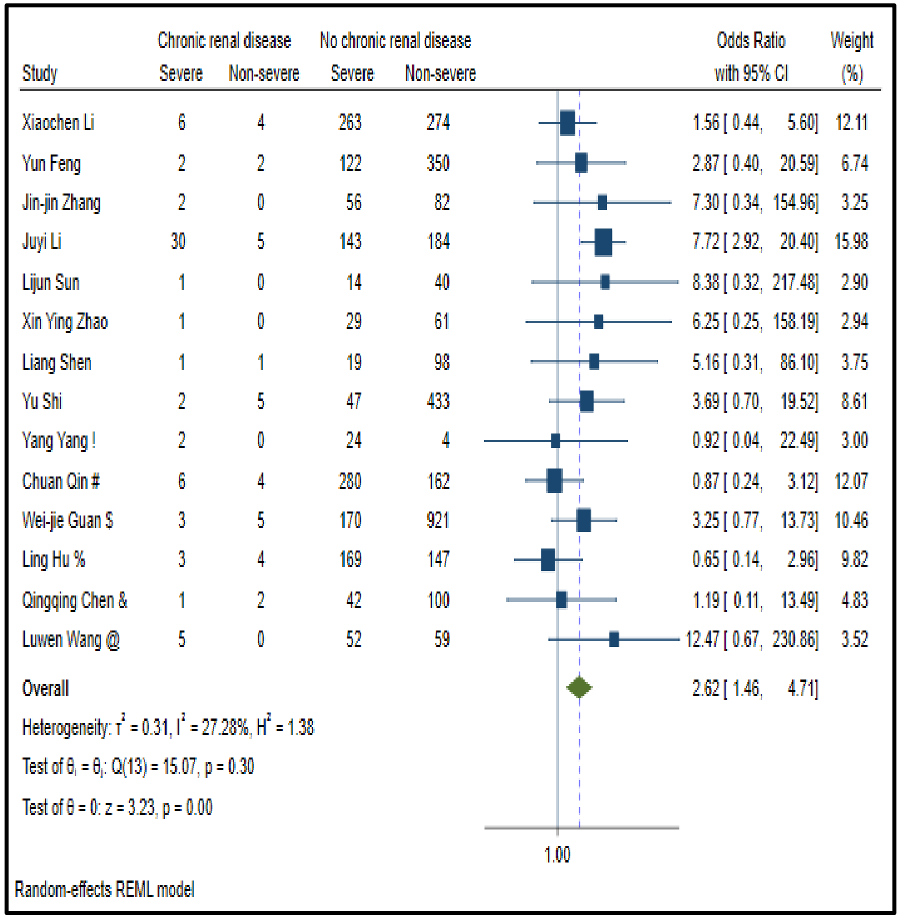

78b

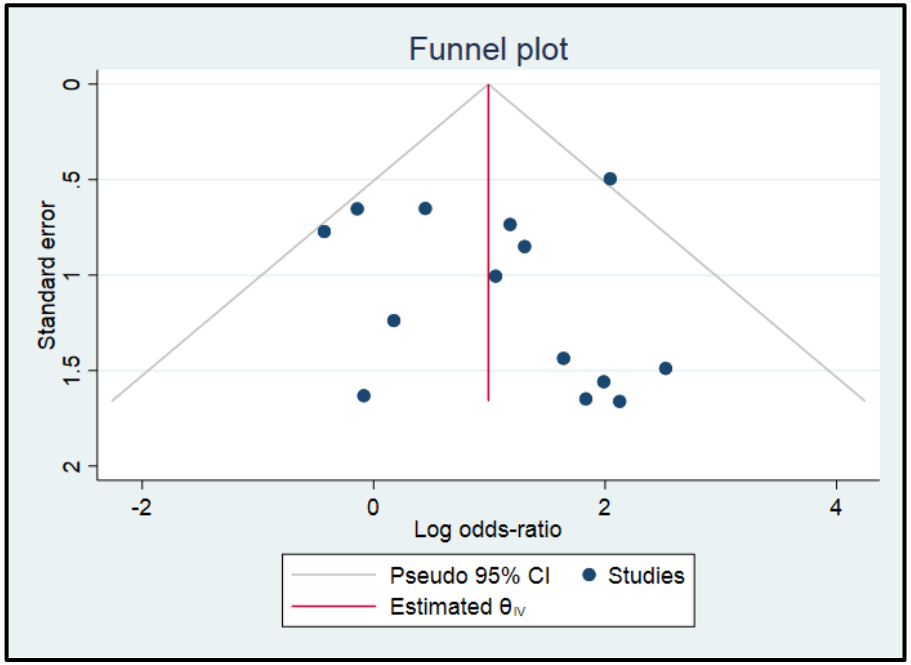

eFigure 79 Pooled Odds ratio of severe disease in patients with liver dysfunction a) Forest plot b) Funnel Plot

79a

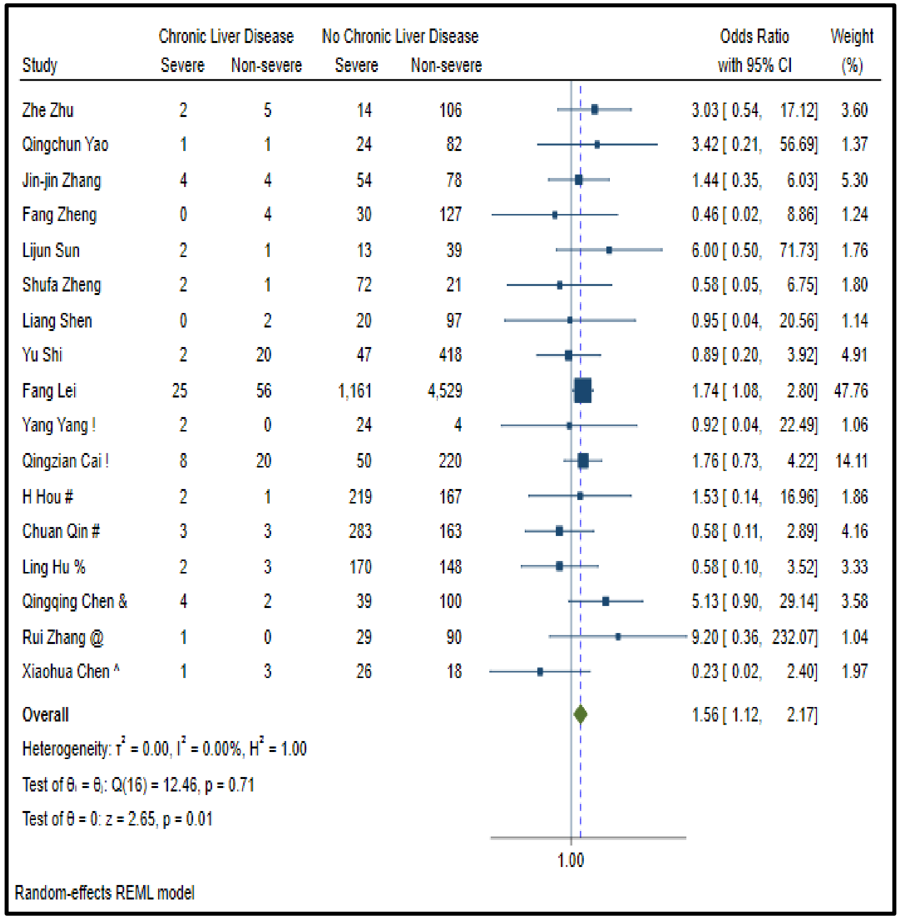

79b

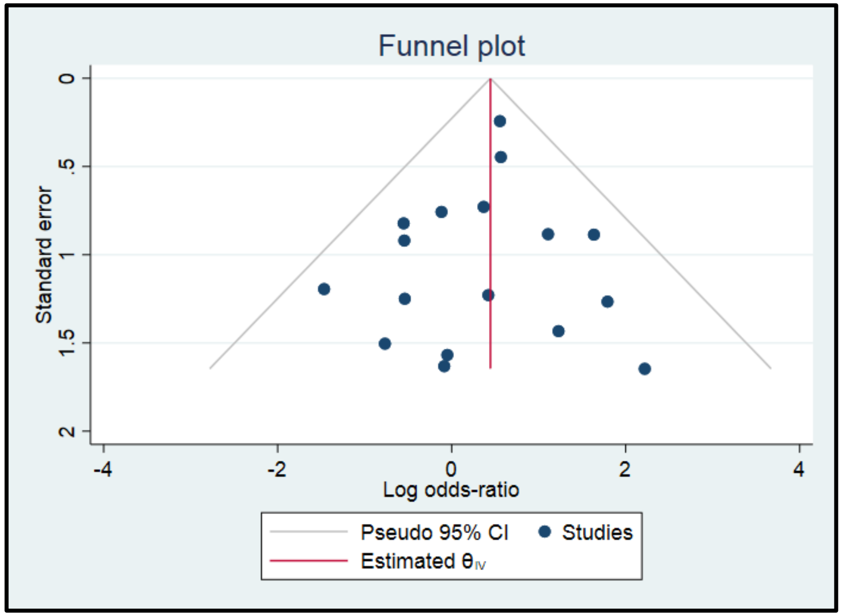

eFigure 80 Pooled Odds ratio of severe disease in patients with Hepatitis B infection - Forest plot

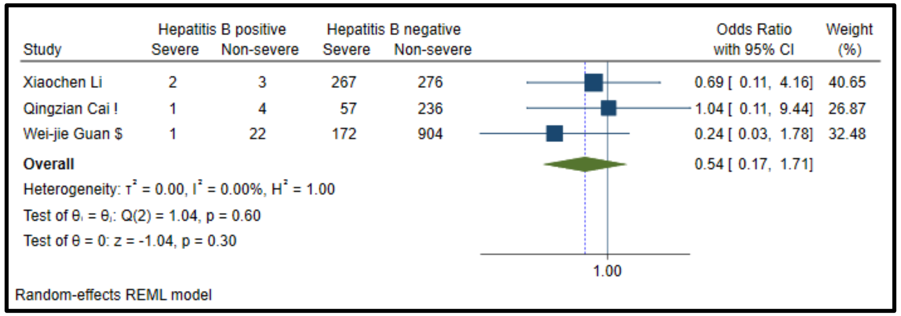

eFigure 81 Pooled Odds ratio of severe disease in patients with malignancy a) Forest plot b) Funnel Plot

81a

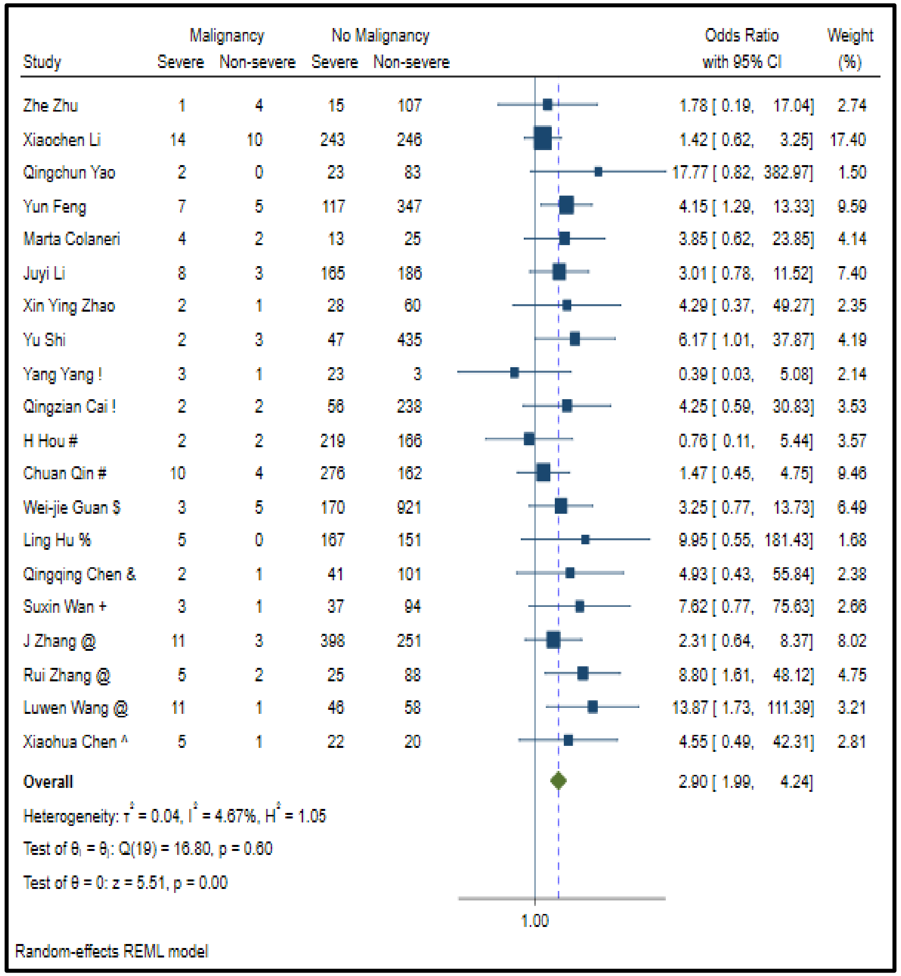

81b

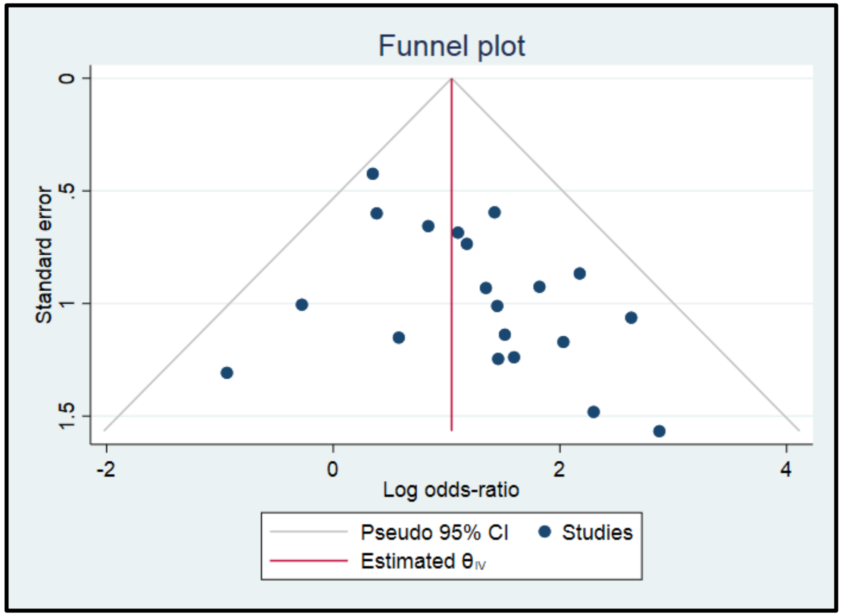

eFigure 82 Pooled Odds ratio of severe disease in patients with HIV- Forest plot

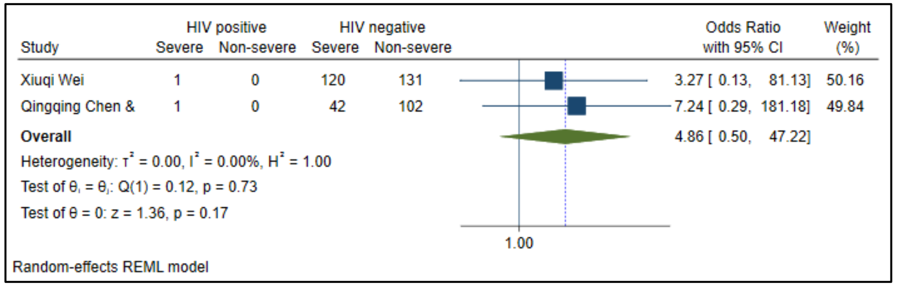

eFigure 83 Pooled Odds ratio of severe disease in patients with immunodeficiency- Forest plot

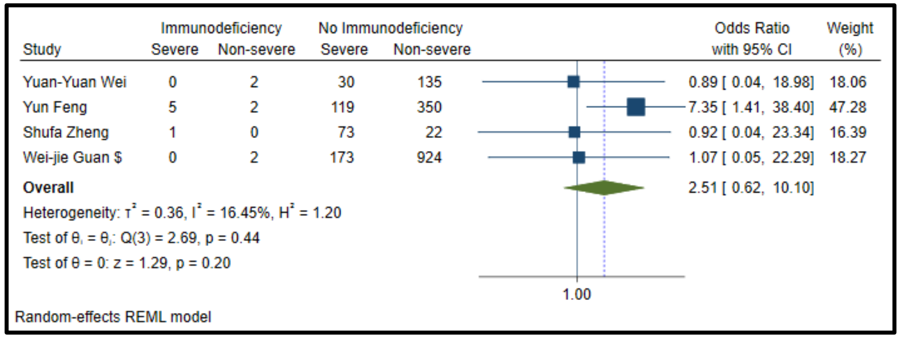

eFigure 84 Pooled Odds ratio of severe disease in patients with other endocrine diseases -Forest plot

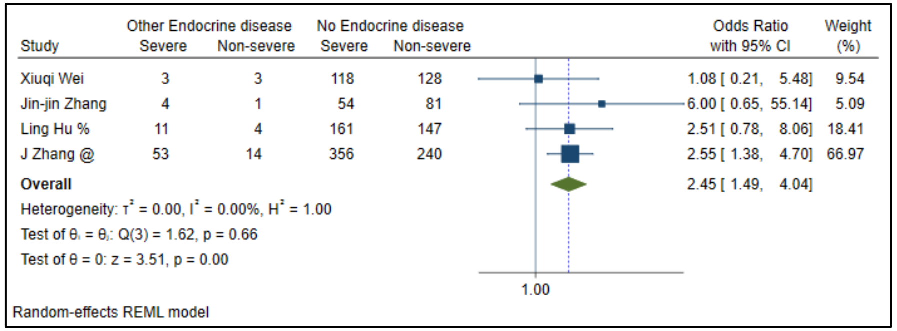

eFigure 85 Pooled Odds ratio of severe disease in patients with fever a) Forest plot b) Funnel Plot

85a

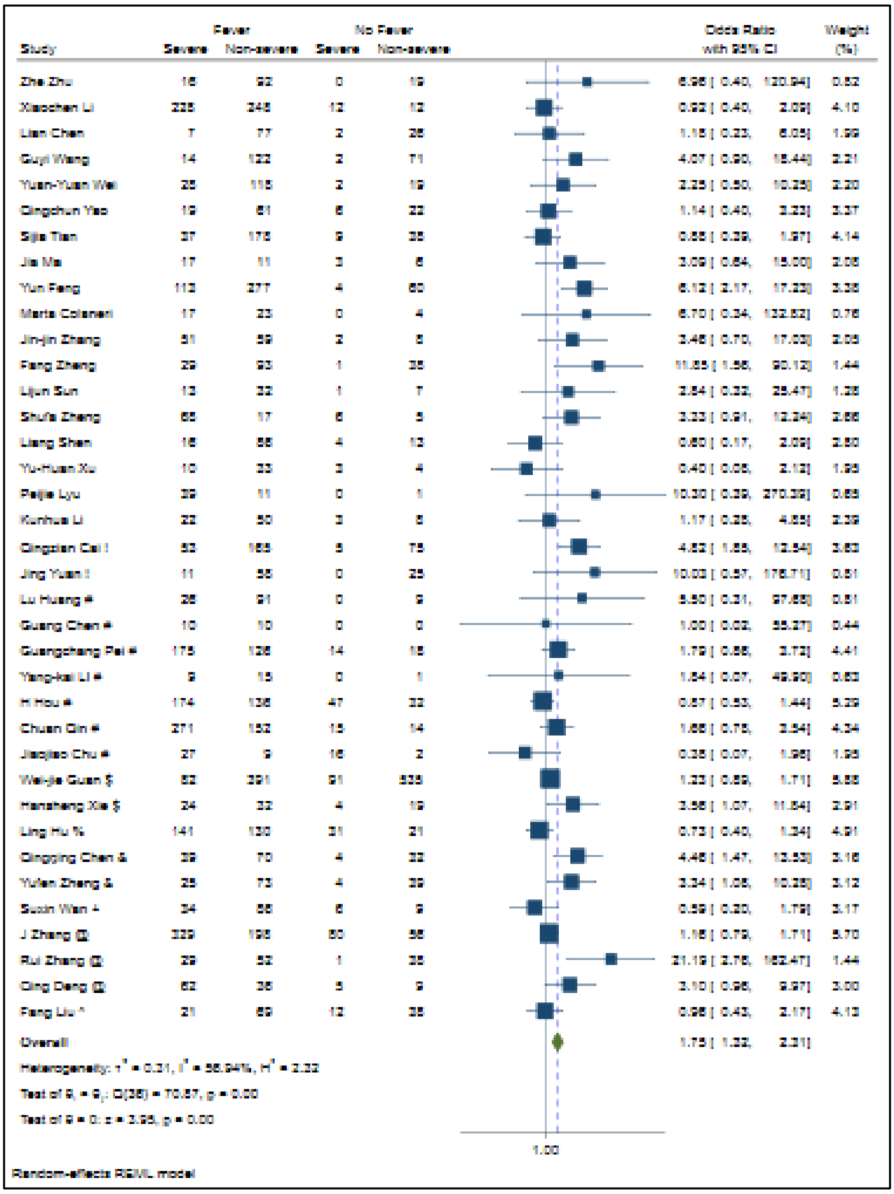

85b

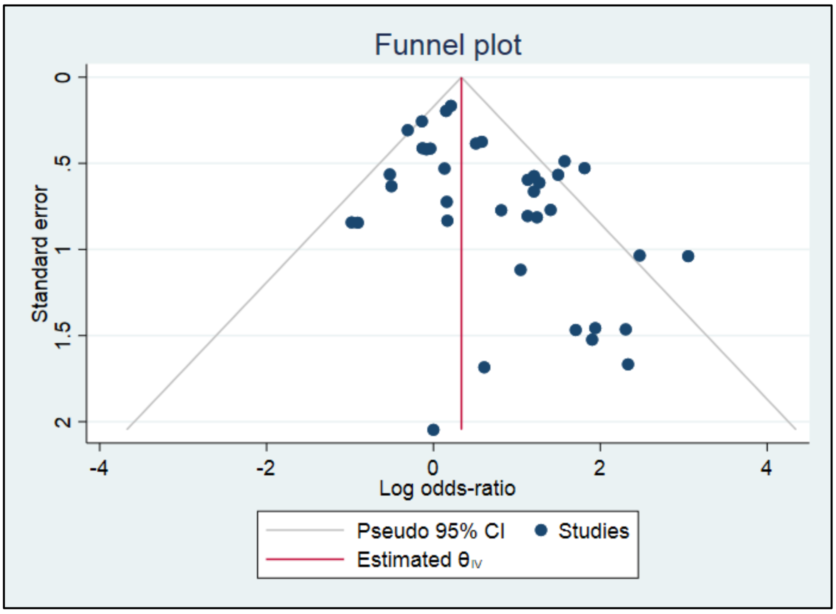

Funnel plot
Standard error
0
.5
1
1.5
2
-4
-2
0
2
4
Log odds-ratio
Pseudo 95% CI
Studies
Estimated θ_IV

eFigure 86 Pooled Odds ratio of severe disease in patients with sore throat a) Forest plot b) Funnel Plot

86a

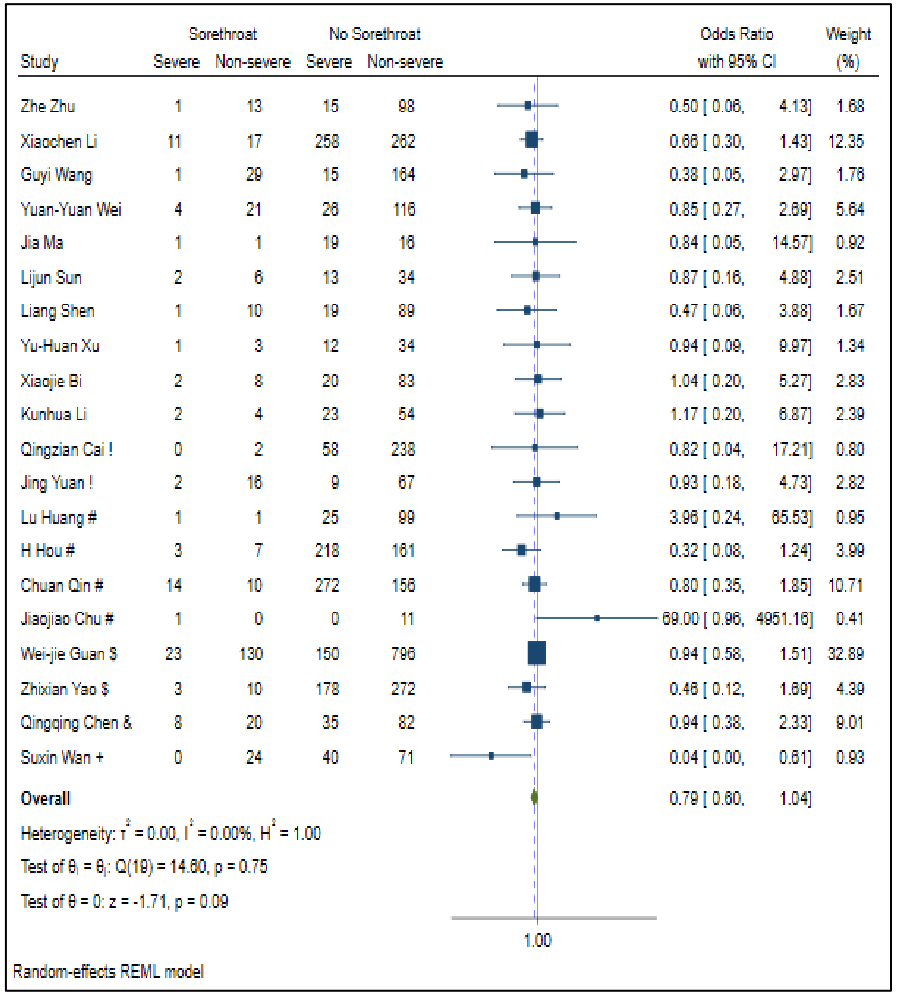

86b

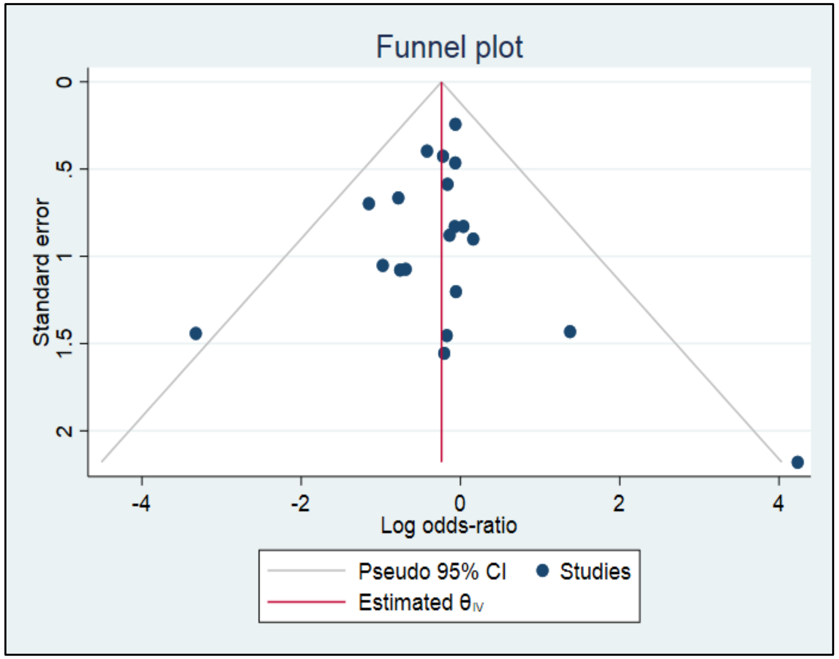

eFigure 87 Pooled Odds ratio of severe disease in patients with cough a) Forest plot b) Funnel Plot

87a

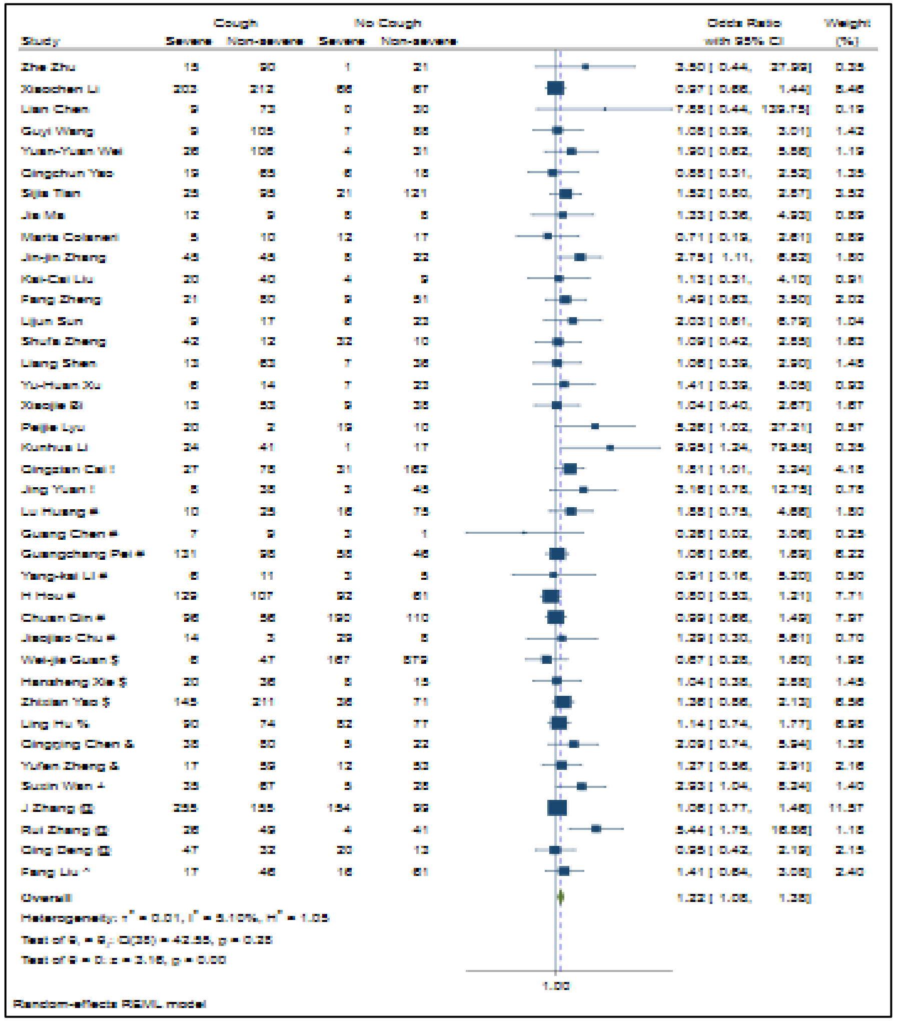

87b

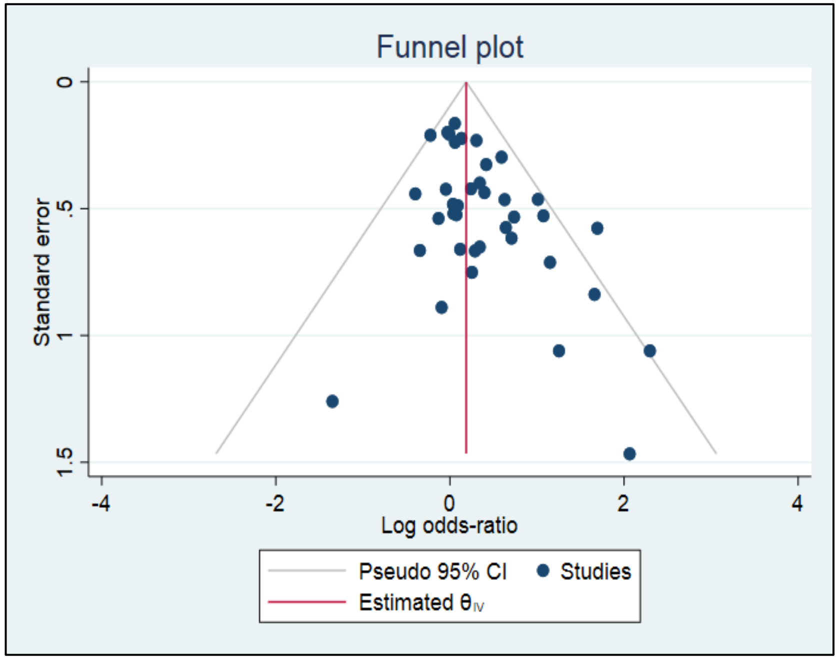

eFigure 88 Pooled Odds ratio of severe disease in patients with expectoration a) Forest plot b) Funnel Plot

88a

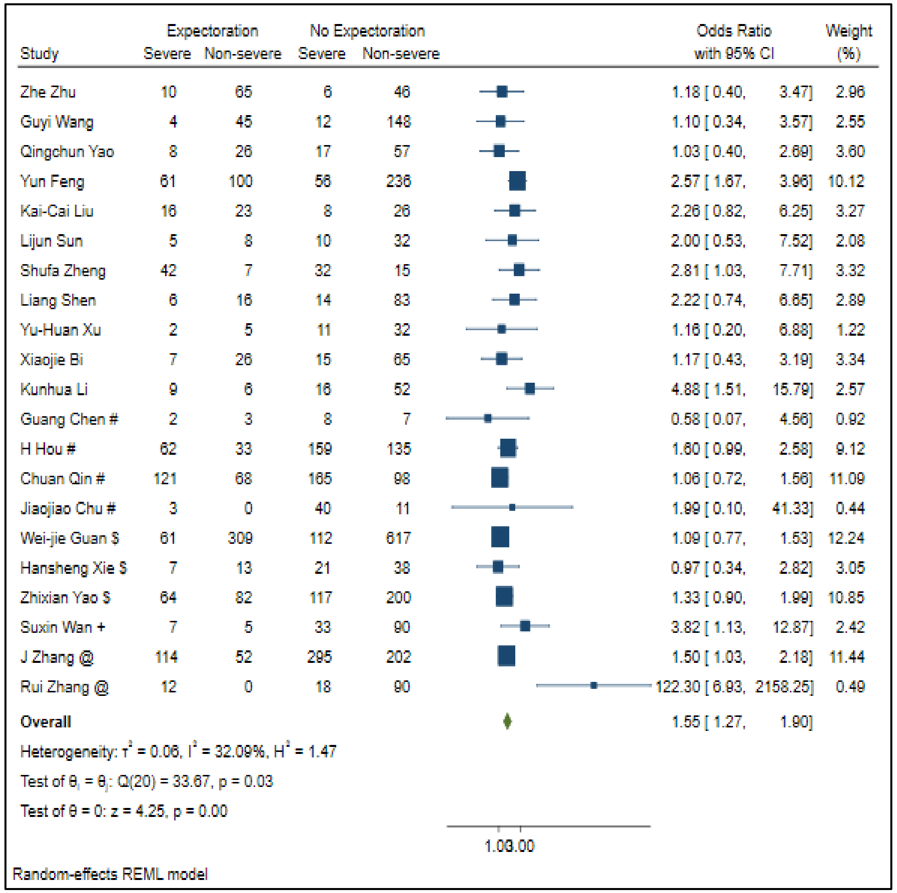

88b

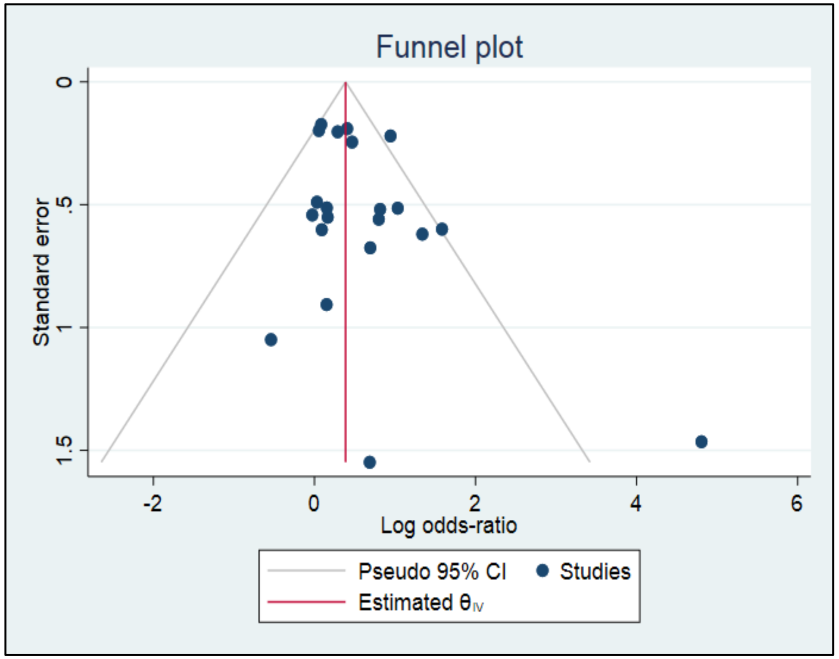

eFigure 89 Pooled Odds ratio of severe disease in patients with vomiting- Forest plot

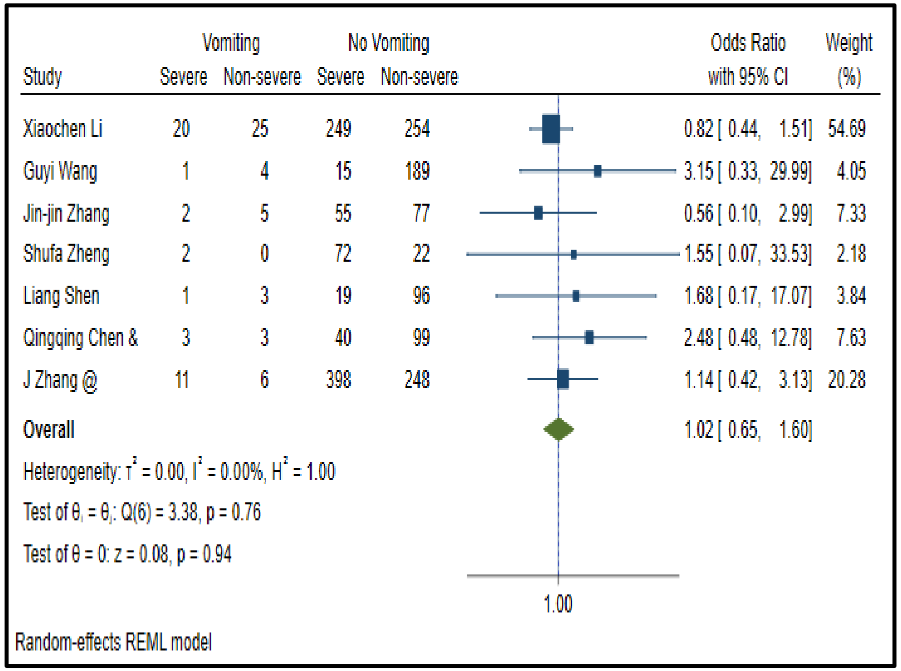

eFigure 90 Pooled Odds ratio of severe disease in patients with diarrhea a) Forest plot b) Funnel Plot

90a

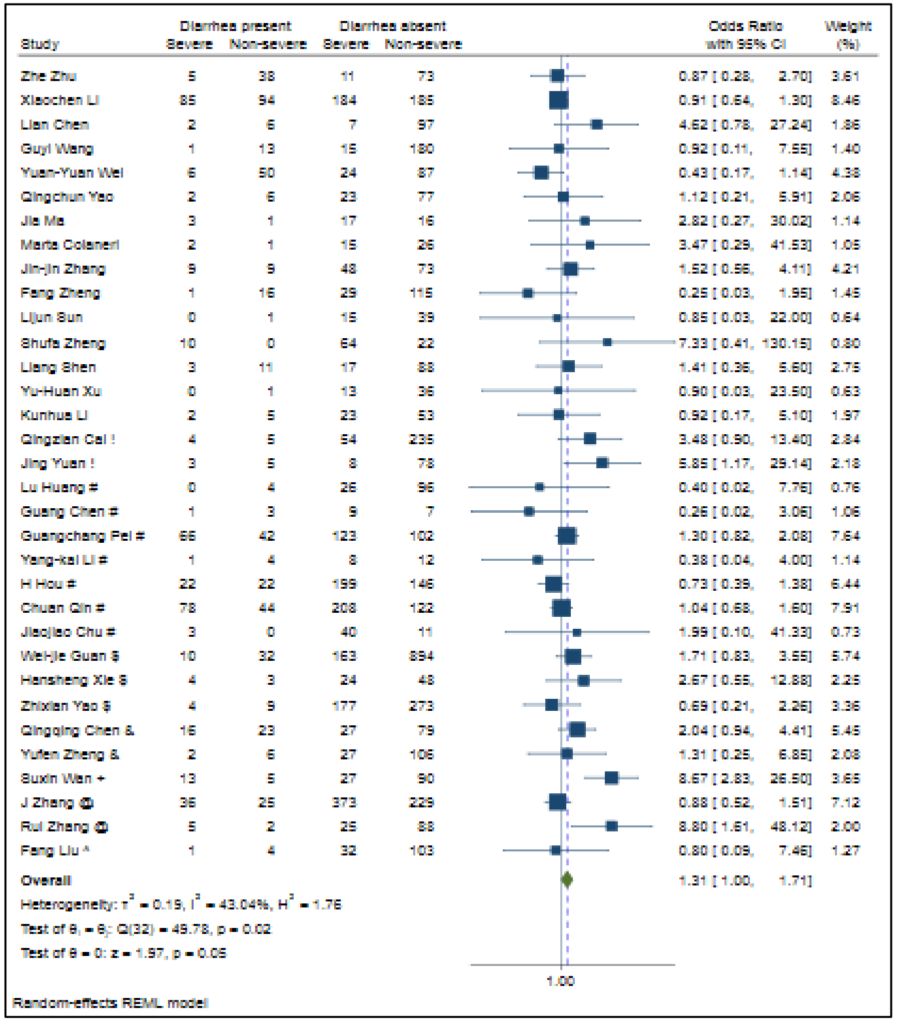

90b

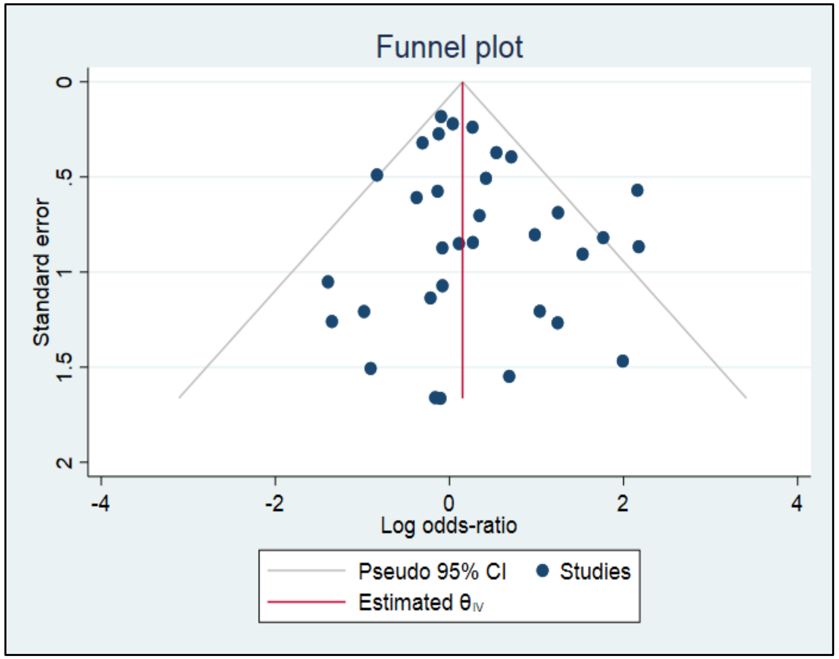

eFigure 91 Pooled Odds ratio of severe disease in patients with nausea a) Forest plot b) Funnel Plot

91a

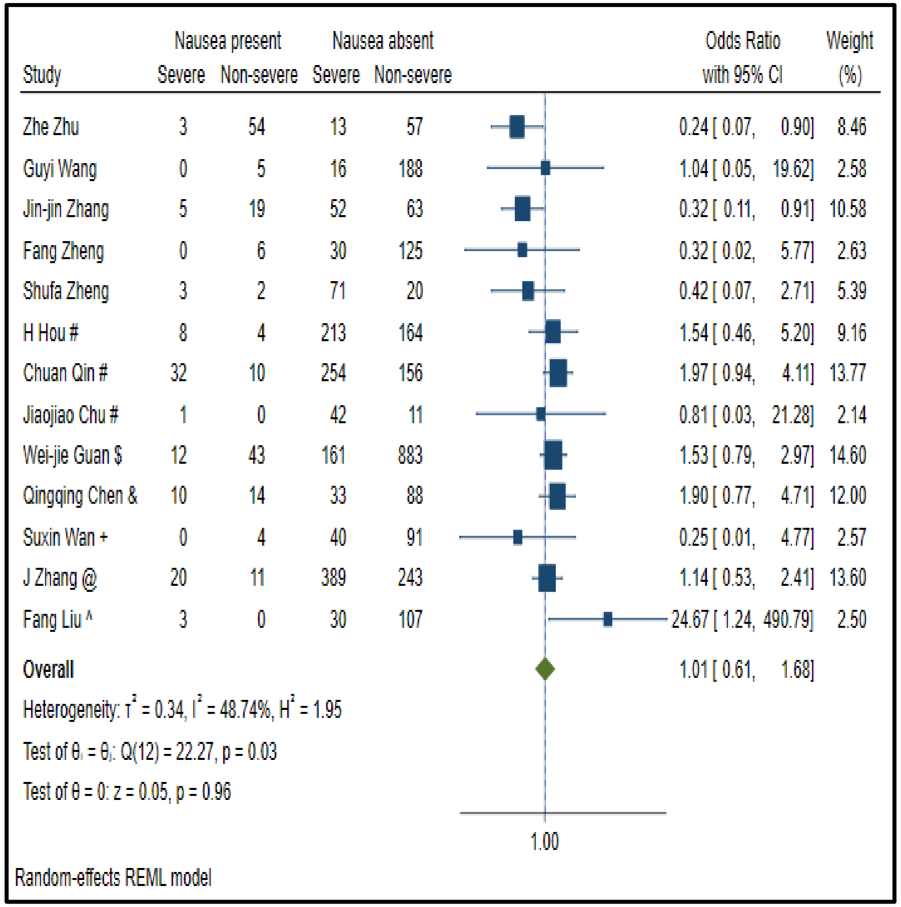

91b

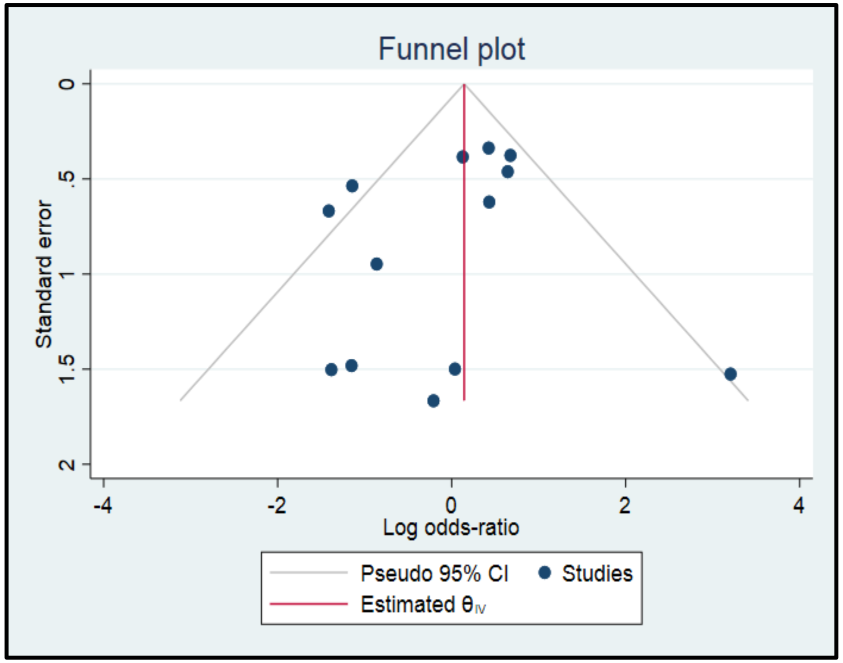

eFigure 92 Pooled Odds ratio of severe disease in patients with myalgia a) Forest plot b) Funnel Plot

92a

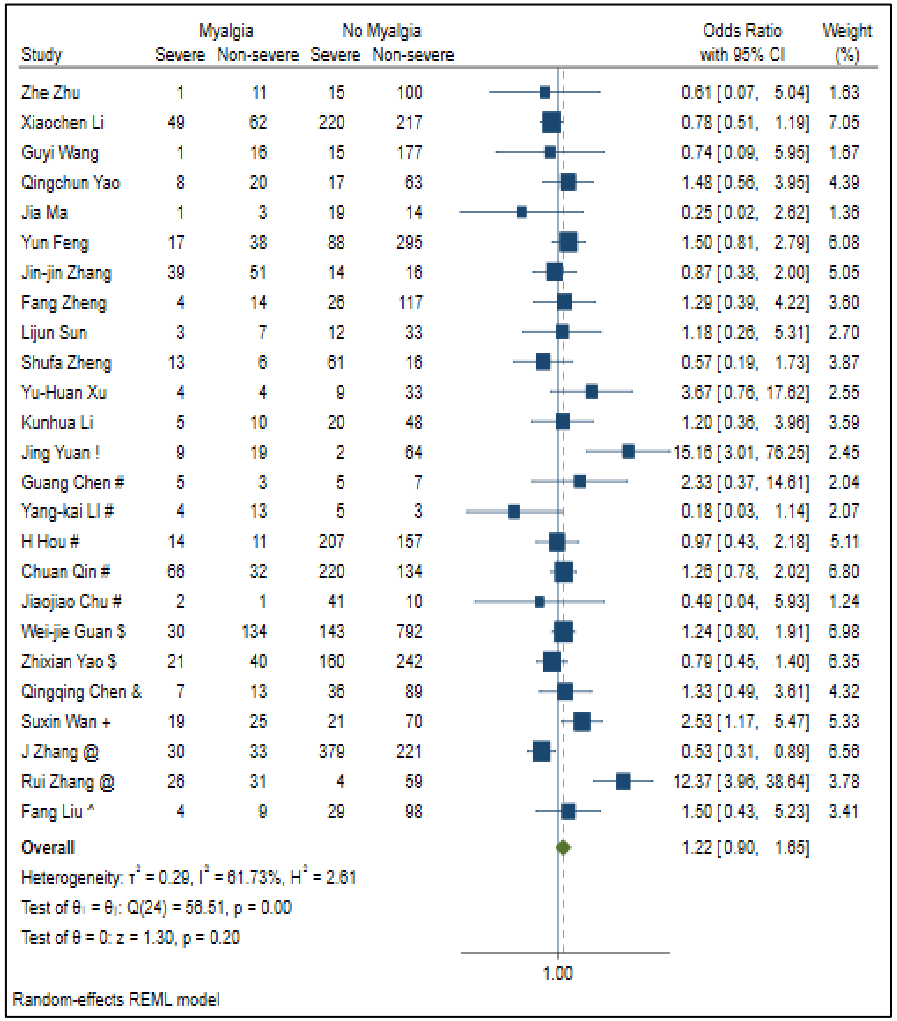

92b

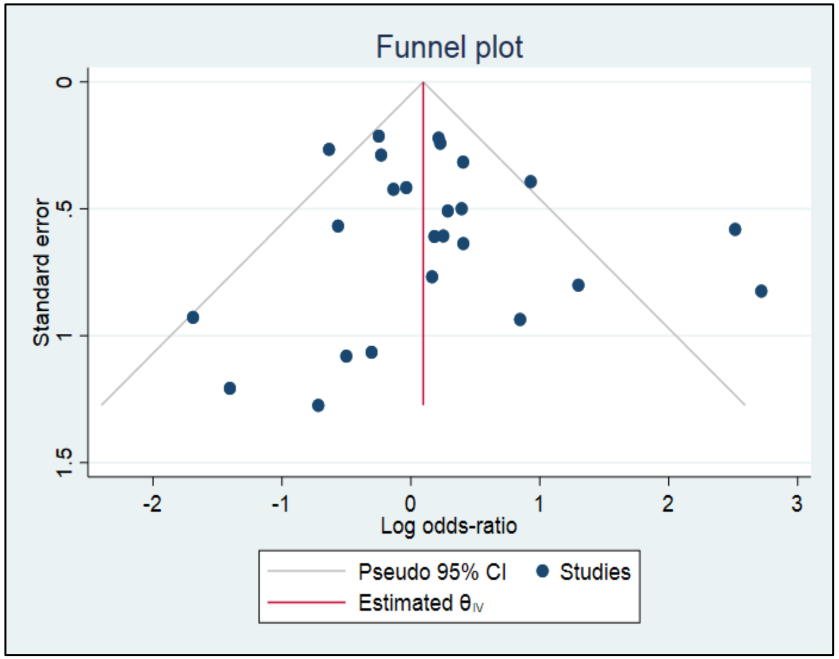

eFigure 93 Pooled Odds ratio of severe disease in patients with headache a) Forest plot b) Funnel Plot

93a

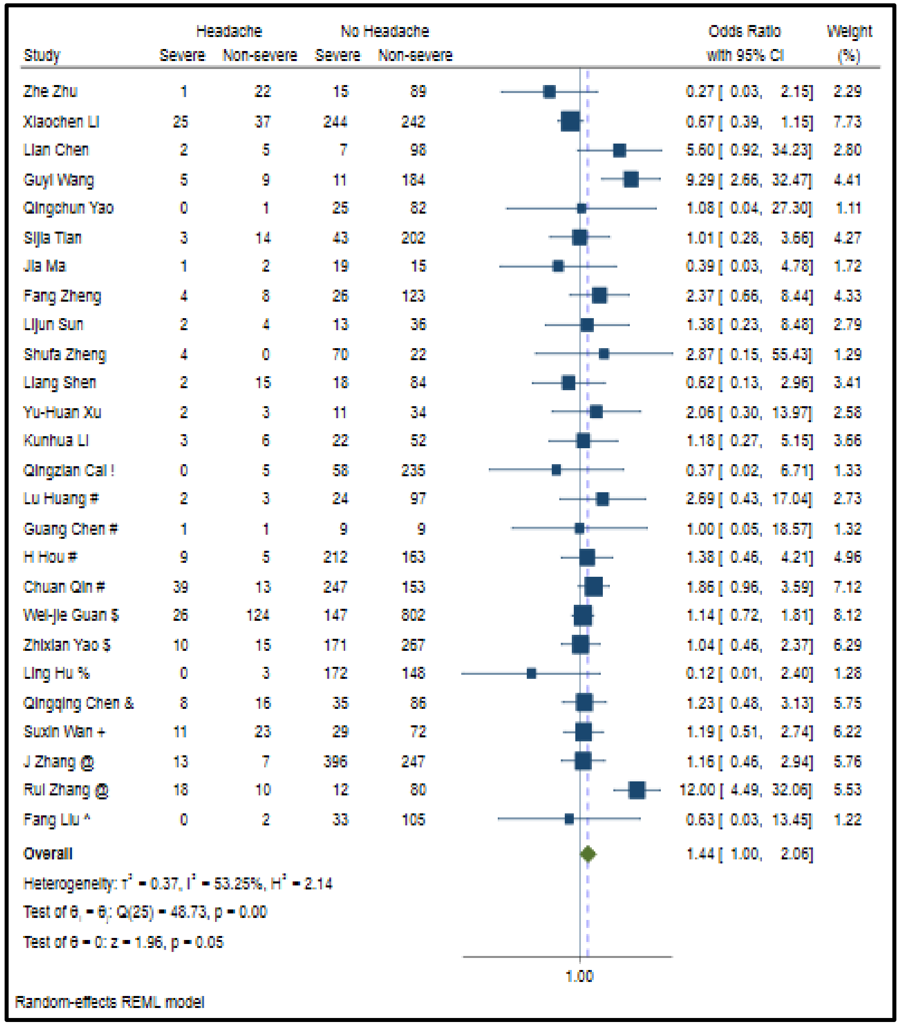

93b

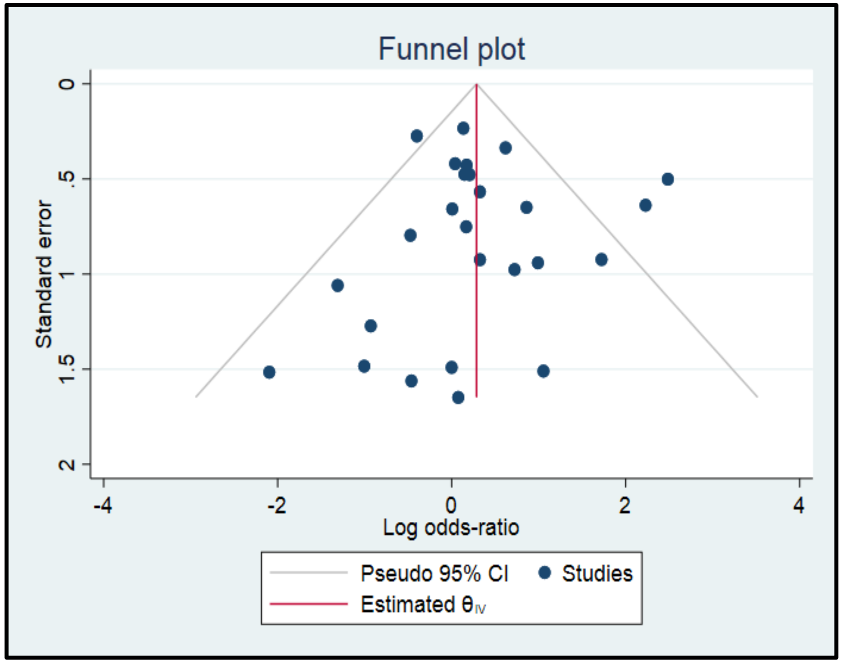

eFigure 94 Pooled Odds ratio of severe disease in patients with anorexia a) Forest plot b) Funnel Plot

94a

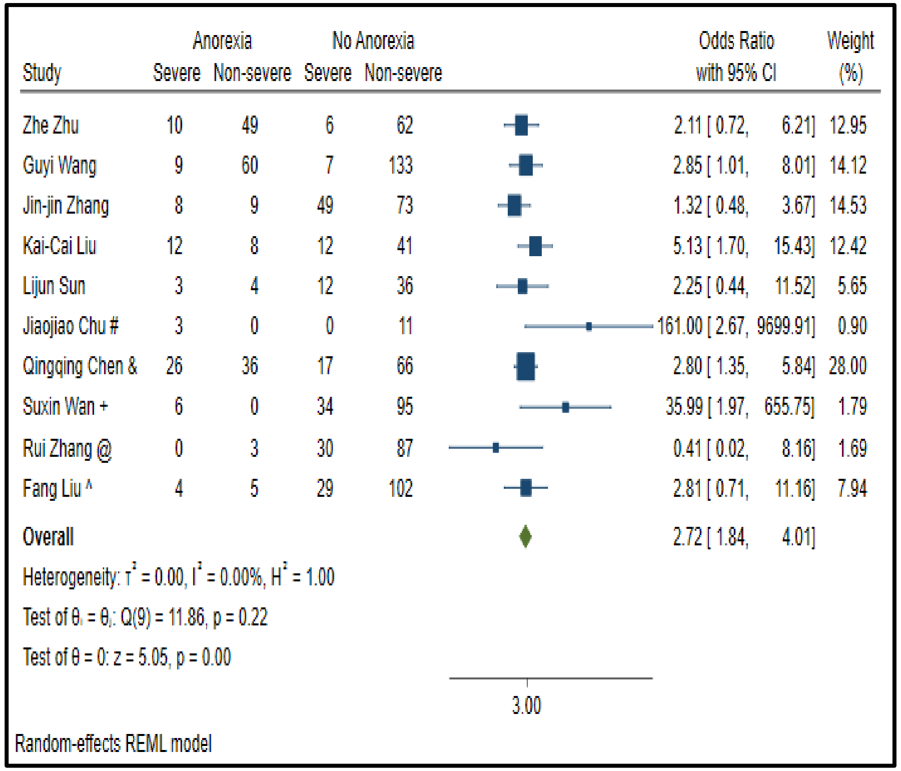

94b

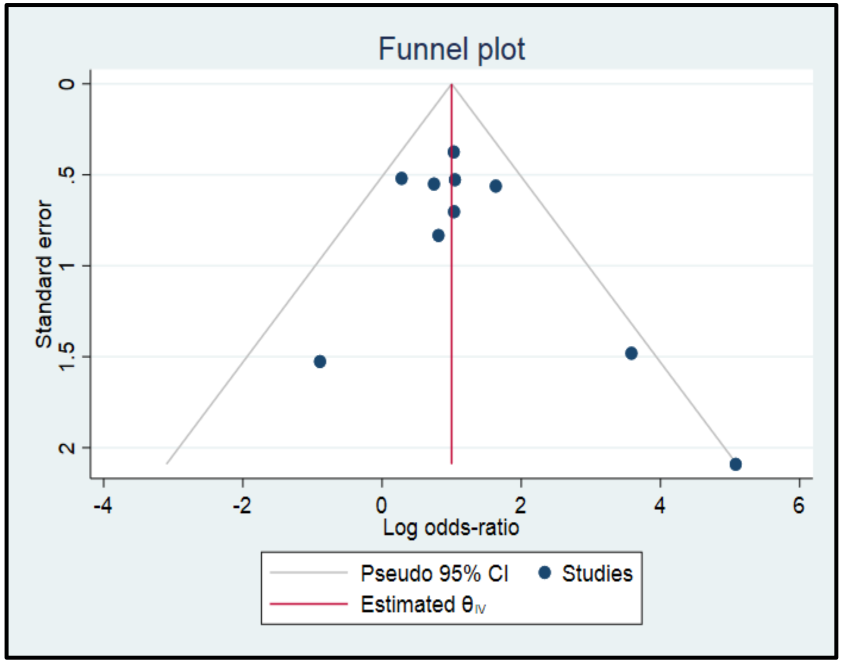

eFigure 95 Pooled Odds ratio of severe disease in patients with chest pain a) Forest plot b) Funnel Plot

95a

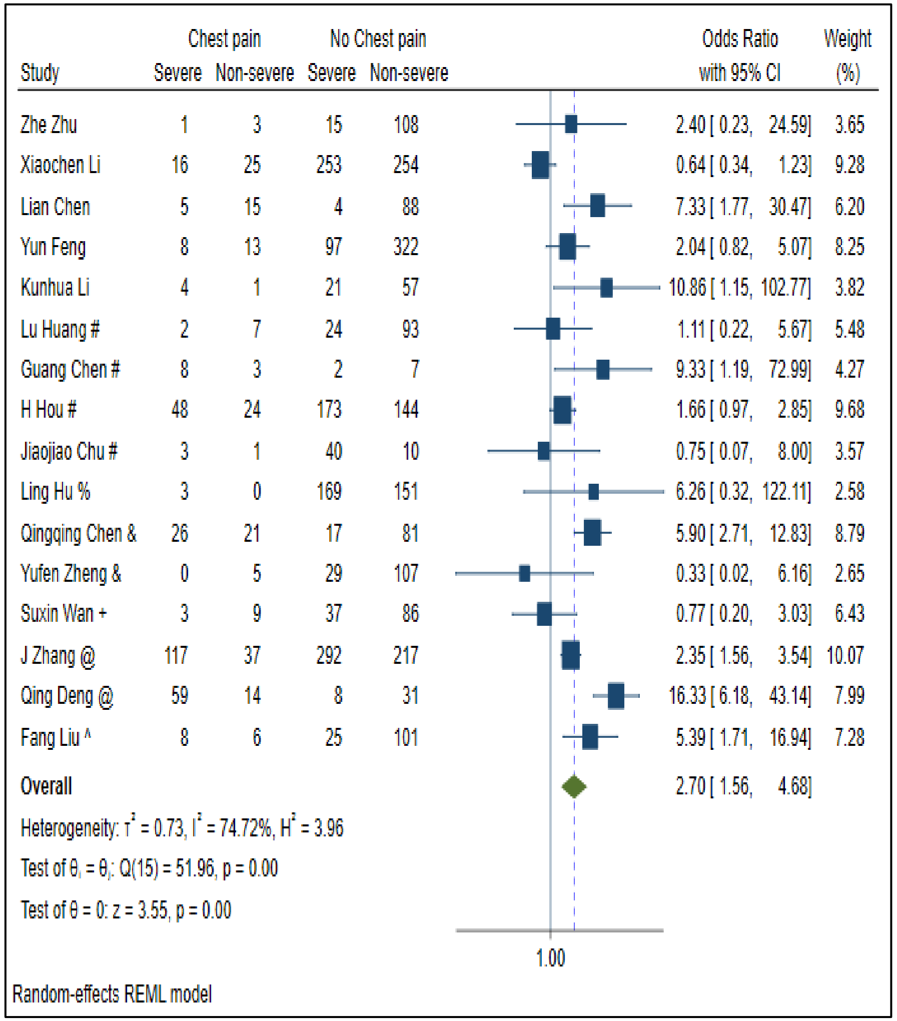

95b

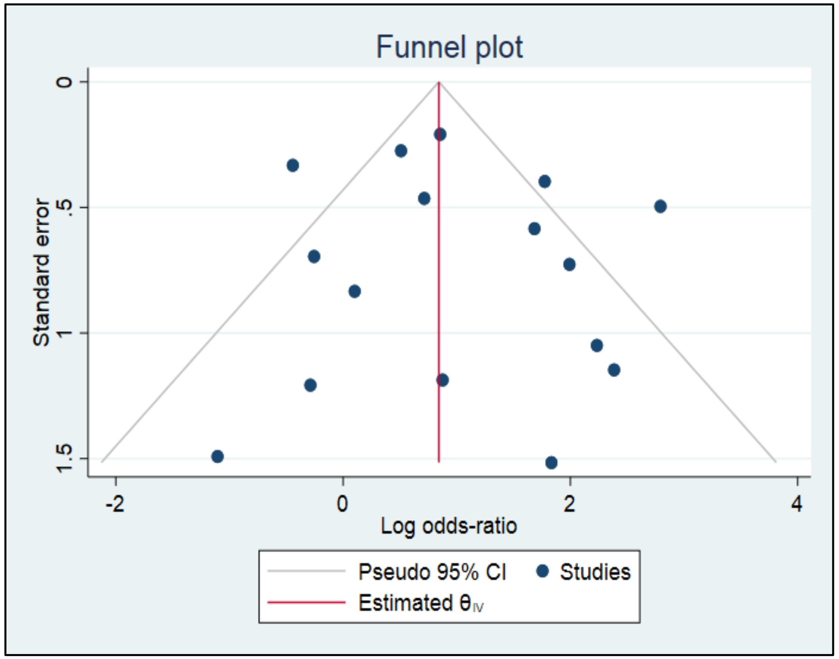

eFigure 96 Pooled Odds ratio of severe disease in patients with dyspnea a) Forest plot b) Funnel Plot

96a

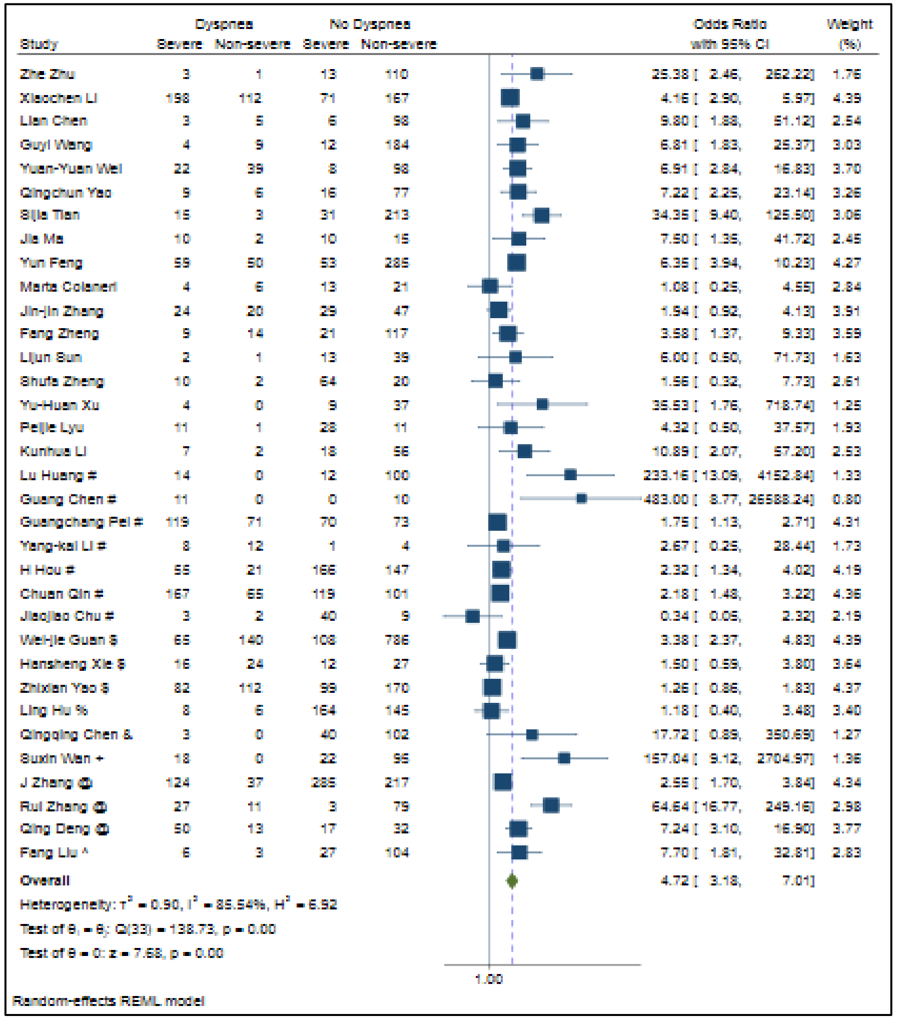

96b

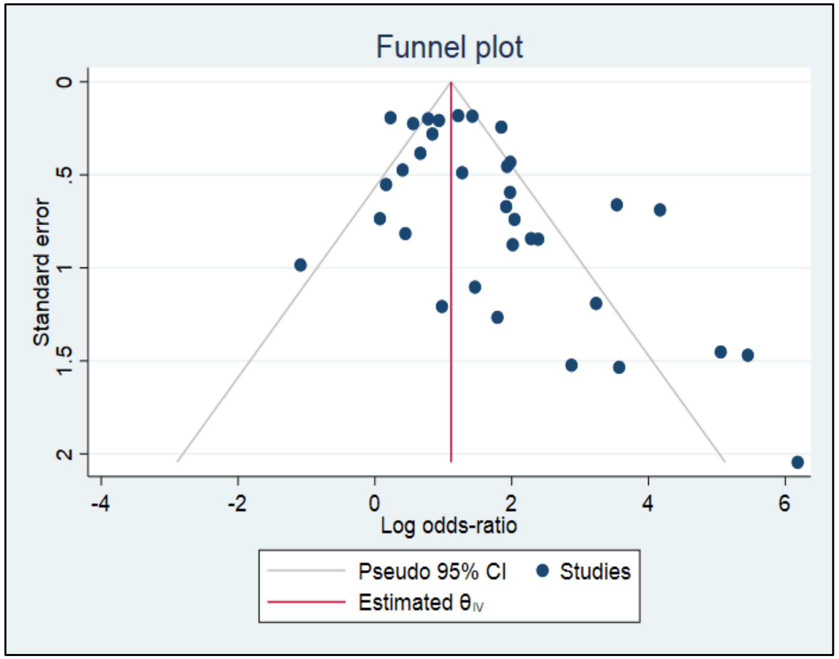

eFigure 97 Pooled Odds ratio of severe disease in patients with hemoptysis - Forest plot

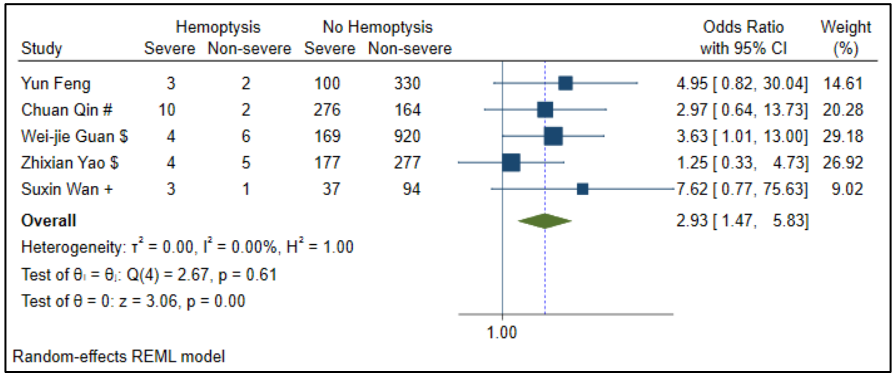

eFigure 98 Pooled Odds ratio of severe disease in patients with abdominal pain - Forest plot

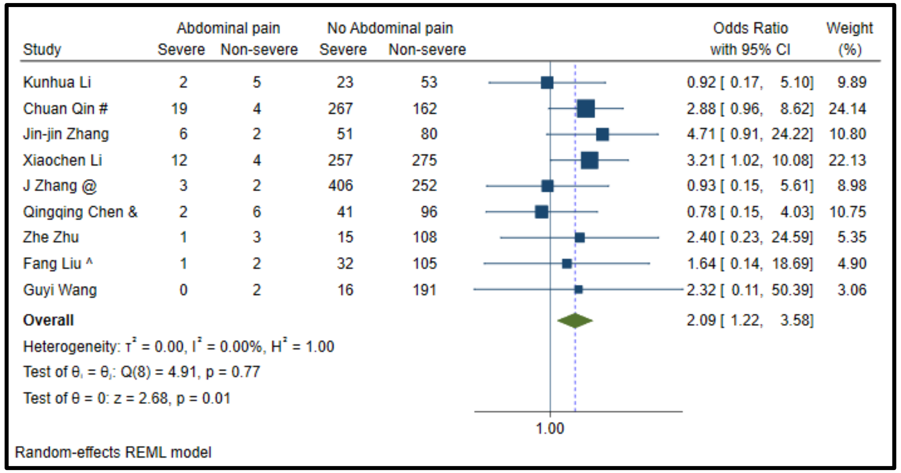

eFigure 99 Pooled Odds ratio of severe disease in patients with palpitations - Forest plot

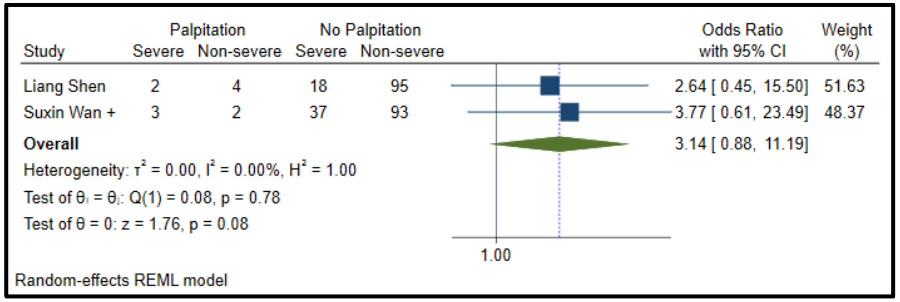

eFigure 100 Pooled Odds ratio of severe disease in patients with rhinorrhea - Forest plot

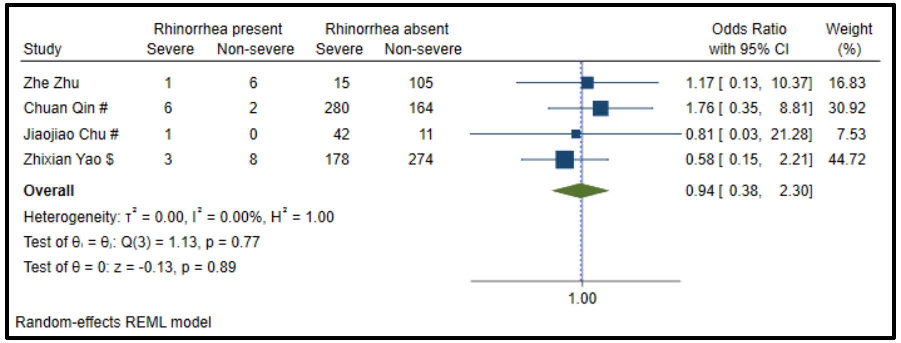

eFigure 101 Pooled Odds ratio of severe disease in patients with fever a) Forest plot b) Funnel Plot

101a

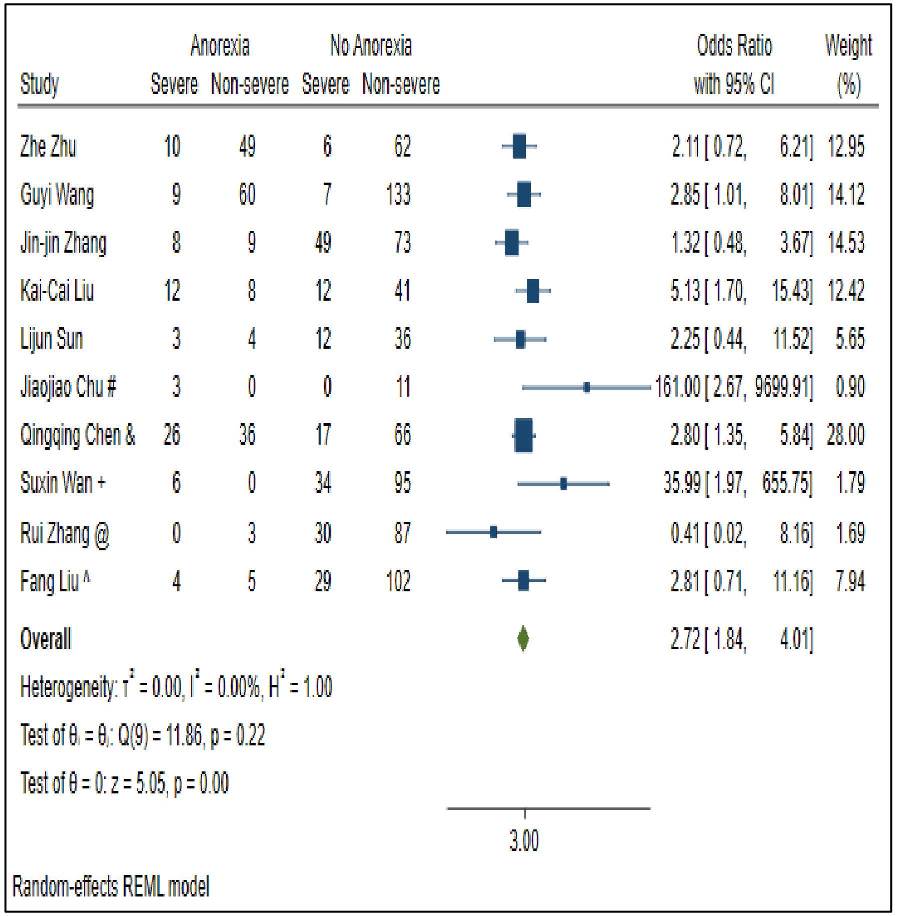

101b

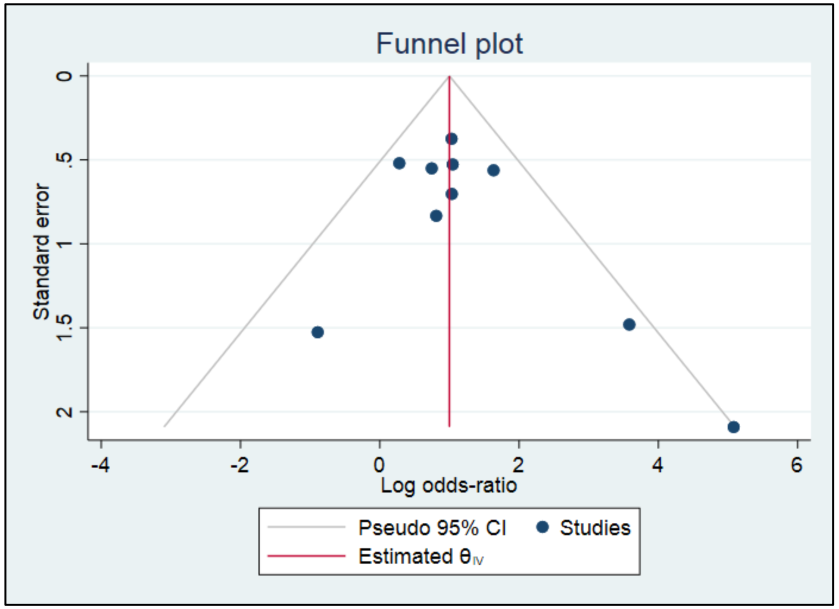

eFigure 102. Pooled Odds of severity in patients with Decreased TLC- Forest plot

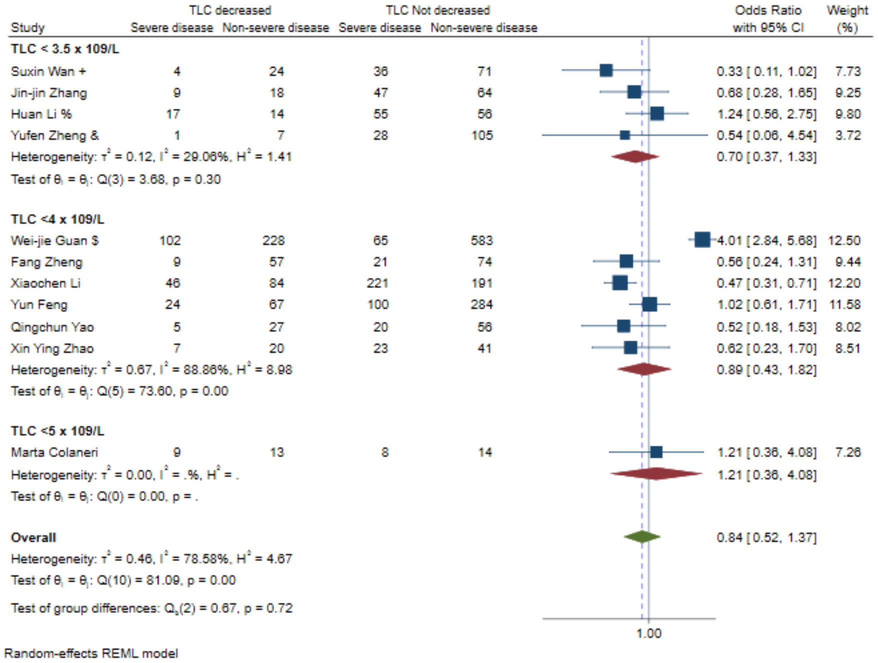

eFigure 103. Pooled Odds of severity in patients with Increased TLC- Forest plot

Study	TLC increased: Severe disease	TLC increased: Non-severe disease	TLC not increased: Severe disease	TLC not increased: Non-severe disease	Odds Ratio with 95% CI	Weight (%)
**TLC > 10 x 109/L**						
Fang Zheng	0	3	30	128	0.60 [0.03, 11.96]	2.23
Xiaochen Li	55	8	212	267	8.66 [4.04, 18.57]	13.70
Yun Feng	26	23	98	328	3.78 [2.07, 6.93]	15.81
Qingchun Yao	11	1	14	82	64.43 [7.70, 538.98]	4.01
Xin Ying Zhao	5	6	25	55	1.83 [0.51, 6.58]	8.33
Ling Hu %	18	5	147	135	3.31 [1.19, 9.15]	10.72
Heterogeneity: $\tau^2 = 0.49$, $I^2 = 63.09\%$, $H^2 = 2.71$					4.65 [2.17, 9.98]	
Test of $\theta_i = \theta_j$: Q(5) = 13.14, p = 0.02						
**TLC > 11 x 109/L**						
Wei-jie Guan $	19	39	148	772	2.54 [1.43, 4.52]	16.20
Heterogeneity: $\tau^2 = 0.00$, $I^2 = .\%$, $H^2 = .$					2.54 [1.43, 4.52]	
Test of $\theta_i = \theta_j$: Q(0) = 0.00, p = .						
**TLC > 9.5 x 109/L**						
Suxin Wan +	3	6	37	89	1.20 [0.29, 5.07]	7.16
Jin-jin Zhang	13	4	43	78	5.90 [1.81, 19.20]	9.14
Huan Li %	15	2	57	58	7.63 [1.67, 34.89]	6.65
Yufen Zheng &	2	7	27	105	1.11 [0.22, 5.66]	6.04
Heterogeneity: $\tau^2 = 0.47$, $I^2 = 47.12\%$, $H^2 = 1.89$					2.94 [1.10, 7.85]	
Test of $\theta_i = \theta_j$: Q(3) = 5.68, p = 0.13						
**Overall**					3.70 [2.31, 5.93]	
Heterogeneity: $\tau^2 = 0.27$, $I^2 = 49.71\%$, $H^2 = 1.99$						
Test of $\theta_i = \theta_j$: Q(10) = 21.90, p = 0.02						
Test of group differences: $Q_b(2) = 1.56$, p = 0.46						

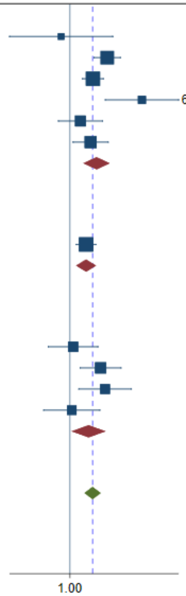

Random-effects REML model

eFigure 104. Pooled Odds of severity in patients with Decreased neutrophil- Forest plot

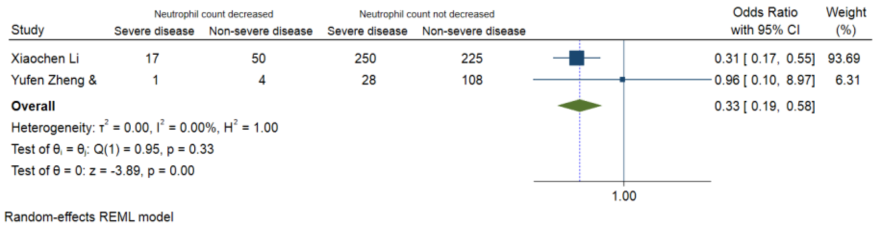

eFigure 105. Pooled Odds of severity in patients with Increased neutrophil- Forest plot

Study	Neutrophil count elevated: Severe disease	Neutrophil count elevated: Non-severe disease	Neutrophil count not elevated: Severe disease	Neutrophil count not elevated: Non-severe disease	Odds Ratio with 95% CI	Weight (%)
Xin Ying Zhao	4	7	26	54	1.19 [0.32, 4.42]	22.12
Yufen Zheng &	8	8	21	104	4.95 [1.67, 14.68]	27.82
Ling Hu %	61	39	104	101	1.52 [0.93, 2.47]	50.06
**Overall**					2.00 [0.93, 4.31]	

Heterogeneity: $\tau^2 = 0.25$, $I^2 = 52.14\%$, $H^2 = 2.09$

Test of $\theta_i = \theta_j$: $Q(2) = 4.18$, $p = 0.12$

Test of $\theta = 0$: $z = 1.76$, $p = 0.08$

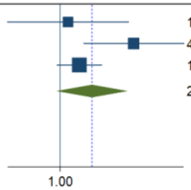

Random-effects REML model

eFigure 106. Pooled Odds of severity in patients with Decreased lymphocyte- Forest plot

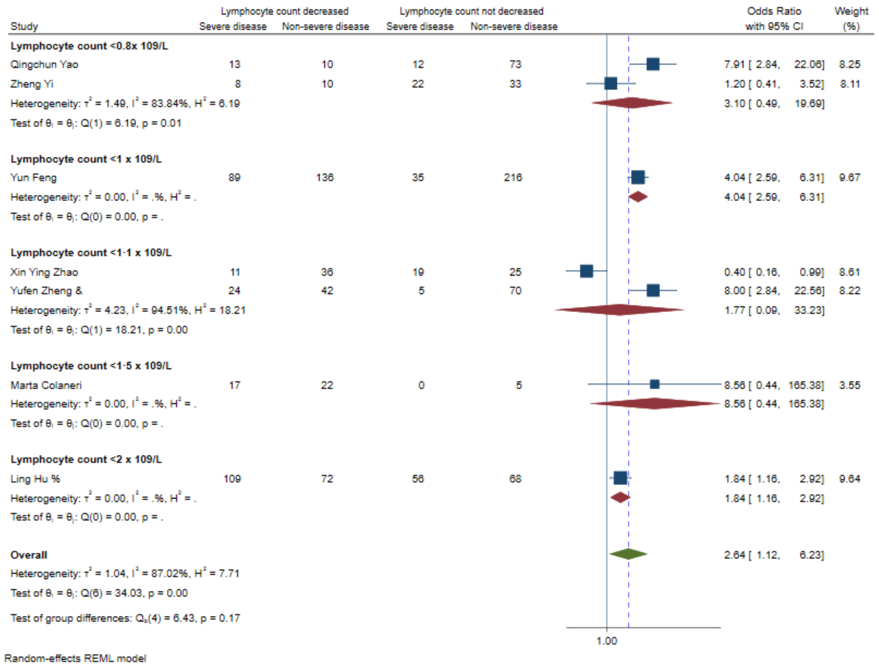

eFigure 107. Pooled Odds of severity in patients with Decreased platelet- Forest plot

Study	Platelet count decreased: Severe disease	Platelet count decreased: Non-severe disease	Platelet count not decreased: Severe disease	Platelet count not decreased: Non-severe disease	Odds Ratio with 95% CI	Weight (%)
**Platelet count < 100 x 109/L**						
Fang Zheng	3	8	27	123	1.71 [0.43, 6.86]	4.98
Qingchun Yao	4	6	21	77	2.44 [0.63, 9.47]	5.22
Ling Hu %	12	4	153	134	2.63 [0.83, 8.34]	6.83
Heterogeneity: τ^2 = 0.00, I^2 = 0.00%, H^2 = 1.00					2.27 [1.08, 4.78]	
Test of $\theta_i = \theta_j$: Q(2) = 0.23, p = 0.89						
**Platelet count < 125 x 109/L**						
Suxin Wan +	12	11	28	84	3.27 [1.30, 8.24]	9.71
Huan Li %	12	7	60	53	1.51 [0.56, 4.13]	8.56
Yufen Zheng &	7	5	21	107	7.13 [2.07, 24.63]	6.07
Heterogeneity: τ^2 = 0.23, I^2 = 45.21%, H^2 = 1.83					3.09 [1.37, 6.99]	
Test of $\theta_i = \theta_j$: Q(2) = 3.70, p = 0.16						
**Platelet count < 150 x 109/L**						
Wei-jie Guan $	90	225	66	488	2.96 [2.07, 4.22]	26.78
Xiaochen Li	89	68	176	206	1.53 [1.05, 2.23]	25.89
Marta Colaneri	10	9	7	18	2.86 [0.82, 10.01]	5.95
Heterogeneity: τ^2 = 0.13, I^2 = 67.23%, H^2 = 3.05					2.22 [1.32, 3.73]	
Test of $\theta_i = \theta_j$: Q(2) = 6.43, p = 0.04						
**Overall**					2.39 [1.72, 3.34]	
Heterogeneity: τ^2 = 0.07, I^2 = 34.31%, H^2 = 1.52						
Test of $\theta_i = \theta_j$: Q(8) = 11.24, p = 0.19						
Test of group differences: $Q_b(2)$ = 0.48, p = 0.79						

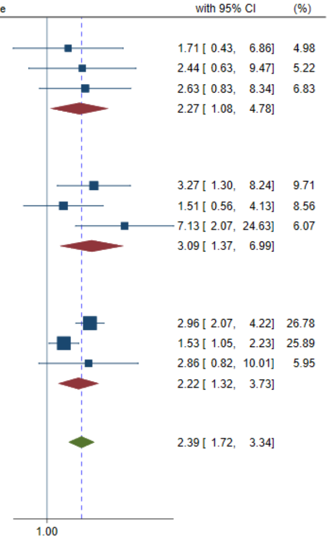

Random-effects REML model

eFigure 108. Pooled Odds of severity in patients with Decreased Albumin- Forest plot

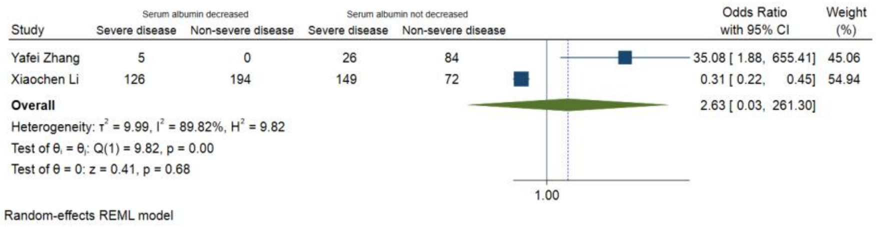

eFigure 109. Pooled Odds of severity in patients with Increased globulin- Forest plot

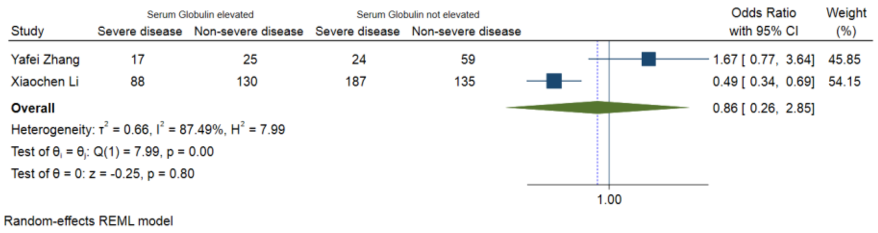

eFigure 110. Pooled Odds of severity in patients with Increased Total bilirubin- Forest plot

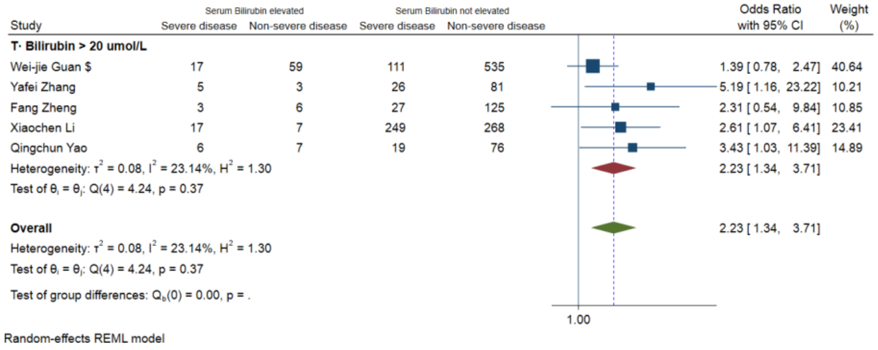

eFigure 111. Pooled Odds of severity in patients with Increased AST- Forest plot

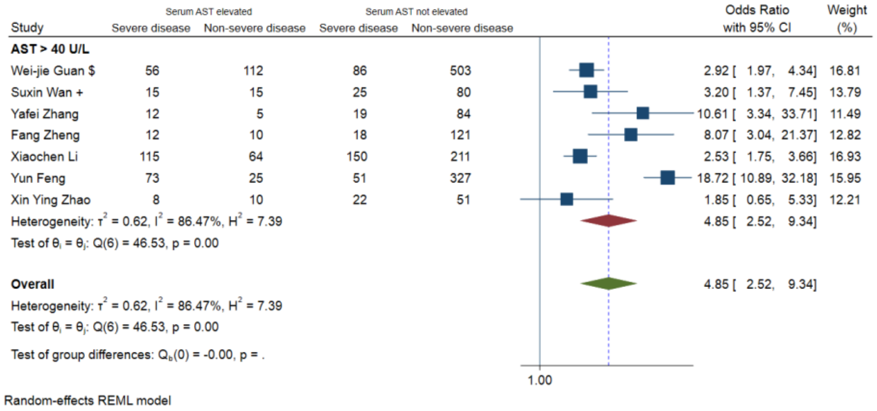

eFigure 112. Pooled Odds of severity in patients with Increased ALT- Forest plot

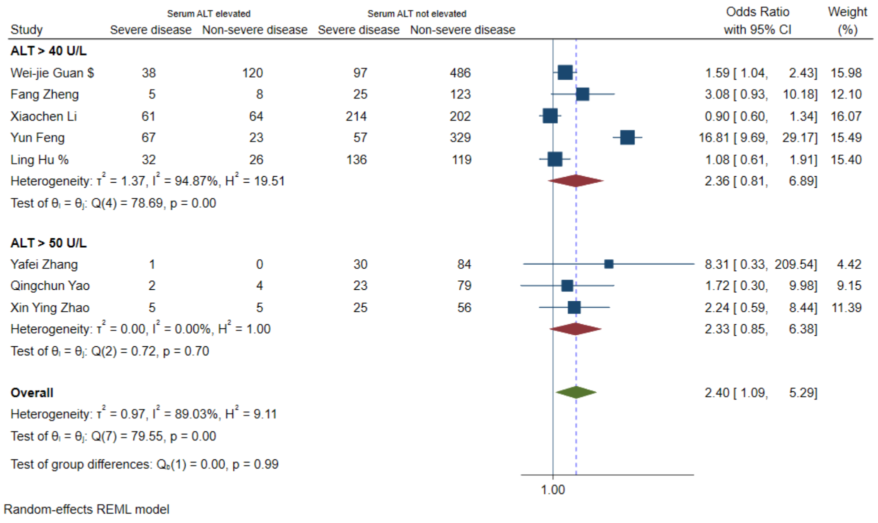

eFigure 113. Pooled Odds of severity in patients with Increased PT- Forest plot

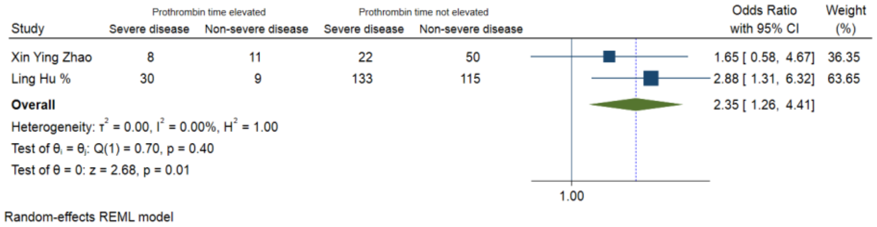

eFigure 114. Pooled Odds of severity in patients with Elevated CK Total- Forest plot

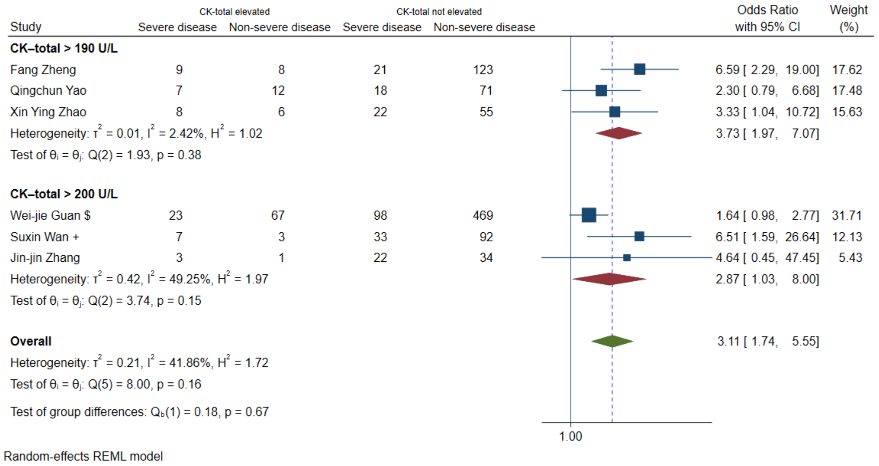

eFigure 115. Pooled Odds of severity in patients with Elevated CK MB- Forest plot

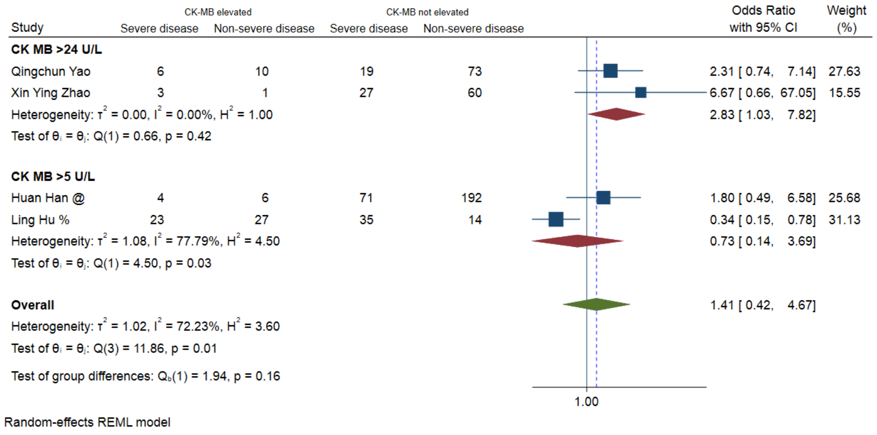

eFigure 116. Pooled Odds of severity in patients with Increased BUN- Forest plot

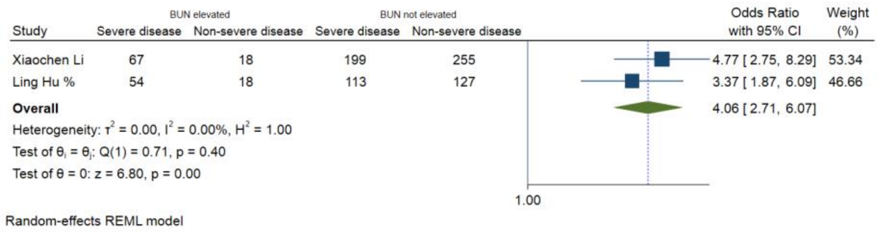

eFigure 117. Pooled Odds of severity in patients with Increased creatinine- Forest plot

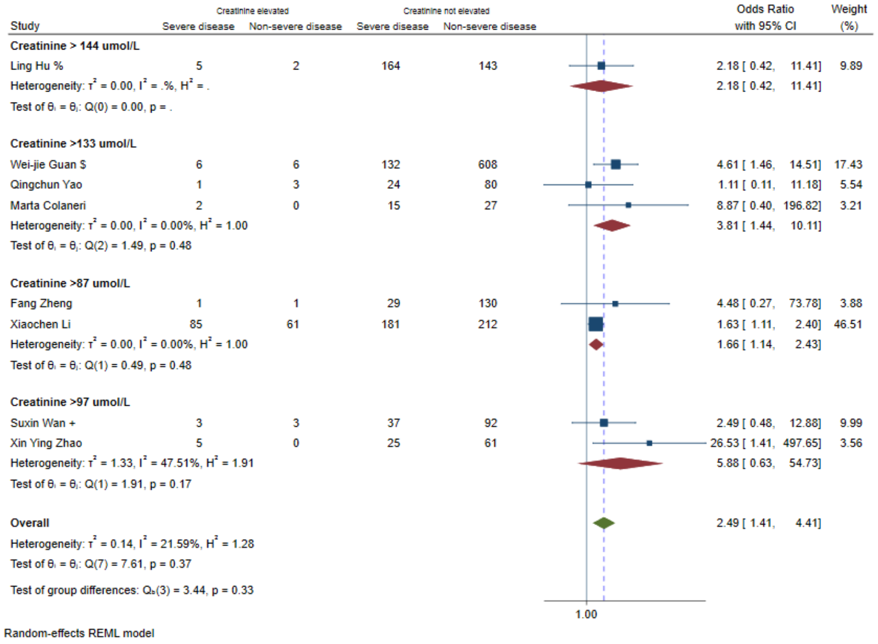

eFigure 118. Pooled Odds of severity in patients with Elevated LDH- Forest plot

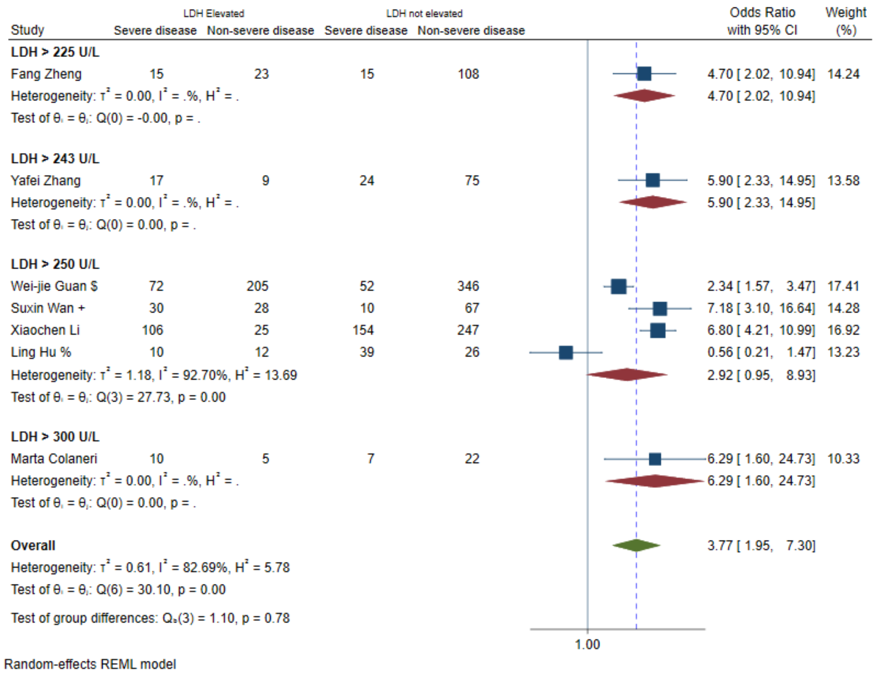

eFigure 119. Pooled Odds of severity in patients with Elevated D-Dimer- Forest plot

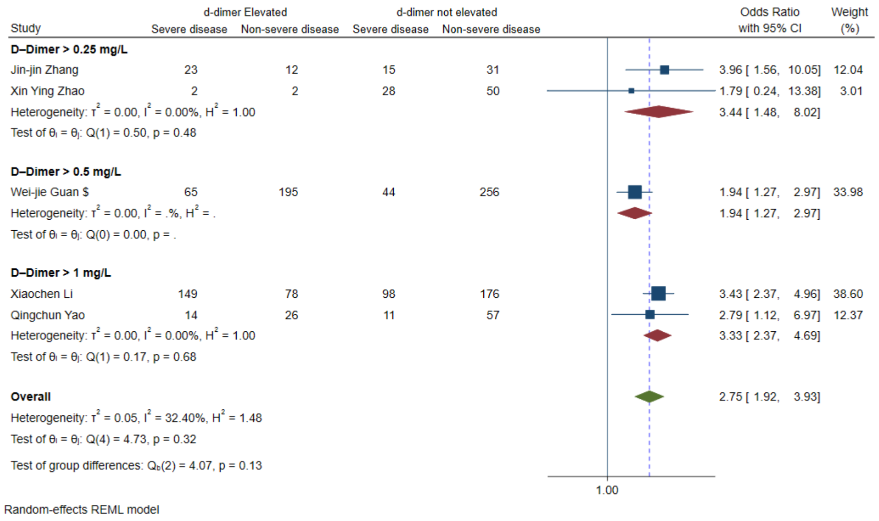

eFigure 120. Pooled Odds of severity in patients with Increased CRP- Forest plot

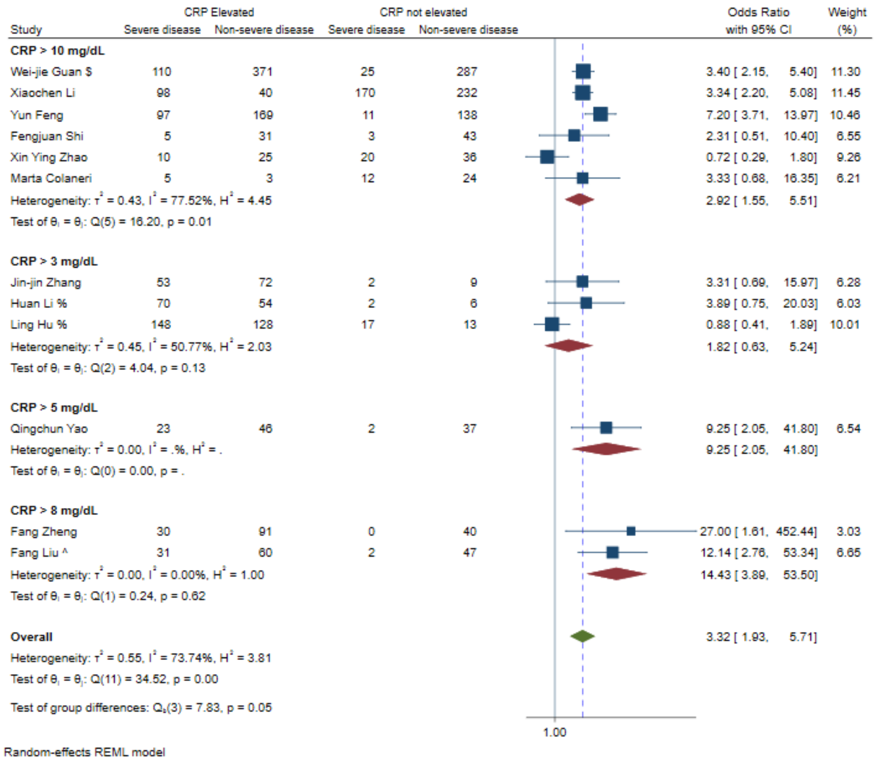

eFigure 121. Pooled Odds of severity in patients with Elevated Procalcitonin- Forest plot

Study	Procalcitonin Elevated Severe disease	Procalcitonin Elevated Non-severe disease	Procalcitonin not elevated Severe disease	Procalcitonin not elevated Non-severe disease	Odds Ratio with 95% CI	Weight (%)
**Procalcitonin >0·05 µg/L**						
Huan Li %	15	57	5	55	2.89 [0.99, 8.51]	12.70
Qingchun Yao	20	41	5	42	4.10 [1.41, 11.95]	12.82
Heterogeneity: τ^2 = 0.00, I^2 = 0.00%, H^2 = 1.00					3.45 [1.61, 7.37]	
Test of $\theta_i = \theta_j$: Q(1) = 0.20, p = 0.65						
**Procalcitonin >0·1 µg/L**						
Suxin Wan +	19	6	21	89	13.42 [4.77, 37.73]	13.38
Jin-jin Zhang	25	16	25	52	3.25 [1.48, 7.15]	17.93
Heterogeneity: τ^2 = 0.79, I^2 = 78.13%, H^2 = 4.57					6.34 [1.58, 25.38]	
Test of $\theta_i = \theta_j$: Q(1) = 4.57, p = 0.03						
**Procalcitonin >0·5 µg/L**						
Wei-jie Guan $	16	19	101	497	4.14 [2.06, 8.33]	19.96
Xiaochen Li	43	3	194	246	18.18 [5.55, 59.47]	11.23
Fang Liu ^	5	3	28	104	6.19 [1.39, 27.49]	8.08
Xin Ying Zhao	3	1	27	58	6.44 [0.64, 64.84]	3.91
Heterogeneity: τ^2 = 0.26, I^2 = 39.61%, H^2 = 1.66					6.97 [3.12, 15.58]	
Test of $\theta_i = \theta_j$: Q(3) = 4.44, p = 0.22						
**Overall**					5.50 [3.38, 8.93]	
Heterogeneity: τ^2 = 0.18, I^2 = 38.70%, H^2 = 1.63						
Test of $\theta_i = \theta_j$: Q(7) = 10.69, p = 0.15						
Test of group differences: $Q_b(2)$ = 1.69, p = 0.43						

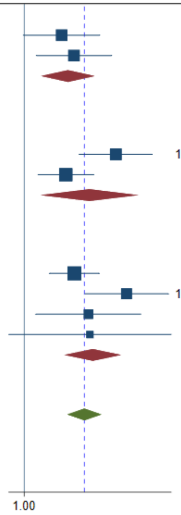

Random-effects REML model

eFigure 122. Pooled Odds of severity in patients with Increased SAA- Forest plot

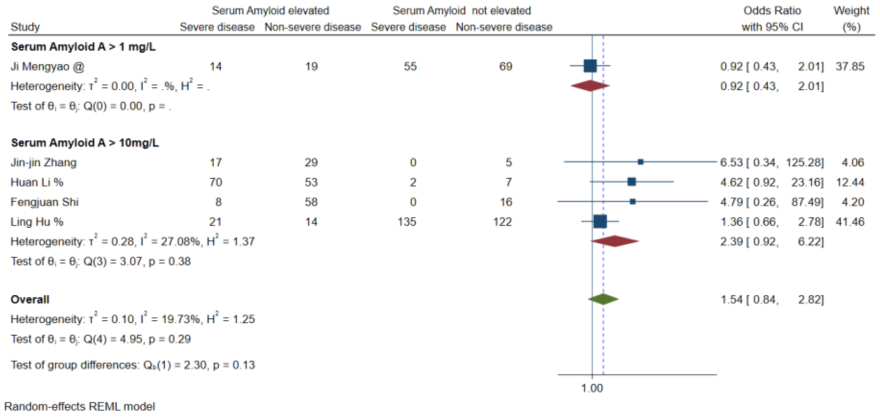

eFigure 123. Pooled Odds of severity in patients with Increased IL-6- Forest plot

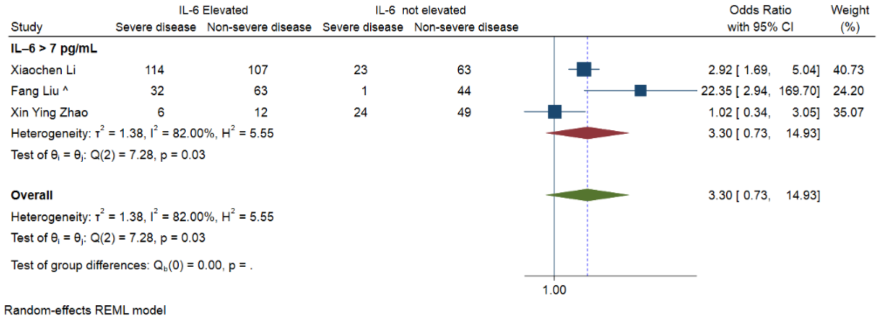

eFigure 124. Pooled Odds of severity in patients with Increased NT-ProBNP- Forest plot

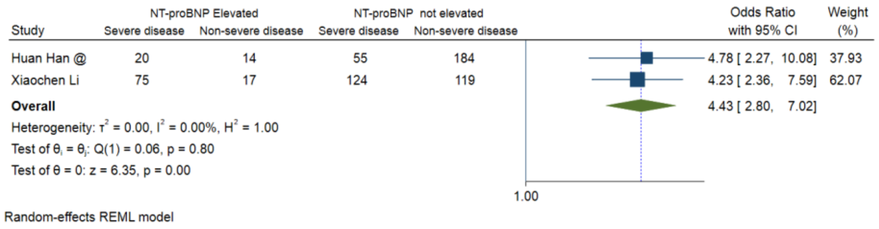

eFigure 125. Pooled Odds of severity in patients with Elevated troponin- Forest plot

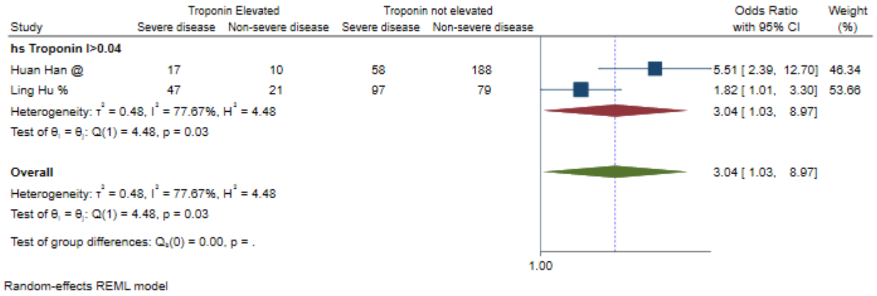

eFigure 126 Pooled Odds ratio of severe disease in patients with radiological peripheral lung involvement - Forest plot

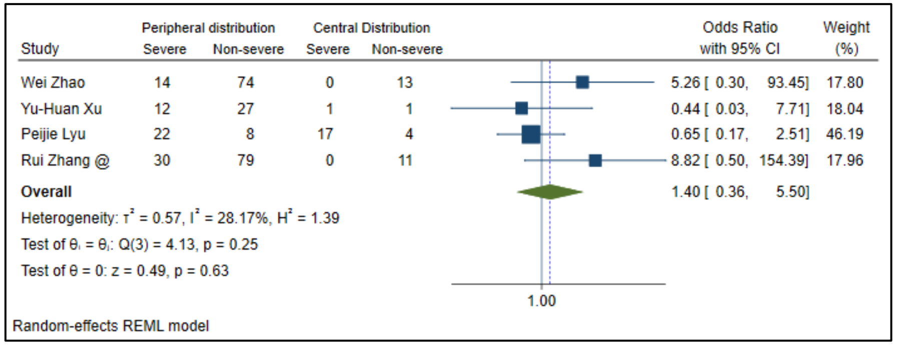

eFigure 127 Pooled Odds ratio of severe disease in patients with radiological bilateral lung involvement a) Forest plot b) Funnel Plot

127a

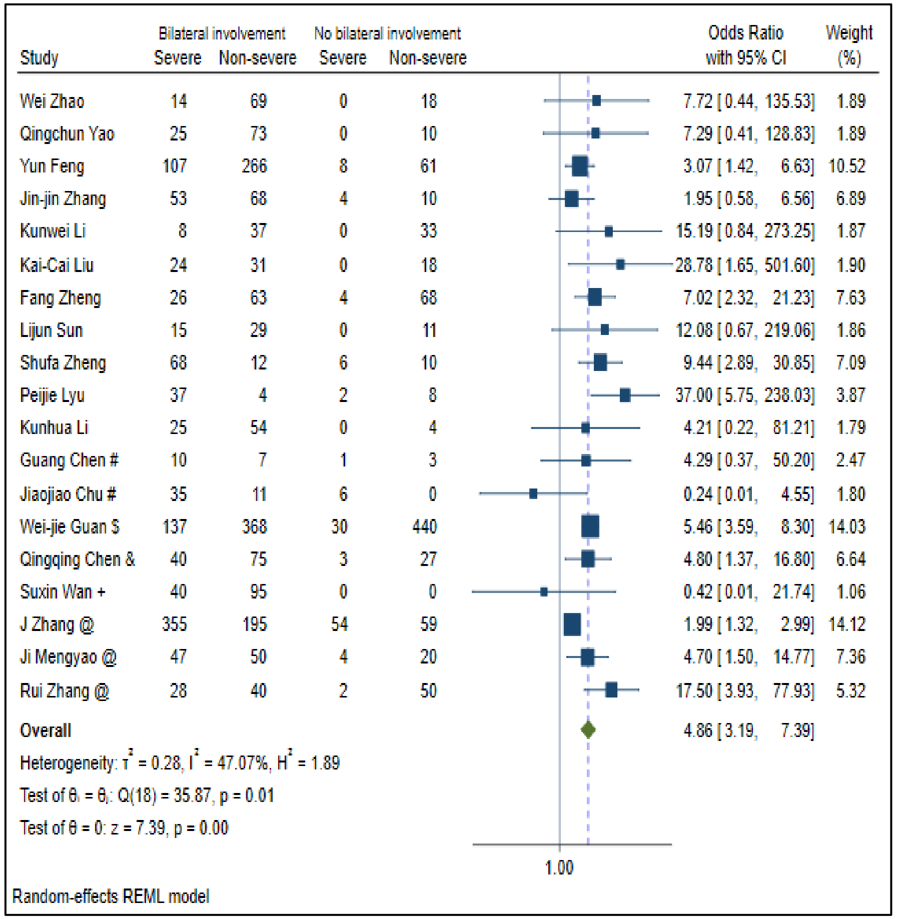

127b

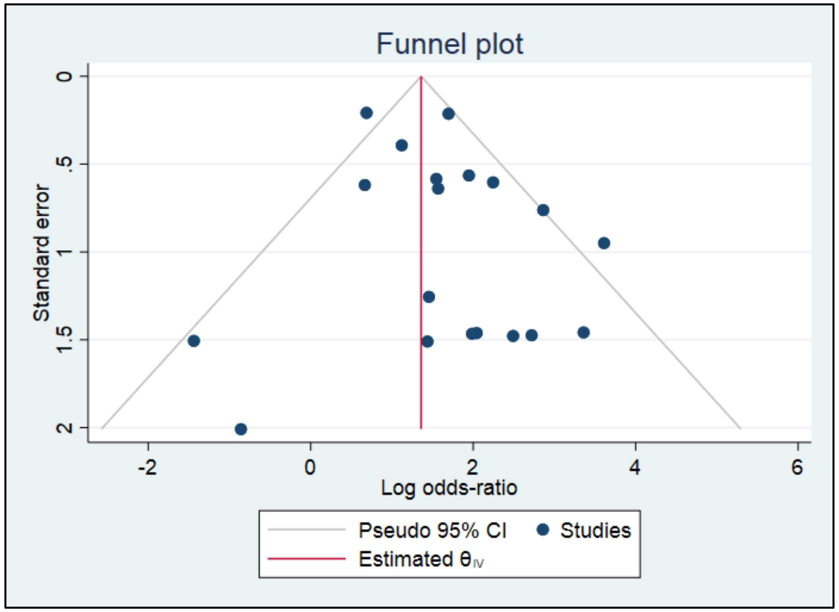

eFigure 128 Pooled Odds ratio of severe disease in patients with consolidation - Forest plot

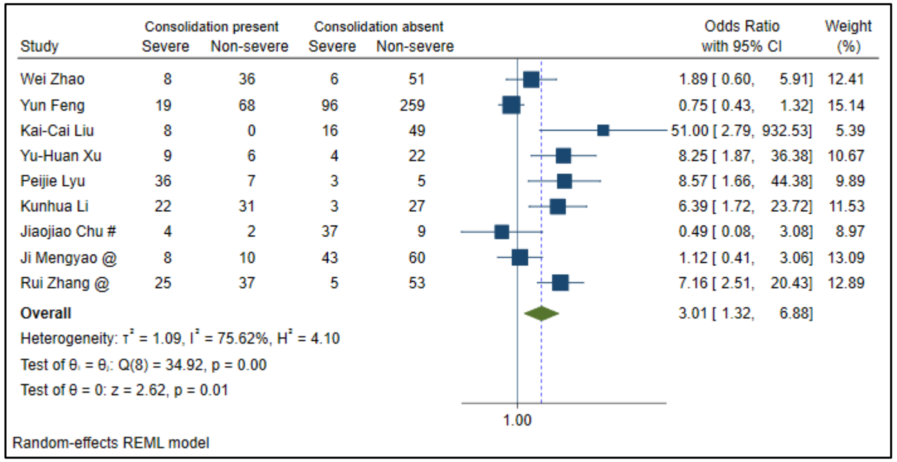

eFigure 129 Pooled Odds ratio of severe disease in patients with ground glass opacity - Forest plot

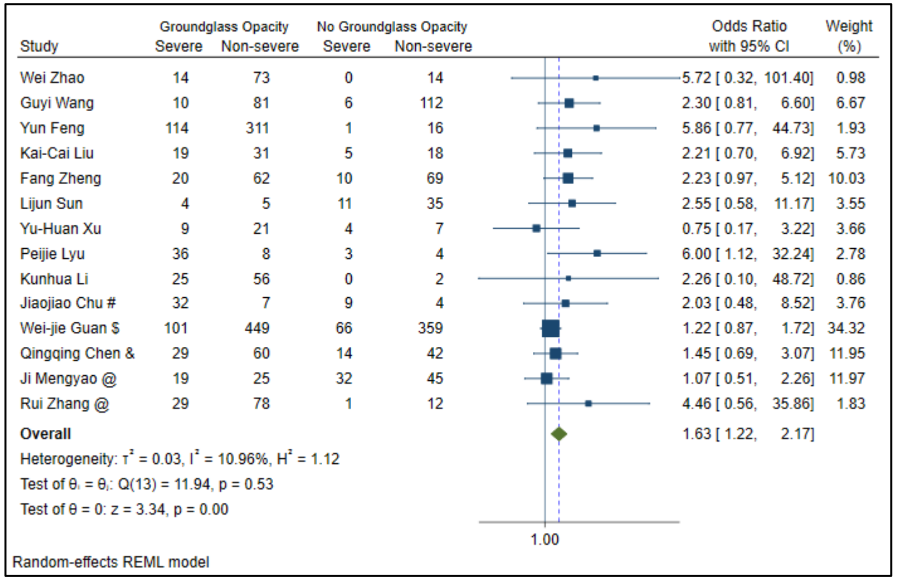

eFigure 130 Pooled Odds ratio of severe disease in patients with mixed GGO - Forest plot

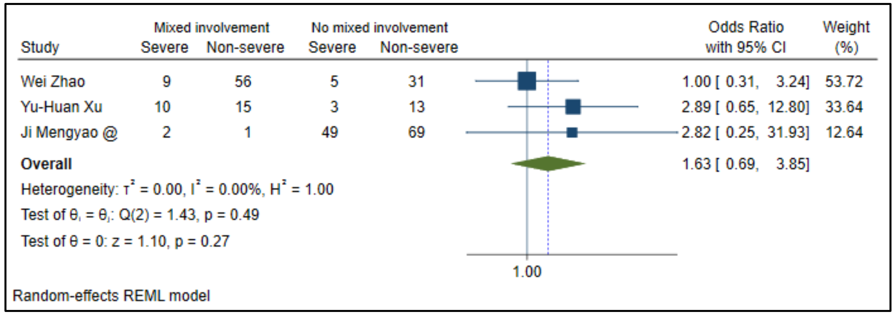

eFigure 131 Pooled Odds ratio of severe disease in patients with air bronchogram - Forest plot

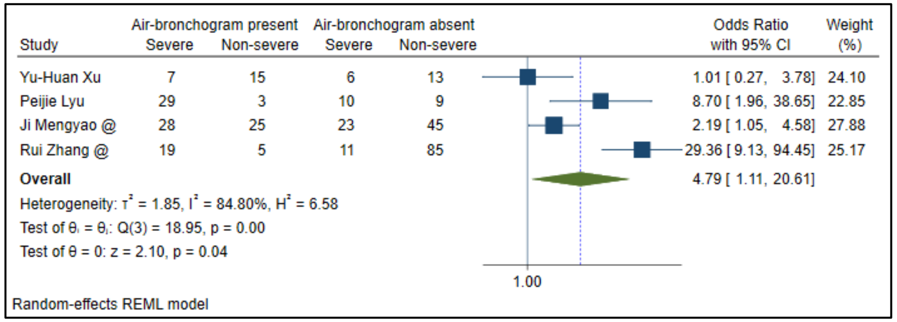

eFigure 132 Pooled Odds ratio of severe disease in patients with nodular infilterates - Forest plot

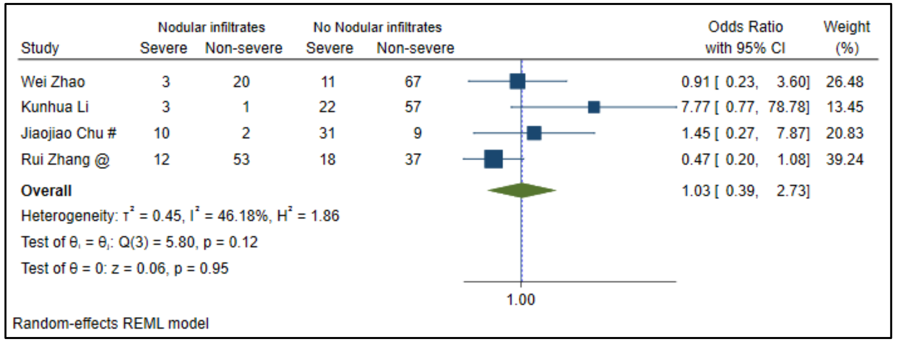

eFigure 133 Pooled Odds ratio of severe disease in patients with consolidation - Forest plot

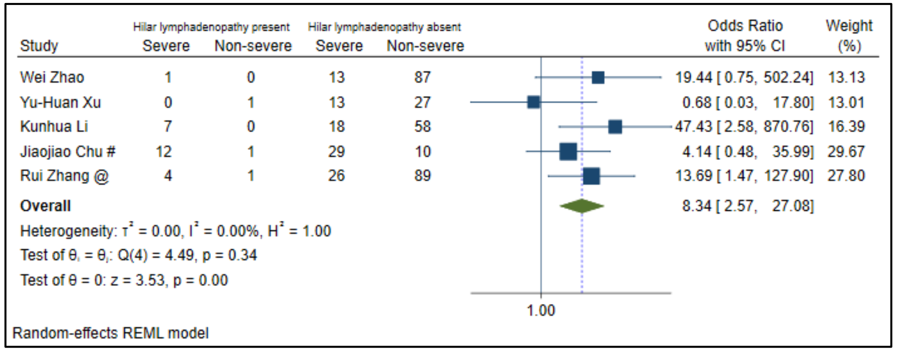

eFigure 134 Pooled Odds ratio of severe disease in patients with radiological unifocal involement - Forest plot

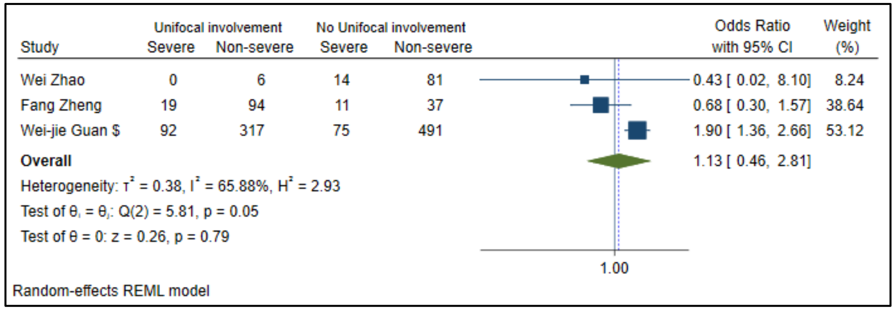

eFigure 135 Pooled Odds ratio of severe disease in patients with pleural effusion - Forest plot

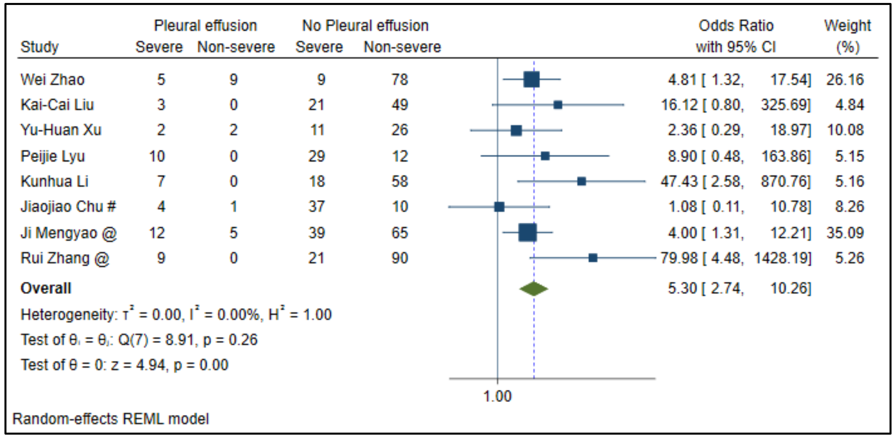

eFigure 136 Pooled Odds ratio of severe disease in patients with interlobular septal thickening - Forest plot

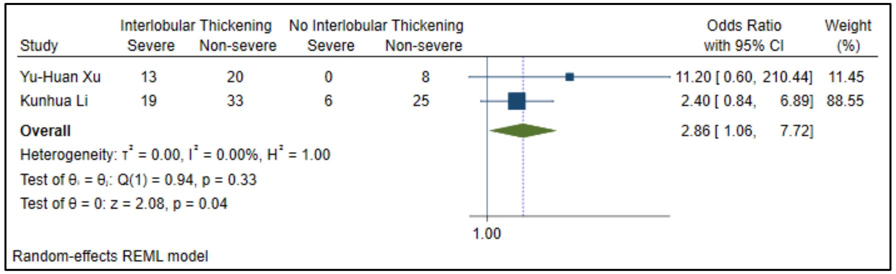

eFigure 137 Pooled Odds ratio of severe disease in patients with bronchiectasis- Forest plot

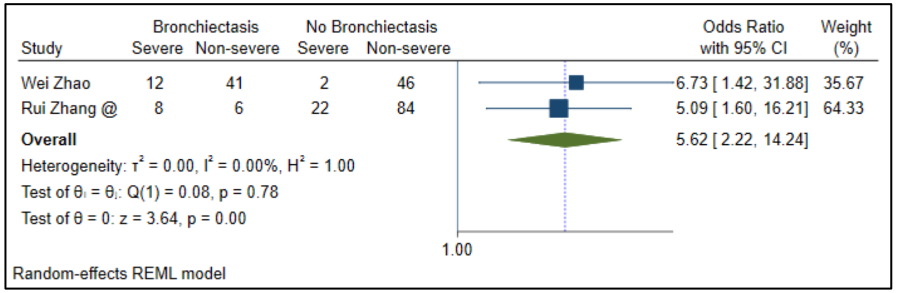

eFigure 138 Pooled Odds ratio of severe disease in patients with Linear opacity - Forest plot

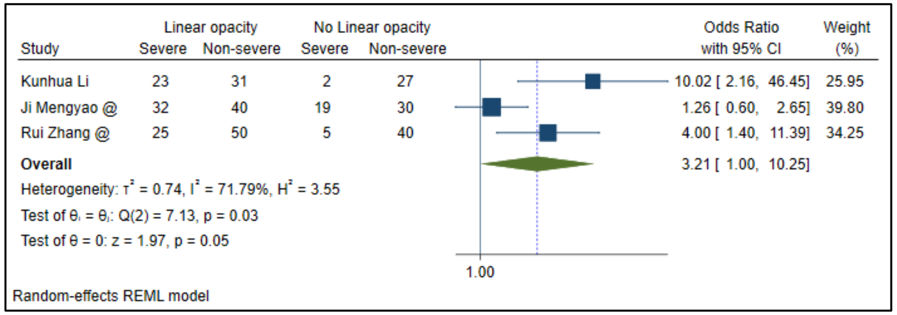

eFigure 139 Pooled Odds ratio of severe disease in patients with crazy pavement sign - Forest plot

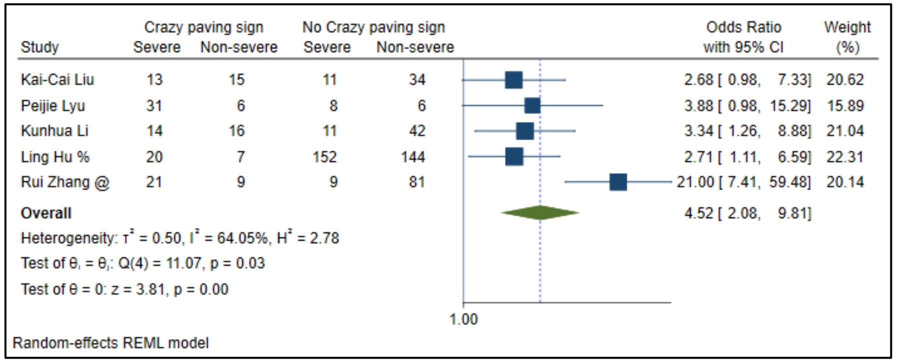

eFigure 140 Pooled Odds ratio of severe disease in patients with reticular pattern - Forest plot

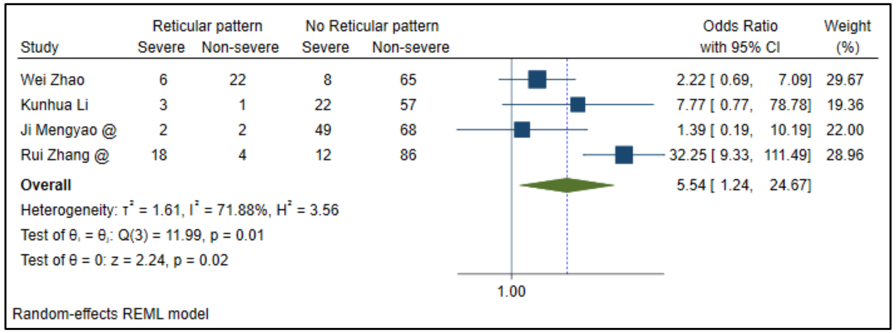

Section VII

Supplementary figures

Association of clinical and laboratory characteristics as continuous variables with mortality and severe disease in patients with COVID 19

eFigure 141 Percentage change in mortality with unit increase in mean age

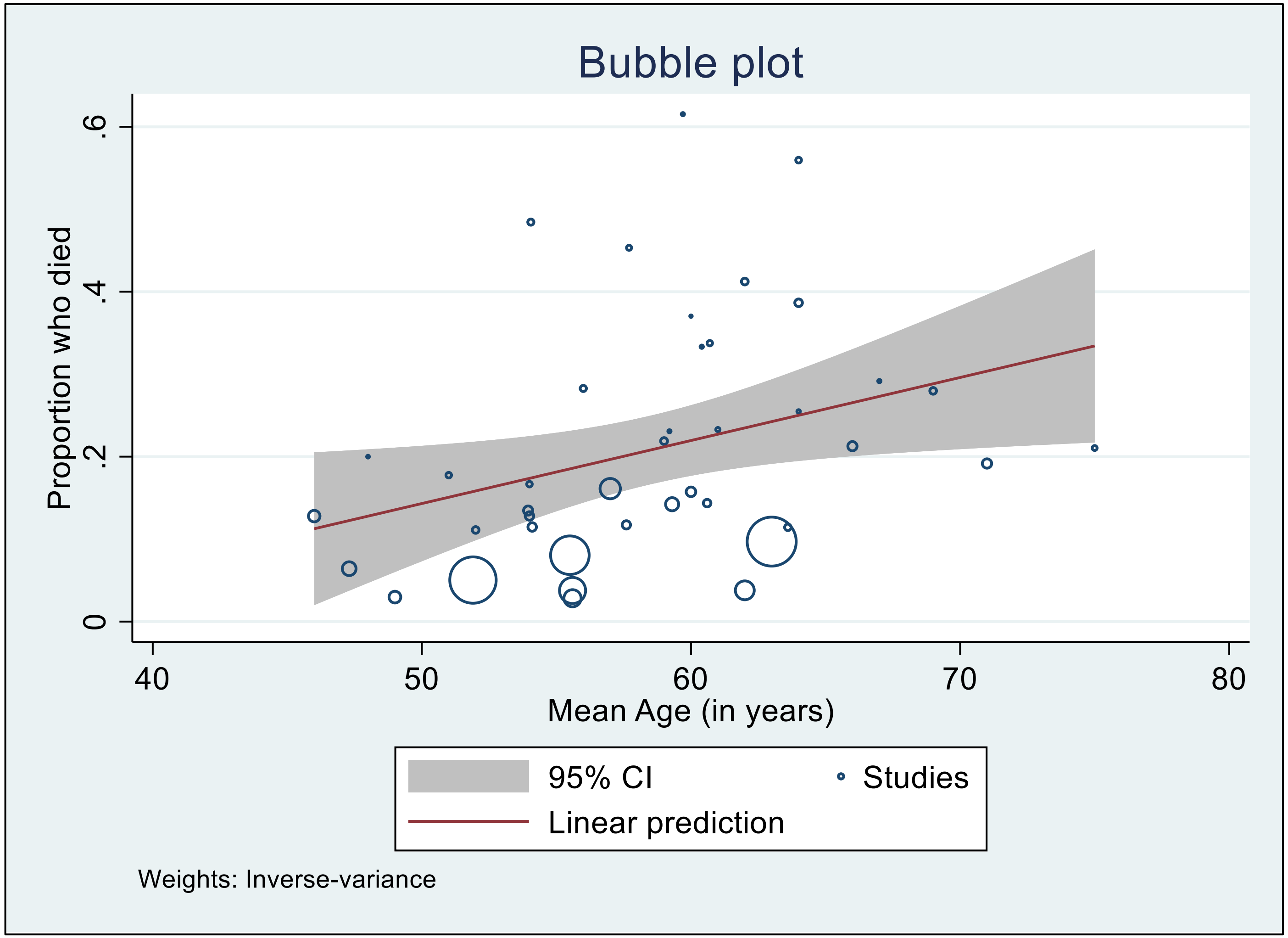

eFigure 142 Percentage change in mortality with unit increase in mean Hb

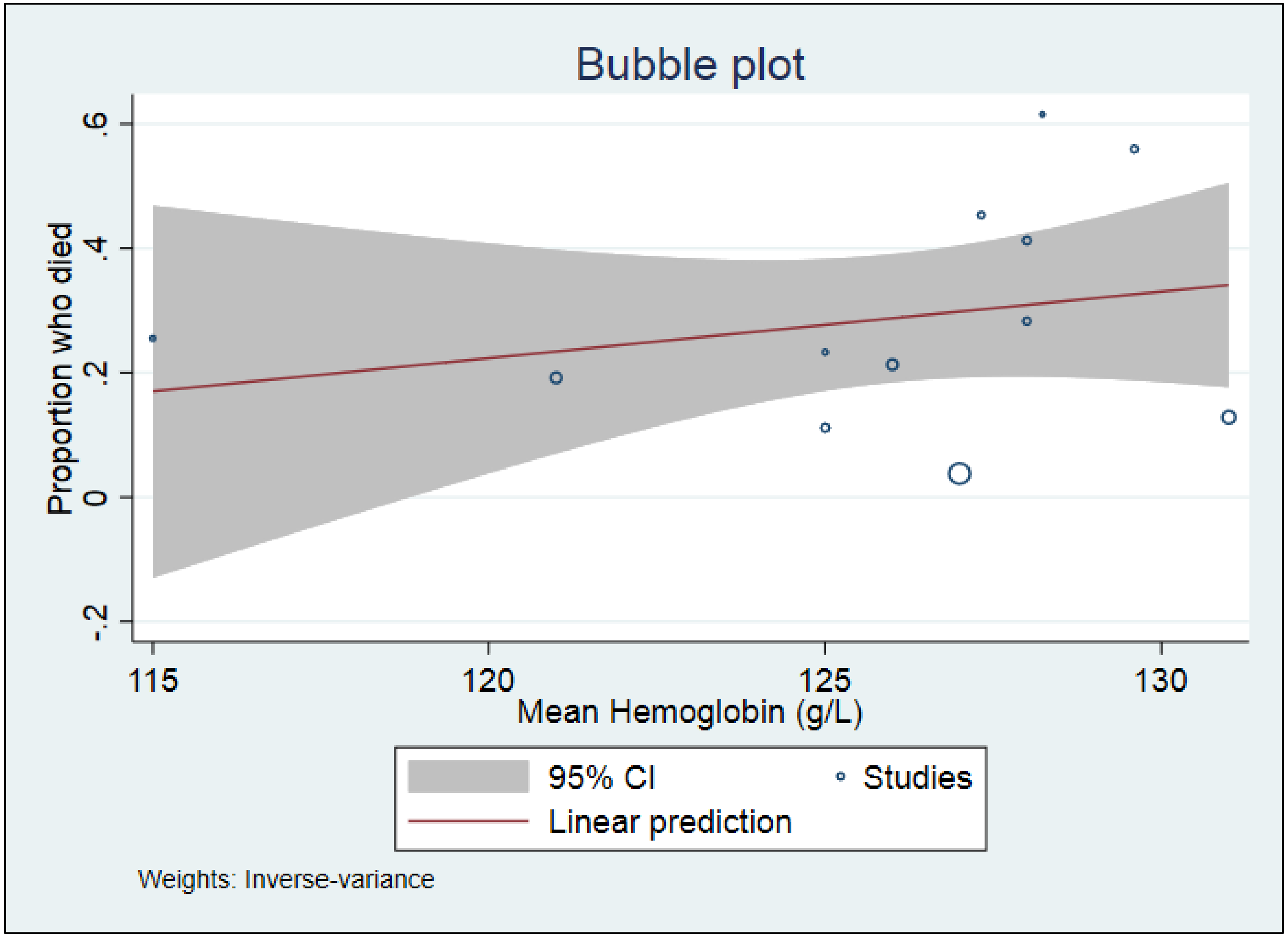

eFigure 143 Percentage change in mortality with unit increase in mean Total leukocyte count

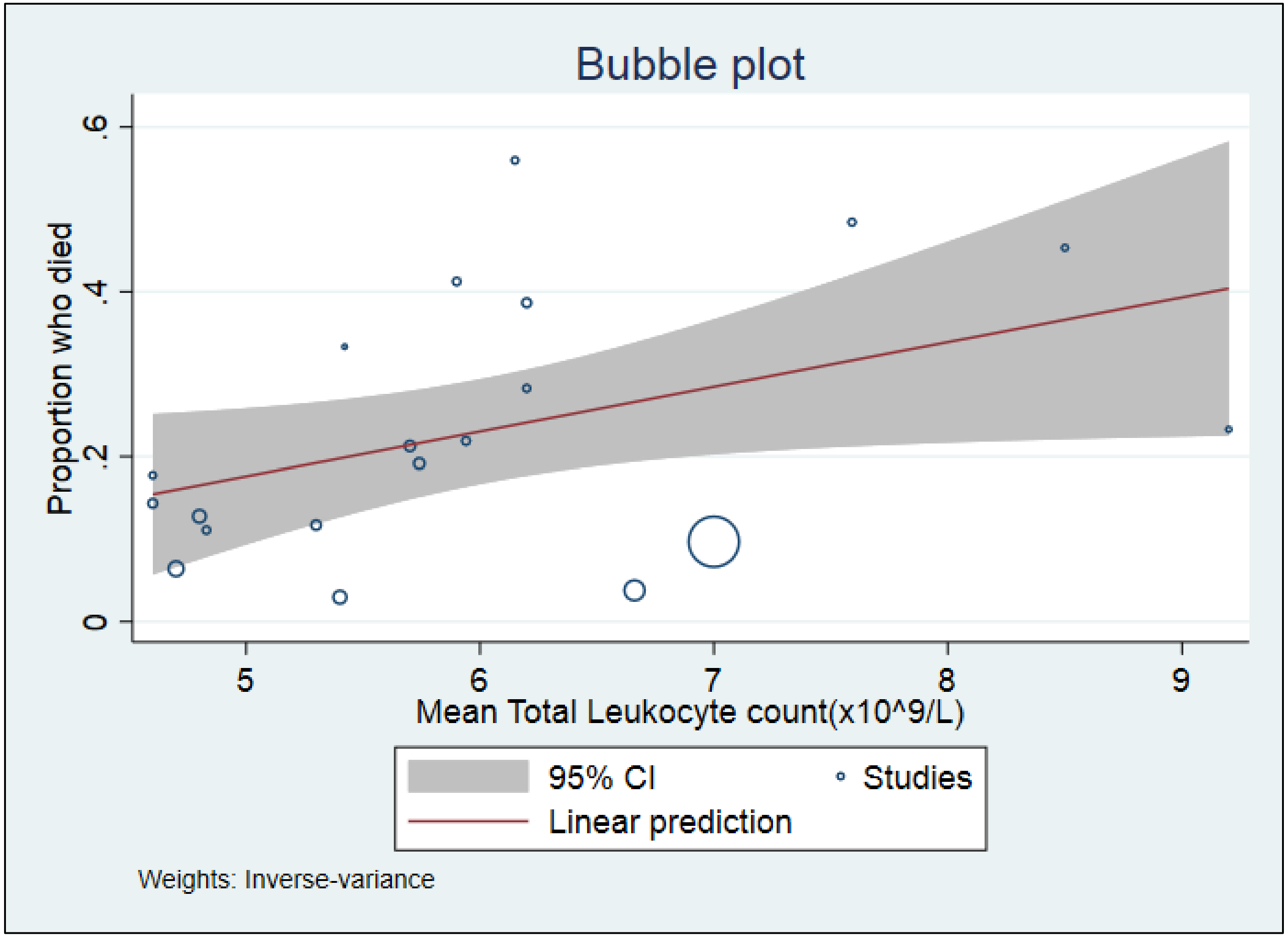

eFigure 144 Percentage change in mortality with unit increase in mean lymphocyte count

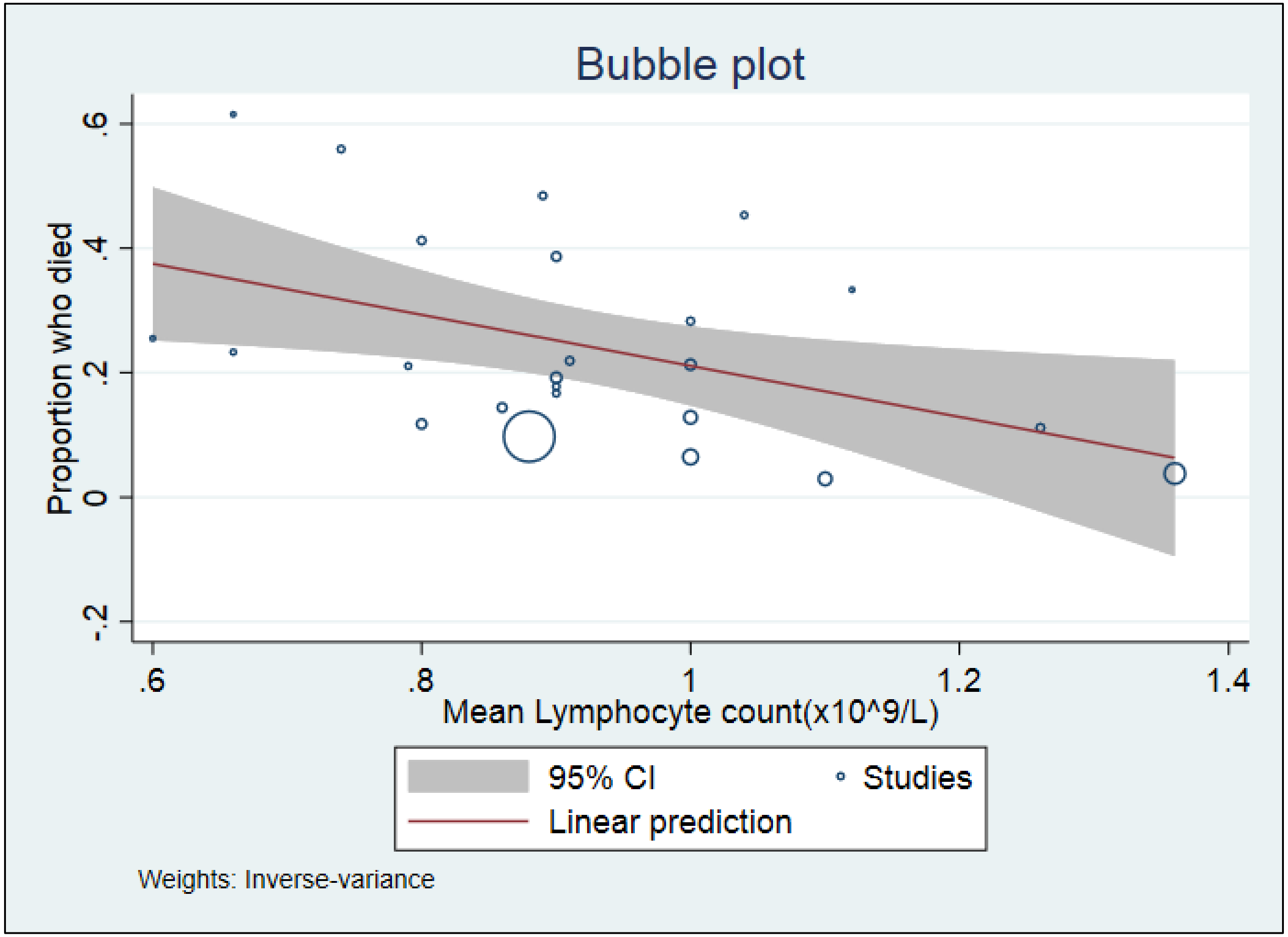

eFigure 145 Percentage change in mortality with unit increase in mean neutrophil count

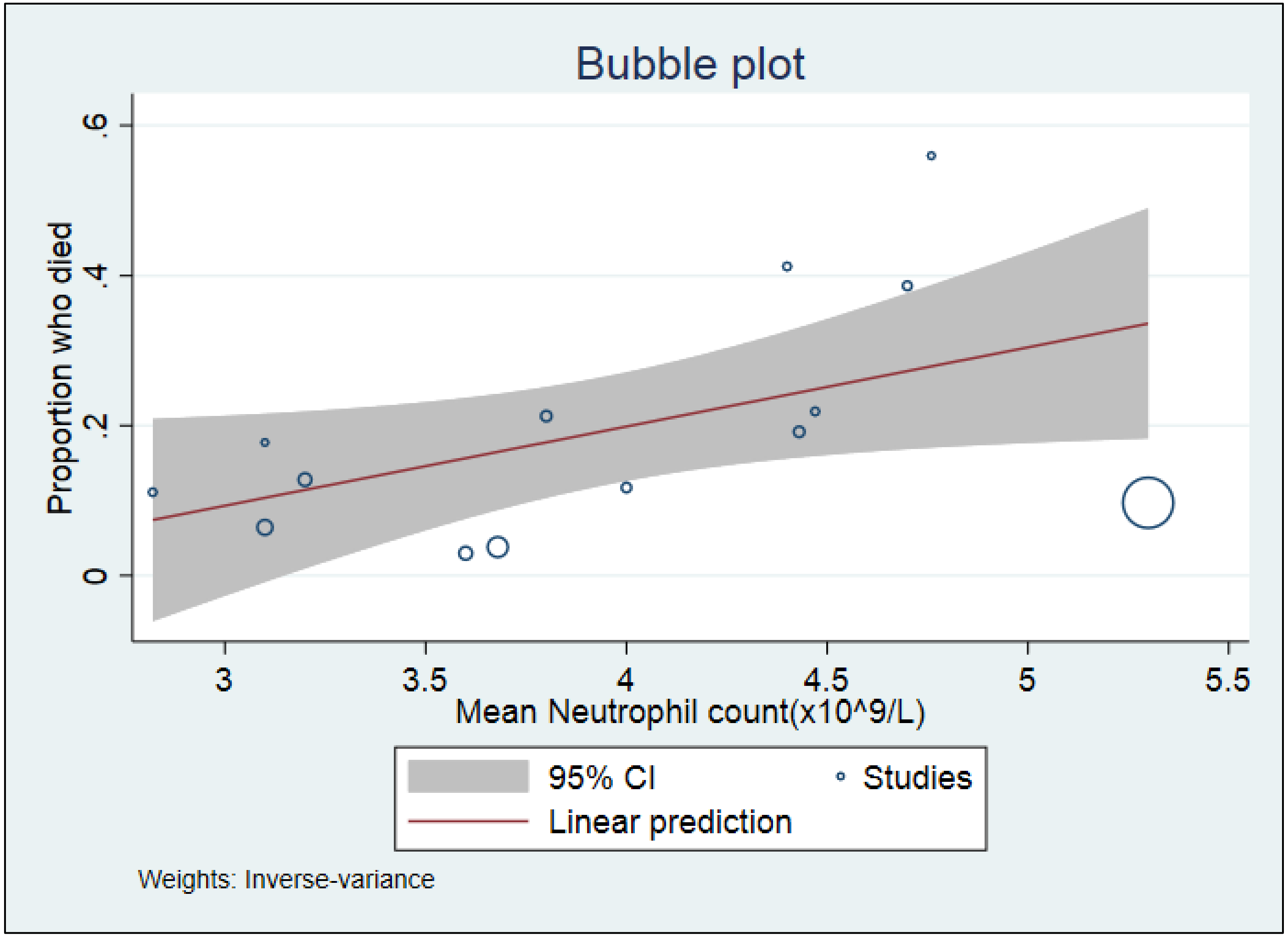

eFigure 146 Percentage change in mortality with unit increase in mean platelet count

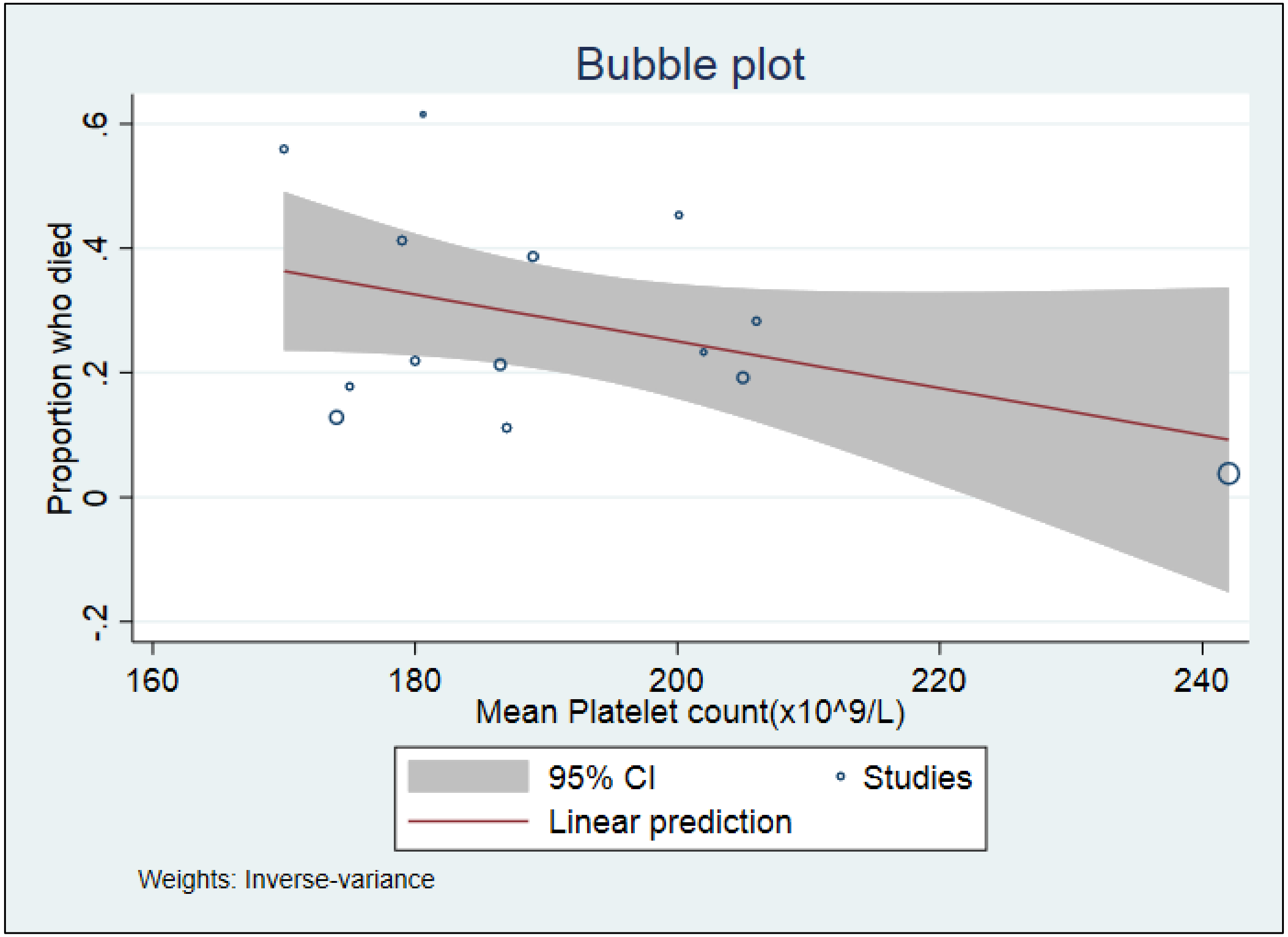

eFigure 147 Percentage change in mortality with unit increase in mean prothrombin time

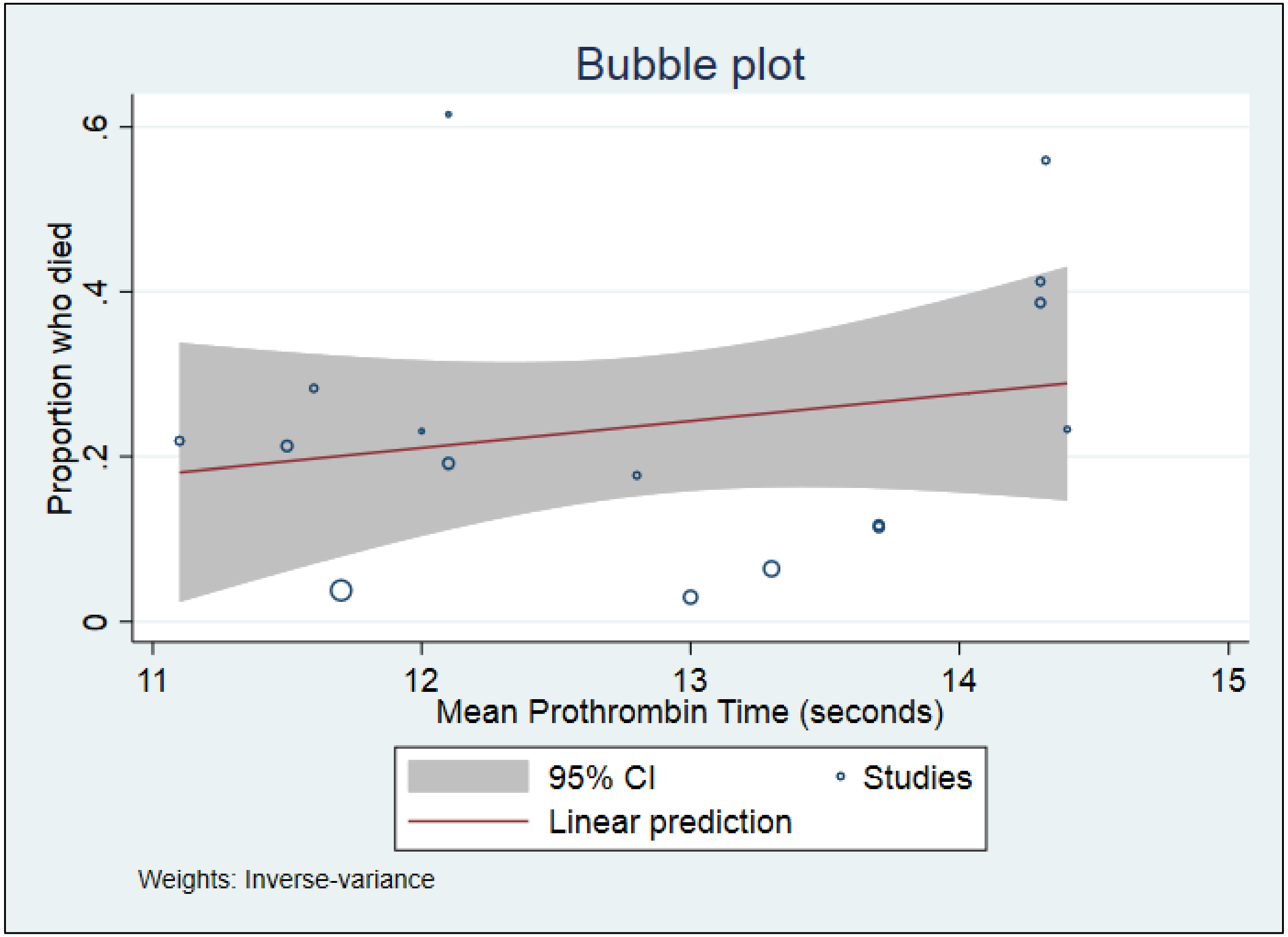

eFigure 148 Percentage change in mortality with unit increase in mean aPTT

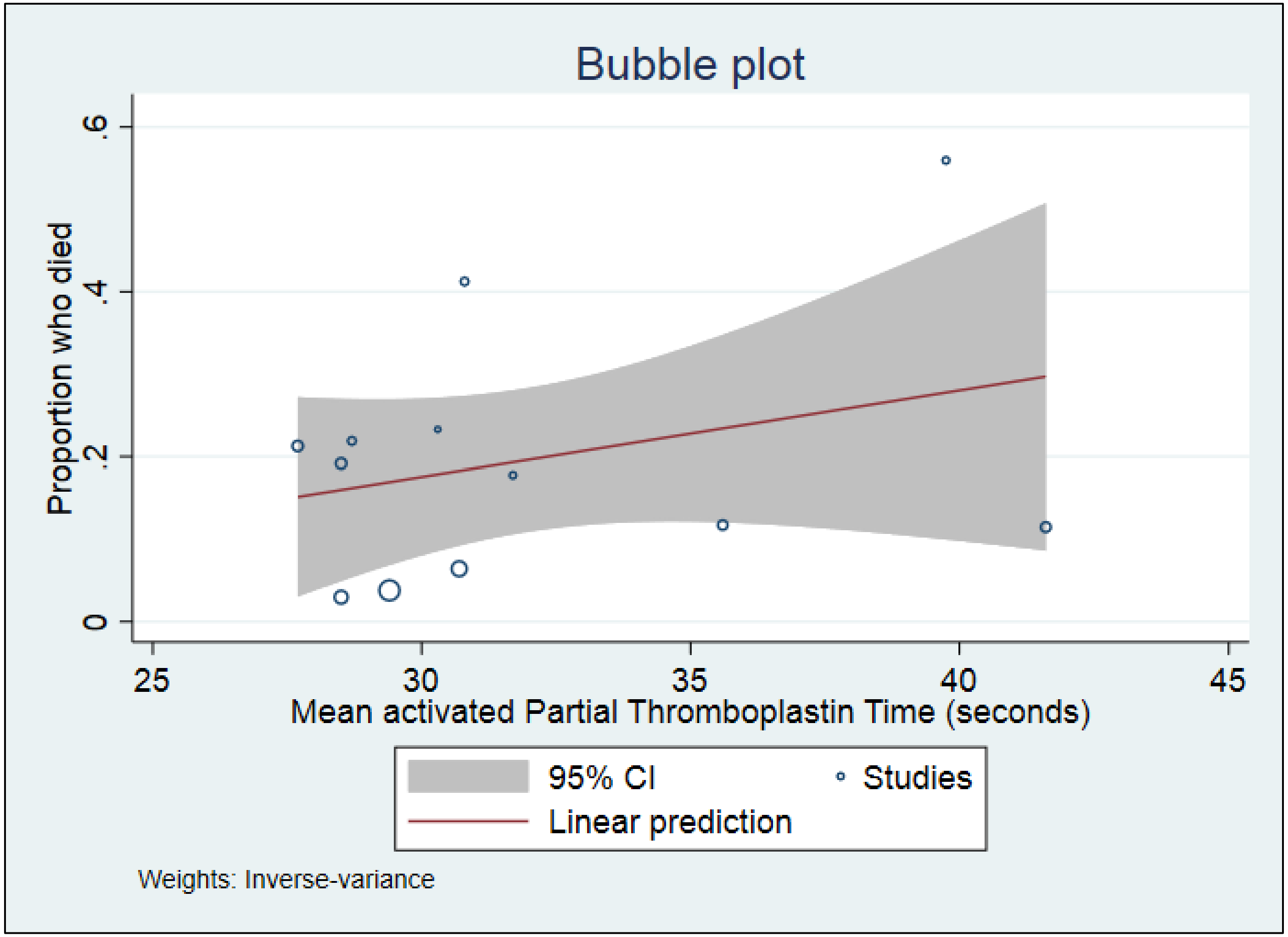

eFigure 149 Percentage change in mortality with unit increase in mean CRP

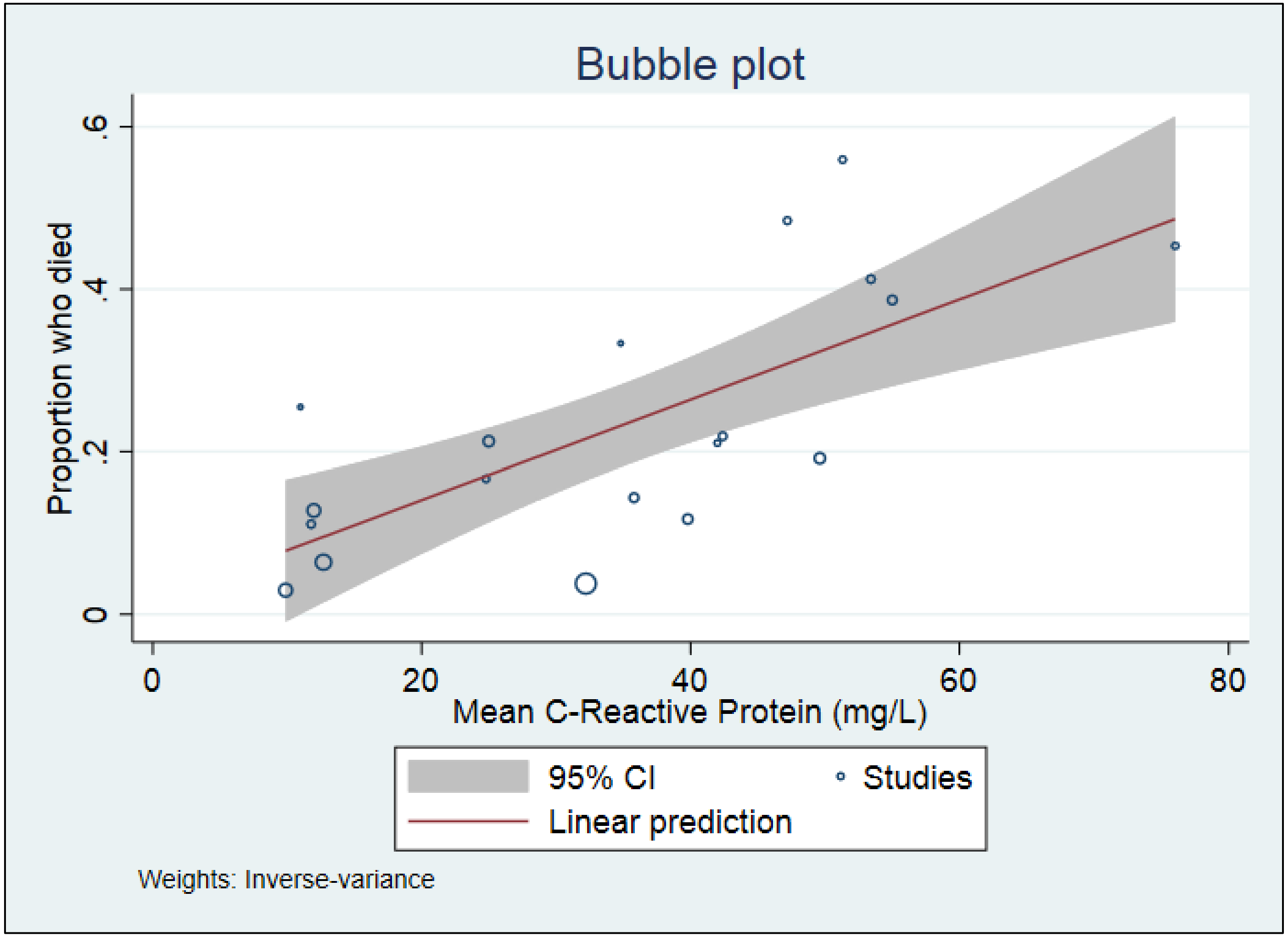

eFigure 150 Percentage change in mortality with unit increase in mean D dimer

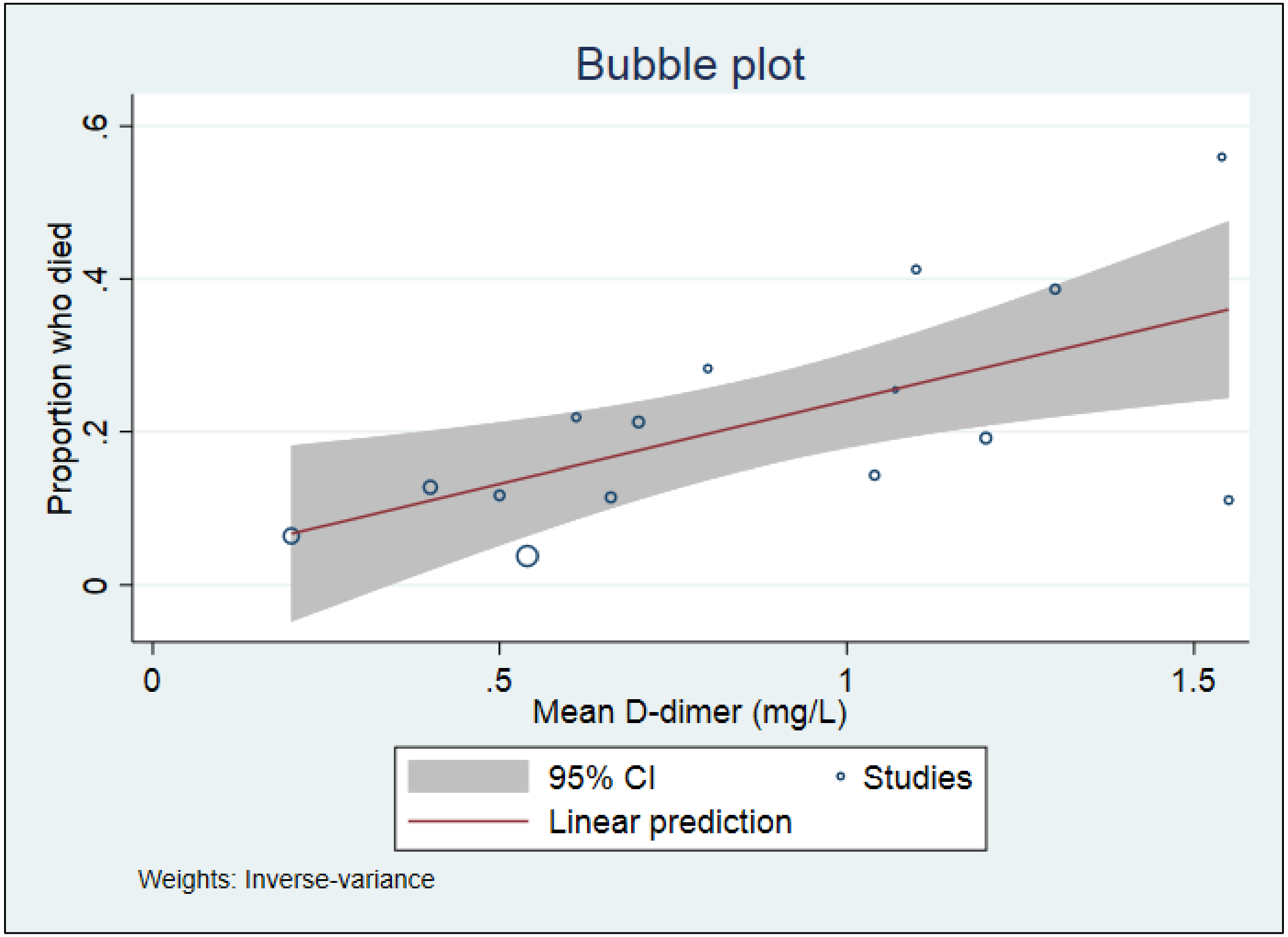

eFigure 151 Percentage change in mortality with unit increase in mean Pro-calcitonin

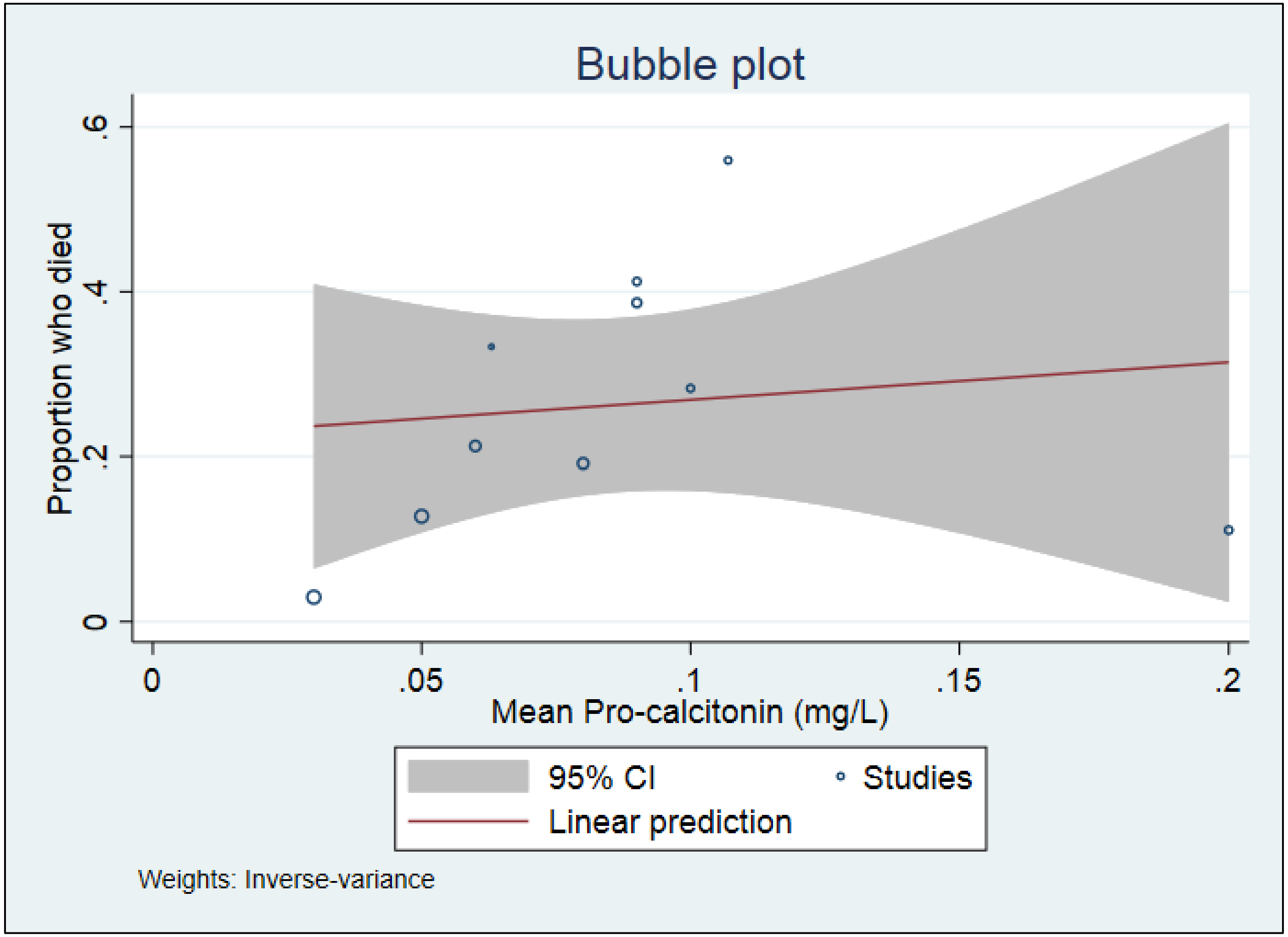

eFigure 152 Percentage change in mortality with unit increase in mean serum ferritin

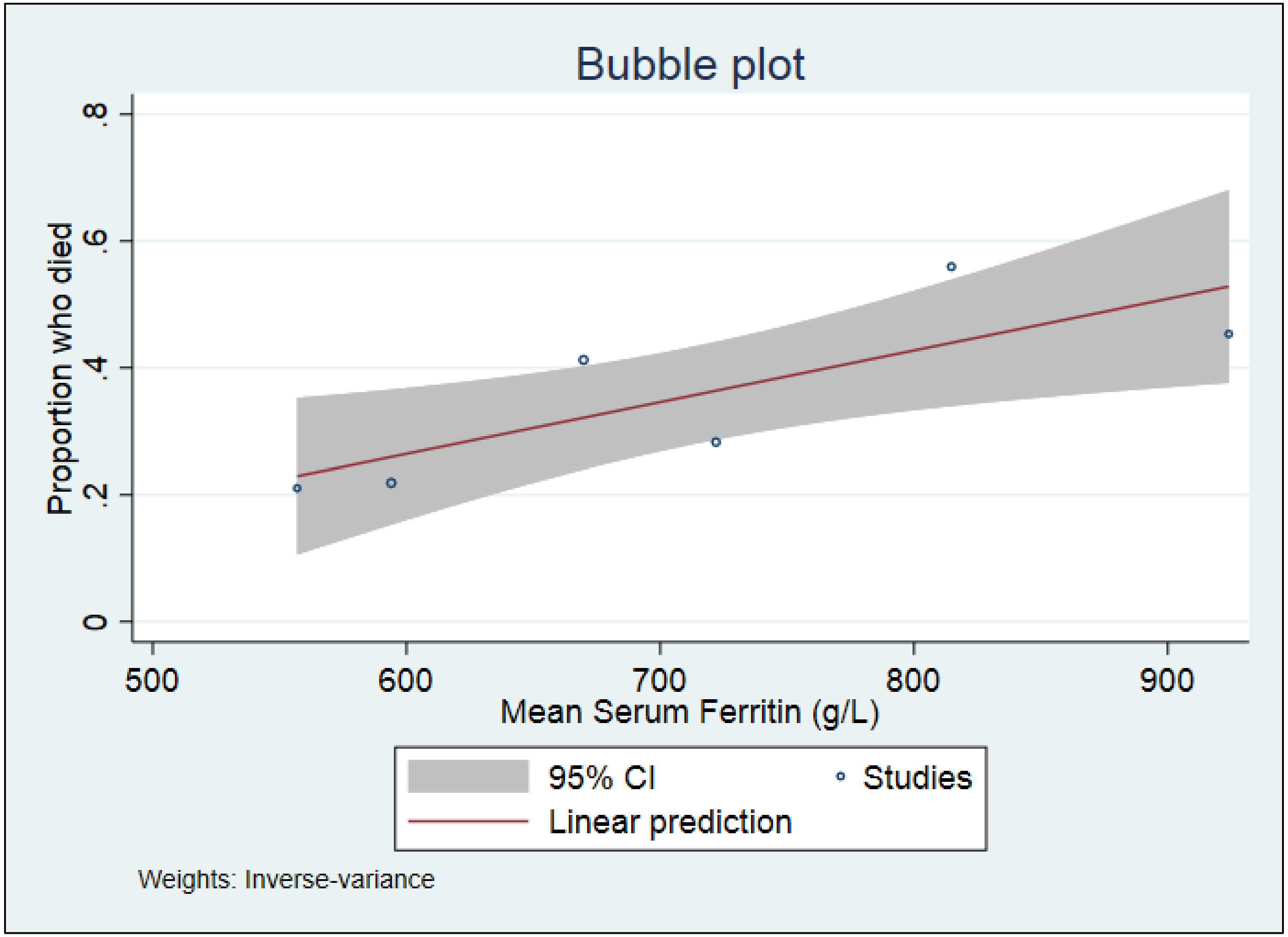

eFigure 153 Percentage change in mortality with unit increase in mean serum LDH

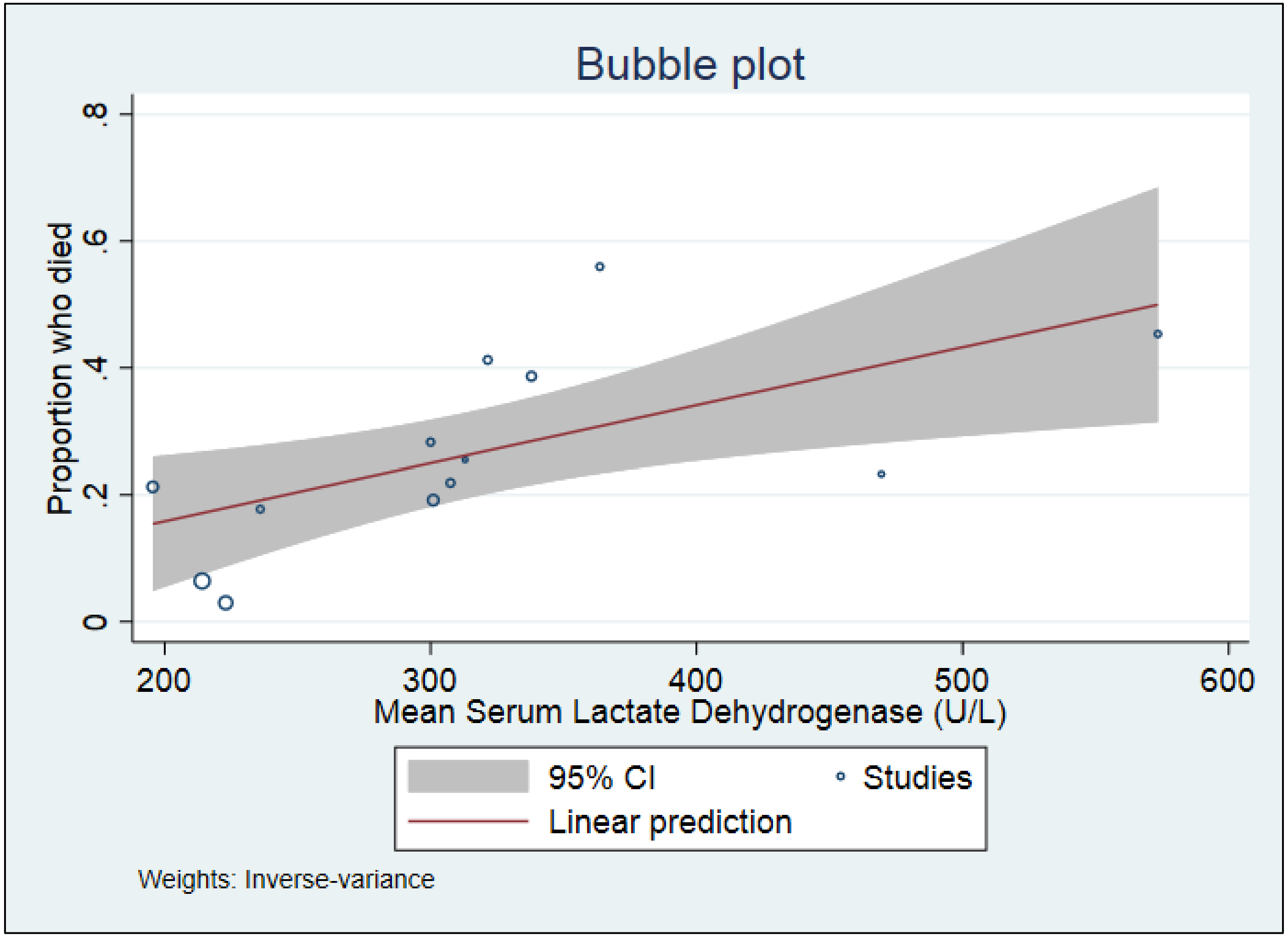

eFigure 154 Percentage change in mortality with unit increase in mean a) Serum creatinine b) Serum creatinine excluding critical patients

154a

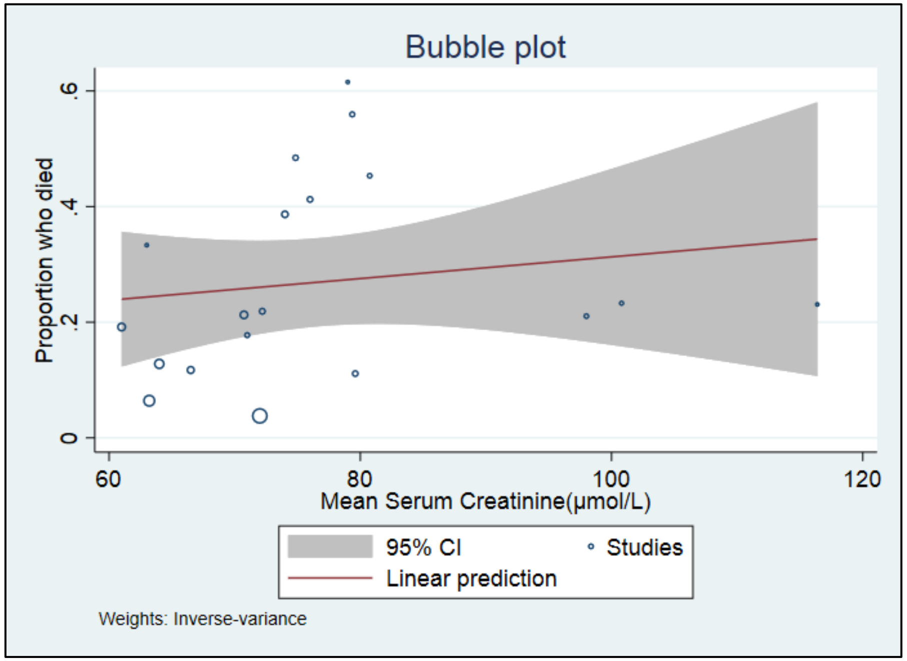

154b

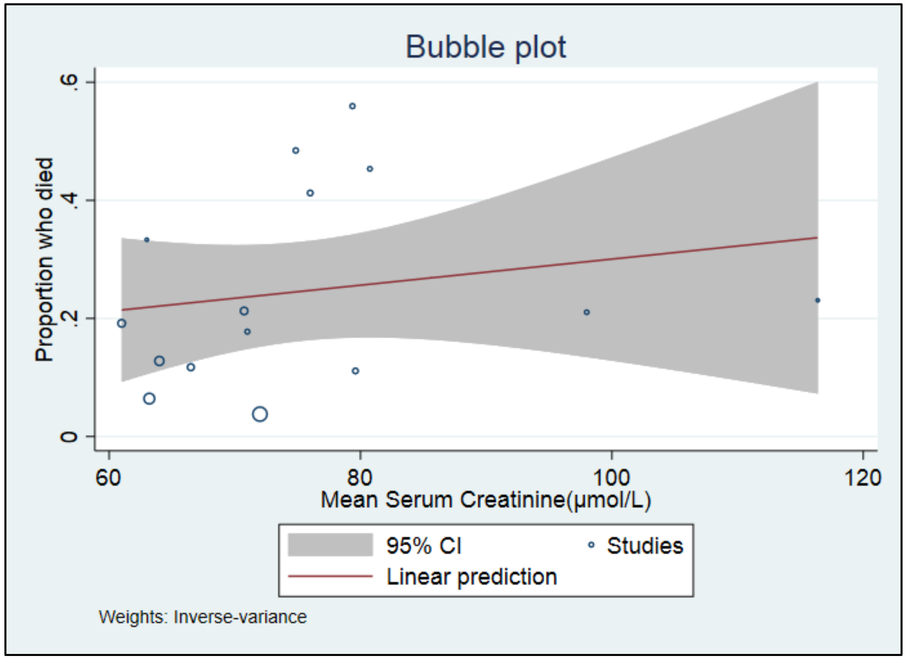

eFigure 155 Percentage change in mortality with unit increase in mean BUN

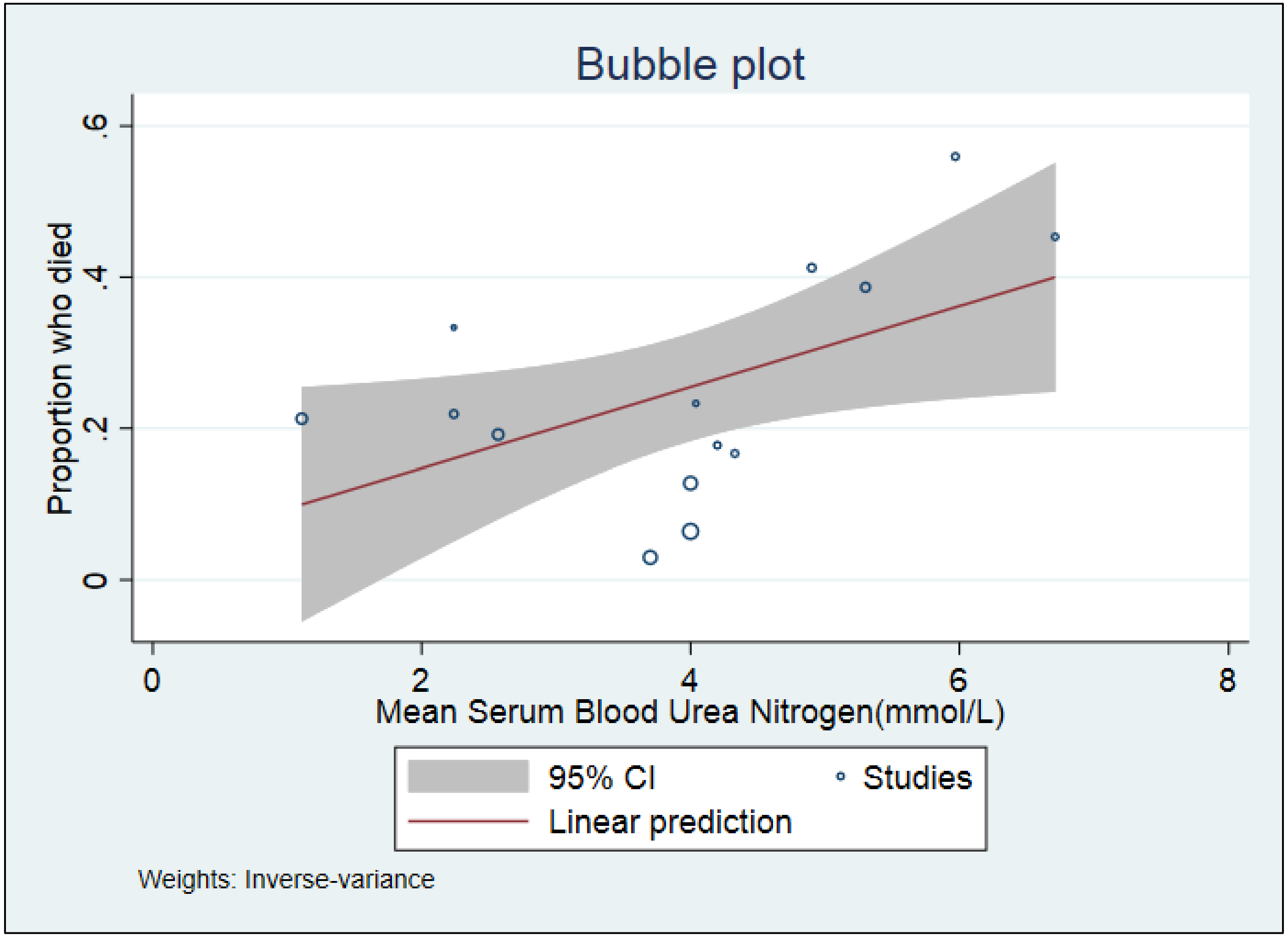

eFigure 156 Percentage change in mortality with unit increase in mean total bilirubin

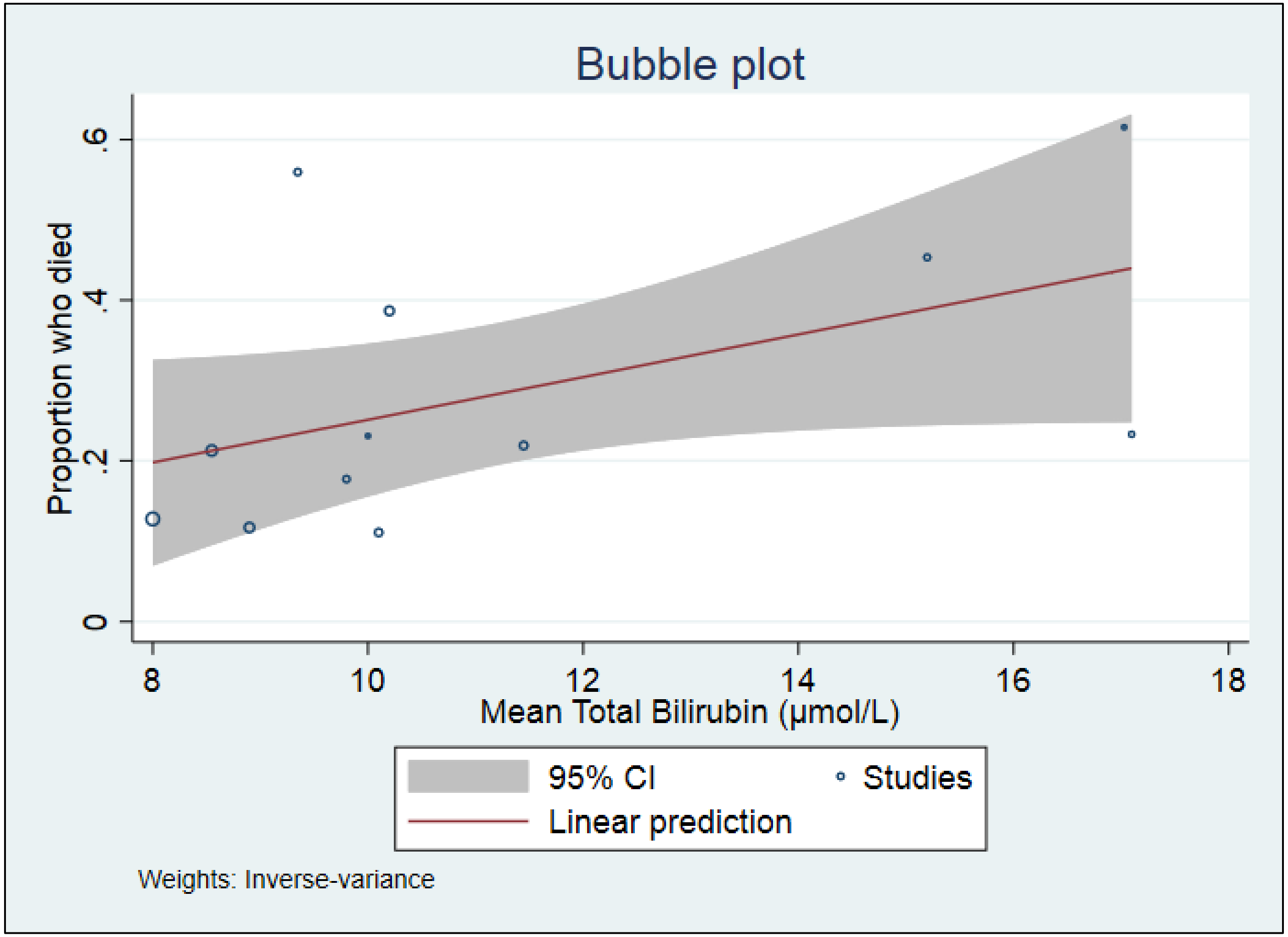

eFigure 157 Percentage change in mortality with unit increase in mean albumin

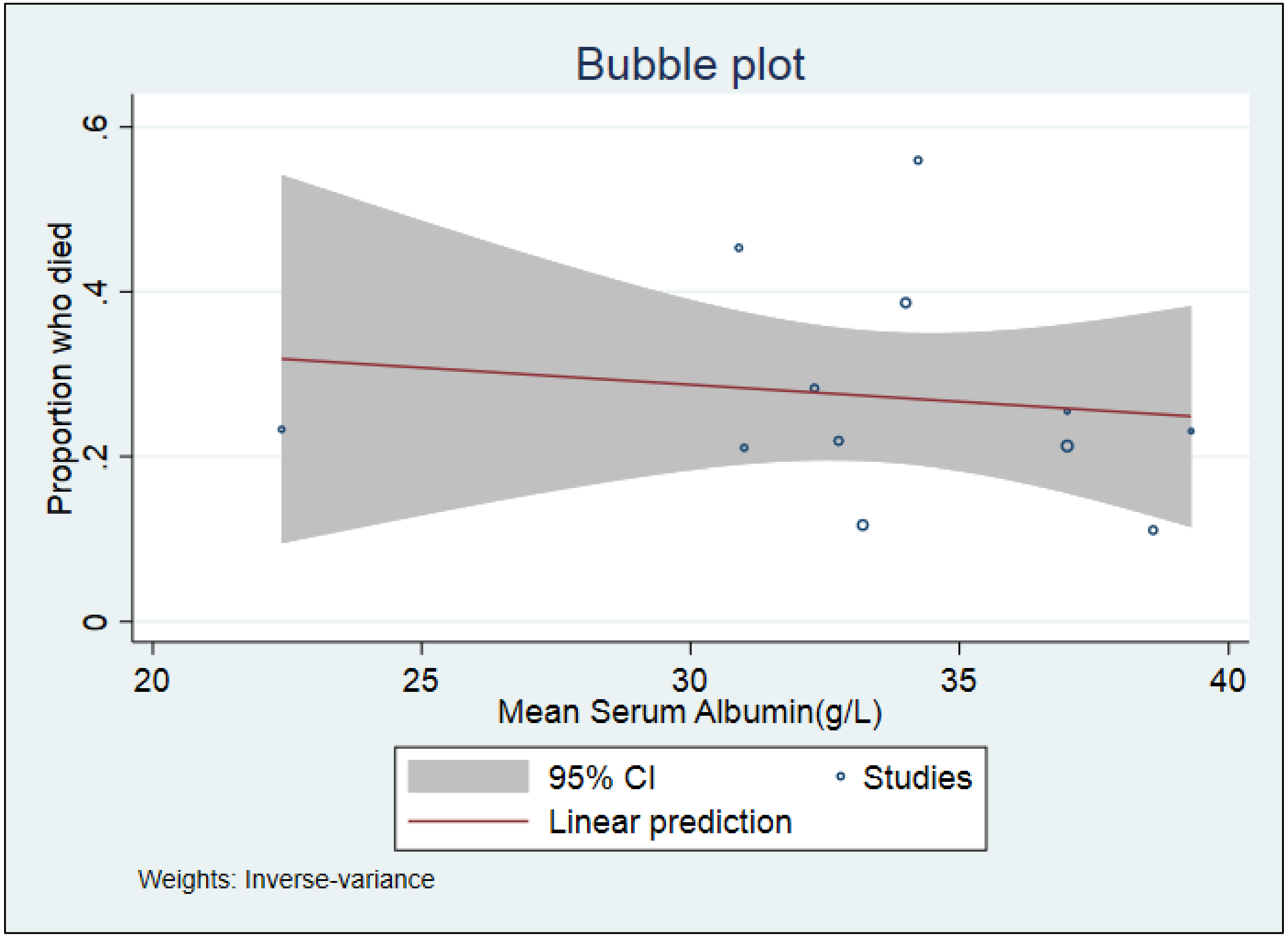

eFigure 158 Percentage change in mortality with unit increase in mean AST

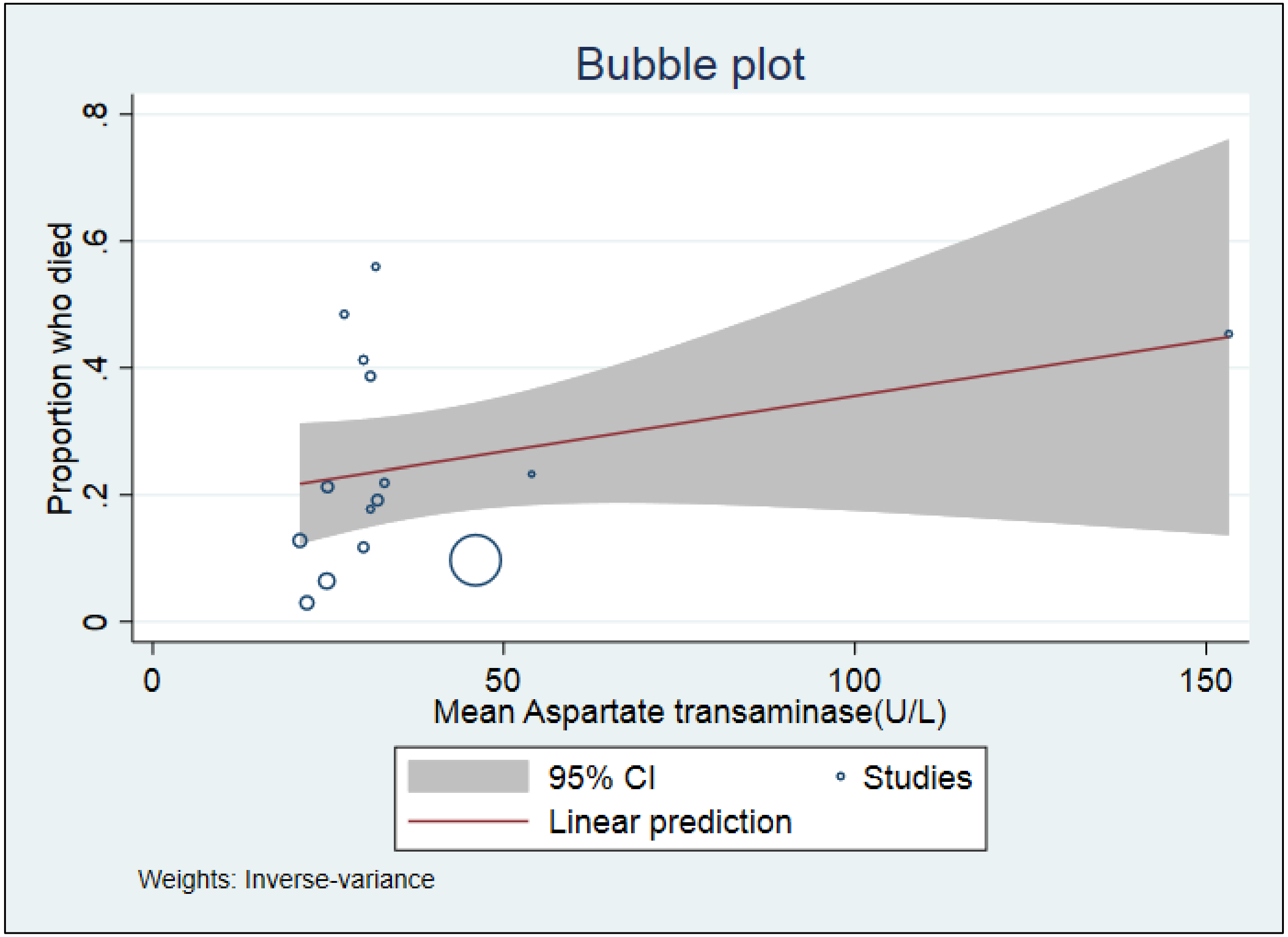

eFigure 159 Percentage change in mortality with unit increase in mean ALT

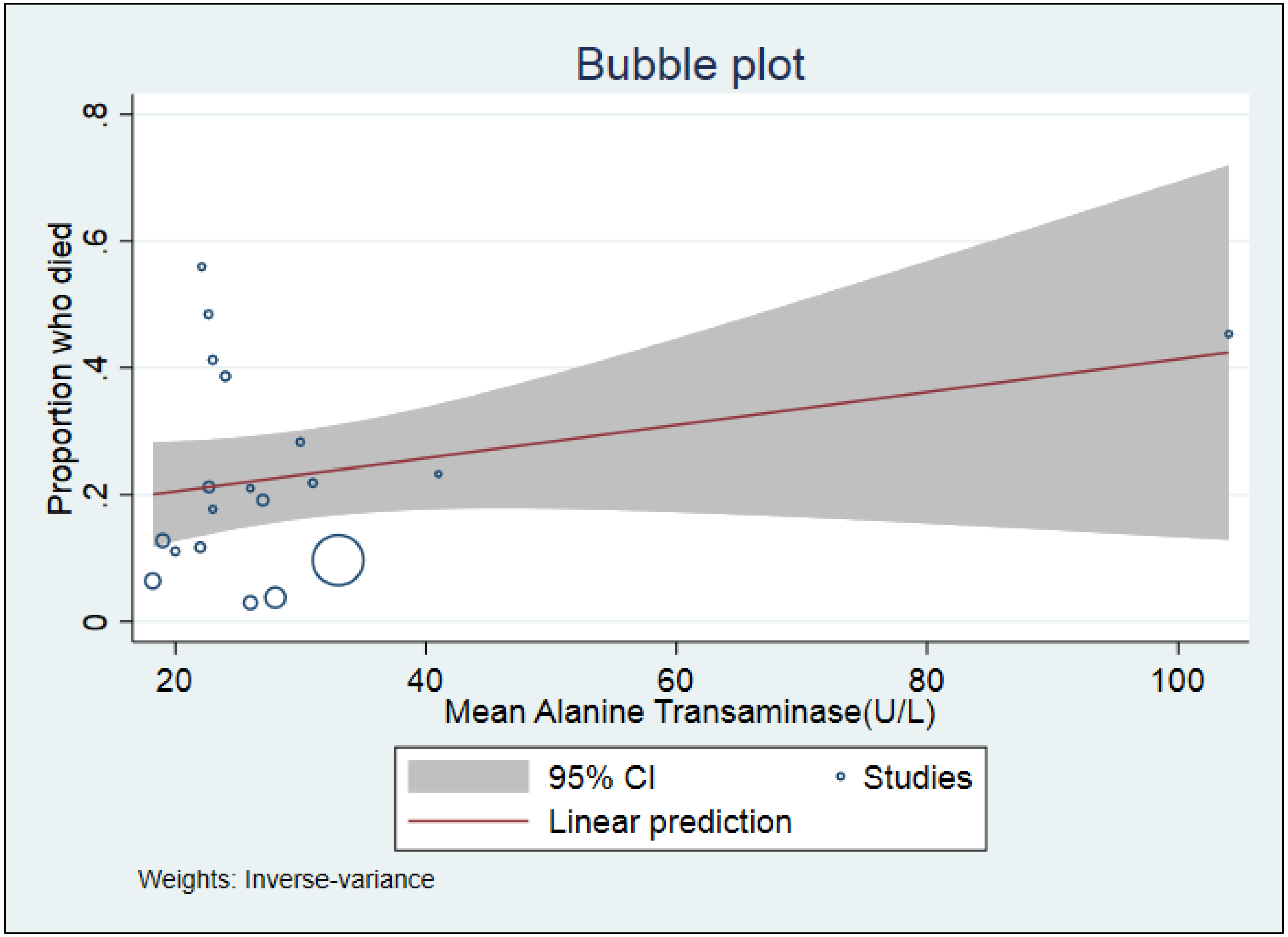

eFigure 160 Percentage change in mortality with unit increase in mean CK Total

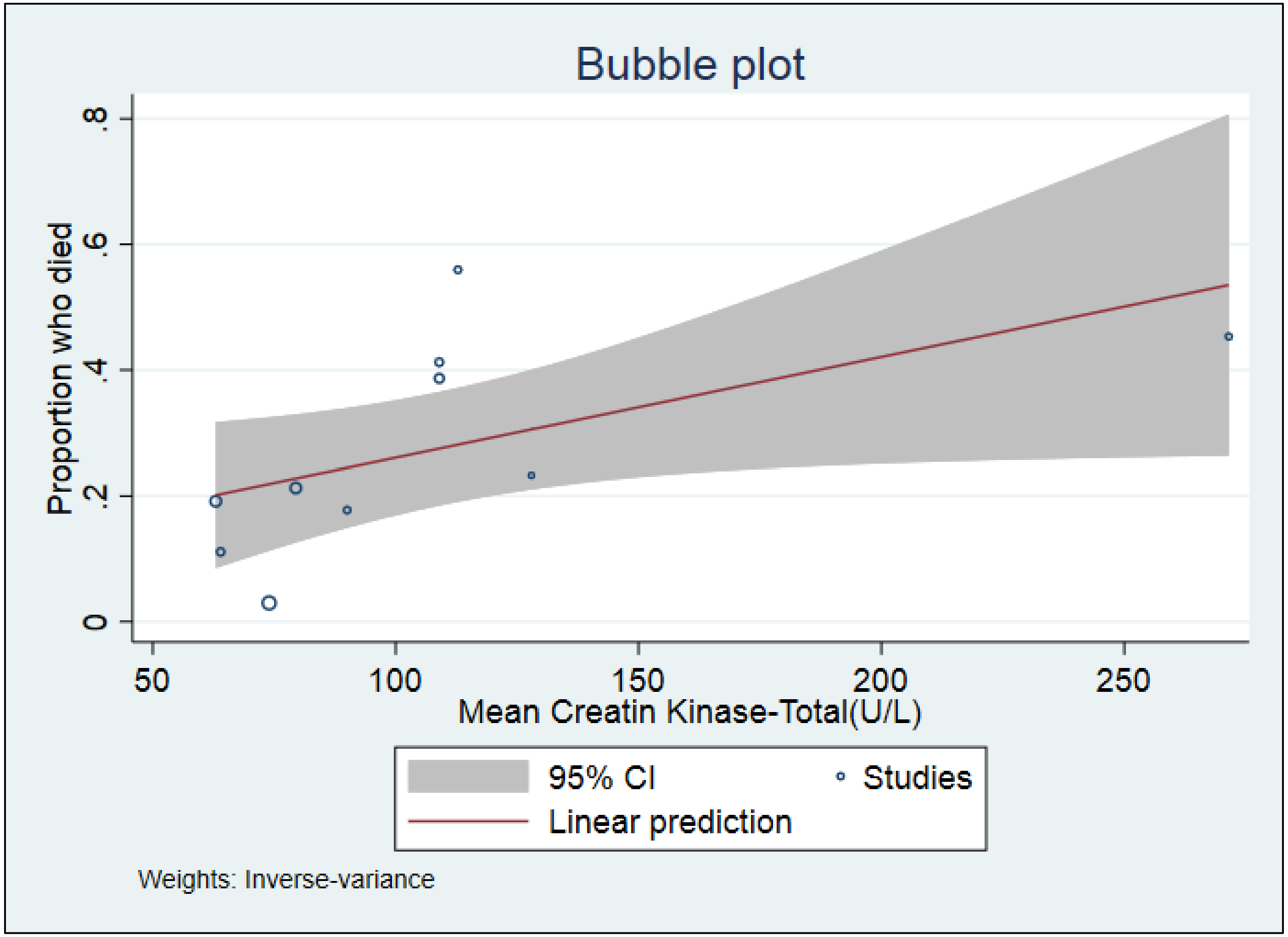

eFigure 161 Percentage change in mortality with unit increase in mean IL6

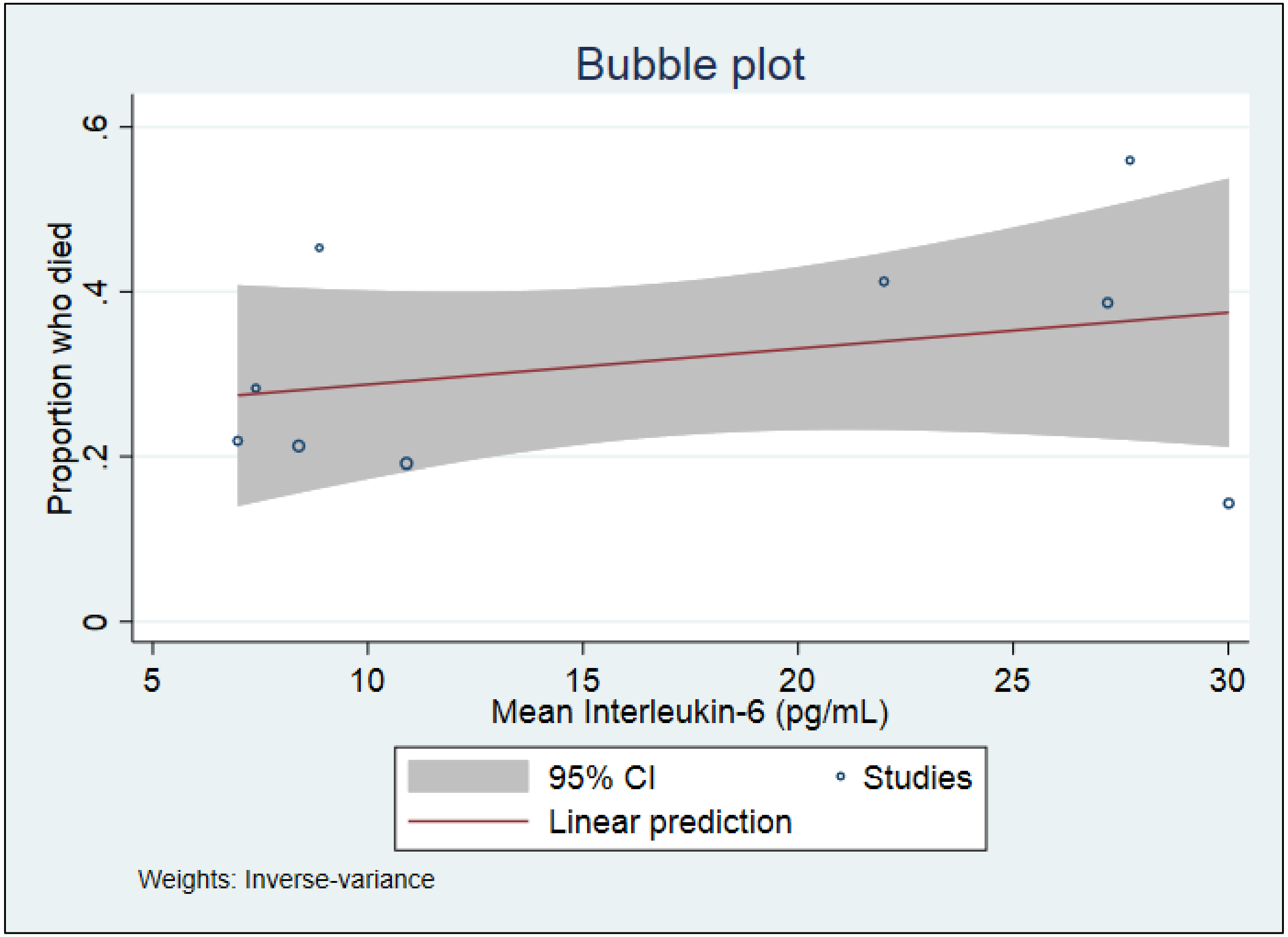

eFigure 162 Percentage change in severe disease with unit increase in mean age

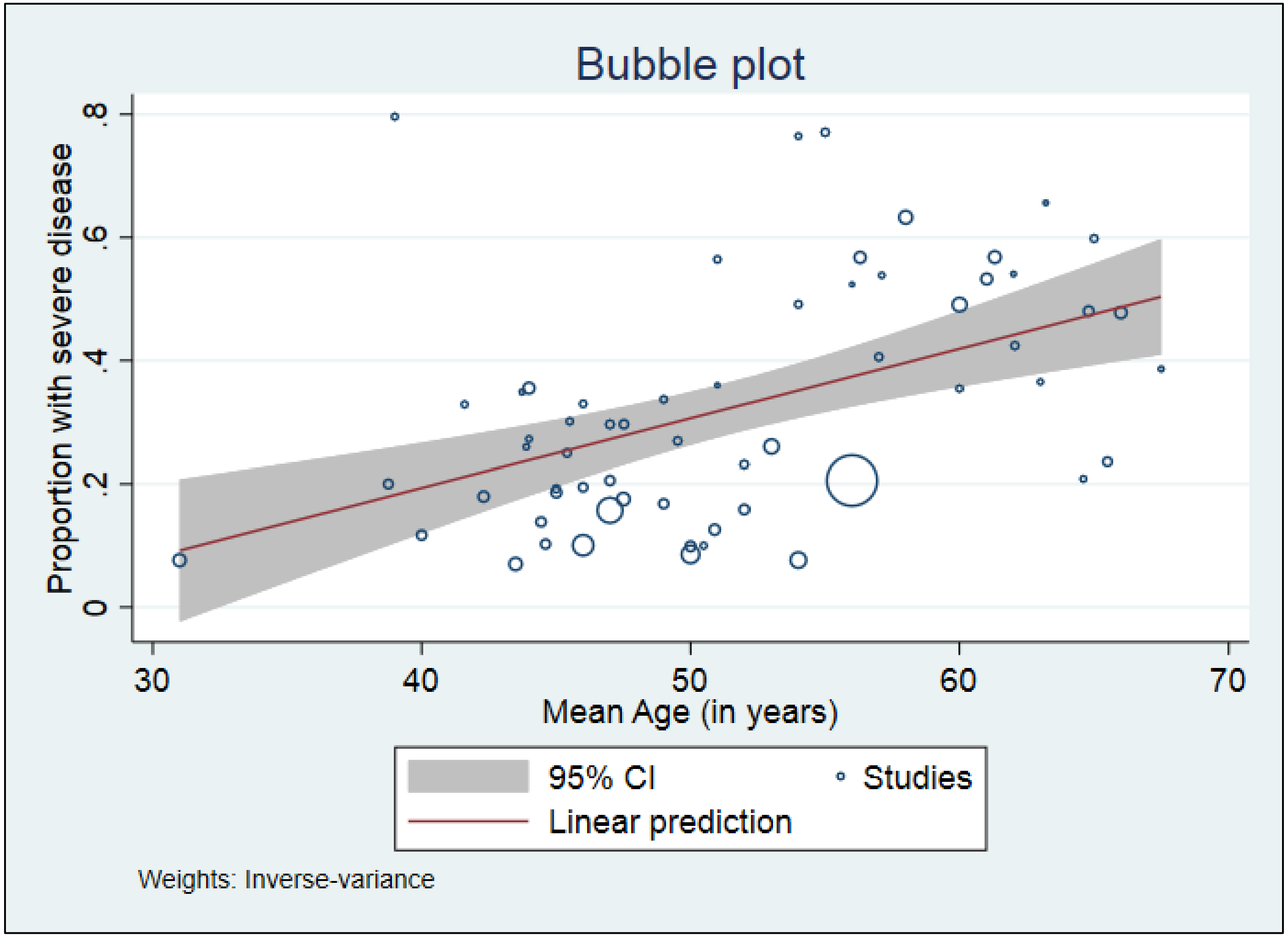

eFigure 163 Percentage change in severe disease with unit increase in mean Hb

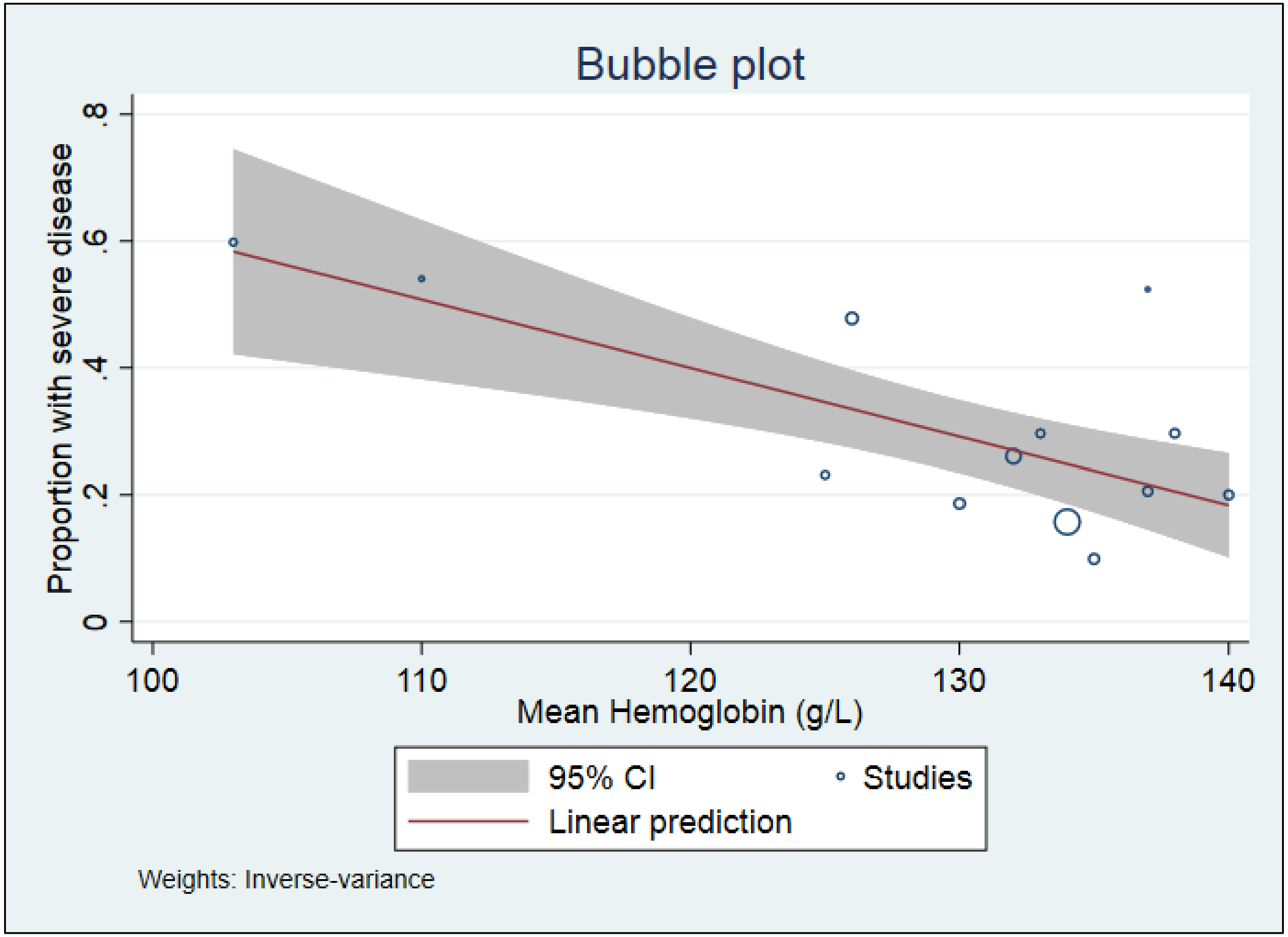

eFigure 164 Percentage change in severe disease with unit increase in mean Total Leukocyte count

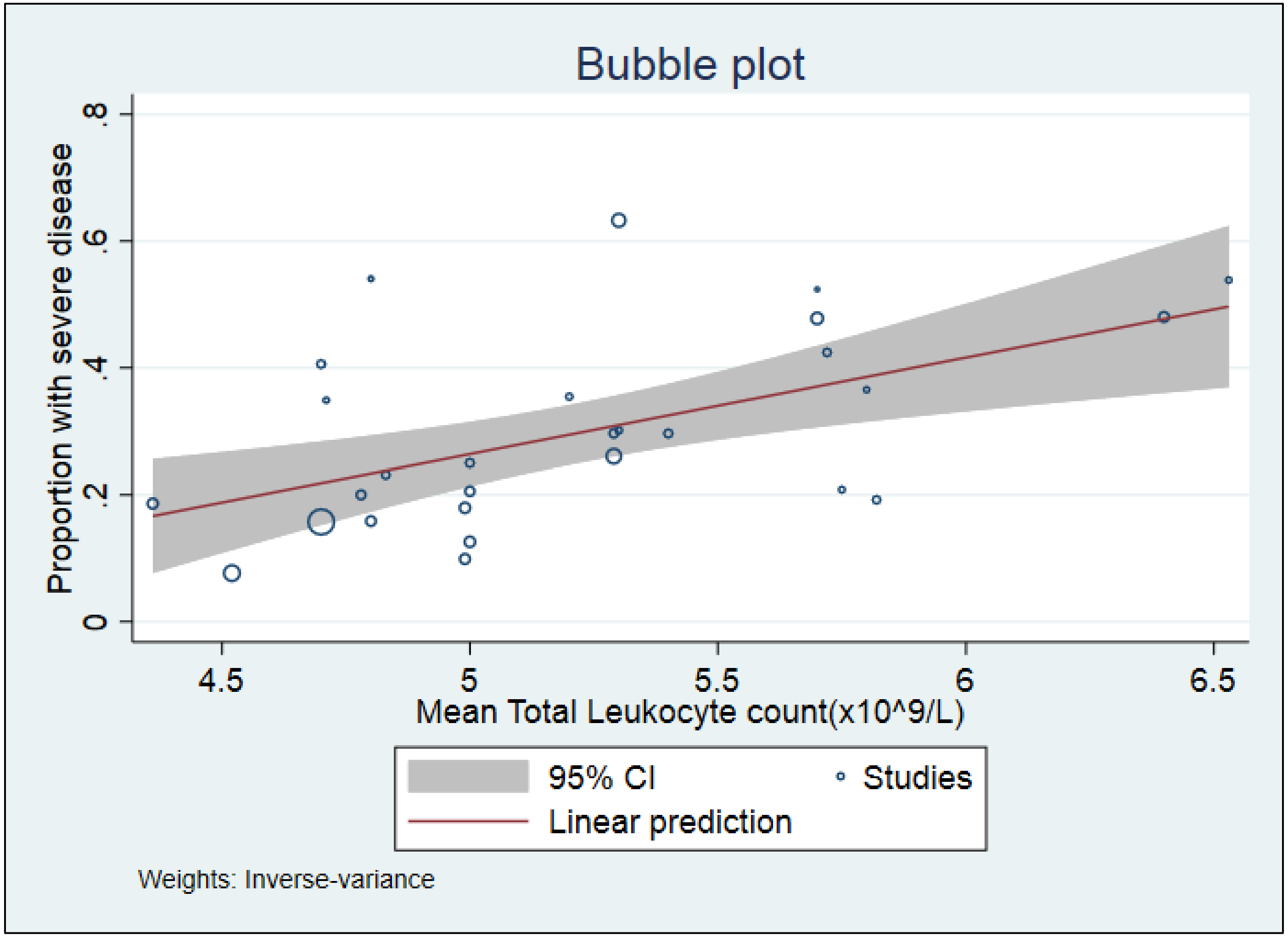

eFigure 165 Percentage change in severe disease with unit increase in mean Lymphocyte count

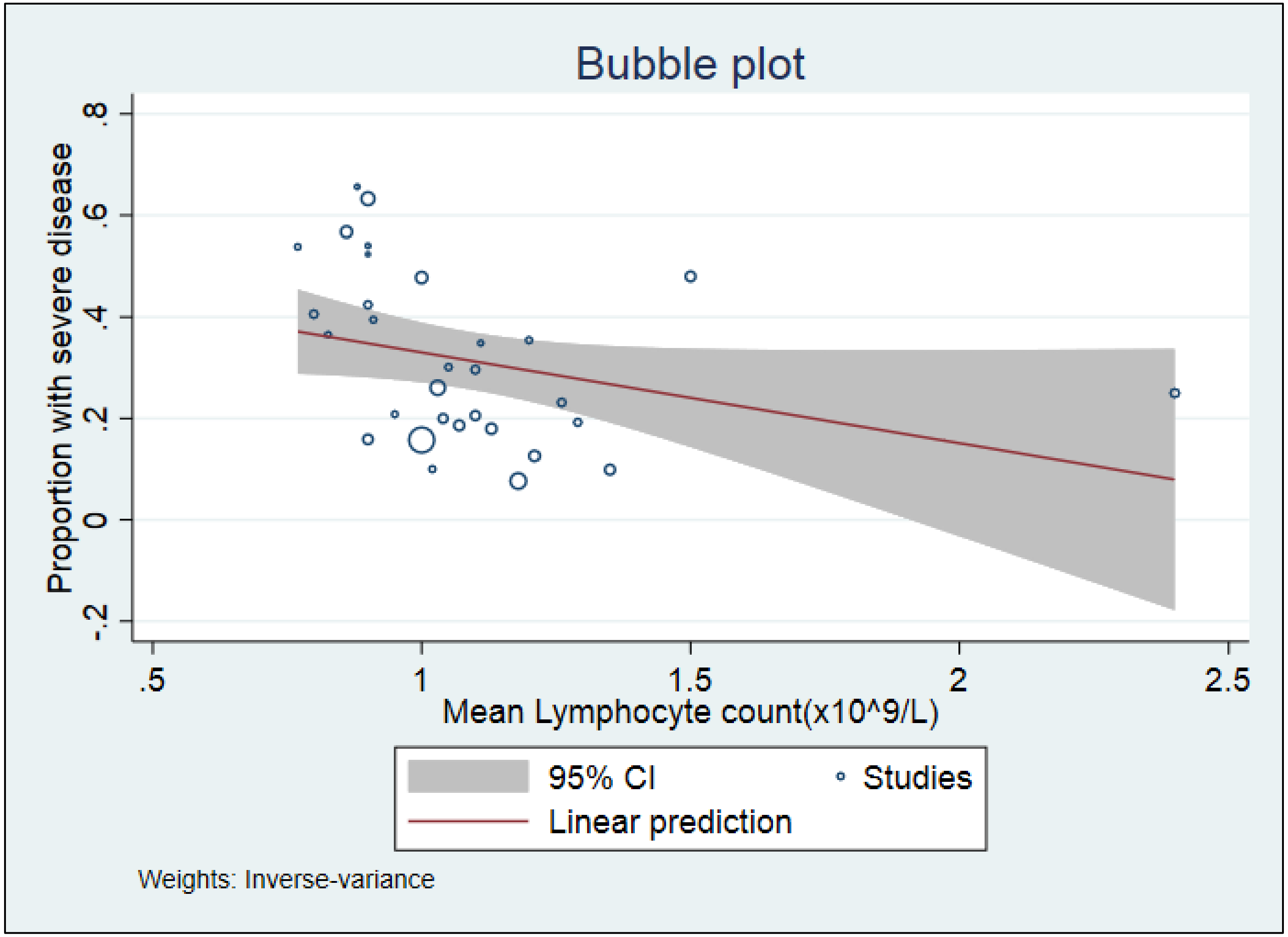

eFigure 166 Percentage change in severe disease with unit increase in mean Neutrophil count

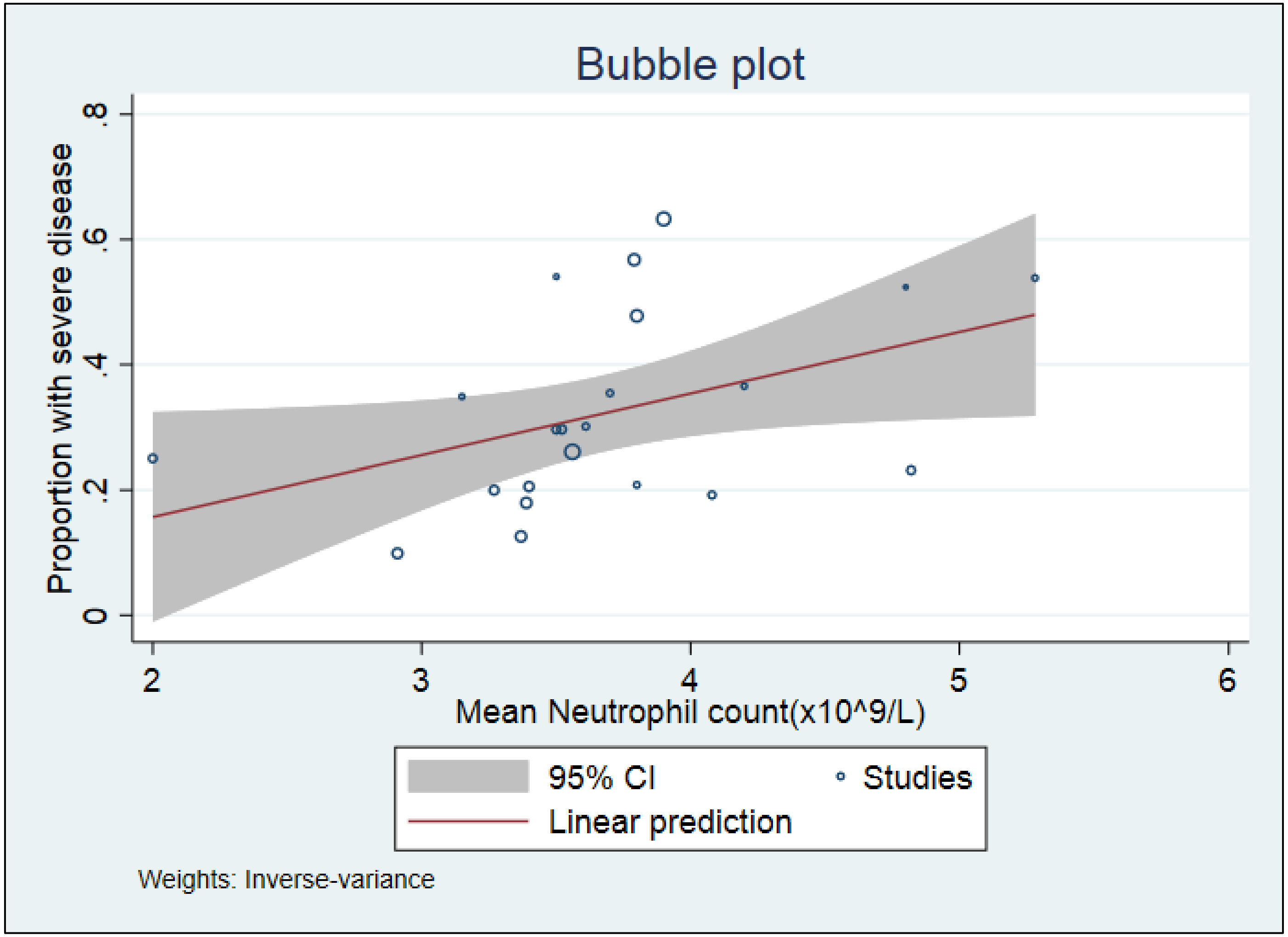

eFigure 167 Percentage change in severe disease with unit increase in mean platelet count

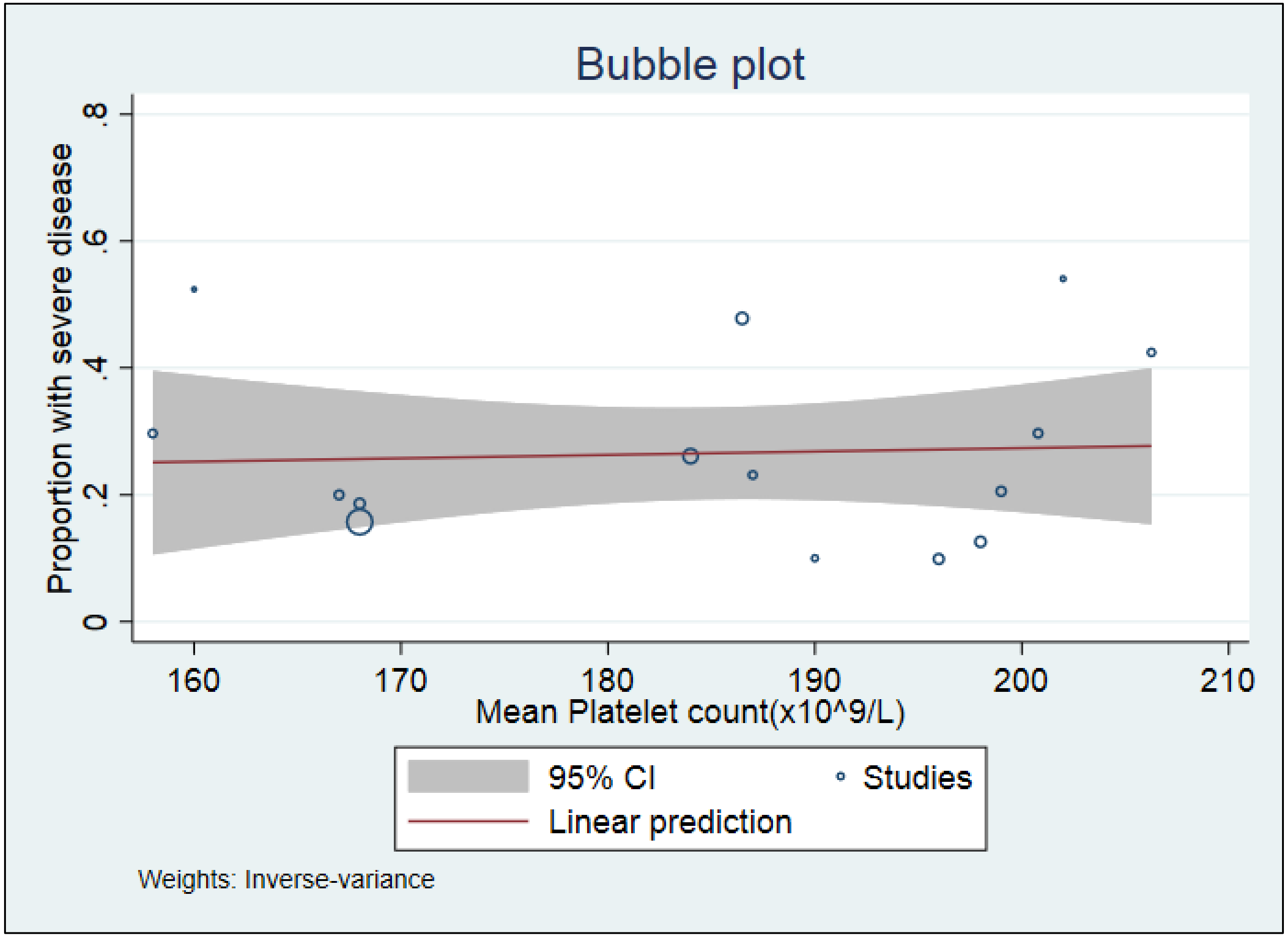

eFigure 168 Percentage change in severe disease with unit increase in mean prothrombin time

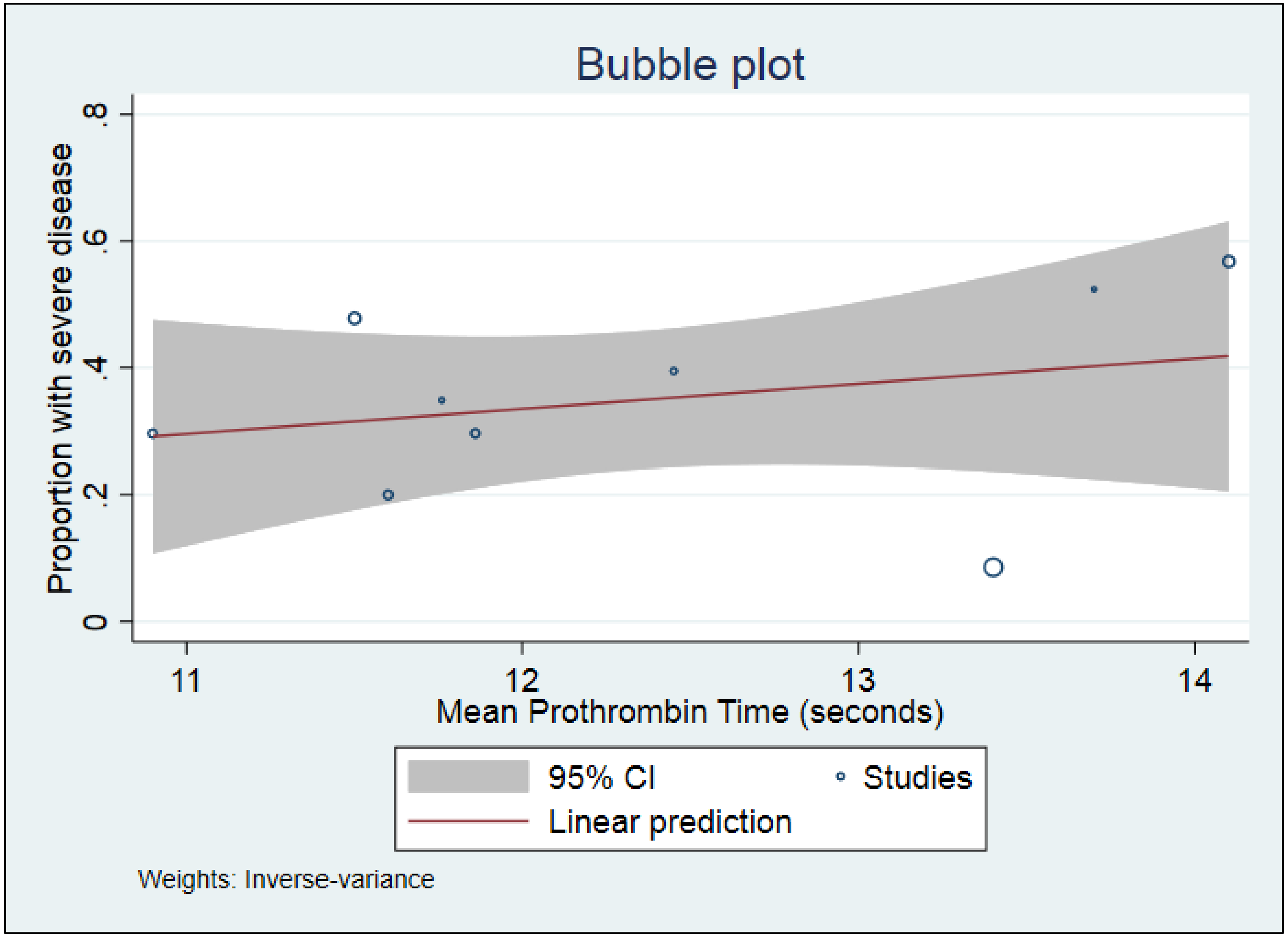

eFigure 169 Percentage change in severe disease with unit increase in mean aPTT

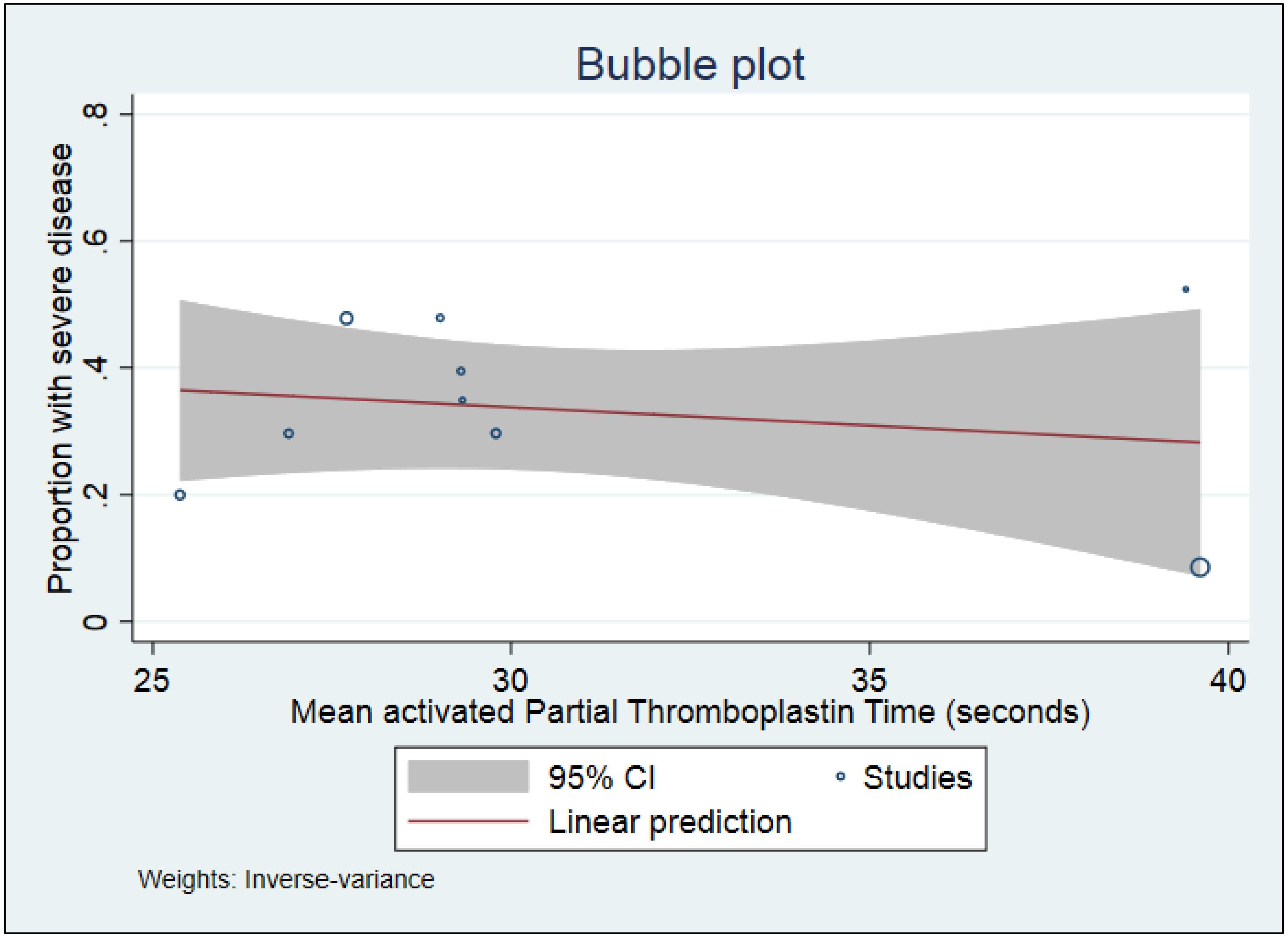

eFigure 170 Percentage change in severe disease with unit increase in mean CRP

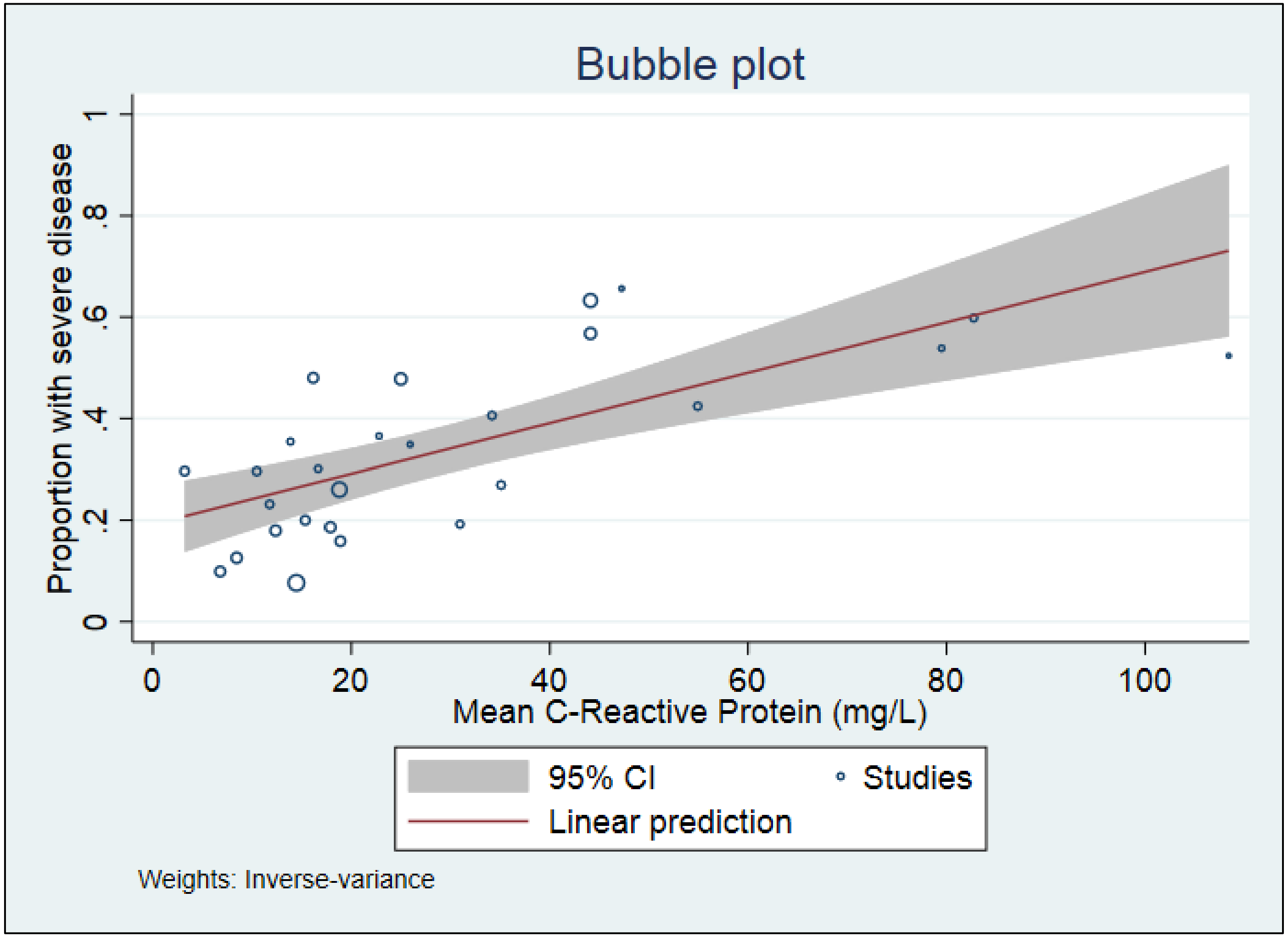

eFigure 171 Percentage change in severe disease with unit increase in mean D Dimer

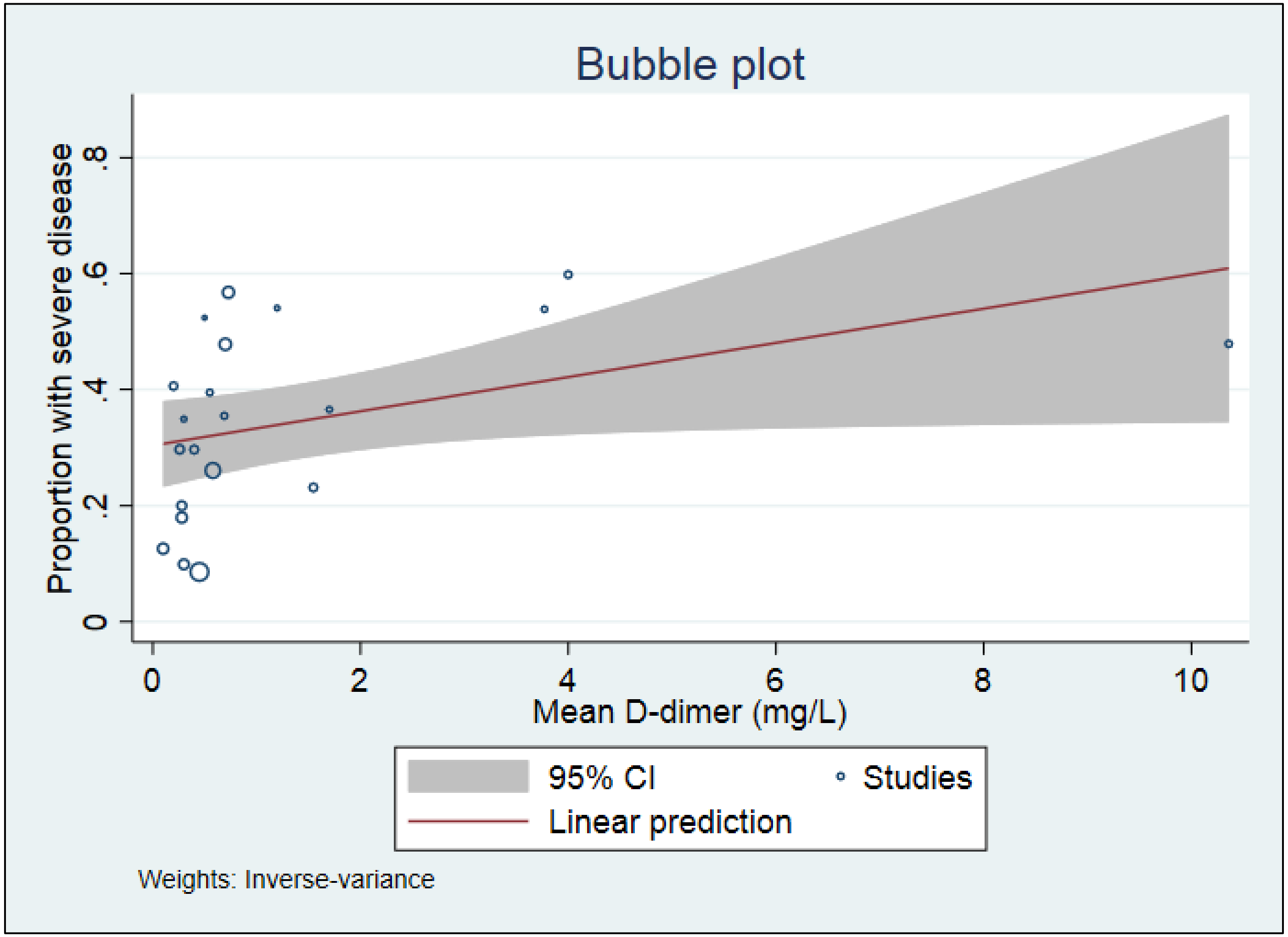

eFigure 172 Percentage change in severe disease with unit increase in mean Pro-calcitonin

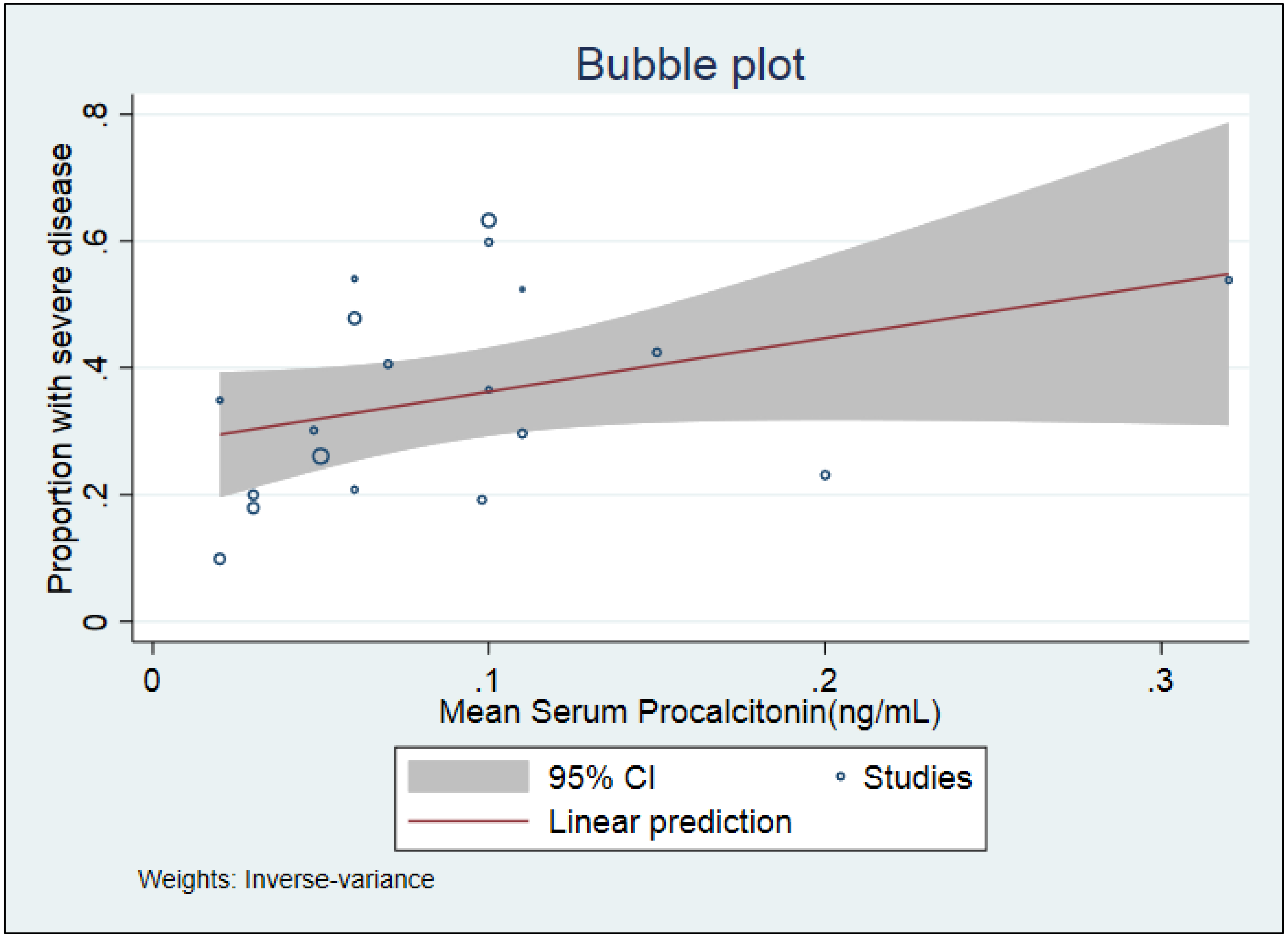

eFigure 173 Percentage change in severe disease with unit increase in mean serum LDH

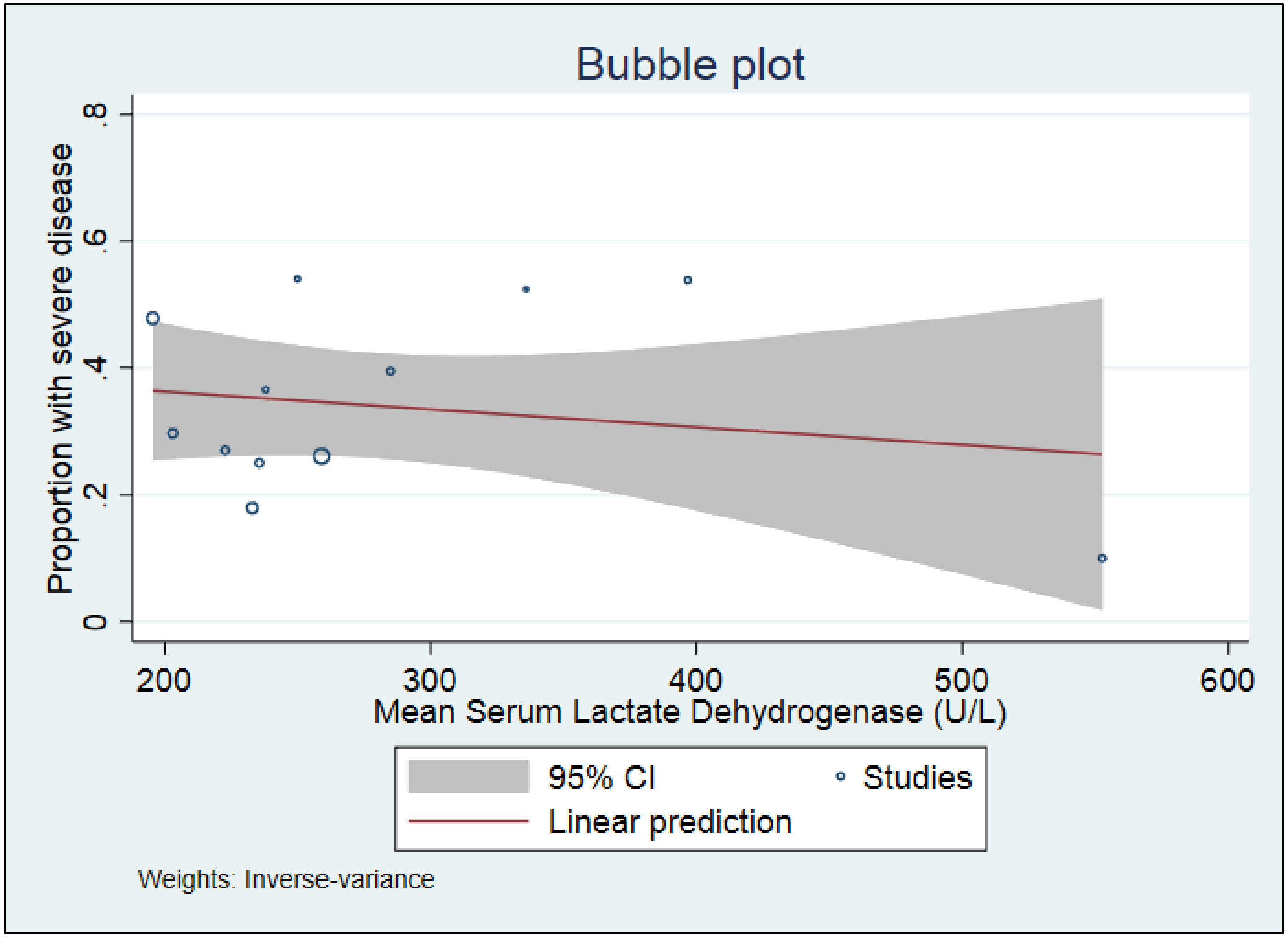

eFigure 174 Percentage change in severe disease with unit increase in mean ESR

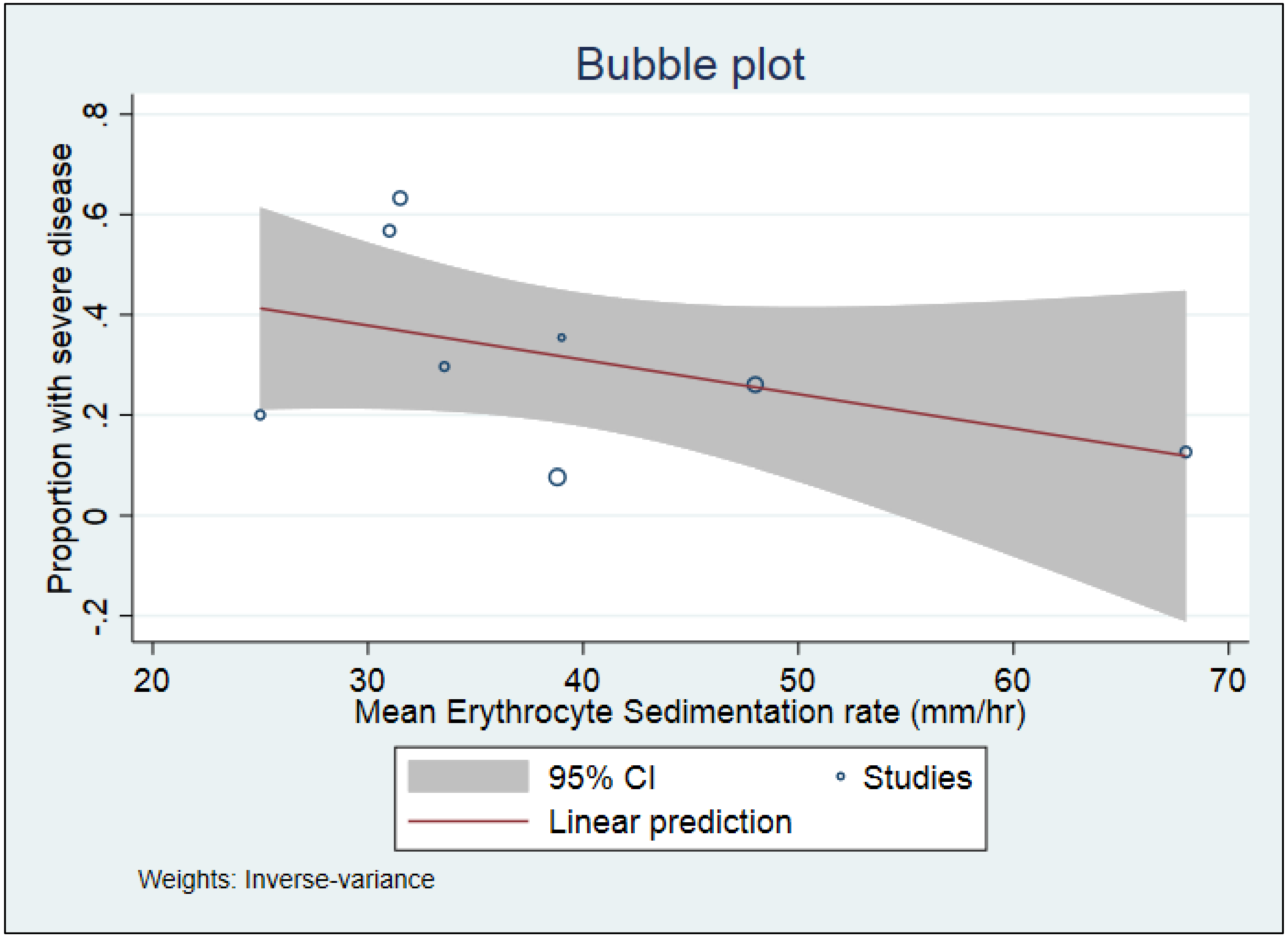

eFigure 175 Percentage change in severe disease with unit increase in mean serum creatinine

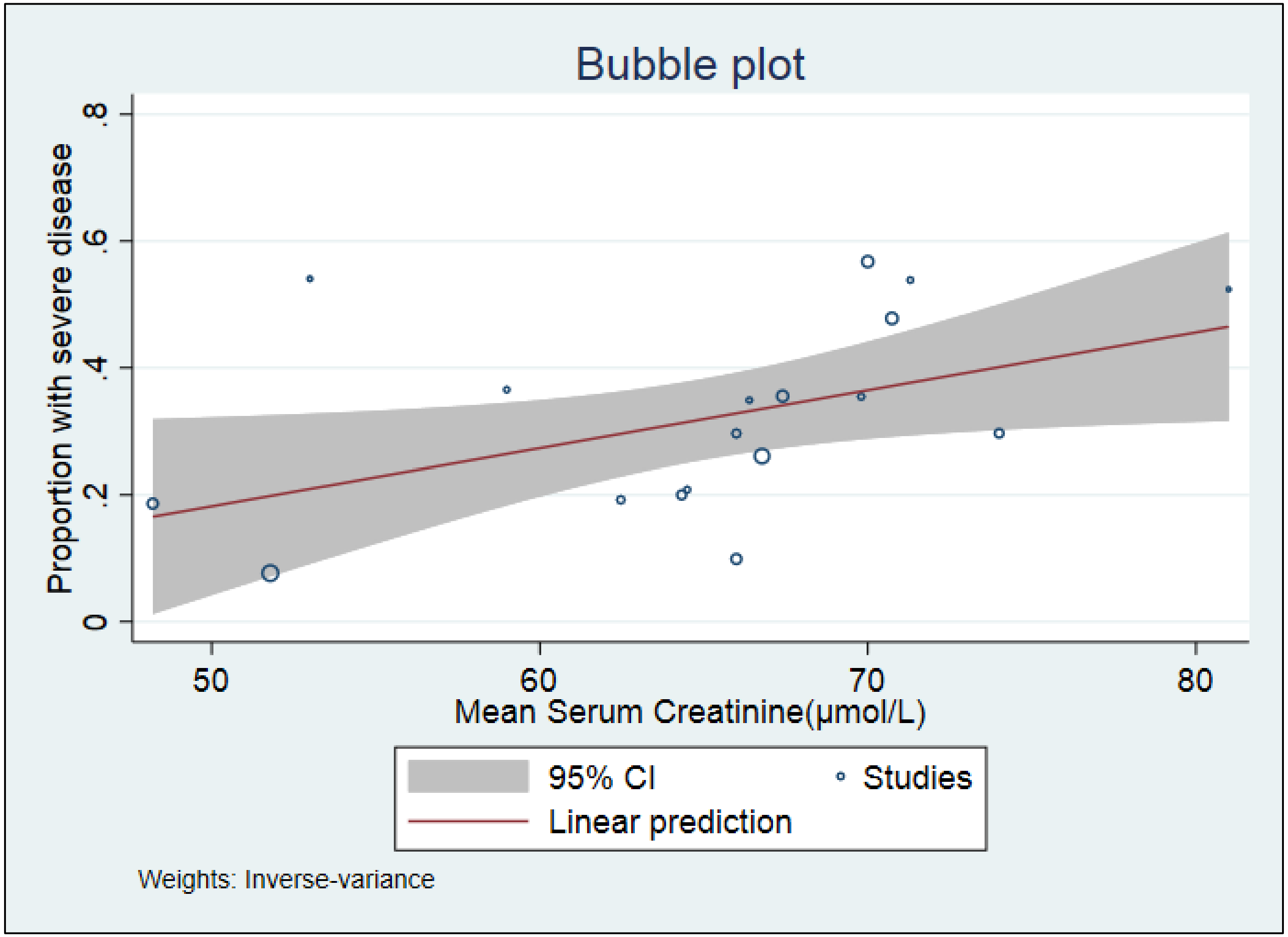

eFigure 176 Percentage change in severe disease with unit increase in mean serum BUN

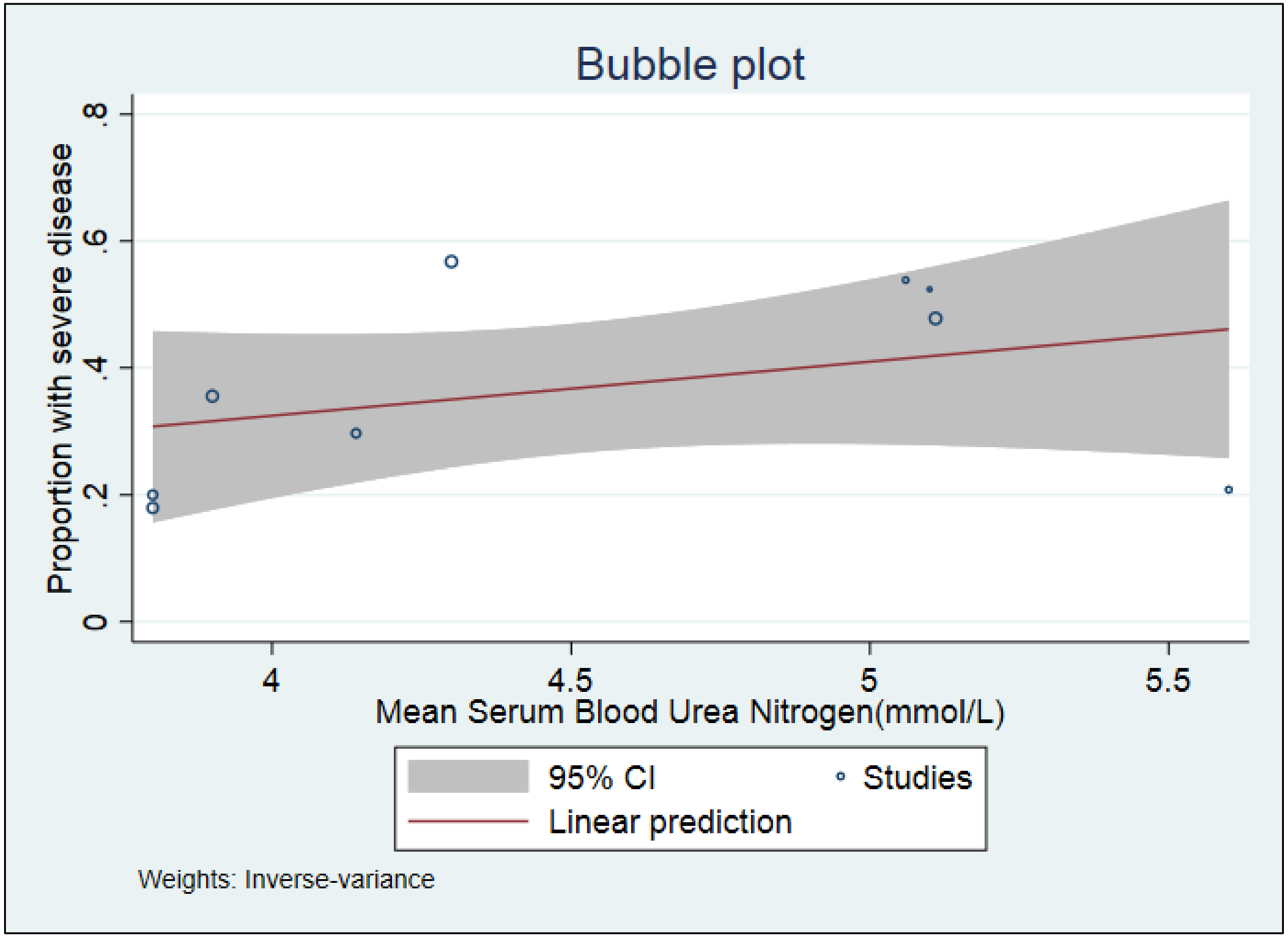

eFigure 178 Percentage change in severe disease with unit increase in mean total bilirubin

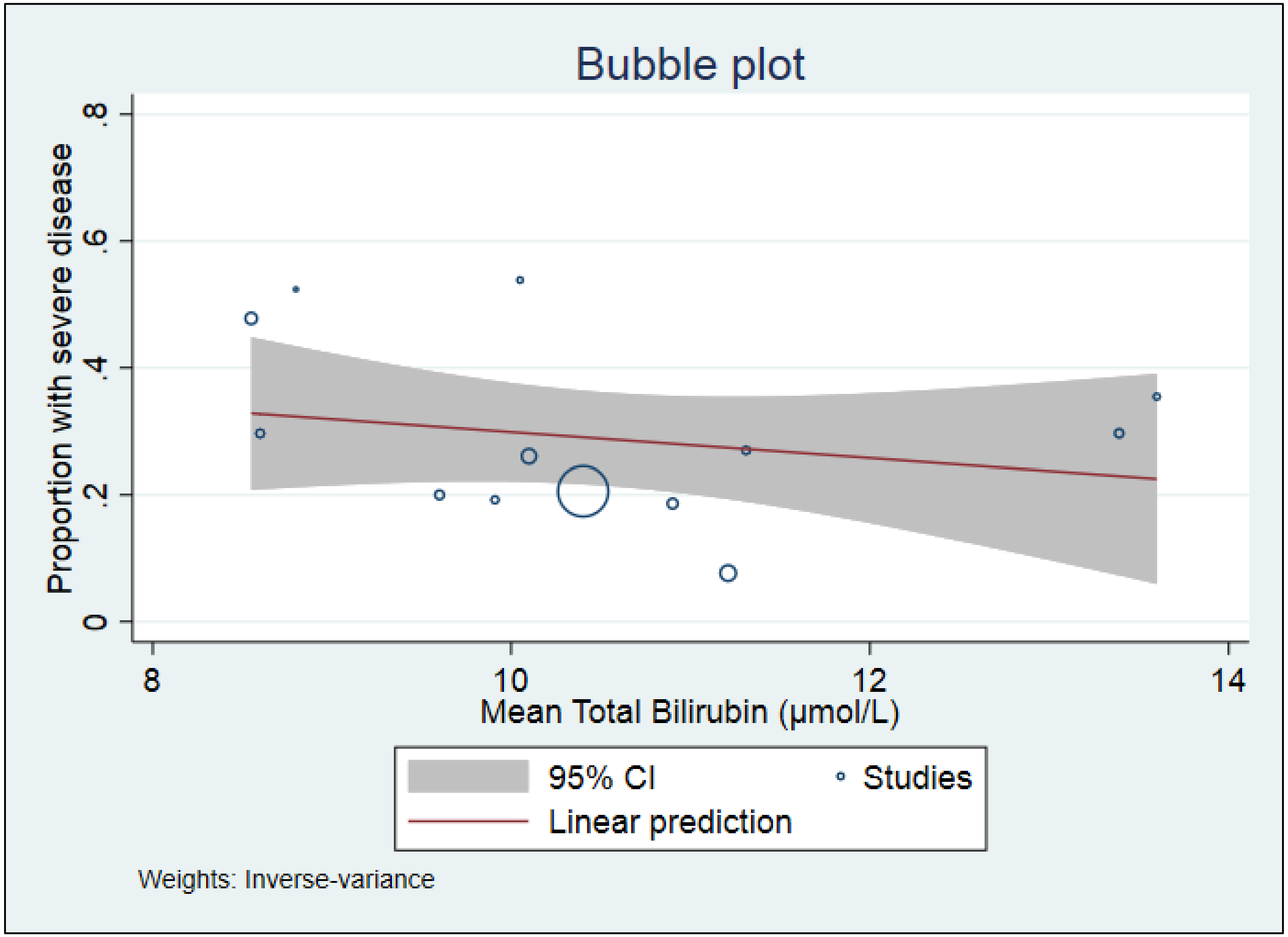

eFigure 179 Percentage change in severe disease with unit increase in mean serum albumin

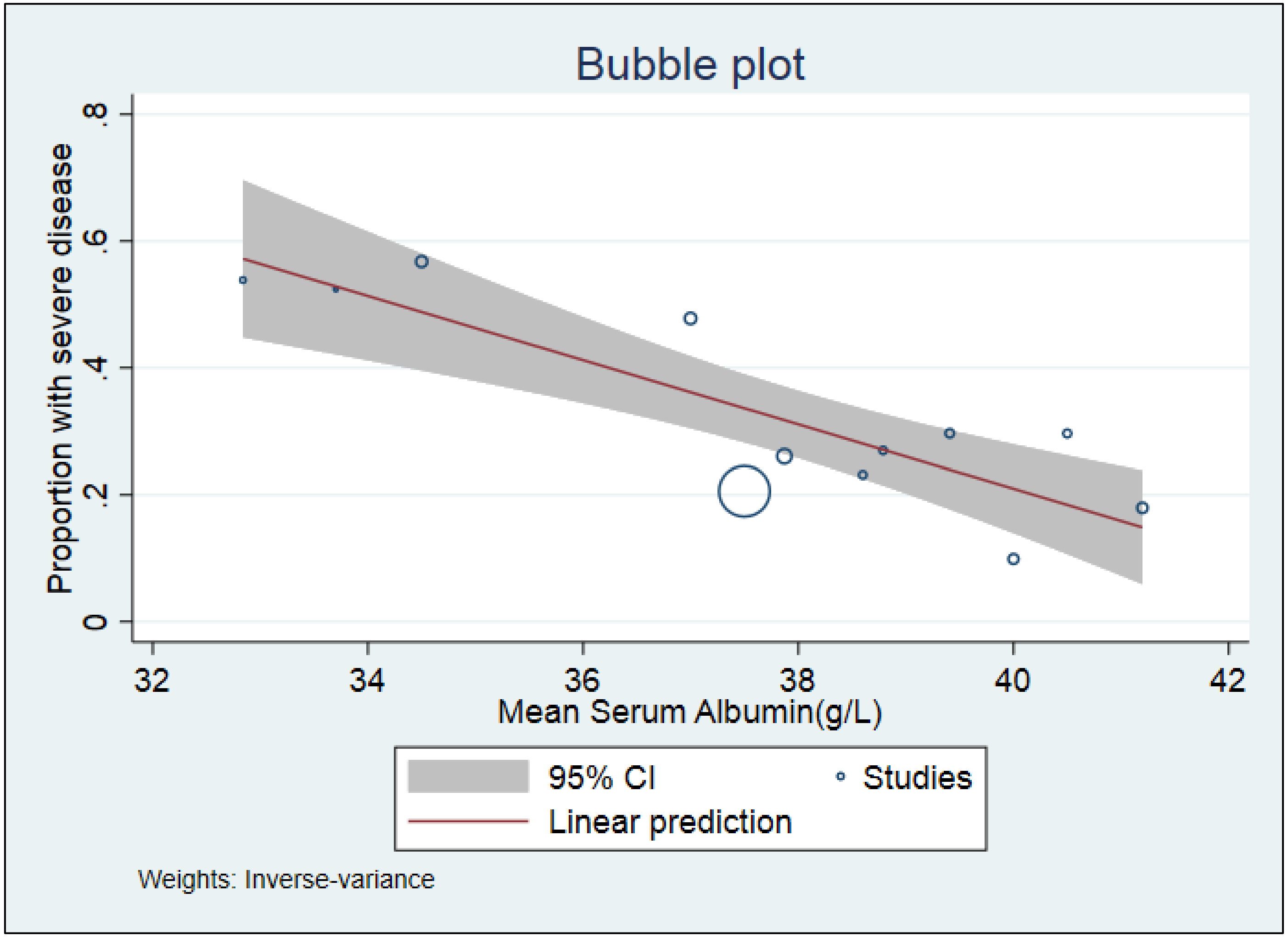

eFigure 180 Percentage change in severe disease with unit increase in mean AST

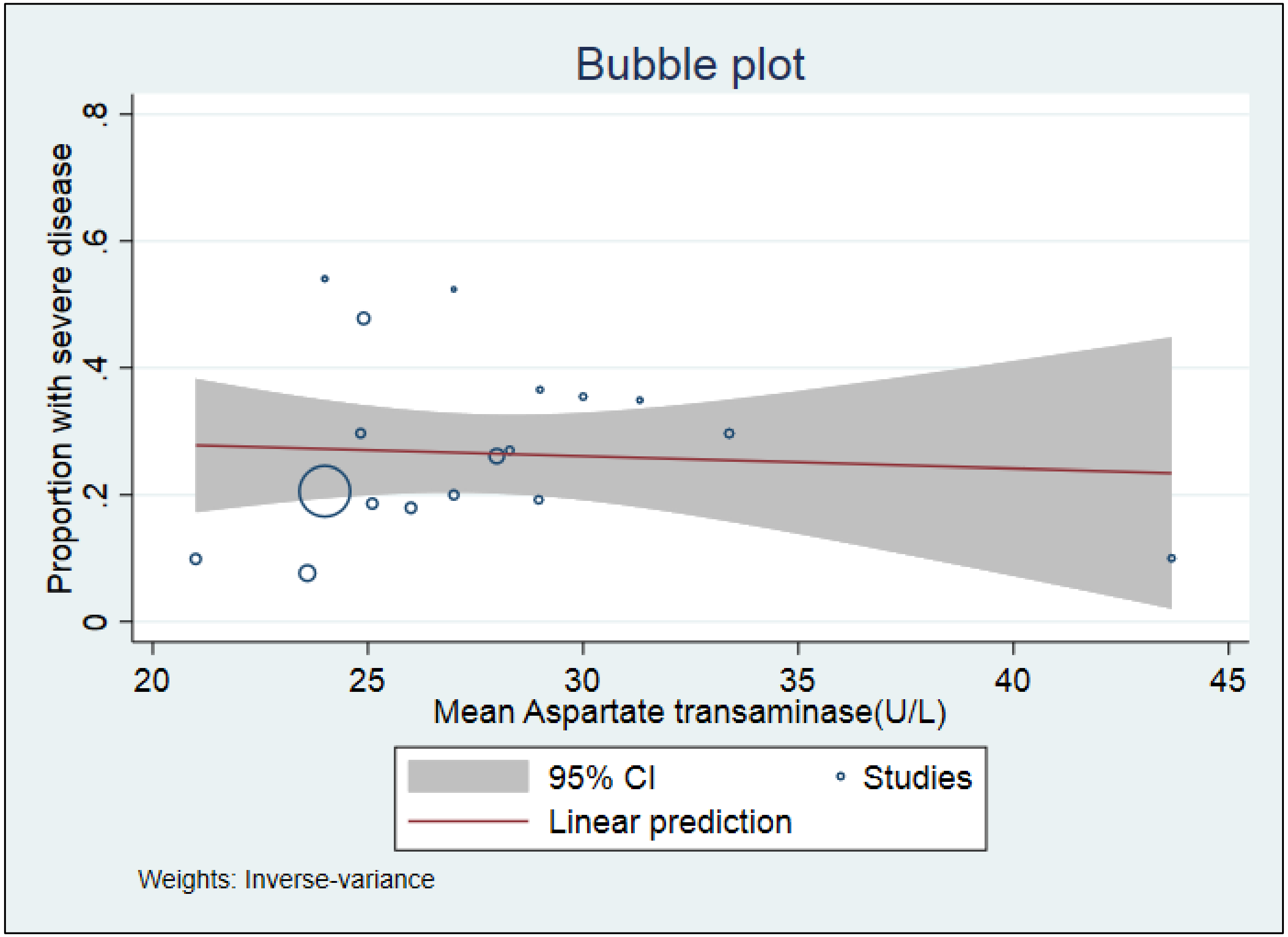

eFigure 181 Percentage change in severe disease with unit increase in mean ALT

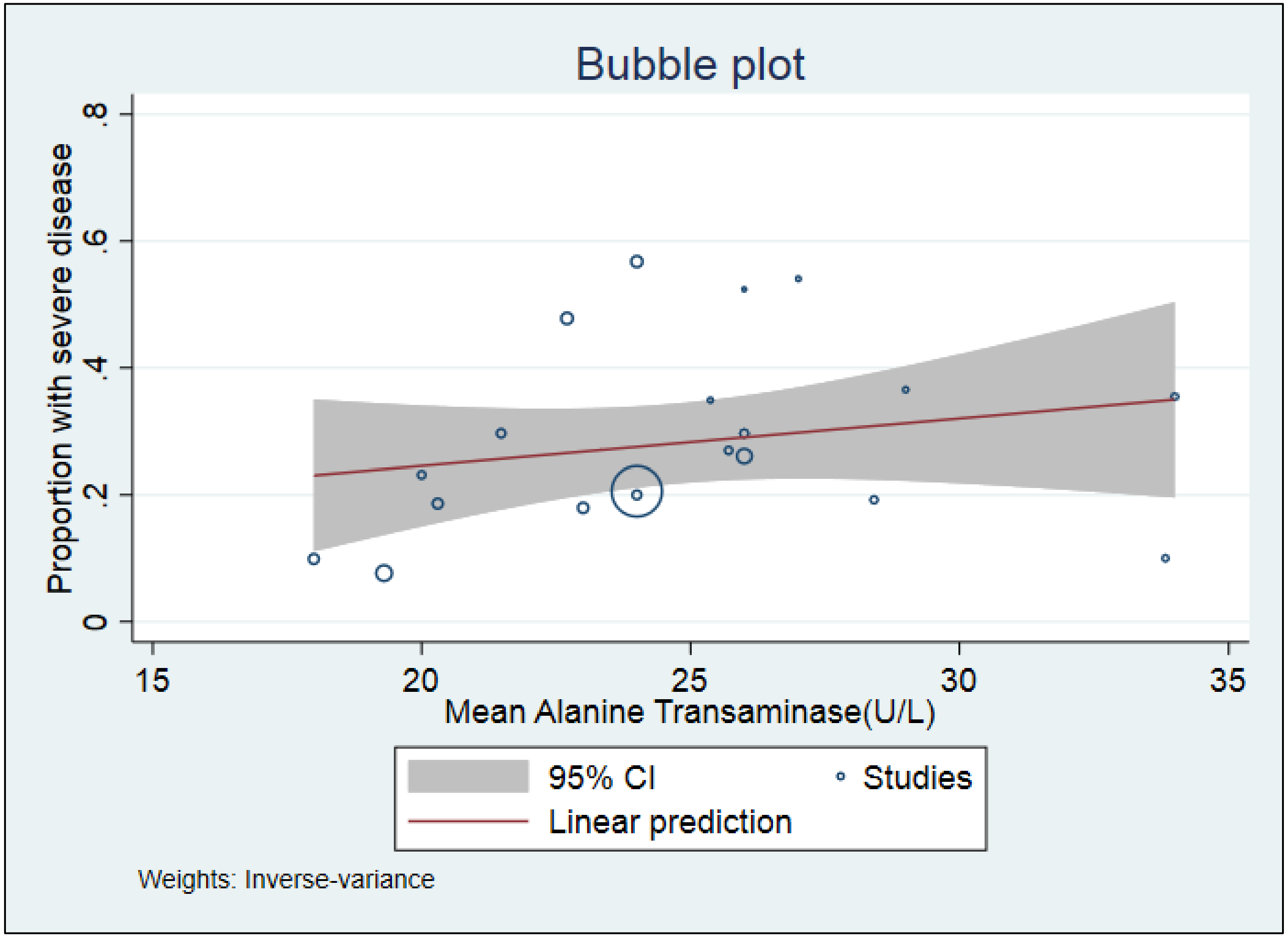

ALT

eFigure 182 Percentage change in severe disease with unit increase in mean Fibrinogen

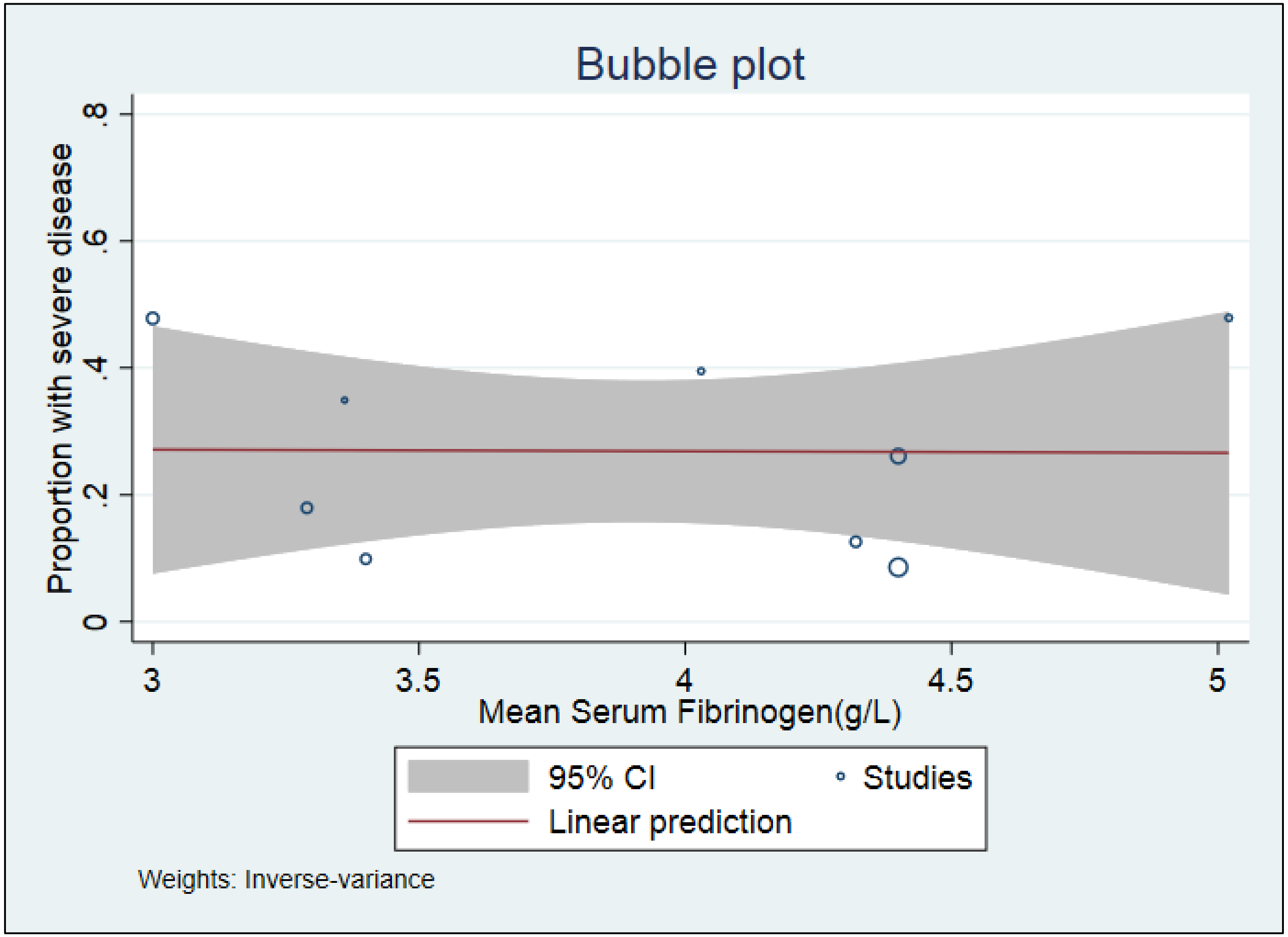

eFigure 183 Percentage change in severe disease with unit increase in mean procalcitonin

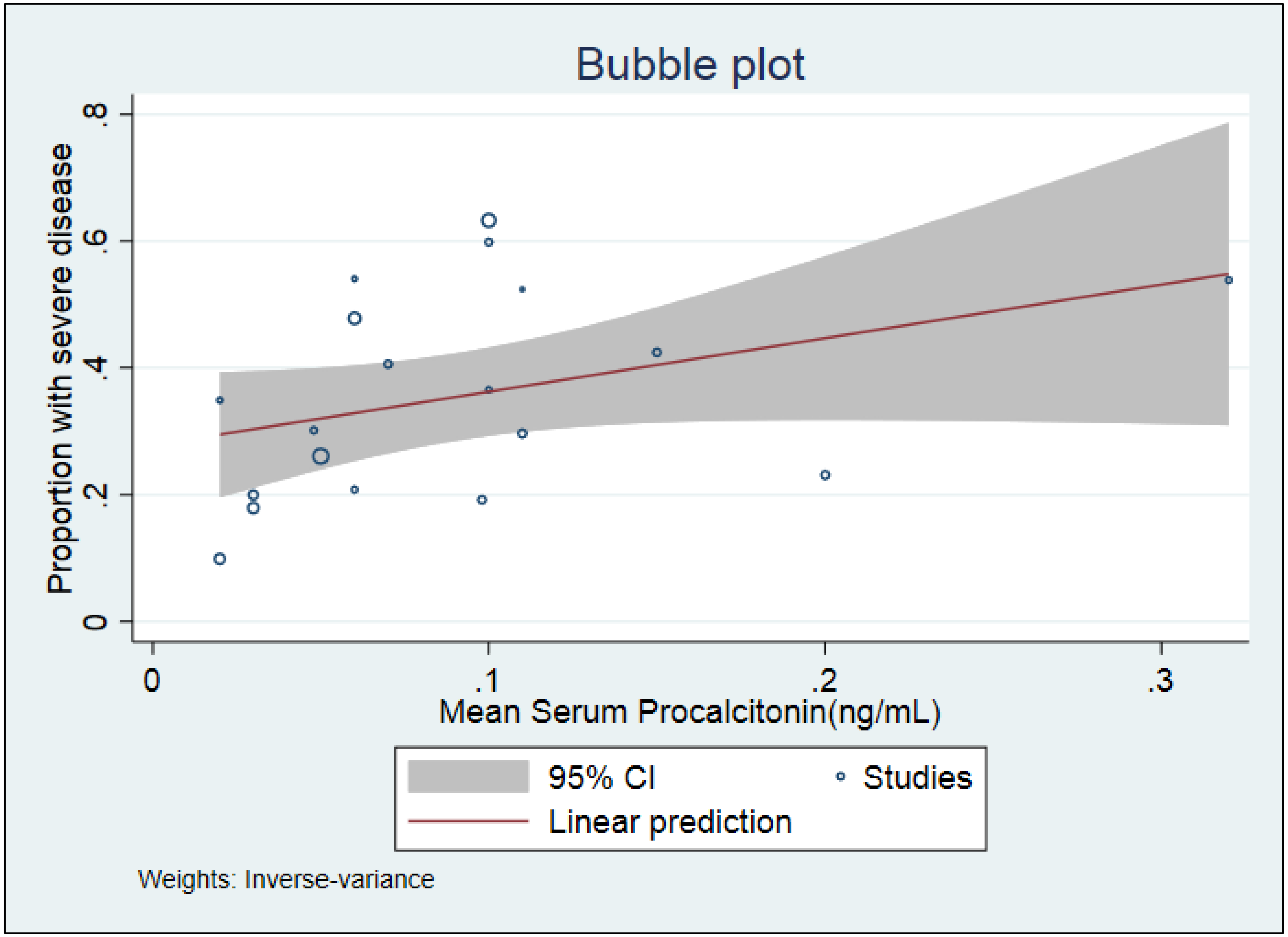

eFigure 184 Percentage change in severe disease with unit increase in mean CK total

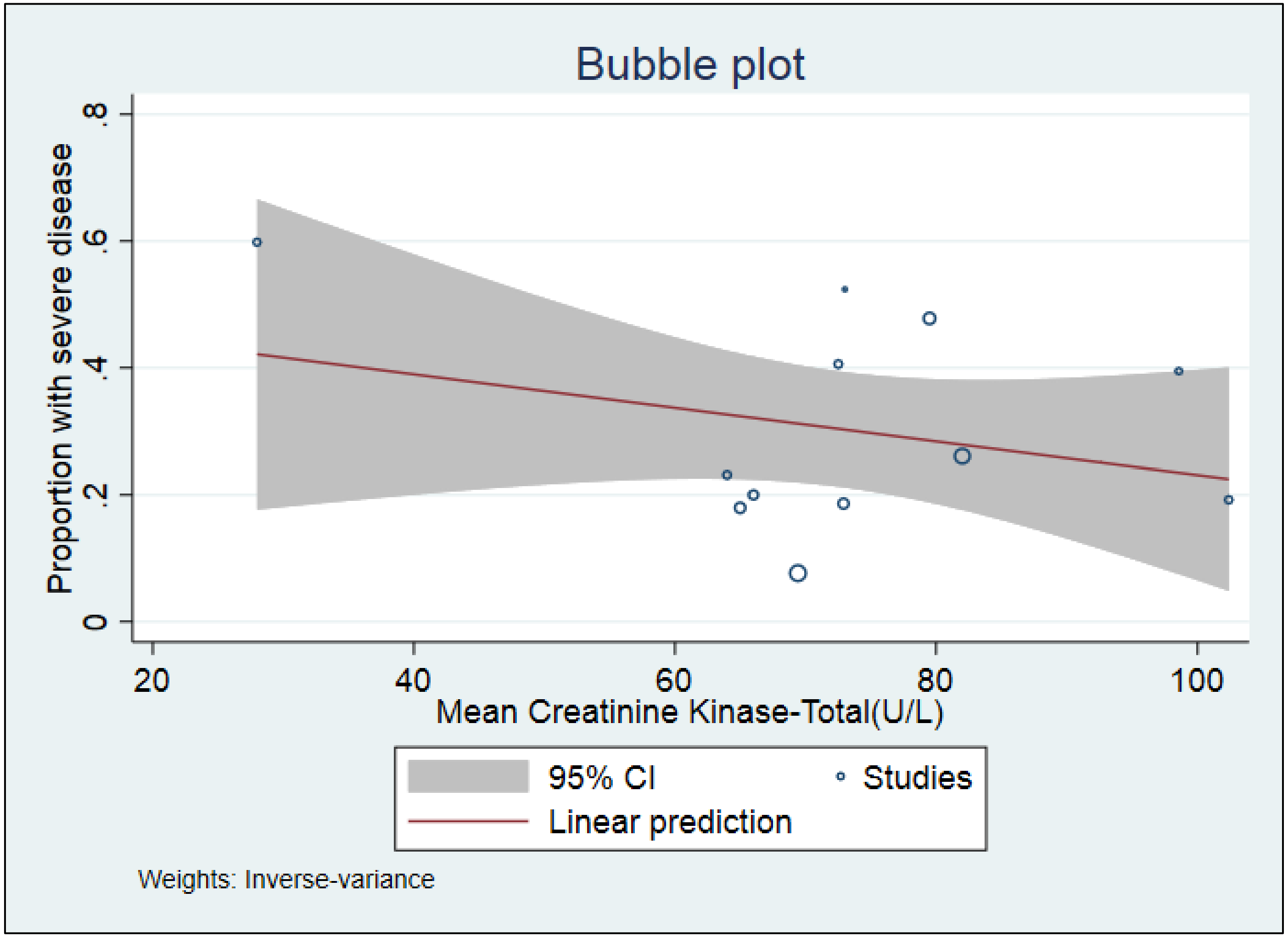

eFigure 185 Percentage change in severe disease with unit increase in mean serum CK MB

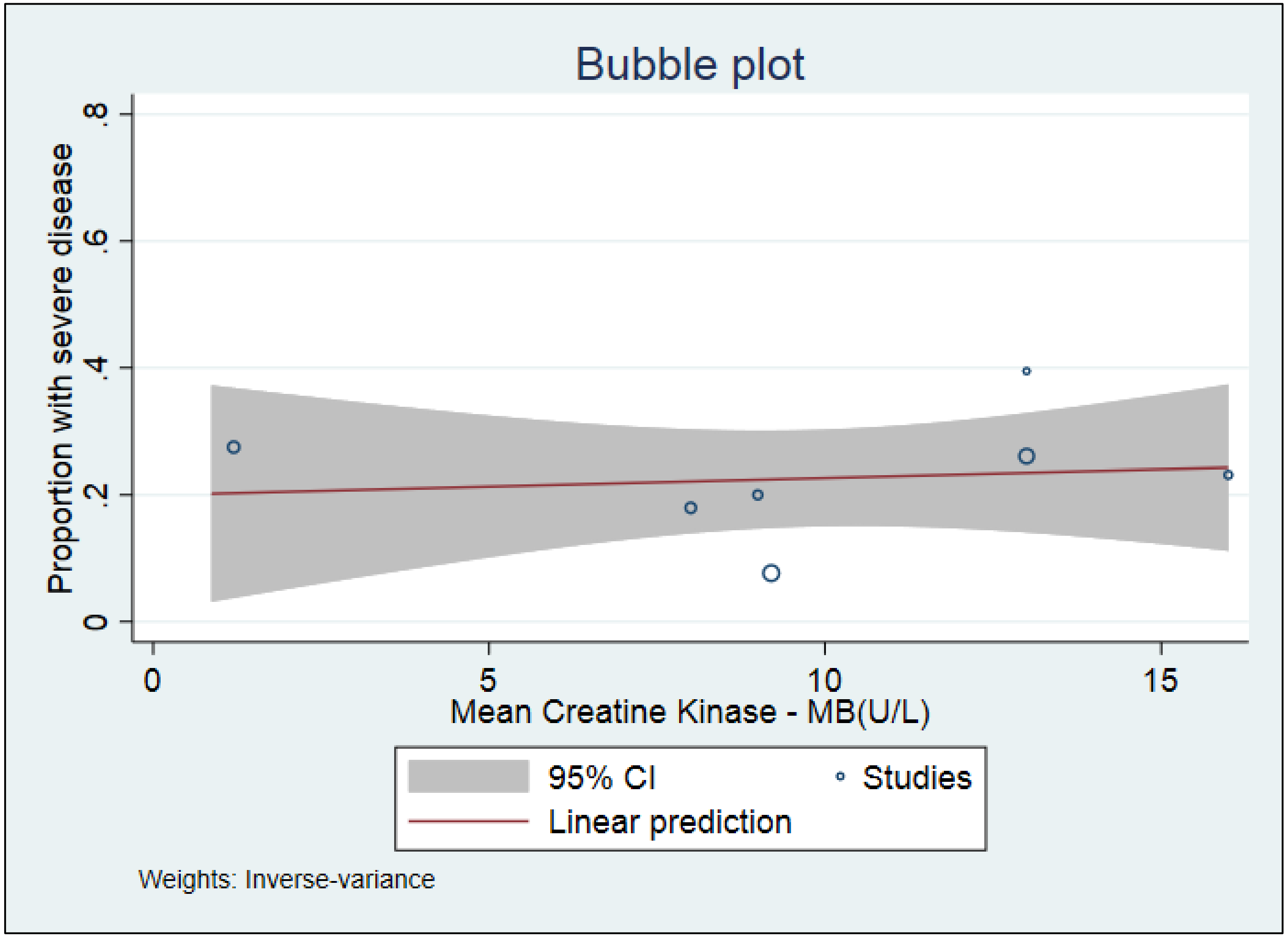

Section VII

Supplementary table 6A: Definitions of “severe disease”

Source	Definition
**American Thoracic Society guidelines for community-acquired pneumonia(110)**	Validated definition includes either one major criterion or three or more minor criteria<br>Minor criteria<br>- Respiratory rate > 30 breaths/min<br>- PaO2/FIO2 ratio < 250<br>- Multi lobar infiltrates<br>- Confusion/disorientation<br>- Uremia (blood urea nitrogen level > 20 mg/dl)<br>- Leukopenia* (white blood cell count, 4,000 cells/ml)<br>- Thrombocytopenia (platelet count, 100,000/ml)<br>- Hypothermia (core temperature, 368C)<br>- Hypotension requiring aggressive fluid resuscitation<br>Major criteria<br>- Septic shock with need for vasopressors<br>- Respiratory failure requiring mechanical ventilation
**Chinese National Health Commission guidelines(111)**<br>Severe disease in our review had features of “severe” or “critical” patients according to the CNHC guidelines.	Severe patients met any of the following condition:<br>1) respiratory rate ≥30 breaths per minute.<br>2) oxygen saturation≤93% in a resting state;<br>3) arterial oxygen tension (PaO_2)/inspiratory oxygen fraction (FiO_2) ≤300 mmHg;<br>4) respiratory failure<br>Critical patients<br>1) respiratory failure and requiring mechanical ventilation<br>2) shock<br>3) patients with other organ failure needing intensive care unit monitoring and treatment.

Supplementary table 6B: Definitions of complications assessed for association with mortality

Definitions of variables	**Source**
Sepsis and Shock	Third International Consensus Definition for Sepsis and Septic Shock(112)
Acute cardiac injury	Fourth universal definition of myocardial infarction Consensus Document(113)
Acute Respiratory Distress Syndrome (ARDS)	The Berlin Definition of Acute Respiratory Distress Syndrome(114)
Acute Kidney Injury (AKI)	KDIGO definition for AKI(115)

Section VIII

References for the supplementary document:

1. Yang X, Yu Y, Xu J, Shu H, Xia J, Liu H, et al. Clinical course and outcomes of critically ill patients with SARS-CoV-2 pneumonia in Wuhan, China: a single-centered, retrospective, observational study. The Lancet Respiratory Medicine. 2020 May;8(5):475–81.

2. Chen T, Wu D, Chen H, Yan W, Yang D, Chen G, et al. Clinical characteristics of 113 deceased patients with coronavirus disease 2019: retrospective study. BMJ. 2020 Mar 26;m1091.

3. Tang N, Li D, Wang X, Sun Z. Abnormal coagulation parameters are associated with poor prognosis in patients with novel coronavirus pneumonia. Journal of Thrombosis and Haemostasis. 2020 Apr;18(4):844–7.

4. Ruan Q, Yang K, Wang W, Jiang L, Song J. Clinical predictors of mortality due to COVID-19 based on an analysis of data of 150 patients from Wuhan, China. Intensive Care Medicine. 2020 May;46(5):846–8.

5. Wu C, Chen X, Cai Y, Xia J, Zhou X, Xu S, et al. Risk Factors Associated With Acute Respiratory Distress Syndrome and Death in Patients With Coronavirus Disease 2019 Pneumonia in Wuhan, China. JAMA Internal Medicine [Internet]. 2020 Mar 13 [cited 2020 May 25]; Available from: https://jamanetwork.com/journals/jamainternalmedicine/fullarticle/2763184

6. Li Y, Peng S, Li L, Wang Q, Ping W, Zhang N, et al. Clinical and Transmission Characteristics of Covid-19 — A Retrospective Study of 25 Cases from a Single Thoracic Surgery Department. Current Medical Science. 2020 Apr;40(2):295–300.

7. Deng Y, Liu W, Liu K, Fang Y-Y, Shang J, zhou L, et al. Clinical characteristics of fatal and recovered cases of coronavirus disease 2019 (COVID-19) in Wuhan, China: a retrospective study. Chinese Medical Journal. 2020 Mar;1.

8. Yuan M, Yin W, Tao Z, Tan W, Hu Y. Association of radiologic findings with mortality of patients infected with 2019 novel coronavirus in Wuhan, China. PLOS ONE. 2019;10.

9. Zhou F, Yu T, Du R, Fan G, Liu Y, Liu Z, et al. Clinical course and risk factors for mortality of adult inpatients with COVID-19 in Wuhan, China: a retrospective cohort study. The Lancet. 2020 Mar;395(10229):1054–62.

10. Cao J, Tu W-J, Cheng W, Yu L, Liu Y-K, Hu X, et al. Clinical Features and Short-term Outcomes of 102 Patients with Corona Virus Disease 2019 in Wuhan, China. Clinical Infectious Diseases [Internet]. 2020 Apr 2 [cited 2020 May 25]; Available from: https://academic.oup.com/cid/advance-article/doi/10.1093/cid/ciaa243/5814897

11. Du R-H, Liang L-R, Yang C-Q, Wang W, Cao T-Z, Li M, et al. Predictors of mortality for patients with COVID-19 pneumonia caused by SARS-CoV-2: a prospective cohort study. European Respiratory Journal. 2020 May;55(5):2000524.

12. Tu W-J, Cao J, Yu L, Hu X, Liu Q. Clinicolaboratory study of 25 fatal cases of COVID-19 in Wuhan. Intensive Care Medicine [Internet]. 2020 Apr 6 [cited 2020 May 25]; Available from: http://link.springer.com/10.1007/s00134-020-06023-4

13. Wang L, He W, Yu X, Hu D, Bao M, Liu H, et al. Coronavirus disease 2019 in elderly patients: Characteristics and prognostic factors based on 4-week follow-up. Journal of Infection. 2020 Jun;80(6):639–45.

14. Wang Y, Lu X, Chen H, Chen T, Su N, Huang F, et al. Clinical Course and Outcomes of 344 Intensive Care Patients with COVID-19. American Journal of Respiratory and Critical Care Medicine [Internet]. 2020 Apr 8 [cited 2020 May 25]; Available from: https://www.atsjournals.org/doi/10.1164/rccm.202003-0736LE

15. Dai M, Liu D, Liu M, Zhou F, Li G, Chen Z, et al. Patients with cancer appear more vulnerable to SARS-COV-2: a multi-center study during the COVID-19 outbreak. Cancer Discovery. 2020 Apr 28;CD-20-0422.

16. Ding M, Zhang Q, Li Q, Wu T, Huang Y. Correlation analysis of the severity and clinical prognosis of 32 cases of patients with COVID-19. Respiratory Medicine. 2020 Jun;167:105981.

17. Barrasa H, Rello J, Tejada S, Martín A, Balziskueta G, Vinuesa C, et al. SARS-CoV-2 in Spanish Intensive Care Units: Early experience with 15-day survival in Vitoria. Anaesthesia Critical Care & Pain Medicine [Internet]. 2020 Apr [cited 2020 May 25]; Available from: https://linkinghub.elsevier.com/retrieve/pii/S2352556820300643

18. Chen T, Dai Z, Mo P, Li X, Ma Z, Song S, et al. Clinical Characteristics and Outcomes of Older Patients with Coronavirus Disease 2019 (COVID-19) in Wuhan, China: A Single-Centered, Retrospective Study. Newman A, editor. The Journals of Gerontology: Series A [Internet]. 2020 Apr 11 [cited 2020 May 25]; Available from: https://academic.oup.com/biomedgerontology/advance-article/doi/10.1093/gerona/glaa089/5819242

19. Trujillo H, Caravaca-Fontán F, Sevillano Á, Gutiérrez E, Caro J, Gutiérrez E, et al. SARS-CoV-2 Infection in Hospitalized Patients With Kidney Disease. Kidney International Reports [Internet]. 2020 May [cited 2020 May 25]; Available from: https://linkinghub.elsevier.com/retrieve/pii/S2468024920312274

20. Webb GJ, Moon AM, Barnes E, Barritt AS, Marjot T. Determining risk factors for mortality in liver transplant patients with COVID-19. The Lancet Gastroenterology & Hepatology [Internet]. 2020 Apr [cited 2020 May 25]; Available from: https://linkinghub.elsevier.com/retrieve/pii/S2468125320301254

21. Yao Q, Wang P, Wang X, Qie G, Meng M, Tong X, et al. Retrospective study of risk factors for severe SARS-Cov-2 infections in hospitalized adult patients. Polish Archives of Internal Medicine [Internet]. 2020 Apr 24 [cited 2020 May 25]; Available from: https://www.mp.pl/paim/issue/article/15312

22. Wei F, Wang H, Huang H, Luo C, Zhou X, Xu N, et al. Acute myocardial injury is common in patients with covid-19 and impairs their prognosis. :6.

23. Tomlins J, Hamilton F, Gunning S, Sheehy C, Moran E, MacGowan A. Clinical features of 95 sequential hospitalised patients with novel coronavirus 2019 disease (COVID-19), the first UK cohort. Journal of Infection [Internet]. 2020 Apr [cited 2020 May 25]; Available from: https://linkinghub.elsevier.com/retrieve/pii/S0163445320302322

24. Yang X, Yang Q, Wang Y, Wu Y, Xu J, Yu Y, et al. Thrombocytopenia and its association with mortality in patients with COVID-19. Journal of Thrombosis and Haemostasis [Internet]. 2020 May 4 [cited 2020 May 25]; Available from: https://onlinelibrary.wiley.com/doi/abs/10.1111/jth.14848

25. Yang R, Gui X, Zhang Y, Xiong Y. The role of essential organ-based comorbidities in the prognosis of COVID-19 infection patients. Expert Review of Respiratory Medicine. 2020 Apr 28;1–4.

26. Richardson S, Hirsch JS, Narasimhan M, Crawford JM, McGinn T, Davidson KW, et al. Presenting Characteristics, Comorbidities, and Outcomes Among 5700 Patients Hospitalized With COVID-19 in the New York City Area. JAMA [Internet]. 2020 Apr 22 [cited 2020 May 25]; Available from: https://jamanetwork.com/journals/jama/fullarticle/2765184

27. Gold JAW, Wong KK, Szablewski CM, Patel PR, Rossow J, da Silva J, et al. Characteristics and Clinical Outcomes of Adult Patients Hospitalized with COVID-19 — Georgia, March 2020. MMWR Morbidity and Mortality Weekly Report. 2020 May 8;69(18):545–50.

28. Gao L, Jiang D, Wen X, Cheng X, Sun M, He B, et al. Prognostic value of NT-proBNP in patients with severe COVID-19. Respiratory Research [Internet]. 2020 Dec [cited 2020 May 25];21(1). Available from: https://respiratory-research.biomedcentral.com/articles/10.1186/s12931-020-01352-w

29. Singh S, Khan A. Clinical Characteristics and Outcomes of COVID-19 Among Patients with Pre-Existing Liver Disease in United States: A Multi-Center Research Network Study. Gastroenterology [Internet]. 2020 May [cited 2020 May 25]; Available from: https://linkinghub.elsevier.com/retrieve/pii/S0016508520305850

30. Zangrillo A, Beretta L, Scandroglio AM, Monti G, Fominskiy E, Colombo S, et al. Characteristics, treatment, outcomes and cause of death of invasively ventilated patients with COVID-19 ARDS in Milan, Italy. Critical Care and Resuscitation. :12.

31. Yan Y, Yang Y, Wang F, Ren H, Zhang S, Shi X, et al. Clinical characteristics and outcomes of patients with severe covid-19 with diabetes. BMJ Open Diabetes Research & Care. 2020 Apr;8(1):e001343.

32. Wang K, Zuo P, Liu Y, Zhang M, Zhao X, Xie S, et al. Clinical and laboratory predictors of in-hospital mortality in patients with COVID-19: a cohort study in Wuhan, China. Clin Infect Dis [Internet]. [cited 2020 May 26]; Available from: https://academic.oup.com/cid/advance-article/doi/10.1093/cid/ciaa538/5828281

33. Wang K, Zhang Z, Yu M, Tao Y, Xie M. 15-day mortality and associated risk factors for hospitalized patients with COVID-19 in Wuhan, China: an ambispective observational cohort study. Intensive Care Medicine [Internet]. 2020 Apr 23 [cited 2020 May 25]; Available from: http://link.springer.com/10.1007/s00134-020-06047-w

34. Zhang L, Yan X, Fan Q, Liu H, Liu X, Liu Z, et al. D-dimer levels on admission to predict in-hospital mortality in patients with Covid-19. Journal of Thrombosis and Haemostasis [Internet]. 2020 Apr 19 [cited 2020 May 25]; Available from: https://onlinelibrary.wiley.com/doi/abs/10.1111/jth.14859

35. Wang D, Yin Y, Hu C, Liu X, Zhang X, Zhou S, et al. Clinical course and outcome of 107 patients infected with the novel coronavirus, SARS-CoV-2, discharged from two hospitals in Wuhan, China. Critical Care [Internet]. 2020 Dec [cited 2020 May 25];24(1). Available from: https://ccforum.biomedcentral.com/articles/10.1186/s13054-020-02895-6

36. Liu Y, Sun W, Guo Y, Chen L, Zhang L, Zhao S, et al. Association between platelet parameters and mortality in coronavirus disease 2019: Retrospective cohort study. Platelets. 2020 May 18;31(4):490–6.

37. Liu Y, Du X, Chen J, Jin Y, Peng L, Wang HHX, et al. Neutrophil-to-lymphocyte ratio as an independent risk factor for mortality in hospitalized patients with COVID-19. Journal of Infection [Internet]. 2020 Apr [cited 2020 May 25]; Available from: https://linkinghub.elsevier.com/retrieve/pii/S0163445320302085

38. Li J, Wang X, Chen J, Zhang H, Deng A. Association of Renin-Angiotensin System Inhibitors With Severity or Risk of Death in Patients With Hypertension Hospitalized for Coronavirus Disease 2019 (COVID-19) Infection in Wuhan, China. JAMA Cardiology [Internet]. 2020 Apr 23 [cited 2020 May 25]; Available from: https://jamanetwork.com/journals/jamacardiology/fullarticle/2765049

39. Nikpouraghdam M, Jalali Farahani A, Alishiri G, Heydari S, Ebrahimnia M, Samadinia H, et al. Epidemiological characteristics of coronavirus disease 2019 (COVID-19) patients in IRAN: A single center study. Journal of Clinical Virology. 2020 Jun;127:104378.

40. Zhang J, Wang X, Jia X, Li J, Hu K, Chen G, et al. Risk factors for disease severity, unimprovement, and mortality in COVID-19 patients in Wuhan, China. Clinical Microbiology and Infection. 2020 Jun;26(6):767–72.

41. Mehta V, Goel S, Kabarriti R, Cole D, Goldfinger M, Acuna-Villaorduna A, et al. Case Fatality Rate of Cancer Patients with COVID-19 in a New York Hospital System. Cancer Discovery. 2020 May 1;CD-20-0516.

42. Zou X, Li S, Fang M, Hu M, Bian Y, Ling J, et al. Acute Physiology and Chronic Health Evaluation II Score as a Predictor of Hospital Mortality in Patients of Coronavirus Disease 2019: Critical Care Medicine. 2020 May;1.

43. Guan W, Ni Z, Hu Y, Liang W, Ou C, He J, et al. Clinical Characteristics of Coronavirus Disease 2019 in China. New England Journal of Medicine. 2020 Apr 30;382(18):1708–20.

44. Xu Y-H, Dong J-H, An W-M, Lv X-Y, Yin X-P, Zhang J-Z, et al. Clinical and computed tomographic imaging features of novel coronavirus pneumonia caused by SARS-CoV-2. Journal of Infection. 2020 Apr;80(4):394–400.

45. Tian S, Hu N, Lou J, Chen K, Kang X, Xiang Z, et al. Characteristics of COVID-19 infection in Beijing. Journal of Infection. 2020 Apr;80(4):401–6.

46. Li K, Wu J, Wu F, Guo D, Chen L, Fang Z, et al. The Clinical and Chest CT Features Associated With Severe and Critical COVID-19 Pneumonia: Investigative Radiology. 2020 Jun;55(6):327–31.

47. Zhao W, Zhong Z, Xie X, Yu Q, Liu J. Relation Between Chest CT Findings and Clinical Conditions of Coronavirus Disease (COVID-19) Pneumonia: A Multicenter Study. American Journal of Roentgenology. 2020 May;214(5):1072–7.

48. Han H, Yang L, Liu R, Liu F, Wu K, Li J, et al. Prominent changes in blood coagulation of patients with SARS-CoV-2 infection. Clinical Chemistry and Laboratory Medicine (CCLM) [Internet]. 2020 Mar 16 [cited 2020 May 25];0(0). Available from: https://www.degruyter.com/view/journals/cclm/ahead-of-print/article-10.1515-cclm-2020-0188/article-10.1515-cclm-2020-0188.xml

49. Qian G-Q, Yang N-B, Ding F, Ma AHY, Wang Z-Y, Shen Y-F, et al. Epidemiologic and clinical characteristics of 91 hospitalized patients with COVID-19 in Zhejiang, China: a retrospective, multi-centre case series. QJM [Internet]. [cited 2020 May 26]; Available from: https://academic.oup.com/qjmed/advance-article/doi/10.1093/qjmed/hcaa089/5809152

50. Qu R, Ling Y, Zhang Y, Wei L, Chen X, Li X, et al. Platelet-to-lymphocyte ratio is associated with prognosis in patients with Corona Virus Disease-19. Journal of Medical Virology [Internet]. 2020 Mar 17 [cited 2020 May 25]; Available from: https://onlinelibrary.wiley.com/doi/abs/10.1002/jmv.25767

51. Gao Y, Li T, Han M, Li X, Wu D, Xu Y, et al. Diagnostic Utility of Clinical Laboratory Data Determinations for Patients with the Severe COVID-19. Journal of Medical Virology [Internet]. 2020 Mar 17 [cited 2020 May 25]; Available from: https://onlinelibrary.wiley.com/doi/abs/10.1002/jmv.25770

52. Liu K-C, Xu P, Lv W-F, Qiu X-H, Yao J-L, Gu J-F, et al. CT manifestations of coronavirus disease-2019: A retrospective analysis of 73 cases by disease severity. European Journal of Radiology. 2020 May;126:108941.

53. Wan S, Xiang Y, Fang W, Zheng Y, Li B, Hu Y, et al. Clinical features and treatment of COVID-19 patients in northeast Chongqing. Journal of Medical Virology [Internet]. 2020 Apr 1 [cited 2020 May 25]; Available from: http://doi.wiley.com/10.1002/jmv.25783

54. Shi Y, Yu X, Zhao H, Wang H, Zhao R, Sheng J. Host susceptibility to severe COVID-19 and establishment of a host risk score: findings of 487 cases outside Wuhan. Critical Care [Internet]. 2020 Dec [cited 2020 May 25];24(1). Available from: https://ccforum.biomedcentral.com/articles/10.1186/s13054-020-2833-7

55. Li K, Fang Y, Li W, Pan C, Qin P, Zhong Y, et al. CT image visual quantitative evaluation and clinical classification of coronavirus disease (COVID-19). European Radiology [Internet]. 2020 Mar 25 [cited 2020 May 25]; Available from: http://link.springer.com/10.1007/s00330-020-06817-6

56. Qin C, Zhou L, Hu Z, Zhang S, Yang S, Tao Y, et al. Dysregulation of Immune Response in Patients with COVID-19 in Wuhan, China. SSRN Electronic Journal [Internet]. 2020 [cited 2020 May 25]; Available from: https://www.ssrn.com/abstract=3541136

57. Huang L, Han R, Ai T, Yu P, Kang H, Tao Q, et al. Serial Quantitative Chest CT Assessment of COVID-19: Deep-Learning Approach. Radiology: Cardiothoracic Imaging. 2020 Apr 1;2(2):e200075.

58. Zhang J, Dong X, Cao Y, Yuan Y, Yang Y, Yan Y, et al. Clinical characteristics of 140 patients infected with SARS-CoV-2 in Wuhan, China. Allergy [Internet]. 2020 Feb 27 [cited 2020 May 25]; Available from: https://onlinelibrary.wiley.com/doi/abs/10.1111/all.14238

59. Zhang G, Zhang J, Wang B, Zhu X, Wang Q, Qiu S. Analysis of clinical characteristics and laboratory findings of 95 cases of 2019 novel coronavirus pneumonia in Wuhan, China: a retrospective analysis. Respiratory Research [Internet]. 2020 Dec [cited 2020 May 25];21(1). Available from: https://respiratory-research.biomedcentral.com/articles/10.1186/s12931-020-01338-8

60. Chen G, Wu D, Guo W, Cao Y, Huang D, Wang H, et al. Clinical and immunological features of severe and moderate coronavirus disease 2019. Journal of Clinical Investigation. 2020 Apr 13;130(5):2620–9.

61. Chu J, Yang N, Wei Y, Yue H, Zhang F, Zhao J, et al. Clinical characteristics of 54 medical staff with COVID-19: A retrospective study in a single center in Wuhan, China. Journal of Medical Virology [Internet]. 2020 Apr 6 [cited 2020 May 25]; Available from: https://onlinelibrary.wiley.com/doi/abs/10.1002/jmv.25793

62. Yuan J, Zou R, Zeng L, Kou S, Lan J, Li X, et al. The correlation between viral clearance and biochemical outcomes of 94 COVID-19 infected discharged patients. Inflammation Research. 2020 Jun;69(6):599–606.

63. Wang L, Li X, Chen H, Yan S, Li D, Li Y, et al. Coronavirus Disease 19 Infection Does Not Result in Acute Kidney Injury: An Analysis of 116 Hospitalized Patients from Wuhan, China. American Journal of Nephrology. 2020;51(5):343–8.

64. Han H, Xie L, Liu R, Yang J, Liu F, Wu K, et al. Analysis of heart injury laboratory parameters in 273 COVID-19 patients in one hospital in Wuhan, China. Journal of Medical Virology [Internet]. 2020 Apr 15 [cited 2020 May 25]; Available from: http://doi.wiley.com/10.1002/jmv.25809

65. Xie H, Zhao J, Lian N, Lin S, Xie Q, Zhuo H. Clinical characteristics of non-ICU hospitalized patients with coronavirus disease 2019 and liver injury: A retrospective study. Liver International [Internet]. 2020 Apr 12 [cited 2020 May 25]; Available from: http://doi.wiley.com/10.1111/liv.14449

66. Cai Q, Huang D, Ou P, Yu H, Zhu Z, Xia Z, et al. COVID-19 in a designated infectious diseases hospital outside Hubei Province, China. Allergy [Internet]. 2020 Apr 17 [cited 2020 May 25]; Available from: https://onlinelibrary.wiley.com/doi/abs/10.1111/all.14309

67. Zhang Y, Zheng L, Liu L, Zhao M, Xiao J, Zhao Q. Liver impairment in COVID-19 patients: A retrospective analysis of 115 cases from a single centre in Wuhan city, China. Liver International [Internet]. 2020 Apr 28 [cited 2020 May 25]; Available from: https://onlinelibrary.wiley.com/doi/abs/10.1111/liv.14455

68. Wang L. C-reactive protein levels in the early stage of COVID-19. Médecine et Maladies Infectieuses. 2020 Jun;50(4):332–4.

69. Chen X, Yang Y, Huang M, Liu L, Zhang X, Xu J, et al. Differences between COVID-19 and suspected then confirmed SARS-CoV-2-negative pneumonia: a retrospective study from a single center. Journal of Medical Virology [Internet]. 2020 Apr 1 [cited 2020 May 25]; Available from: http://doi.wiley.com/10.1002/jmv.25810

70. Zheng F, Tang W, Li H. Clinical characteristics of 161 cases of corona virus disease 2019 (COVID-19) in Changsha. :7.

71. Deng Q, Hu B, Zhang Y, Wang H, Zhou X, Hu W, et al. Suspected myocardial injury in patients with COVID-19: Evidence from front-line clinical observation in Wuhan, China. International Journal of Cardiology [Internet]. 2020 Apr [cited 2020 May 25]; Available from: https://linkinghub.elsevier.com/retrieve/pii/S0167527320311153

72. Li H, Xiang X, Ren H, Xu L, Zhao L, Chen X, et al. Serum Amyloid A is a biomarker of severe Coronavirus Disease and poor prognosis. Journal of Infection. 2020 Jun;80(6):646–55.

73. Zou Y, Guo H, Zhang Y, Zhang Z, Liu Y, Wang J, et al. Analysis of coagulation parameters in patients with COVID-19 in Shanghai, China. BioScience Trends [Internet]. 2020 [cited 2020 May 25]; Available from: https://www.jstage.jst.go.jp/article/bst/advpub/0/advpub_2020.03086/_article

74. Shi F, Wu T, Zhu X, Ge Y, Zeng X, Chi Y, et al. Association of viral load with serum biomakers among COVID-19 cases. Virology. 2020 Jul;546:122–6.

75. Wang G, Wu C, Zhang Q, Wu F, Yu B, Lv J, et al. C-Reactive Protein Level May Predict the Risk of COVID-19 Aggravation. Open Forum Infectious Diseases [Internet]. 2020 May 1 [cited 2020 May 25];7(5). Available from: https://academic.oup.com/ofid/article/doi/10.1093/ofid/ofaa153/5826961

76. Ji M, Yuan L, Shen W, Lv J, Li Y, Li M, et al. Characteristics of disease progress in patients with coronavirus disease 2019 in Wuhan, China. Epidemiology and Infection [Internet]. 2020 [cited 2020 May 25];148. Available from: https://www.cambridge.org/core/product/identifier/S0950268820000977/type/journal_article

77. Shen L, Li S, Zhu Y, Zhao J, Tang X, Li H, et al. Clinical and laboratory-derived parameters of 119 hospitalized patients with coronavirus disease 2019 in Xiangyang, Hubei Province, China. Journal of Infection [Internet]. 2020 Apr [cited 2020 May 25]; Available from: https://linkinghub.elsevier.com/retrieve/pii/S0163445320301663

78. Yang F, Shi S, Zhu J, Shi J, Dai K, Chen X. Clinical characteristics and outcomes of cancer patients with COVID-19. Journal of Medical Virology [Internet]. 2020 May 5 [cited 2020 May 25]; Available from: https://onlinelibrary.wiley.com/doi/abs/10.1002/jmv.25972

79. Ma J, Yin J, Qian Y, Wu Y. Clinical characteristics and prognosis in cancer patients with COVID-19: A single center's retrospective study. Journal of Infection [Internet]. 2020 Apr [cited 2020 May 25]; Available from: https://linkinghub.elsevier.com/retrieve/pii/S0163445320302140

80. Chen Q, Zheng Z, Zhang C, Zhang X, Wu H, Wang J, et al. Clinical characteristics of 145 patients with corona virus disease 2019 (COVID-19) in Taizhou, Zhejiang, China. Infection [Internet]. 2020 Apr 28 [cited 2020 May 25]; Available from: http://link.springer.com/10.1007/s15010-020-01432-5

81. Colaneri M, Sacchi P, Zuccaro V, Biscarini S, Sachs M, Roda S, et al. Clinical characteristics of coronavirus disease (COVID-19) early findings from a teaching hospital in Pavia, North Italy, 21 to 28 February 2020. Eurosurveillance [Internet]. 2020 Apr 23 [cited 2020 May 25];25(16). Available from: https://www.eurosurveillance.org/content/10.2807/1560-7917.ES.2020.25.16.2000460

82. Zhao X-Y, Xu X-X, Yin H-S, Hu Q-M, Xiong T, Tang Y-Y, et al. Clinical characteristics of patients with 2019 coronavirus disease in a non-Wuhan area of Hubei Province, China: a retrospective study. BMC Infectious Diseases [Internet]. 2020 Dec [cited 2020 May 25];20(1). Available from: https://bmcinfectdis.biomedcentral.com/articles/10.1186/s12879-020-05010-w

83. Chen L, Li Q, Zheng D, Jiang H, Wei Y, Zou L, et al. Clinical Characteristics of Pregnant Women with Covid-19 in Wuhan, China. New England Journal of Medicine [Internet]. 2020 Apr 17 [cited 2020 May 25]; Available from: http://www.nejm.org/doi/10.1056/NEJMc2009226

84. Sun L, Shen L, Fan J, Gu F, Hu M, An Y, et al. Clinical Features of Patients with Coronavirus Disease 2019 (COVID-19) from a Designated Hospital in Beijing, China. Journal of Medical Virology [Internet]. 2020 May 5 [cited 2020 May 25]; Available from: https://onlinelibrary.wiley.com/doi/abs/10.1002/jmv.25966

85. Zhu Z, Cai T, Fan L, Lou K, Hua X, Huang Z, et al. Clinical value of immune-inflammatory parameters to assess the severity of coronavirus disease 2019. International Journal of Infectious Diseases. 2020 Jun;95:332–9.

86. Liu Y, Liao W, Wan L, Xiang T, Zhang W. Correlation Between Relative Nasopharyngeal Virus RNA Load and Lymphocyte Count Disease Severity in Patients with COVID-19. Viral Immunology [Internet]. 2020 Apr 10 [cited 2020 May 25]; Available from: https://www.liebertpub.com/doi/10.1089/vim.2020.0062

87. Feng Y, Ling Y, Bai T, Xie Y, Huang J, Li J, et al. COVID-19 with Different Severity: A Multi-center Study of Clinical Features. American Journal of Respiratory and Critical Care Medicine [Internet]. 2020 Apr 10 [cited 2020 May 25]; Available from: https://www.atsjournals.org/doi/10.1164/rccm.202002-0445OC

88. Zhang R, Ouyang H, Fu L, Wang S, Han J, Huang K, et al. CT features of SARS-CoV-2 pneumonia according to clinical presentation: a retrospective analysis of 120 consecutive patients from Wuhan city. European Radiology [Internet]. 2020 Apr 11 [cited 2020 May 25]; Available from: http://link.springer.com/10.1007/s00330-020-06854-1

89. Chen X, Zhao B, Qu Y, Chen Y, Xiong J, Feng Y, et al. Detectable serum SARS-CoV-2 viral load (RNAaemia) is closely correlated with drastically elevated interleukin 6 (IL-6) level in critically ill COVID-19 patients. Clinical Infectious Diseases [Internet]. 2020 Apr 17 [cited 2020 May 25]; Available from: https://academic.oup.com/cid/advance-article/doi/10.1093/cid/ciaa449/5821311

90. Wei X, Su J, Yang K, Wei J, Wan H, Cao X, et al. Elevations of serum cancer biomarkers correlate with severity of COVID-19. Journal of Medical Virology [Internet]. 2020 Apr 29 [cited 2020 May 25]; Available from: https://onlinelibrary.wiley.com/doi/abs/10.1002/jmv.25957

91. Zheng Y, Xiong C, Liu Y, Qian X, Tang Y, Liu L, et al. Epidemiological and clinical characteristics analysis of COVID-19 in the surrounding areas of Wuhan, Hubei Province in 2020. Pharmacological Research. 2020 Jul 1;157:104821.

92. Wang R, Pan M, Zhang X, Han M, Fan X, Zhao F, et al. Epidemiological and clinical features of 125 Hospitalized Patients with COVID-19 in Fuyang, Anhui, China. International Journal of Infectious Diseases. 2020 Jun;95:421–8.

93. Yao Z, Zheng Z, Wu K, Junhua Z. Immune environment modulation in pneumonia patients caused by coronavirus: SARS-CoV, MERS-CoV and SARS-CoV-2. Aging. 2020 May 2;12(9):7639–51.

94. Lei F, Liu Y, Zhou F, Qin J, Zhang P, Zhu L, et al. Longitudinal association between markers of liver injury and mortality in COVID-19 in China. Hepatology [Internet]. 2020 May 2 [cited 2020 May 25]; Available from: https://onlinelibrary.wiley.com/doi/abs/10.1002/hep.31301

95. Lu L, Xiong W, Liu D, Liu J, Yang D, Li N, et al. New onset acute symptomatic seizure and risk factors in coronavirus disease 2019: A retrospective multicenter study. Epilepsia [Internet]. 2020 May 2 [cited 2020 May 25]; Available from: https://onlinelibrary.wiley.com/doi/abs/10.1111/epi.16524

96. Yang Y, Shen C, Li J, Yuan J, Wei J, Huang F, et al. Plasma IP-10 and MCP-3 levels are highly associated with disease severity and predict the progression of COVID-19. Journal of Allergy and Clinical Immunology [Internet]. 2020 Apr [cited 2020 May 25]; Available from: https://linkinghub.elsevier.com/retrieve/pii/S0091674920305765

97. Bi X, Su Z, Yan H, Du J, Wang J, Chen L, et al. Prediction of severe illness due to COVID-19 based on an analysis of initial Fibrinogen to Albumin Ratio and Platelet count. Platelets. 2020 May 5;1–6.

98. Liu F, Li L, Xu M, Wu J, Luo D, Zhu Y, et al. Prognostic value of interleukin-6, C-reactive protein, and procalcitonin in patients with COVID-19. Journal of Clinical Virology. 2020 Jun;127:104370.

99. Pei G, Zhang Z, Peng J, Liu L, Zhang C, Yu C, et al. Renal Involvement and Early Prognosis in Patients with COVID-19 Pneumonia. Journal of the American Society of Nephrology. 2020 Apr 28;ASN.2020030276.

100. Hu L, Chen S, Fu Y, Gao Z, Long H, Wang J, et al. Risk Factors Associated with Clinical Outcomes in 323 COVID-19 Hospitalized Patients in Wuhan, China. Clinical Infectious Diseases [Internet]. 2020 May 3 [cited 2020 May 25]; Available from: https://academic.oup.com/cid/advance-article/doi/10.1093/cid/ciaa539/5828282

101. Liu X, Zhou H, Zhou Y, Wu X, Zhao Y, Lu Y, et al. Risk factors associated with disease severity and length of hospital stay in COVID-19 patients. Journal of Infection [Internet]. 2020 Apr [cited 2020 May 25]; Available from: https://linkinghub.elsevier.com/retrieve/pii/S0163445320302164

102. Wei Y-Y, Wang R-R, Zhang D-W, Tu Y-H, Chen C-S, Ji S, et al. Risk factors for severe COVID-19: Evidence from 167 hospitalized patients in Anhui, China. Journal of Infection [Internet]. 2020 Apr [cited 2020 May 25]; Available from: https://linkinghub.elsevier.com/retrieve/pii/S016344532030219X

103. Li X, Xu S, Yu M, Wang K, Tao Y, Zhou Y, et al. Risk factors for severity and mortality in adult COVID-19 inpatients in Wuhan. Journal of Allergy and Clinical Immunology [Internet]. 2020 Apr [cited 2020 May 25]; Available from: https://linkinghub.elsevier.com/retrieve/pii/S0091674920304954

104. Zheng Y, Zhang Y, Chi H, Chen S, Peng M, Luo L, et al. The hemocyte counts as a potential biomarker for predicting disease progression in COVID-19: a retrospective study. Clinical Chemistry and Laboratory Medicine (CCLM) [Internet]. 2020 Apr 29 [cited 2020 May 25];0(0). Available from: https://www.degruyter.com/view/journals/cclm/ahead-of-print/article-10.1515-cclm-2020-0377/article-10.1515-cclm-2020-0377.xml

105. Wang F, Hou H, Luo Y, Tang G, Wu S, Huang M, et al. The laboratory tests and host immunity of COVID-19 patients with different severity of illness. JCI Insight [Internet]. 2020 May 21 [cited 2020 May 25];5(10). Available from: https://insight.jci.org/articles/view/137799

106. Lyu P, Liu X, Zhang R, Shi L, Gao J. The performance of chest CT in evaluating the clinical severity of COVID-19 pneumonia: identifying critical cases based on CT characteristics. Investigative Radiology. 2020 Apr;1.

107. Liu R, Ma Q, Han H, Su H, Liu F, Wu K, et al. The value of urine biochemical parameters in the prediction of the severity of coronavirus disease 2019. Clinical Chemistry and Laboratory Medicine (CCLM) [Internet]. 2020 Apr 14 [cited 2020 May 25];0(0). Available from: https://www.degruyter.com/view/journals/cclm/ahead-of-print/article-10.1515-cclm-2020-0220/article-10.1515-cclm-2020-0220.xml

108. Hou H, Zhang B, Huang H, Luo Y, Wu S, Tang G, et al. Using IL-2R/lymphocytes for predicting the clinical progression of patients with COVID-19. Clinical & Experimental Immunology [Internet]. 2020 May 15 [cited 2020 May 25]; Available from: https://onlinelibrary.wiley.com/doi/abs/10.1111/cei.13450

109. Zheng S, Fan J, Yu F, Feng B, Lou B, Zou Q, et al. Viral load dynamics and disease severity in patients infected with SARS-CoV-2 in Zhejiang province, China, January-March 2020: retrospective cohort study. BMJ. 2020 Apr 21;m1443.

110. Metlay JP, Waterer GW, Long AC, Anzueto A, Brozek J, Crothers K, et al. Diagnosis and Treatment of Adults with Community-acquired Pneumonia. An Official Clinical Practice Guideline of the American Thoracic Society and Infectious Diseases Society of America. Am J Respir Crit Care Med. 2019 Oct 1;200(7):e45–67.

111. Lin L, Li TS. Interpretation of "Guidelines for the Diagnosis and Treatment of Novel Coronavirus (2019-nCoV) Infection by the National Health Commission (Trial Version 5)." Zhonghua Yi Xue Za Zhi. 2020;100(0):E001–E001.

112. Singer M, Deutschman CS, Seymour CW, Shankar-Hari M, Annane D, Bauer M, et al. The Third International Consensus Definitions for Sepsis and Septic Shock (Sepsis-3). JAMA. 2016 Feb 23;315(8):801–10.

113. Fourth Universal Definition of Myocardial Infarction [Internet]. American College of Cardiology. [cited 2020 Jun 17]. Available from: http%3a%2f%2fwww.acc.org%2flatest-in-cardiology%2ften-points-to-remember%2f2018%2f08%2f24%2f00%2f09%2ffourth-universal-definition-of-mi-esc-2018

114. Acute Respiratory Distress Syndrome: The Berlin Definition. JAMA. 2012 Jun 20;307(23):2526–33.

115. Kellum JA, Lameire N, Aspelin P, Barsoum RS, Burdmann EA, Goldstein SL, et al. Kidney disease: Improving global outcomes (KDIGO) acute kidney injury work group. KDIGO clinical practice guideline for acute kidney injury. Kidney International Supplements. 2012 Mar;2(1):1–138.
