# Supplementary material for: Factors associated with disease severity and mortality among patients with COVID-19: A systematic review and meta-analysis
Source: PLoS One. 2020 Nov 18;15(11):e0241541. doi: 10.1371/journal.pone.0241541 (PMC7673562; doi:10.1371/journal.pone.0241541)
Supplement: S1 File — (DOCX) [file pone.0241541.s003.docx]

**Study Protocol**

**Factors Associated with Mortality and Disease Severity among Patients with COVID-19 Disease: A Systematic Review and Meta-Analysis**

**Background**

In the wake of the recent unprecedented increase in the number of cases of Coronavirus disease-2019 (COVID-19) and the overwhelming increase in the requirement for the medical care of the patients who develop complications, it has become an important need to determine the factors that are associated with a worse prognosis. This study can be of help in generating a triage protocol for the health facilities regarding the level of care that should be provided to the individuals with the infection. With the massive influx of studies in the past 2 to 3 months and with the conflicting findings, a systematic review on this topic which takes into consideration the heterogeneity between the studies, becomes a necessity.

**Research question**

To determine the demographic, clinical, laboratory, and radiological features that are associated with mortality and disease severity among patients diagnosed with COVID-19.

**Electronic searches**

We will search PubMed, EMBASE and the WHO COVID-19 database by using the search strategy mentioned below. All relevant published studies from these databases will be screened and extracted from inception up to March 26, 2020. An update of the existing literature search may be performed depending on the timeline of publication of the review.

**PubMed Search Strategy**

"COVID-19" [Supplementary Concept] OR "severe acute respiratory syndrome coronavirus 2" [Supplementary Concept] OR 2019 novel coronavirus [tw] OR SARS2 [tw] OR coronavirus disease-19 OR COVID-19 [tw] OR "COVID 19"[tw] OR "COVID19"[tw] OR "COVID2019"[tw] OR "COVID 2019"[tw] OR "COVID-2019"[tw] OR "novel coronavirus"[tw] OR "new coronavirus"[tw] OR "novel corona virus"[tw] OR "new corona virus"[tw] OR "SARS-CoV-2"[tw] OR "SARSCoV2"[tw] OR "SARS-CoV2"[tw] OR "2019nCoV"[tw] OR "2019-nCoV"[tw] OR "2019 coronavirus"[tw] OR "2019 corona virus"[tw] OR "coronavirus disease 2019"[tw] OR "severe acute respiratory syndrome coronavirus 2"[nm] OR "severe acute respiratory syndrome coronavirus 2"[tw] OR "sars-coronavirus-2"[tw] OR "coronavirus disease 2019"[tw] OR "corona virus disease 2019"[tw] OR (wuhan[tw] AND (“2019/01/01”[PDAT]: “3000/12/31”[PDAT]))

**Embase Search Strategy**

'2019 novel coronavirus'/exp  OR  SARS2:ab,ti,kw OR ‘Wuhan coronavirus’:ab,ti,kw OR ‘Wuhan seafood market pneumonia virus’:ab,ti,kw OR coronavirus disease-19:ab,ti,kw OR COVID-19:ab,ti,kw OR ‘COVID 19’:ab,ti,kw OR ‘COVID19’:ab,ti,kw OR ‘COVID2019’:ab,ti,kw OR ‘COVID 2019’:ab,ti,kw OR ‘COVID-2019’:ab,ti,kw OR ‘novel coronavirus’:ab,ti,kw OR ‘new coronavirus’:ab,ti,kw OR ‘novel corona virus’:ab,ti,kw OR ‘new corona virus’:ab,ti,kw OR ‘SARS-CoV-2 ’:ab,ti,kw OR ‘SARSCoV2’:ab,ti,kw OR ‘SARS-CoV2:ab,ti,kw’ OR ‘2019nCo:ab,ti,kw V’:ab,ti,kw OR ‘2019-nCoV’:ab,ti,kw OR ‘2019 coronavirus’:ab,ti,kw OR ‘2019 corona virus’:ab,ti,kw OR ‘coronavirus disease 2019’:ab,ti,kw OR ‘severe acute respiratory syndrome coronavirus 2’:ab,ti,kw OR ‘severe acute respiratory syndrome coronavirus 2’:ab,ti,kw OR ‘sars-coronavirus-2’:ab,ti,kw OR ‘coronavirus disease 2019’:ab,ti,kw OR ‘corona virus disease 2019’:ab,ti,kw OR (wuhan:ti,ab,kw AND [2019-2020]/py)

***Other searches***

We will also include the studies that are cited in other published journal articles and reviews. We will not use abstracts and conference reports if we are not able to obtain full information from the study authors. Studies in the pre-prints will not be included for this review because of the lack of peer review involved in the publication of these articles.

**Methods**

**Type of studies included**

Observational studies that reported a direct comparison of clinical, laboratory or radiologic characteristics between a) patients who died and those who survived or b) patients with severe disease and those without severe disease, will be included in the review. Case reports, case series and other description studies will be excluded as measures of association could not be obtained from these study designs. The study design will be assessed independently by two reviewers and then labelled as cohort, case control or cross-sectional analytical according. Case series and cohort will be distinguished based on the criteria outlined by Dekkers et al(1). Only articles in the English language will be included in review.

**Condition or domain being studied**

Microbiologically confirmed SARS-CoV-2 infection

**Participants/population**

We will include studies on patients admitted for COVID-19 in the hospital irrespective of the age of the participants. We will only include studies reporting primary hospital data on patients, while studies with centralized data from national health agencies and databases will be excluded from our review. Data will be collected on the name of the hospital, date of hospital admission of the participants and the names of the investigators.

**Exposure(s)**For exposure reported as binary parameters (Table 1), summary data on the patients with the following exposures (with the characteristics) and comparators (without the characteristics) will be recorded.

**Table 1:**

| **Demographic/ Behavioural**  Male Sex  Smoking status  **Co-morbidities**  Diabetes  Hypertension  Cardiovascular diseases  Congestive Heart Failure  Cerebrovascular Disease  COPD  Asthma  CKD  Chronic liver disease  Hepatitis B infection  HIV  Cancer  Immunodeficiency  Endocrine diseases | **Clinical features**  Fever  Sore throat  Cough  Expectoration  Vomiting  Diarrhea  Nausea  Myalgia  Headache  Anorexia  Chest pain  Dyspnea  Hemoptysis  Abdominal pain  Palpitations  Rhinorrhea  Anosmia | |
| --- | --- | --- |
| **Complications**  ARDS  Shock  Sepsis  Bacteremia  Acute cardiac injury  Acute heart failure  DIC  GI bleeding  Acute Kidney Injury  Acute liver injury  Hepatic encephalopathy  Ventilator associated pneumonia | **Radiological findings**  Peripheral distribution  Bilateral involvement  Consolidation  GGO  Mixed GGO and consolidation  Air bronchogram  Nodular infiltrates  Hilar Lymphadenopathy  Tree in bud appearance  Unifocal involvement  Pleural effusion  Pleural thickening  Inter–lobular septal thickening  Bronchiectasis  Linear infiltrates  Crazy pavement sign  Reticular pattern | |
| **Laboratory Parameters (Along with the cut offs)**  Total leucocyte count (x109/L)  Neutrophil count (x109/L)  Lymphocyte count (x109/L)  Platelet count (x109/L)  Albumin (g/L)  Globulin (g/L)  T· Bilirubin (umol/L)  AST (U/L)  ALT (U/L)  Prothrombin time (> 16s)  CK–total (U/L)  CK-MB (U/L)  BUN (mmol/L)  Creatinine (umol/L)  Na+ (mmol/L)  K+ (mmol/L)  LDH (U/L)  Myoglobin (µg/L)  Cystatin C (mg/L)  D–Dimer (mg/L)  CRP (mg/L)  ESR (mm/h)  Procalcitonin (µg/L)  S. ferritin (μg/L)  IL–1β (ng/L)  IL–2 (U/L)  IL–6 (ng/L)  IL–6 (ng/L)  IL–8 (ng/L)  IL–10 (ng/L)  TNF–Alpha (ng/L)  NT–ProBNP (ng/L)  hs Tn I (ng/L) | |  |

The mean of the continuous parameters with and without the outcome were recorded (Table 1). The median values reported in studies were transformed into mean(2).

**Comparator(s)/control**

Absence of the exposures listed above

**Primary outcome(s)**

1. Mortality in patients confirmed to have COVID-19
2. Disease severity in patients with COVID-19

**Secondary outcome(s)**

No secondary outcomes will be assessed

**Method of outcome measurement**

**Risk of Mortality among SARS CoV-2 infection:**

Defined as death as measured in studies following confirmed Coronavirus infection.

**Odds of severe disease among SARS CoV-2 infection:**

Severe disease as defined by the American Thoracic Society guidelines for the treatment of Community-acquired Pneumonia (3) or the Chinese National Health Commission guidelines for the Treatment of Novel Coronavirus infection (4)

**Data extraction (selection and coding)**

**Selection of studies**

The articles retrieved from different databases will be imported into the Covidence platform for title/ abstract screening and full text screening. Each study will be independently screened by two reviewers based on the title and abstract of the articles obtained from the literature search.  Conflicts on screening will be resolved through consensus between the lead authors.

This will be followed by the full text screening of each article independently by two reviewers. Conflicts on full-text screening will be resolved through consensus between the lead authors.

**Data extraction and management**

Data from each study, meeting our inclusion criteria will be extracted by two reviewers, who will gather information on patient outcome, comorbidities, presenting symptoms, admission laboratory markers, and pertinent radiological or other imaging information.

Disagreements will be resolved by ta third independent reviewer.

**Dealing with duplicate and companion publications**

The duplicate studies/articles will be automatically identified in the Covidence platform and be removed.

**Data from clinical trials registers and CSR**

We do not anticipate obtaining any clinical trial data on COVID-19 relevant to our research question.

**Risk of bias (quality) assessment**

**Assessment of risk bias in included studies**

Two authors will independently assess the risk of bias for each of the studies included for the analysis using the Newcastle Ottawa scale(5). Any discrepancy that is present will be resolved through discussion and/or by a third reviewer.

Each of the study will be assessed for the following:

*Selection*

1) Representativeness of the exposed cohort

2) Selection of the non-exposed cohort

3) Ascertainment of exposure

4) Demonstration that outcome of interest was not present at start of study

*Comparability*

1) Comparability of cohorts on the basis of the design or analysis

*Outcome*

1) Assessment of outcome

2) Was follow-up long enough for outcomes to occur

3) Adequacy of follow up of cohorts

**Data synthesis**

The primary outcomes of our study are mortality in patients admitted with COVID-19 infection and the development of Severe disease. We will classify studies according to the outcome measured and will analyse the results for each outcome separately.

Data on the study characteristics, source of funding, demographic characteristics, comorbidities, clinical symptoms, in-hospital complications, laboratory, and radiological features of the study participants will be extracted. Data on sex of the patient, smoking status, presence of comorbidities, clinical symptoms, in-hospital complications, and radiological features will be extracted as binary variables, while age will be treated as continuous data. For the laboratory parameters, data will be extracted in both continuous and categorical form (all reported cut-offs). The relevant information on time points for lab measurements, radiological evaluation and the disease severity assessment were extracted for the included studies. Continuous variables were recorded as median; and the mean from the studies were transformed to median for the analysis.

**Analysis**

Meta-analysis with random effects model will be performed to obtain pooled effect sizes for the outcomes of interest. The associations between binary predictors and mortality will be reported using pooled risk ratios (with 95% CI).  Odds ratios (with 95% CI) will be used to determine the association between the various factors and the presence of severe disease. All reported cut-offs from different studies will be synthesised in the meta-analysis as subgroups with an overall pooled risk estimates, assuming that different cut-offs (in continuous scale) do not greatly influence the effect size for a particular outcome. For continuous variables, meta-regression will be performed to assess the percentage change in mortality or the presence of severe disease with unit increase in the median laboratory value reported in the studies.  For the binary exposures, both unadjusted effect sizes and adjusted effected sizes (if adjusted for the same variables) will be extracted and used for analysis when feasible. All analyses are planned to be carried out using the meta package in Stata (StataCorp, version 16).

**Assessment of reporting biases**

Funnel plots and Egger’s test will be used to assess publication bias, small study effects and error in methodological design.

**Sensitivity analyses**

We plan to conduct sensitivity analyses by excluding low quality studies (NOS ≤ 5), and those that did not report the time points of assessment of laboratory, radiological and severe disease assessment. Sensitivity analyses will be performed to assess for quality of the study by doing a “low risk of bias” analysis that only includes studies that were deemed to be a low risk of bias.  Other decisions made during the review process will also be examined by a sensitivity analysis, to determine the impact(s) of these decisions.

**Analysis of subgroups or subsets**

The following characteristics may introduce clinical heterogeneity and we plan to perform subgroup analyses.

- Different laboratory cut offs

Further decisions regarding subgroup analysis, will be made based on the parameters deemed fit during the review process.

**Funding sources/sponsors**

There is no funding source for this review

**Conflicts of interest**

Nothing to declare

**Language**

English

**Subject index terms**

COVID-19; Coronavirus; SARS-CoV-2; 2019-nCoV; viral Coronavirus disease 2019; Severe acute respiratory syndrome coronavirus 2; clinical characteristics; co-morbidities; complications; laboratory; CT scan; computed tomography; radiology; risk factors; severe disease; survival; died; survived; inflammatory; coagulation
