# Supplementary material for: Factors associated with disease severity and mortality among patients with COVID-19: A systematic review and meta-analysis
Source: PLoS One. 2020 Nov 18;15(11):e0241541. doi: 10.1371/journal.pone.0241541 (PMC7673562; doi:10.1371/journal.pone.0241541)
Supplement: Attachment — Submitted filename: A. Response Letter for submission.docx [file pone.0241541.s004.docx]

Division of Pulmonary and Critical Care Medicine

Department of Medicine

5200 Eastern Avenue

Mason F. Lord Building, Center Tower, 3rd Floor

Baltimore, **MD** 21224

PLOS ONE

Editor-in-Chief

Editorial Office

October 11, 2020

Dear Editor:

Thank you for the opportunity to submit our revised manuscript, entitled “**Factors Associated with Disease Severity and Mortality among Patients with Coronavirus Disease 2019: A Systematic Review and Meta-Analysis**,” to PLOS ONE.

Here are our responses to the Reviewer’s comments:

To the Editor:

1) We have added the corresponding author’s ORCID ID.
2) Tables were added to the manuscript.
3) We have added captions for the figures, and are embedded in the manuscript.
4) All supplemental material in the attached PDF have tables and figures with titles.

To Reviewer #1:

A very interesting systematic review article to read and it highlights some important findings. Some concerns that need to addressed.
1. “We did not include articles uploaded in the preprint servers, as they are not peer reviewed and the findings may not be reliable”
The pre-prints serve as a source of unpublished data and if the authors have planned to not include unpublished data, they should state it

*Response: We have re-worded the statement as such:*

*We only included article published in peer-reviewed academic journals; we did not include articles uploaded in the preprint servers, as they are not peer reviewed and the findings may not be reliable(10).*

2. “Data extraction was performed independently by at least two of the authors (VC, NT, WH, MM, RS, AK, AH, II, AN, SA, ES, MS, AS, KW, and TP) and conflicts were resolved by a consensus between two authors (MM and VC)”

Were these two authors acting as arbitors ? If so please mention it.

*Response: An important question to address; these authors were acting as arbitors and mentioned as such in the manuscript.*

3. “The risk assessment for bias for all the studies included in this review was performed using the Newcastle-Ottawa quality assessment scale (NOS) for observational studies”

Please mention that you used the NOS for cohort studies

*Response: We have added that language into the manuscript.*


4. The protocol registration number is missing. If it was registered apriori?

*Response: The protocol was not registered.*

We believe this revised manuscript will be of great value to your readership.

We affirm that this work has not been published, accepted for publication, or under editorial review elsewhere. We grant the Journal full access to all aspects of the research and writing process. We, the authors, have no disclosures or financial conflicts of interest relevant to this manuscript to report. All authors contributed to the writing, editing, and content of this manuscript. The images in this report have never been printed elsewhere.

Sincerely,

Panagis Galiatsatos, M.D., M.H.S.

Johns Hopkins Medicine

pgaliat1@jhmi.edu
